# Regioisomerism vs Conformation: Impact of Molecular Design on the Emission Pathway in Organic Light-Emitting Device Emitters

**DOI:** 10.1021/acsami.3c19212

**Published:** 2024-04-26

**Authors:** Prasannamani Govindharaj, Aleksandra J. Wierzba, Karolina Kęska, Michał Andrzej Kochman, Gabriela Wiosna-Sałyga, Adam Kubas, Przemysław Data, Marcin Lindner

**Affiliations:** † Institute of Organic Chemistry, Polish Academy of Sciences, Kasprzaka 44/52, 01-224 Warsaw, Poland; ‡ Department of Molecular Physics, Faculty of Chemistry, Łódź University of Technology, Stefana Żeromskiego 114, 90-543 Łódź, Poland; § Institute of Physical Chemistry, Polish Academy of Sciences, Kasprzaka 44/52, 01-224 Warsaw, Poland

**Keywords:** TADF, RTP, S-T inversion, regioisomerism, OLED, TICT, charge transfer

## Abstract

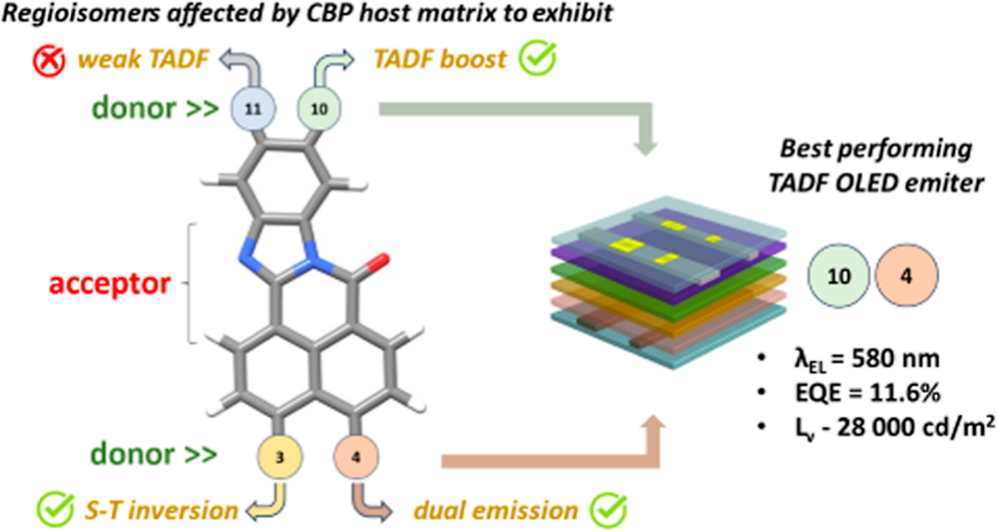

Despite the design and proposal of several new structural motifs as thermally activated delayed fluorescent (TADF) emitters for organic light-emitting device (OLED) applications, the nature of their interaction with the host matrix in the emissive layer of the device and their influence on observed photophysical outputs remain unclear. To address this issue, we present, for the first time, the use of up to four regioisomers bearing a donor–acceptor–donor electronic structure based on the desymmetrized naphthalene benzimidazole scaffold, equipped with various electron-donating units and possessing distinguished conformational lability. Quantum chemical calculations allow us to identify the most favorable conformations adopted by the electron-rich groups across the entire pool of regioisomers. These conformations were then compared with conformational changes caused by the interaction of the emitter with the Zeonex and 4,4′-bis(*N*-carbazolyl)-1,1′-biphenyl (CBP) matrices, and the correlation with observed photophysics was monitored by UV–vis absorption and steady-state photoluminescence spectra, combined with time-resolved spectroscopic techniques. Importantly, a CBP matrix was found to have a significant impact on the conformational change of regioisomers, leading to unique TADF emission mechanisms that encompass dual emission and inversion of the singlet–triplet excited-state energies and result in the enhancement of TADF efficiency. As a proof of concept, regioisomers with optimal donor positions were utilized to fabricate an OLED, revealing, with the best-performing dye, an external quantum emission of 11.6%, accompanied by remarkable luminance (28,000 cd/m^2^). These observations lay the groundwork for a better understanding of the role of the host matrix. In the long term, this new knowledge can lead to predicting the influence of the host matrix and adopting the structure of the emitter in a way that allows the development of highly efficient and efficient OLEDs.

## 1 Introduction

**Figure 1:**
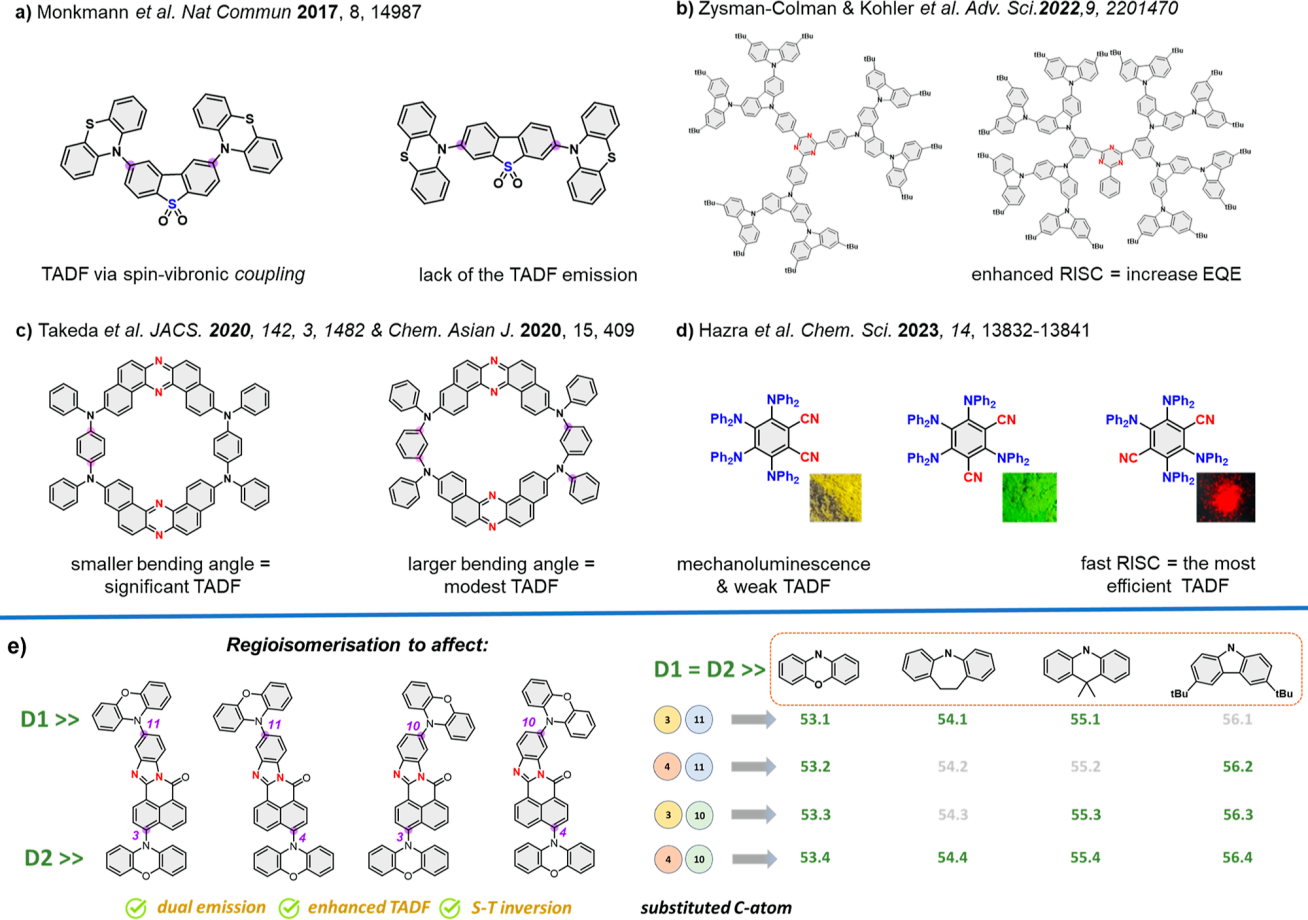
(a–d) Molecular structures of selected regioisomeric emitters with respect to their emissive behaviors (pictures in (d) represent the color of the emission). The structures (a) and (b) were reproduced with permission from ref (20) (copyright 2017, Nature Portfolio) and ref (22) (copyright 2022, Wiley-VCH), respectively. The first structure in (c) was reproduced from ref (25) (copyright 2020, American Chemical Society) and the second one was reproduced with permission from ref (26) (copyright 2020, Wiley-VCH). Structures in (d) were adapted from ref (27), available under a CC-BY-NC license, copyright 2023, Hazra et al. (e) General structure of regioisomeric emitters based on bis-substituted NBIs (with four different variants: 3/4/11/10) (green represents isolated isomers, while gray represents those that were not isolated in a pure form). Curved arrows point out the impact of the specific position on the observed photophysical phenomenon. For the sake of clarity, D1 and D2 are denoted as donors, while A is the acceptor.

In recent years, a tremendous growth of research toward luminescent display and lighting technologies based on organic light-emitting devices (OLEDs)^1^ has been motivated by their low production costs, ease of processing, superior color purity, and performance.^2,3^ Additionally, OLEDs have provided a significant platform in photodynamic therapy,^4,5^ sensing technologies,^6^ and wearable electronics,^7^ among other applications.^8^ In this context, organic molecules that exhibit a phenomenon of thermally activated delayed fluorescence (TADF) can revolutionize the domain of OLED emitters as they are capable of harvesting both singlet and triplet excitons, giving, theoretically, 100% of internal quantum efficiency (IQE).^9^ When the energy difference between the singlet–triplet excited states (Δ*E*_ST_) is sufficiently small and spin–orbit coupling (SOC) is nonzero, triplet excitons can transition back to the singlet energy level through thermally activated reverse intersystem crossing (RISC). After the singlet energy level is populated, triplet excitons efficiently relax to the ground state. Therefore, there has been swift progress in the development of molecular engineering aimed at narrowing the Δ*E*_ST_ energy gap.^10−16^ To realize this scenario, variable donor–acceptor–donor (D–A–D) architectures have been examined as their twisted electron-rich moieties can increase the dihedral angle(s) between D–A units. The implementation of such electronic structures provokes intermolecular charge transfer, for which second-order spin-vibronic SOC promotes efficient RISC. Under these circumstances, the role of regioisomers can be vitally important for the properties of the singlet and triplet excited states.^17−19^ Such minimal structural changes (without any modifications of functional groups) also underscore the impact of the topology of the π-conjugated scaffold on the mechanism of emission, the efficiency of electroluminescence, and stability under the current applied. The development of regioisomers to enhance TADF functionalities is still in progress and is limited to a select few structural frameworks. Among the systems revealed so far, Monkman and co-workers^20^ reported on 2,8- and 3,7-bis(10*H*-phenothiazin-10-yl)dibenzo[*b*,*d*]thiophene-*S*,*S*-dioxide, for which efficient TADF via spin-vibronic coupling was noticed (2,8-DPTZ-DBTO_2_) with a maximum external quantum emission of (EQE_max_) of 7%, whereas TADF was not observed for its 3,7-DPTZ-DBTO_2_ counterpart, as marked in Figure 1a. The groups of Ifor and Zysman-Colman shifted their focus from small-molecule emitters^21^ to the regioisomeric G2 and G3 dendrimers (Figure 1b) to assess their suitability as solution-processed, host-free, TADF OLED emitters.^22,23^ In this context, the tris(phenyl)triazine core was branched with multiple [bis(3,6-ditBucarbazole)Cz] units at either one (tBuCz2 mTRZ) or two (tBuCz4 mTRZ) meta-positions of the phenylene linker. Comparative studies with the previously reported para-decorated analogue (tBuCz3pTRZ)^24^ revealed the superiority of asymmetric units. This superiority was evident in the remarkable OLED performance achieved using tBuCz2 mTRZ (EQE_max_ = 19.9%) as well as tBuCz4 mTRZ (EQE_max_ = 23.8%) as emitting systems. This correlation was rationalized by better control over the reorganization energies, leading to an enhancement of the RISC process. The impact of the substitution pattern on the photophysics of TADF emitters was also faced by Takeda and Data, who put an emphasis on macrocyclic emitters with a structure composed of DBPHZ and diphenyl amines, forming a saddle-like structure. As demonstrated in Figure 1c, their phenylene linkers were bridged in the *para* (p-1 DBPHZ)^25^ and *meta* (m-1 DBPHZ)^26^ positions to provide topological isomers. The comparison of their physicochemical properties disclosed a larger bending angle (and thus a shorter distance between amine and the A-unit) for m-1 DBPHZ. This was reflected in an increased Δ*E*_ST_ = 0.31 eV and much weaker TADF emission with respect to the *para* (p-1 DBPHZ) versus the *meta* (m-1 DBPHZ). The latter entity showed a narrow energy gap (Δ*E*_ST_ = 0.18 eV), leading to light production in the TADF OLED (λ_EL_ = 605 nm; EQE_max_ = 6.9%). Very recently, Hazra and co-workers^27^ reported on the examination of the 3-fold isomers (ortho/meta/para) of the well-established dicyanobenzene scaffold decorated with diphenylamine moieties. This investigation revealed significant variations not only in the Δ*E*_ST_ energy but also in distinct behaviors in the crystalline phase, resulting in distinguishable mechanoluminescence that ranged from green to orange emission exclusively for ortho isomers, which is attributed to its crystallization in the centrosymmetric *P*_21_/*C* space group. Consequently, there is a need for studies that would explore the relationship between even higher-order regioisomers (four different positions) and their photophysical properties, which can indeed be affected by the host matrix. Nonetheless, such a conceptually new molecular design has not yet been identified and investigated in the context of TADF emitters and their use in the OLEDs.

Thus, to lay their foundations, herein we demonstrate how the solid-state matrix can affect the molecular conformation of TADF emitters using the example of up to four regioisomers with a D–A–D electronic structure. A correlation between the occupied positions of donors and their photophysical output reveals unique emissive behaviors, manifested by enhanced TADF, dual emission, and inversion of singlet–triplet excited states energy levels, as underpinned in Figure 1e. As clear evidence, regioisomers were further explored for OLED applications as TADF emitters, showing that these optimal donor positions exhibited an EQE as high as 11.6% with a remarkable luminance of 28,000 cd/m^2^, while suboptimal regioisomers disclosed undesired aggregation and room-temperature phosphorescence (RTP).

## 2 Results and Discussion

### 2.1 Synthesis

To study the TADF phenomenon, in the solid state, with higher-order regioisomers, the naphthalene benzimidazole (NBI) scaffold can be conceived as the ideal molecular candidate and is envisioned to have a high degree of symmetry. Building on that, we designed and synthesized a bis-substituted NBI which can be functionalized with various electron-rich amines and form four isomers with the D–A–D electronic structure (Figure 1e). Along this line, we successfully synthesized and isolated the entire set of phenoxazine-based (PXZ) D–A–D compounds (**53.1-4**); three out of the four were obtained for 9,9′-dimethylacridine (DMAc, **55.1** and **55.3-4**) and 3,6-ditertbutyl-carbazole (tBuCz) derivatives (**56.2-4**), and two out of the four were obtained for the iminodibenzyl (IDB) family (**55.1** and **55.4**). All of the obtained dyes were subsequently investigated for their photophysical and electroluminescent behaviors, theoretically and experimentally. The detailed synthetic process for D–A–D emitters decorated with electron-rich moieties (including PXZ, DMAC, tBuCz, and IDB) in positions 3,11, 4,11, 3,10, and 4,10-NBI is outlined in Scheme S1 (Supporting Information). The regioisomeric emitters were assembled within two scalable synthetic steps comprising acid-catalyzed condensation of 4-bromo naphthalene imide and 3-bromo-1,2-diaminobenzene, followed by Buchwald–Hartwig amination with electron-rich moieties. A mixture of isomers were isolated via common silica gel column chromatographic separation and characterized by a combination of ^1^H, ^13^C, and 2D NMR spectroscopies along with high-resolution mass spectrometry (ESI), which helped to unambiguously confirm the identity and purity of the obtained dyes. For the sake of clarity, we combined and displayed the structures of all isolated isomers as well as these four, which were obtained as a mixture of isomers, in Supporting Information (Figure S22).

### 2.2 Electronic Structure Calculations

**Figure 2:**
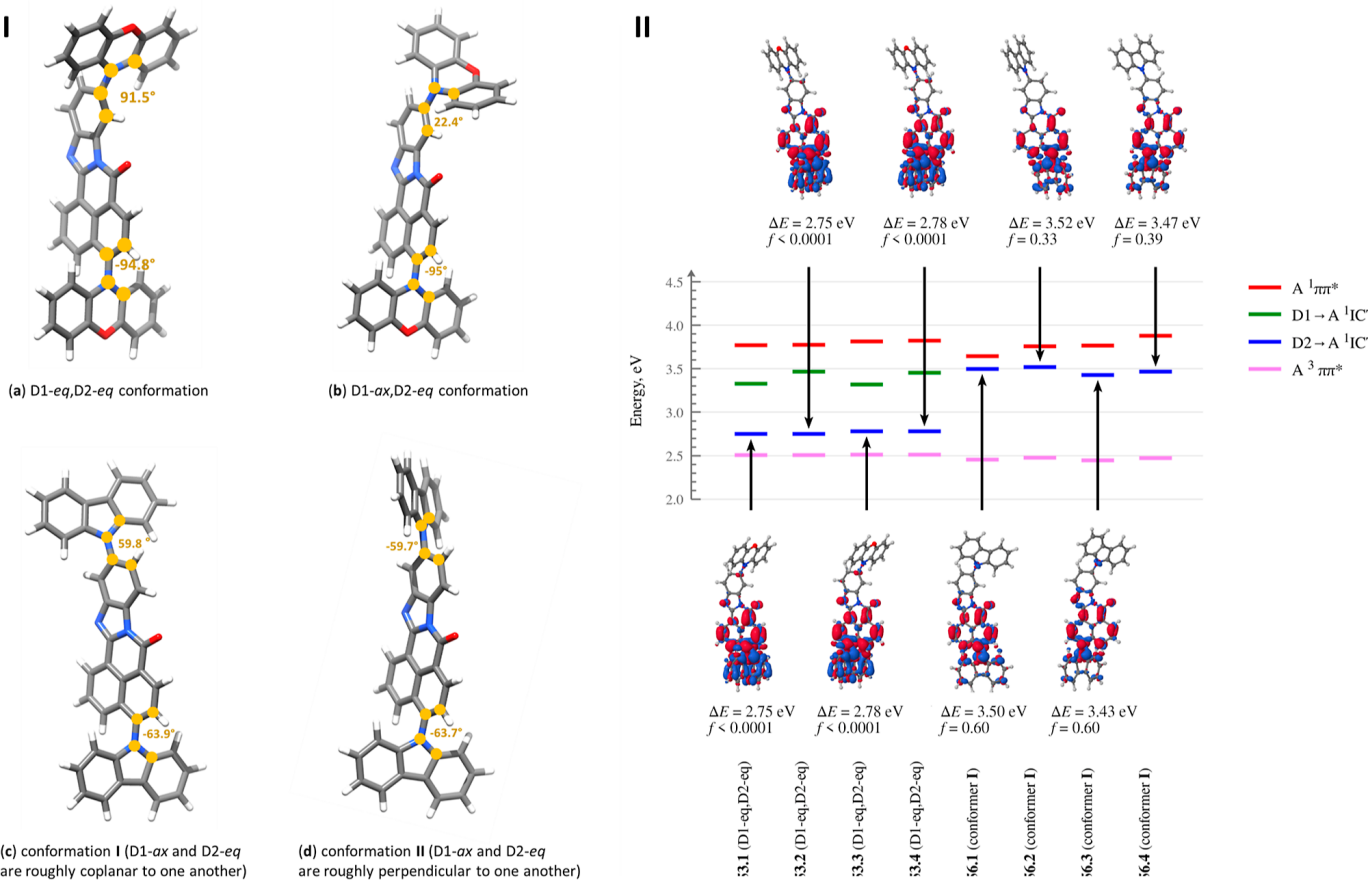
(I) DFT-optimized conformation of selected dyes manifested by marked dihedral angles (a–d); (II) EDDMs for the lowest few singlets and the triplet of compound 53.1 in the D1-a, D2-a conformation. The EDDMs are plotted in the form of isosurfaces with isovalues of ±0.0025 e/a03. The red and blue isosurfaces delimit regions in which the electron density is increased and decreased, respectively, relative to the ground state.

Recent computational research has revealed the significant impact of conformational motion, particularly in the electron-rich units of TADF emitters.^28−31^ This motion, characterized by the distribution of the dihedral angle,^32,33^ plays a crucial role in influencing both the S–T energy gap and the oscillator strength. This is essential for establishing a high radiative decay of the delayed fluorescence (DF) emission. In light of these considerations, our studies began with the implementation of density functional theory (DFT) calculations to identify predominant conformation preferences across a set of regioisomeric emitters. For the sake of convenience, we conducted these calculations using two representative series of molecules, namely, **53.1**-**4** and **55.1**-**4**, whose structures are illustrated in Figure 2. As each of the fluorophores under study has two identical electron-donating moieties (one at position 11 or 10, and another either at position 4 or 3), we denote the former moiety as **D1** and the latter as **D2**. The fused 1,8-NBI electron-accepting moiety is denoted as **A** (see Figure 2) for the sake of clarity. In the ground electronic state of the **53.X** series, the PXZ electron-donating moieties D1 and D2 can potentially adopt quasi-equatorial (eq) or quasi-axial (ax) orientations with respect to the A moiety. Thus, each of these compounds can potentially exist in four conformations arising from the different orientations of the two electron-donating moieties, i.e., D1-eq, D2-eq; D1-eq, D2-ax; D1-ax, D2-eq; and D1-ax, D2-ax. Regarding the D1 moiety, geometry optimizations confirm the existence of minima on the potential energy surface (PES) of the ground state that correspond to D1-eq and D1-ax conformations. In the case of the D2 moiety, however, we found that only a D2-ax conformation corresponds to a minimum on the ground-state PES. Attempts to optimize a D2-ax conformation result in the D2 moiety relaxing spontaneously toward a quasi-equatorial conformation. The inability of the D2 moiety to adopt a quasi-axial conformation is presumably due to steric interactions with the **A** fragment. It must be thus stressed that each compound in the **53.X** series has only two stable conformations: D1-eq, D2-eq and D1-ax, D2-eq (Figure 2a,b), which are manifested by the corresponding dihedral angles to be 91.5 and 22.4° (for D1, respectively) and −94.8 and −95° (analogously for D2). Notably, the populations of the D1-ax, D2-eq conformers are expected to be on the order of 0.01. As an illustration, Figure 2I shows the D1-eq, D2-eq and D1-ax, D2-eq conformers of compound **53.1**. For all four compounds in the **53.X** series, the D1-eq, D2-eq conformation is lower in energy than the D1-ax, D2-eq conformation by around 20 kJ/mol. We therefore conclude that the D1-eq, D2-eq conformation is the dominant form of all four compounds.

In regard to the **56.X** series, the Cz moieties are less bulky than the PXZ moieties. Consequently, all compounds in this series adopt equatorial-like conformations in which the Cz moieties are slightly twisted with respect to the plane of the A moiety. More specifically, each compound has two inequivalent equatorial conformations that differ in the relative orientation of the D1 and D2 moieties. For reference, the two possible conformations of compound **56.2** are illustrated in Figure 2I. In conformation **I** (Figure 2c), the D1 and D2 moieties are roughly coplanar (judging by their mutual orientation), whereas in conformation **II** (Figure 2d), they are roughly perpendicular. For each compound, the two equatorial conformations lie very close in energy and are also found to have similar electronic excitation spectra. This is because the D1 and D2 moieties are separated from one another by the intervening A moiety, and their orientation relative to one another makes little difference, as demonstrated through the value of the corresponding dihedral angles (Figure 2c,d). In the solution phase, conformers **I** and **II** are expected to coexist in roughly equal abundances.

#### 2.2.1 Electronic Excitation Spectra

Having investigated the conformational preference of the 53.X and 56.X series of compounds, we moved on to examine their electronic excitation spectra. The main results of the calculations—the vertical excitation energies and the electronic structures of the low-lying excited states—are presented visually in Figure 2, **II**. A more detailed breakdown of the vertical excitation spectrum of each compound is reported in Table S1 and Figures S1 and S2 (Supporting Information). For all four **53.X** compounds in this series, the lowest singlet excited state is found at an energy of roughly 2.7–2.8 eV (the exact energy depends on the specific compound in the series). This state arises from intramolecular charge transfer (ICT) from the D2 moiety onto the 1,8-NBI-like fragment of the A moiety (D2 → A ^1^ICT). As an illustration of the electronic structure of the D2 → A1 ICT state of each compound, Figure 2 shows plots of the electron density difference maps (EDDMs). The energy of the D2 → A ^1^ICT state is rather insensitive to the positions of electron-donating moieties (D1 and D2) as the S_0_ → S_1_ vertical excitation energies for all four compounds in **53.X** fall within a narrow energy range. The lack of sensitivity to the position of the D1 moiety is to be expected as that moiety is not involved in this ICT state. More surprising is the insensitivity of the energy of the D2 → A ^1^ICT state to the position of the D2 moiety. Intuitively, one would expect that the energy of an ICT state should be sensitive to the geometry of the linkage between the electron-donating and -accepting moieties, and there are several examples in the literature where this is indeed the case.^34,35^ For this reason, we do not rule out the possibility that the lack of sensitivity of the energy of the D2 → A ^1^ICT to the position of the D1 moiety is an artifact of the time-dependent DFT (TDDFT) calculations. It would follow that compounds in the **53.X** series may differ from one another substantially in terms of their photophysics. Another singlet ICT state, which corresponds to a shift of electron density from the D1 moiety onto the A moiety (D1 → A ^1^ICT), is found a few tenths of an electron Volt above the D2 → A ^1^ICT state. The energy of the D1 → A ^1^ICT state is somewhat sensitive to the position of the D1 group but not to the position of the D2 group. The two low-energy ICT states of the **53.X** series of compounds exhibit very low oscillator strengths. This is because of the quasi-equatorial orientations of the D1 and D2 moieties—the occupied and virtual orbitals involved in either ICT state show very little spatial overlap with one another. Absorption by these ICT states may be responsible for the broad, weak bands seen in the photoabsorption spectra of the **53.X** series in the range of around 450–550 nm (which corresponds to photon energies in the range of around 2.2–2.8 eV). Moreover, for each compound in the **53.X** series, we find a bright ^1^ππ*-type excited state at an energy of roughly 3.8 eV. This state is largely localized on the A moiety. Accordingly, we denote it as the A ^1^ππ* state. The vertical excitation energy into the A ^1^ππ* state is insensitive to the positions of the D1 and D2 moieties, which are not involved in this state. The transition into this state gives rise to intense photoabsorption bands of these compounds near 375 nm (which corresponds to a photon energy of around 3.3 eV). Regarding the triplet states for each compound in the **53.X** series, the lowest triplet state is a ^3^ππ*-type state at an energy of roughly 2.5 eV that is localized on the A moiety. Its energy is insensitive to the positions of the D1 and D2 moieties. The second-lowest triplet state of each compound is the triplet D2 → A ^3^ICT state, located at an energy of roughly 2.7–2.8 eV. As with its singlet counterpart, the energy of this state is calculated to be insensitive to the positions of the D1 and D2 moieties. We now move on to the **56.X** series of compounds in which D1 and D2 are Cz moieties. The calculated vertical excitation spectra are shown on the right side of Figure 2. The full calculated results are relegated to Table S2 in the Supporting Information. For the sake of brevity, we only include the spectrum of conformer **I** of each compound; the spectrum of conformer **II** is very similar to that of conformer I. Since Cz is a relatively weaker electron-donating moiety than PXZ, in the **56.X** series of compounds, the ICT states—both singlet and triplet—are found to be higher in energy than those in the **53.X** series. Another important difference is that in the **56.X** series, both the D1 and D2 moieties adopt equatorial-like orientations. As the D1 and D2 moieties are only slightly skewed with respect to the plane of the A moiety, their π- and π*-type orbitals overlap well with those of the A moiety. Consequently, some of the D1 → A and D2 → A ^1^ICT states of the **56.X** series of compounds have a substantial admixture of ^1^ππ* character, and they exhibit high oscillator strengths for excitation from the ground state. In each compound in the 56.X series, the lowest singlet excited state is the D2 → A ^1^ICT state, at an energy of roughly 3.4–3.5 eV (depending on the specific compound in the series). Its energy is insensitive to the position of the D1 and D2 moieties. Slightly higher in energy, at around 3.6–3.8 eV, we find a ^1^ππ*-type state that is localized on the A moiety (A ^1^ππ*). Both of these states exhibit large oscillator strengths, and they are responsible for the first photoabsorption band of the given compound in the range of around 400–500 nm (which corresponds to photon energies in the range of around 2.5–3.1 eV). In the triplet manifold, the lowest state is a ^3^ππ*-type state that is localized on the A moiety at an energy of roughly 2.4–2.5 eV. This state is well-separated in energy from the higher triplet states, which are found at energies of roughly 3.2 eV and higher. Based on the above theoretical calculations, it is reasonable to envision that the pool of NBI isomers has the potential to exhibit distinguishing behavior in the TADF emission process.

### 2.3 Steady-State Photophysics

**Figure 3:**
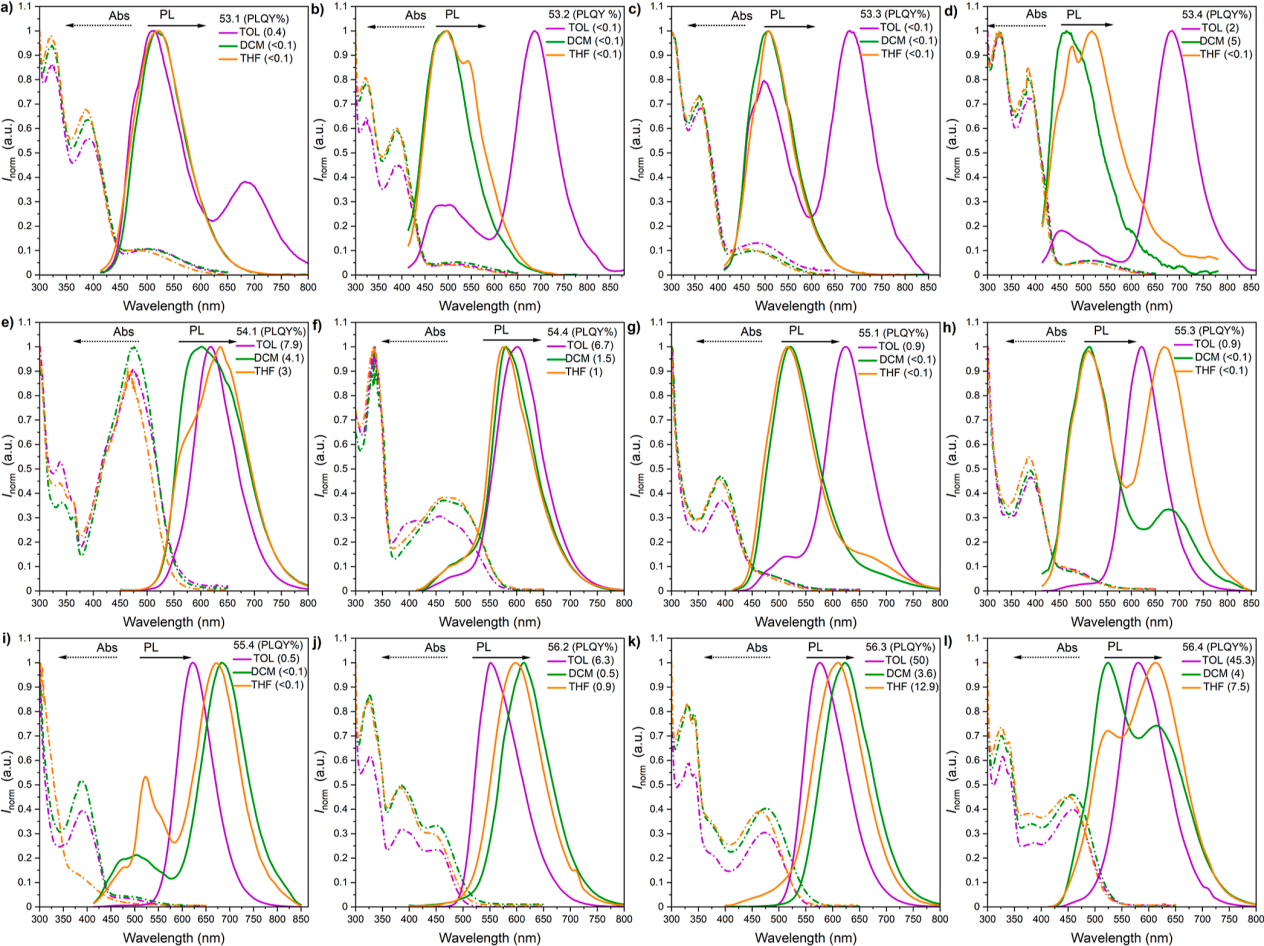
Absorption and emission spectra (a–l) of synthesized isomers in different solvents.

To experimentally approach the effect of regioisomerism on the in-depth photophysics of D–A–D compounds, UV–vis absorption and steady-state photoluminescence (PL) spectra of diluted solutions of all of the compounds were measured. The corresponding spectra are shown in Figure 3, while all experimental details are combined in Table 1. An absorbance peak at higher energy is due to the intense π–π* transitions. Conclusively, the impact of the regioisomeric effect is even more pronounced in the emission spectra of the solutions. Notably, all investigated isomers show quite complicated PL emissions, particularly visible in nonpolar solvents (toluene). For the set of **53.X** isomers, two broad and structureless emission bands were observed. More precisely, the emission bands exhibited by the isomers are as follows: the (i) 3,11 isomer—**53.1**: 512 nm (green) and 681 nm (red); (ii) 4,11 isomer—**53.2**: 485 nm (cyan) and 686 nm (red); (iii) 3,10 isomer—**53.3**: 497 nm (cyan) and 680 nm( red); and (iv) 4,10 isomer—**53.4**: 452 nm (blue) and 684 nm (red), which clearly indicates that all four phenoxazine isomers of **53.X** could appear in a dual conformational form (D1eq–D2eq and D1eq–D2ax). While switching solvent media to a more polar one, the dominant emission occurred in the range 470–550 nm, strictly due to the two possible D1eq–D2ax conformations of the phenoxazine moiety. These experimental results^36−38^ are in good agreement with the TDDFT results (vide supra) and represent confirmation of two conformers. In the case of the **54.X** sets, **54.4** also exhibited dual emission in all solvents used, this time caused by the flexibility of the dibenzoazepine unit that permits different conformations.^39,40^ The dual CT emission can be ascribed to the mixture of nearly planar or orthogonal conformers. These two different conformers exhibited two different emission peaks with a heterogeneous ratio of intensity, showcasing the most stabilized conformers among them. Considering the origin of the peak at 420–525 nm, a very weak emission was found with a weak CT character (which differs from the pure azepine donor, ∼320–400 nm). Consequently, this is associated with a less stable, nearly planar conformation, while the next one (525–800 nm) with another CT state comes from the second nearly orthogonal conformation of azepine. In contrast, **54.1** exhibited a very broad single CT emission in nonpolar solvents (toluene), whereas in a polar solvent, the peak between 500 and 575 nm is probably due to a mixture of two different conformers. The 55.X sets bearing DMAC as a donor part are pseudoplanar segments and exhibit two possible conformations: a planar (1) and a crooked (2) form. Accordingly, the first (1) arguably occurs at 500–840 nm in polar solvents (DCM and THF) and at 550–800 nm in nonpolar solvents (toluene), while the second (2) occurs at 425–580 and 450–550 nm, respectively. Regarding **55.3** and **55.4** in polar solvents, dual emissions clearly appeared in a nonhomogeneous order. In nonpolar solvents, very weak emission (*E*_1_) from the crooked form appeared only in **55.3** and **55.1**, whereas in the case of **55.4**, only *E*_2_ emission due to the planar conformation was observed (Figure 3).

**Table 1:** Summary of Steady-State Photophysical Data of Diluted Solutions of Compounds^a^

compounds	solvents	λ_abs_ (nm)	λ_PL_ (nm)	Φ_PL_^b^
**53.1**	TOL	323,391,509	512 and 681	0.4
	DCM	324,390,496	519	<0.01
	THF	323,359,468	523	<0.01
**53.2**	TOL	391,384,522	485 and 686	<0.01
	DCM	324,389,522	496	<0.01
	THF	322,385,510	497	<0.01
**53.3**	TOL	363,487	497 and 680	<0.01
	DCM	356,472	507	<0.01
	THF	358,469	508	<0.01
**53.4**	TOL	325,391,517	452 and 685	2.0
	DCM	325,383,516	464	5.0
	THF	322,383,500	518	<0.01
**54.1**	TOL	338,475	618	7.9
	DCM	338,477	601	4.1
	THF	338,469	637	2.0
**54.4**	TOL	335,455	600	6.7
	DCM	335,462	580	1.5
	THF	336,472	575	1.0
**55.1**	TOL	393,490	624	0.9
	DCM	390,490	521	<0.01
	THF	389,489	517	<0.01
**55.3**	TOL	390,482	621	0.9
	DCM	389,481	501	<0.01
	THF	385,480	608	<0.01
**55.4**	TOL	390	622	0.5
	DCM	389	683	<0.01
	THF	375	672	<0.01
**56.2**	TOL	327,385,454	552	6.3
	DCM	326,381,451	613	0.5
	THF	326,381,453	598	0.9
**56.3**	TOL	330,344,471	576	49.8
	DCM	330,342,476	623	3.6
	THF	330,343,464	611	12.9
**56.4**	TOL	328,340,460	580	45.3
	DCM	326, 341,457	525 and 612	3.98
	THF	327,341,449	524 and 613	7.5

a Solution concentration: 10^–5^ M; determined with an integrated sphere in a normal atmosphere.

b PLQY given in %.

This difference in peak inhomogeneity indicates that **55.1** has a comparatively stronger ICT interaction between the acceptor and donors.^41^

In the **56.X** series in a polar solvent, **56.4** and **56.3** exhibited dual emission, most likely related to the formation of aggregates. All these results indicate that the steady-state photophysics of all the series are strongly affected by regioisomerism as well as conformational isomerism from the donor moieties. Once it is pronounced in a solution, it is worth recognizing the nature of the interaction of regioisomers with the host matrix in the solid state.

### 2.4 Time-Resolved Spectroscopic Analysis

**Figure 4:**
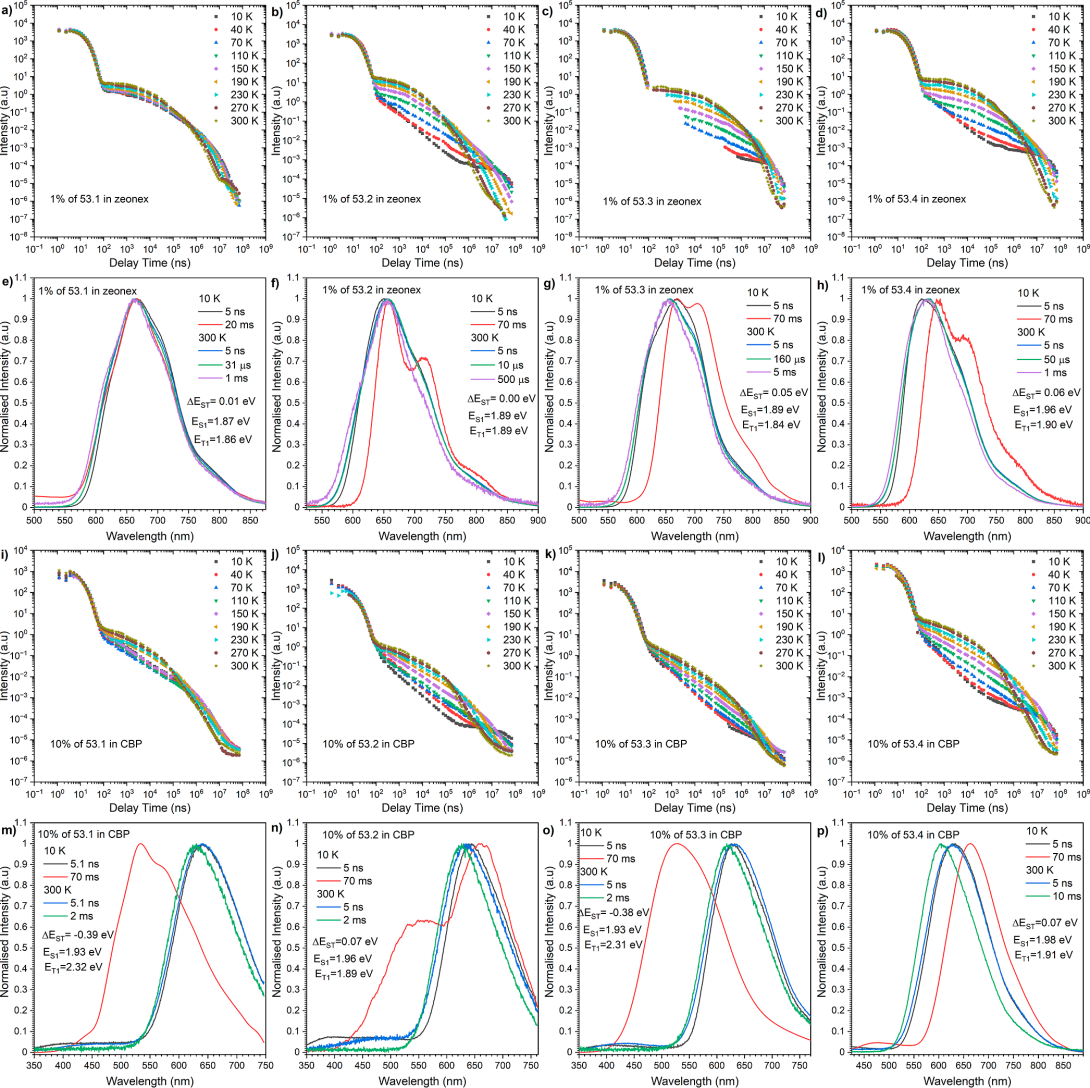
Time-resolved PL decay profiles (intensity vs delay time) (a–d and i–l) and spectra (e–h and m–p) of compounds 53.1–53.4 in Zeonex (a–h) and CBP (i–p). The energies correspond to the maximum emission peaks and λ_ex_ = 355 nm.

**Figure 5:**
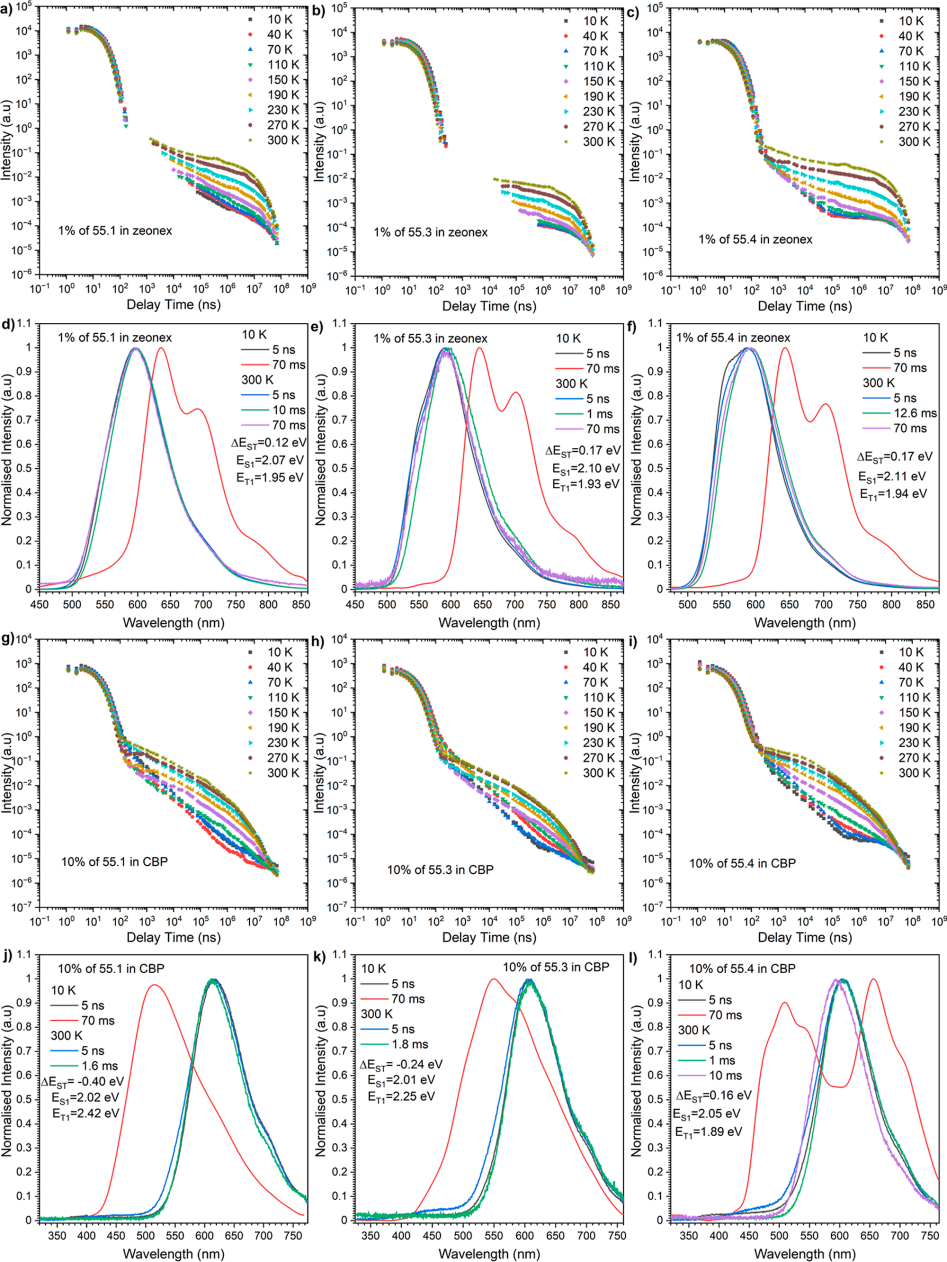
Time-resolved PL decay profiles (intensity vs delay time) (a–c and g–i) and spectra (d–f and j–i) of compounds 55.1–55.4 in Zeonex (a–f) and CBP (g–l). The energies correspond to the maximum emission peaks and λ_ex_ = 355 nm.

To comprehend the detailed photophysics of the DF process of all the isomers, we utilized time-resolved spectroscopic techniques, and therefore, the obtained data are collected in Table 2. The emissive properties of the entire set of dyes were analyzed in the nonpolar, Zeonex, as well as in the polar 4,4′-bis(*N*-carbazolyl)-1,1′-biphenyl (CBP), as the host matrices. Interestingly, likewise in solvents, the significant impact of the conformation of emitters was observed (as the presence of both quasi-axial and quasi-equatorial conformations was noticed), and depending on the host, we could identify some similarities and discrepancies. In the solid matrix, this rotation can be completely impeded by bulky donors and matrix rigidity. On the contrary, in solvents, the entire population of isomers exhibited dual emission from different conformers due to the increased flexibility in this environment. To compare the effect of regioisomers on DF properties, we considered comparing whole sets of isomers as the performed measurements revealed the impact on the photophysics to arise not only from the molecular structure itself but also from the host materials. This phenomenon is well-evidenced for compounds **53.X** in Zeonex (Figure 4a–h), for which the isomeric effect barely influences the singlet ^1^CT state, whereas the triplet state (both ^3^LE and ^3^CT) is significantly affected. This resulted in very small energy Δ*E*_ST_ gaps, which are more than sufficient to trigger the TADF process via RISC transition. It must also be underpinned due to the fact that different isomeric positions lead to changes in electron density on asymmetric acceptors and donors. This affects the ^3^LE and ^3^CT contributions in the overall T_1_ state. When it comes to **53.1** and **53.3**, a greater contribution from ^3^CT was observed, hinting at a potential change in the dihedral angle formed by the donor merged at position 3 of the NBI scaffold in comparison to site 4. Alternatively, this effect could be attributed to the mixture of different conformers (Figure 4e–h, red spectra). The flexibility of the polymer can be beneficial from the viewpoint of TADF efficiency as it can promote the formation of the more favorable quasi-equatorial conformation, resulting in a very low Δ*E*_ST_ due to the ^1^CT energy being close to the triplet energy of the acceptor. Moreover, a significant observation was made while isomers were deposited with the CBP matrix. Although TADF-related emission was found, an unexpected shift of the phosphorescence spectra to higher energies occurred (in comparison to the Zeonex host). This process provided a negative singlet–triplet gap (Δ*E*_ST_) for isomers **53.1** and **53.3** in which the PXZ donor is substituted at position 3 (Figure 4m,o). For the remaining isomers of type, the dual phosphorescence is observed for isomers **53.2**, where the donor is at position 4 (Figure 4n), while in this fashion, there is no remarkable relationship between positions 10 and 11 of the donor and photophysical outputs. Truly, the experimentally determined inversion of the excited-state levels is not in line with the results of the theoretical calculation, which revealed that the lowest triplet excited-state energy should be the same or lower than that of the singlet excited state. On the other hand, the next triplet excited state is associated with the 3 or 4 position donor unit. We furthermore found the possibility of a conformational change from quasi-equatorial to quasi-axial at those positions. While it is tough to rationalize why the emission from the triplet excited state at the acceptor moiety is turned off, the observed phosphorescence emission from isomers **53.1** and **53.3** originates from a triplet excited state localized on the donor moiety at position 3. It is known that such behavior is associated with the quasi-axial conformation, where this particular donor unit is pushed over the acceptor surface and locked. This specific molecular organization acts as a steric hindrance and usually causes an increase of the energy of the singlet excited state (^1^CT), which is also accompanied by the RTP process.^42^ Nevertheless, in our case, the impact is completely different. While we still observe the TADF process, this can be associated with a twisted ICT (TICT) effect, commonly visible in multiresonance (MR) TADF emitters. Here, the presence of a large steric hindrance and fully decoupled HOMO–LUMO energy states do not allow for normal CT.^43,44^ We also notice a conformational impact in isomer **53.2**, where a mixture of two distinct conformers is observed in the solid state (Figure 4n). Even though the entire set of **53.X** isomers disclosed moderate emission in the solid state (Φ_P_ up to 22%), an evident contrast in TADF properties was observed among different regioisomers containing the PXZ donor. First of all, isomers with the donor moiety at position 4 have a higher TADF contribution (DF/PF) in comparison to those in position 3 (2.36 for 53.2 and 1.33 for **53.1**, respectively). Second, isomers with a donor group at position 10 have higher TADF contribution than that of the same group at position 11 (5.22 for **53.4** and 2.36 for **53.2**, respectively). This is associated with an increase of the radiative rate constant on position 10, and k_RISC_, which is important in order to increase the TADF contribution (Table 2). Interestingly, such distinctive differences were also determined in the remaining pool of IBZ, DMAc, and Cz isomers. Accordingly, for IDB derivatives **54.X**, unusual behavior was found for the Zeonex host, in which we do not observe delayed emission whatsoever, in contrast to the CBP host, for which delayed emission is observed but at very long times >5 ms (Figure S7). The unusual lack of this TADF or RTP emission can be correlated with the singlet excited-state energy that is similar to the localized triplet excited-state energy of ca. 1.9–2.0 eV. This should be related to the visible TADF emission. On the other hand, D–A derivatives based on the IDB donor are well-known for their quasi-axial conformation, which pushes emission to the RTP mechanism.^45,46^ Upon closer inspection of the behavior of isomers **54.1** and **54.4** in the CBP host, a small inversion was again observed, which straightforwardly supports the concept of the impact of quasi-axial conformation and TICT mechanisms. If the presence of the quasi-equatorial conformation were the case, the typical TADF mechanism would be observed. When it comes to regioisomers with DMAC-based donors (**55.X**), they exhibit a similar behavior as the above-mentioned **53.X** series. In a Zeonex matrix, although there is a higher Δ*E*_ST_ gap, the TADF emission is still visible (Figure 5a–f). In a CBP host, we observed Δ*E*_ST_ gap inversion for the same donor at position 3 and a mixture of the phosphoresce emission for position 4, which is again caused by the quasi-axial conformation (Figure 5j–l). In a similar fashion to the isomers from the **53.X** series, the highest DF contribution was measured for derivatives with donors at positions 10 and 4 (**55.4** DF/PF = 4.43), but in this case, not only position 10 contributed to the increase of k_RISC_ but also position 4 (Table 2). In turn, a clear observation of DF emission with a thermally activated process is made when the PH emission disappears along with increasing temperature, and once again, emission from the S_1_ state becomes prominent. Regarding dyes **54.X** and **56.X** in Zeonex, only **56.2** exhibited a delayed component at room temperature, while the other isomers showed prompt fluorescence (PF) exclusively. For **56.2** in Zeonex (Figure S8d), a mixed contribution of minor TADF (500–600 nm) and dominant RTP (600–850 nm) was observed due to higher energy splitting compared to that of **53.X** and **55.X**. The presence of PF alone in the **54.X** set is attributed to the nonemissive nature of T_1_ at both low and high temperatures (Figure S7a–f). While switching from a Zeonex to a CBP host, we observed an intriguing behavior in dyes **53.1**, **53.3**, **55.1**, and **55.3**, in which a negative Δ*E*_ST_ gap was noted (Table 2 and Figures 2m,o and 3j,k). An inversion of the Δ*E*_ST_ gap is reasoned by emission from either a different triplet state (above the T_1_ state, often denoted as T_*n*_ where *n* > 1) or E_S1_ < E_T1_. In this class of isomers, the presence of a higher triplet energy (E_T1_) and lower singlet energy (E_S1_) results in a substantial inversion of the Δ*E*_ST_ gap (>−0.20 eV).

**Table 2:** Summary of the General Photophysical Properties Obtained from Time-Resolved Spectra

dye	λ_em_ [nm]^a^	host	Φ_PL_^b^	τ_PF_ [ns]^c^	τ_DF_ [μs]^d^	τ_RTP_ [ms]^e^	DE/PF^f^	*k*_r_ 10^7^ s^–1^^g^	*k*_nr_ 10^8^ s^–1^^g^	*k*_ISC_ 10^6^ s^–1^^h^	*k*_RISC_ 10^5^ s^–1^^h^	*E*_a_ [eV]^i^	S_1_ [eV]^j^	T_1_ [eV]^j^	Δ*E*_ST_ [eV]^k^
**53.1**	664	Zeonex	4.5	13.63 ± 1.15	25.16 ± 2.96		1.81	1.17	7.01	4.73	0.11	0.0057	1.87	1.85	0.02
	642	CBP	3.3	8.92 ± 1.02	3.30 ± 0.37		1.33	1.57	10.84	6.40	0.71	0.0221	1.93	2.32	–0.39
**53.2**	656	Zeonex	9.7	13.78 ± 0.89	1.19 ± 0.05		2.04	2.32	6.55	4.87	2.56	0.0305	1.89	1.89	0.001
	634	CBP	12.4	12.5 ± 0.47	15.719 ± 3.2		2.36	2.96	7.01	5.62	0.21	0.0427	1.96	1.89	0.07
**53.3**	657	Zeonex	19.8	12.44 ± 0.91	36.70 ± 3.9		2.88	4.10	6.45	5.97	0.13	0.0239	1.89	1.84	0.05
	641	CBP	4.9	6.74 ± 0.13	2.76 ± 0.1		1.49	2.97	14.11	8.75	0.88	0.0324	1.93	2.31	–0.38
**53.4**	632	Zeonex	17.6	14.27 ± 0.90	4.85 ± 0.596		3.63	2.66	5.77	5.50	0.96	0.0364	1.96	1.90	0.06
	626	CBP	21.2	10.63 ± 0.69	0.5 ± 0.038		5.22	3.20	7.42	7.90	12.44	0.0415	1.98	1.91	0.07
**54.1**	617	Zeonex	41.5	7.79 ± 0.33				53.27	7.51				2.01		
	617	CBP	12.0	6.17 ± 0.27		18.21 ± 1.29	0.02	19.12	14.26	0.33			2.01	2.05	–0.04
**54.4**	600	Zeonex	25.1	13.12 ± 0.73				19.13	5.71				2.07		
	596	CBP	8.9	9.99 ± 0.50		3.98 ± 0.52	0.10	8.11	9.12	0.94			2.08	2.09	–0.01
**55.1**	598	Zeonex	25.7	16.44 ± 0.60	8.9 ± 0.836		2.06	5.11	4.52	4.10	0.34	0.0735	2.07	1.95	0.12
	613	CBP	31.9	11.31 ± 0.22	3.34 ± 0.316		2.51	8.05	6.02	6.32	1.05	0.0495	2.02	2.42	–0.40
**55.3**	590	Zeonex	25.0	18.05 ± 1.29	3147.88 ± 55.25		0.86	7.42	4.16	2.57	0.001	0.0957	2.10	1.93	0.17
	606	CBP	24.7	11.13 ± 0.57	16.83 ± 1.6		1.65	8.36	6.77	5.60	0.16	0.0325	2.05	2.25	–0.20
**55.4**	587	Zeonex	21.2	14.79 ± 0.35	3.59 ± 0.35		4.18	2.77	5.33	5.46	1.44	0.0994	2.11	1.94	0.17
	604	CBP	39.1	10.43 ± 0.21	14.94 ± 0.73		4.43	6.90	5.84	7.82	0.36	0.0492	2.05	1.89	0.16
**56.2**	543	Zeonex	19.6	19.7 ± 0.47		54.64 ± 14.3	0.02	9.75	4.08	0.11			2.28	1.95	0.33
	552	CBP	23.1	14.51 ± 0.20		1.13 ± 0.08	0.13	14.13	5.30	0.77			2.09	1.91	0.18
**56.3**	590	Zeonex	60.7	10.39 ± 0.18				58.42	3.78				2.10	2.09	0.00
	586	CBP	42.4	8.35 ± 0.53	304.78 ± 83.19		0.03	49.46	6.90	0.31	0.003	0.0014	2.11	2.18	–0.07
**56.4**	577	Zeonex	74.2	12.16 ± 0.223				61.02	2.12				2.15	1.96	0.19
	586	CBP	62.1	7.5766 ± 0.06				81.96	5.00				2.12	2.29	–0.17

a The maximum wavelength of PL spectra.

b PLQY in the host under vacuum.

c PF lifetime.

d DF lifetime.

e RTP lifetime.

f Ratio of delayed emission (DF and RTP) to PF.

g Estimates of *k*_r_ and *k*_nr_ assuming that the emitting state is formed with unit efficiency such that *k*_r_ = Φ/τ and *k*_nr_ = (1 – Φ)/τ.^47^

h Values of RISC rate constant, *k*_RISC_*k*_RISC_ = (DF/PF)/τ_DF_.^47^ Rough estimation of the rate constant values as this system does not fulfill all the assumptions to use the above-mentioned equations.^47^

i Activation energy of triplet to singlet transfer (error ±0.01).

j Singlet and triplet energy (error ±0.03 eV).

k Energy splitting (error ±0.05 eV). All parameters are estimated at 300 K.

### 2.5 Organic Light-Emitting Diode Analysis

**Figure 6:**
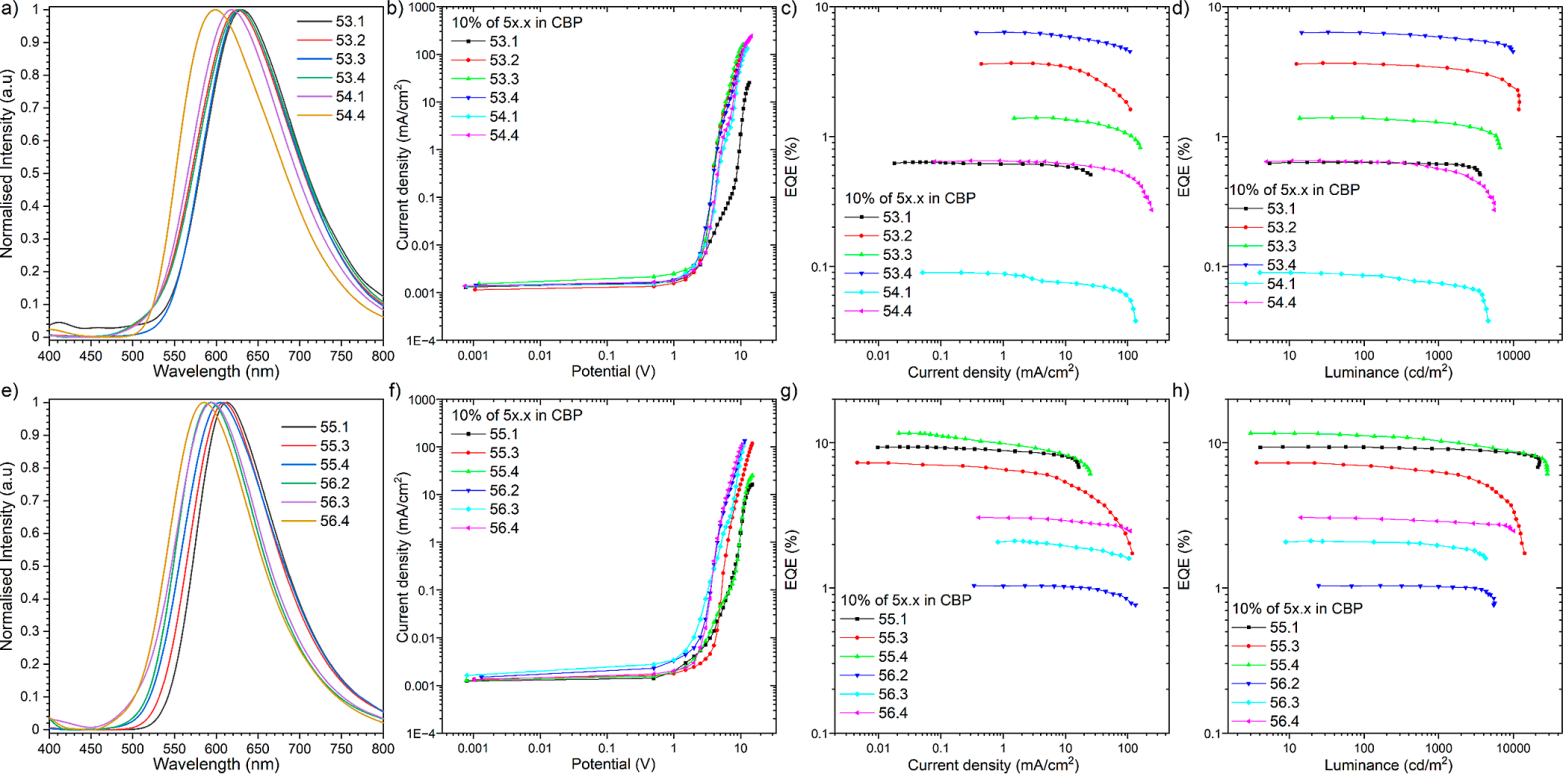
Characteristics of the OLEDs based on emitters. Electroluminescence spectra (a,e). Current density-bias characteristics (b,f). EQE—current density (c,g). EQE luminance characteristics (d,h).

A strong correlation between distinct TADF emission behavior and the substitution mode across the entire pool of regioisomers prompted us to explore them as emitters in an OLED, thus verifying their applicability as optoelectronic devices (Figure 6). Prior to device fabrication, the thermal stability of the entire set of regioisomers was evaluated by means of thermogravimetric analysis (TGA)/differential scanning calorimetry (DSC) techniques. In this context, we found most of our dyes to be stable around 300 °C (for detailed results, see Supporting Information, Figures S10–S21). To choose the appropriate OLED structure, the HOMO and LUMO energy levels of the compounds were determined by cyclic voltammetry (CV) (Figure S3, Supporting Information). The CV indicated very good electrochemical stability at both p and n doping. The devices were fabricated using a thermal evaporation technique.

For all compounds, the optimal configuration of the device was obtained as follows: ITO/1,4,5,8,9,11-hexaazatriphenylenehexacarbonitrile (HAT-CN) (10 nm)/*N*,*N*′(di(1-naphthyl)-*N*,*N′*-diphenyl-(1,1′-biphenyl)-4,4′-diamine (NPB) (30 nm)/10% of **5X.X** in CBP (30 nm)/1,3,5-tri(*m*-pyridin-3-ylphenyl)benzene (TmPyPB) (40 nm)/lithium fluoride (LiF) (1 nm)/Al (100 nm)] (Figure 5). The characteristics of the OLED structures revealed a good efficiency of **55.X** TADF emitters in a CBP host containing an OLED between 8 and 11% (Figure 6g,h), which exceeds the theoretical maximum of the OLED based on only the fluorescence process (ca. 5%). The devices based on isomers **53.X** also exhibit electroluminescence based on a TADF process; nevertheless, the low photoluminescence quantum yield (PLQY) of the sole material itself explained the lower device EQE (Figure 6c,d). OLEDs based on isomers **54.X** and **56.X** had emission mostly from the classical fluorescence process. The highest luminance of the device at around 28,000 cd/m^2^ was observed for compound **55.4**, which also had the highest EQE (11.6%). From the “inverted” emitters, the highest luminance and efficiency were obtained for isomer **55.1** with values of 22,000 cd/m^2^ and 9.3%, respectively. This suggests a rather low impact of the TICT mechanism and conformation on the final efficiency but rather the overall emissivity of the compounds (PLQY). There was no significant impact of the isomers on the overall stability and roll-off of the devices; in this configuration, all of the devices had good stability, which suggests the appropriate device configuration.

## 3 Conclusions

In summary, a unique approach involving the use of higher-order NBI-based regioisomers was employed to correlate the impact of the host matrix on the conformational change of emitters, leading to distinctive TADF emission mechanisms. A better DF efficiency (DF/PF ratio) was observed in isomers where the donor is attached close to the carbonyl moiety of the acceptor (4,10 isomers), i.e., PXZ and tBuCz, which exhibited superior TADF photophysics compared to those of other isomers. This phenomenon lets us clearly illustrate that the photophysics of the compounds are influenced not only by regioisomerism but also by the nature of the host material. Moreover, the primary impact is observed in the T_1_ state rather than in the S_1_ state, as transparently seen from the emission profiles Analogously, S–T inversion was detected in the IDB-linked dyes (3,11; 4,10) and tBuCbz-terminated species (3,10; 4,10) sets, although the energy inversion is not as pronounced (<−0.20), unlike the previous dye sets. The best-performing regioisomers (4,10) in the series, possessing electron-donating DMAc groups, show a very high efficiency of 11.6% with a pronounced luminance of 28,000 cd/m^2^. The performed studies delineate a new path for a better fundamental understanding of the interaction of TADF emitters vs host matrixes and its consequences, but it also can lead to the revival of the NBI scaffold, which postmodification can permit access to highly efficient OLED emitters, which is now under investigation in our group.

## 4 Experimental Section

### 4.1 Synthesis of Regioisomers

The synthetic procedures and spectroscopic identification of the obtained regioisomers **53.1-4**; **54.1**,**4**; **55.1**,**3-4**; and **56.2-4** are given in Sections SI–8 of the Supporting Information.

### 4.2 General Remarks

All of the reagents and solvents utilized in the experiments were obtained commercially and used without further purification. Reaction-grade solvents such as CH_2_Cl_2_, ethyl acetate, and hexane were distilled before application. For reactions sensitive to water, solvents underwent drying using the Swift solvent purification system by MBraun. Meanwhile, reactions susceptible to moisture and oxygen were conducted under an inert argon atmosphere. The progression of each reaction was monitored using thin-layer chromatography (TLC) on silica gel-coated (60 F_254_ Merck) aluminum foil plates. Purification of intermediates and final products was accomplished through column chromatography using a Merck Kieselgel 60 Merck. Characterization of all intermediates and target compounds was carried out using ^1^H NMR, ^13^C, and 2D NMR spectroscopies and HRMS spectrometry (via EI-MS). NMR spectra were recorded using Bruker AM 500 MHz, Bruker AM 600 MHz, Varian 600 MHz, or Varian 400 MHz instruments, with tetramethylsilane (TMS) employed as the internal standard. Chemical shifts for ^1^H NMR are reported in parts per million (ppm) relative to TMS (δ 0.00 ppm) and CDCl_3_ (δ 7.26 ppm). Chemical shifts for ^13^C NMR are expressed in ppm relative to CDCl_3_ (δ 77.16 ppm). Data are presented in the following format: chemical shift, multiplicity (s = singlet, d = doublet, dd = doublet of doublets, t = triplet, td = triplet of doublets, q = quartet, p = quintet, hept = septet, and m = multiplet), coupling constant (Hz), and integration. EI mass spectra were recorded using an AutoSpec Premier spectrometer, while IR spectra were obtained using a JASCO FT/IR-6200 spectrometer. TGA experiments were conducted using a Mettler-Toledo TGA/DSC 3+ thermal gravimetric analyzer under a nitrogen atmosphere with a temperature range of 50 to 500 °C and a heating rate of 5 °C/min. The temperatures corresponding to 5 and 10 wt % of mass loss were determined. Additionally, DSC experiments (heating/cooling at 5 °C/min) were performed using a Mettler-Toledo DSC 3 analyzer.

### 4.3 Photophysics

Photophysical measurements were conducted in a similar fashion as the preceding protocol.^34^ The UV–vis spectra were obtained by using a Shimadzu UV-2550 spectrophotometer, while steady-state emission spectra were measured with a Jobin Yvon Horiba Fluoromax 3 spectrofluorometer. PL spectra were calibrated for the detector efficiency based on specific calibration files provided by the instrument manufacturer. PL measurements in solution were conducted using PL cuvettes (Aireka Cells; path length: 1 cm). Toluene solutions of the analyzed compounds were degassed through 5 freeze/thaw/pump cycles using a custom-made degassing cell equipped with a young tap (path length: 1 cm). Temperature-dependent experiments were performed within a Janis Research cryostat cooled with liquid nitrogen. PLQYs were determined using an integrating sphere both in solution and in the solid state. Matrix-doped films were prepared on cleaned and dried sapphire disc substrates, with 1 wt % of the emitter in the Zeonex host. PF, phosphorescence, and DF spectra and decays were measured using nanosecond-gated luminescence and lifetime investigations (ranging from 400 ps to 1 s). A Q-Spark A50-TH-RE high-energy pulsed DPSS laser (λ_em_ = 355 nm) and a Stanford Computer Optics sensitive gated iCCD camera with subnanosecond resolution were utilized for these experiments. Time-resolved analysis of PF/DF was conducted by progressively increasing gate and integration times exponentially. Temperature-dependent experiments under vacuum were carried out within a Janis Research cryostat cooled with helium. Time-resolved spectra were recorded using a Stanford Computer Optics 4Picos iCCD camera, with gate and delay times exponentially increased to avoid overlap. Each emission spectrum collected for the respective emitter was integrated to generate an accurate luminescence decay profile.

#### 4.3.1 Devices

The OLEDs were fabricated following procedures similar to those described previously.^26,34^ HAT-CN was used as a hole injection layer, NPB was used as a hole transport layer, and TmPyPB was introduced as an electron transport layer. LiF and aluminum were used as the cathodes. Organic semiconductors and aluminum were deposited at a rate of 1 Å s^–1^, and the LiF layer was deposited at 0.1 Å s^–1^. CBP was used as the host for all emitters. All materials were purchased from Sigma-Aldrich or Lumtec and purified by temperature-gradient sublimation in vacuum. OLEDs have been fabricated on precleaned, patterned indium–tin-oxide (ITO)-coated glass substrates with a sheet resistance of 20 Ω/sq and an ITO thickness of 100 nm. All small molecules and cathode layers were thermally evaporated in a Kurt J. Lesker Spectros evaporation system under a pressure of 10^–7^ mbar without breaking the vacuum. The sizes of pixels were 4, 8, and 16 mm^2^. Each emitting layer has been formed by the codeposition of the dopant and host at the specific rate to obtain the 10% content of the emitter. The characteristics of the devices were recorded using a 6 in. integrating sphere (Labsphere) inside the glovebox connected to a source meter unit and an Ocean Optics USB4000 spectrometer.

### 4.4 Calculations

DFT calculations were conducted by using the QChem 5.0 software package. The ωB97X-D functional was employed for geometry optimization, while ωPBE was appropriately adjusted for calculations of excited-state levels. Nonequilibrium PCM models were employed to account for solvation effects. Additional information regarding the calculations can be found in the Supporting Information (SI).

## Supporting Information Available

^1^H, ^13^C, and 2D NMR spectroscopies along with high-resolution mass spectrometry; detailed photophysical methods and spectra; and computational data and coordinates (PDF)

## Notes

The data presented in this manuscript also constitute the subject of the patent call, which was previously submitted to the Polish Patent office (P.444060).
