# Correction to “Regioisomerism vs Conformation: Impact of Molecular Design on the Emission Pathway in Organic Light-Emitting Device Emitters”

**DOI:** 10.1021/acsami.4c13359

**Published:** 2024-09-13

**Authors:** Prasannamani Govindharaj, Aleksandra J. Wierzba, Karolina Kęska, Michał Andrzej Kochman, Gabriela Wiosna-Sałyga, Adam Kubas, Przemysław Data, Marcin Lindner

The original article contains a minor error in the description of the electronic structure calculations reported in our article, which does not affect our study’s findings and conclusions.

Section 4.4 (Calculations) on page 23665 should be revised as follows:

The ground-state geometries of the compounds under study were optimized at the density functional theory (DFT) level. Afterward, their electronic excitation spectra were calculated with the use of the linear response time-dependent DFT (TDDFT) method. TDDFT was also employed in excited-state geometry optimizations. In either case, the ωB97XD exchange-correlation functional^48^ was used in combination with the def2-SVP basis set.^49^ A more detailed description of the simulation methodology is provided in the Supporting Information, in Section SI-1: Computational Methods.

The findings and conclusions of our paper remain unchanged, as the error was limited to the abridged summary of the computational methodology in Section 4.4 (Calculations) on page 23665. The calculations themselves, and their results, were not affected.

Two new refs (refs 48 and 49) must be added to the original paper:

(48) Chai, J.-D.; Head-Gordon, M. Long-Range Corrected Hybrid Density Functionals with Damped Atom-Atom Dispersion Corrections. *Phys. Chem. Chem. Phys.***2008**, *10*, 6615–6620. DOI: 10.1039/B810189B.

(49) Weigend, F.; Ahlrichs, R. Balanced Basis Sets of Split Valence, Triple Zeta Valence and Quadruple Zeta Valence Quality for H to Rn: Design and Assessment of Accuracy. *Phys. Chem. Chem. Phys.***2005**, *7*, 3297–3305. DOI: 10.1039/b508541a.

Moreover, on page 23665, the funding information in the Acknowledgments section should be changed as follows: P.D. acknowledges the Polish National Science Centre funding, grant no. 2022/45/B/ST5/03712. P.G. acknowledges that the publication was produced as part of the Doctoral Candidate’s participation in a project of the National Agency for Academic Exchange within the framework of the “STER” Programme - Internationalisation of Doctoral Schools” as part of the project “Curriculum for advanced doctoral education & training - CADET Academy of Lodz University of Technology”.

