# Supplementary material for: Regioisomerism vs Conformation: Impact of Molecular Design on the Emission Pathway in Organic Light-Emitting Device Emitters
Source: ACS Appl Mater Interfaces. 2024 Apr 26;16(18):23654–67. doi: 10.1021/acsami.3c19212 (PMC11082840; doi:10.1021/acsami.3c19212)
Supplement: Supplementary file 1 — am3c19212_si_001.pdf [file am3c19212_si_001.pdf]

Supporting Information

Regioisomerism vs conformation: impact of molecular design on the emission pathway in OLED emitters

‡Prasannamani Govindharaj,[c] ‡Aleksandra J. Wierzba,[a] Karolina Kęska,[a] ‡Michał Andrzej Kochman,[b] Gabriela Wiosna-Sałyga,[c] Adam Kubas,*[b] Przemysław Data,*[c] Marcin Lindner,*[a]

[a]Institute of Organic Chemistry, Polish Academy of Sciences, Kasprzaka 44/52, 01-224 Warsaw, Poland;

[b]Institute of Physical Chemistry, Polish Academy of Sciences, Kasprzaka 44/52, 01-224 Warsaw, Poland;

[c]Łódź University of Technology, Department of Molecular Physics, Stefana Żeromskiego 114, Łódź 90-543, Poland

‡ these authors contributed equally

Corresponding Authors:*

Marcin Lindner - https://orcid.org/0000-0002-5514-674X; marcin.lindner@icho.edu.pl

Przemysław Data - https://orcid.org/0000-0002-1831-971X; przemyslaw.data@p.lodz.pl

Adam Kubas - https://orcid.org/ 0000-0002-5508-0533; akubas@ichf.edu.pl

SI-1 Computational Methods 3
SI-2 Fluorescence Emission Spectra 18
SI-3 Cyclic Voltammetry 19
SI-4 Additional steady state photophysics 21
SI-5 Time resolved photophysics 23
SI-6 OLED devices 26
SI-7 TGA/DSC and DSC (heating/cooling) measurements 27
SI-8. Synthesis - general information 33
SI-8.1 Synthetic protocols 34
SI-8.2 Copies of NMR spectra and 2D NMR signal assignments 39
SI-8.3 Structural analysis based on 2D NMR spectra for compound 53.1 106

SI-1 Computational Methods

The calculations were performed for the **53.X** and the **56.X** as the reference series of compounds. In the case of the **56.X** series of compounds, the *tert*-butyl groups on the carbazole moiety were deleted and replaced by hydrogens. For the sake of completeness, in our calculations we did include compound **56.1**, even though its synthesis was unsuccessful.

The geometries of the compounds under study were optimized at the density functional theory (DFT) level. Afterwards, their electronic excitation spectra were calculated with the use of the linear response time-dependent DFT (TDDFT) method. The TDDFT method was also used to optimize the excited-state equilibrium geometry of each compound, and to calculate its fluorescence emission energy.

The DFT and TDDFT calculations were performed with the computational chemistry software package Gaussian 09, Revision D.01.[4] We elected to employ the ωB97XD[1] exchange-correlation functional, as this functional performs reasonably well in the calculation of intramolecular charge transfer (ICT) states.[2,3] The def2-SVP basis set$_5$ was used at all times, and the so-called 'Superfine' integration grid was applied. As a measure to reduce the computational cost of the TDDFT calculations, we imposed the Tamm-Dancoff approximation (TDA). All DFT-optimized ground-state geometries were confirmed to correspond to energy minima through analytical calculations of vibrational frequencies. In the case of the TDDFT-optimized excited-state geometries, however, vibrational frequency calculations would have been prohibitively expensive in terms of computing time and were not performed.

Table S1: Vertical excitation spectra of the **53.X** series of compounds as calculated at the TDA-ωB97XD/def2-SVP level of theory – vertical excitation energies (Δ*E*) and associated oscillator strengths (*f*).

Compound	State	Δ*E*, eV	*f*
**53.1** (conformer D1-*eq*,D2-*eq*)	S_1 (D2→A ICT)	2.748	$< 1 \times 10^{-4}$
	S_2 (D1→A ICT)	3.327	$< 1 \times 10^{-4}$
	S_3 (A $^1\pi\pi^*$)	3.773	0.757
	S_4 (D1 $^1\pi\pi^*$)	3.996	0.004
	T_1 (A $^3\pi\pi^*$)	2.505	0
	T_2 (D2→A ICT)	2.736	0
	T_3 (D1 $^3\pi\pi^*$)	3.182	0
	T_4 (D2 $^3\pi\pi^*$)	3.252	0
**53.2** (conformer D1-eq,D2-eq)	S_1 (D2→A ICT)	2.748	$< 1 \times 10^{-4}$
	S_2 (D1→A ICT)	3.466	2×10^{-4}
	S_3 (A $^1\pi\pi^*$)	3.776	0.732
	S_4 (D1 $^1\pi\pi^*$)	4.023	0.008
	T_1 (A $^3\pi\pi^*$)	2.504	0
	T_2 (D2→A ICT)	2.736	0
	T_3 (D1 $^3\pi\pi^*$)	3.174	0
	T_4 (D2 $^3\pi\pi^*$)	3.254	0
**53.3** (conformer D1-*eq*,D2-*eq*)	S_1 (D2→A ICT)	2.782	$< 1 \times 10^{-4}$
	S_2 (D1→A ICT)	3.319	$< 1 \times 10^{-4}$
	S_3 (A $^1\pi\pi^*$)	3.815	0.891
	S_4 (D1 $^1\pi\pi^*$)	4.000	0.005
	T_1 (A $^3\pi\pi^*$)	2.510	0
	T_2 (D2→A ICT)	2.770	0
	T_3 (D1 $^3\pi\pi^*$)	3.181	0
	T_4 (D2 $^3\pi\pi^*$)	3.247	0
**53.4** (conformerD1-eq,D2-eq)	S_1 (D2→A ICT)	2.779	$< 1 \times 10^{-4}$
	S_2 (D1→A ICT)	3.454	2×10^{-4}
	S_3 (A $^1\pi\pi^*$)	3.822	0.862
	S_4 (D1 $^1\pi\pi^*$)	4.033	0.009
	T_1 (A $^3\pi\pi^*$)	2.511	0
	T_2 (D2→A ICT)	2.768	0
	T_3 (D1 $^3\pi\pi^*$)	3.173	0
	T_4 (D2 $^3\pi\pi^*$)	3.246	0

Table S2: Vertical excitation spectra of the **56.X** series of compounds as calculated at the TDA-ωB97XD/def2-SVP level of theory – vertical excitation energies (Δ*E*) and associated oscillator strengths (*f*).

Compound	State	Δ*E*, eV	*f*
**56.1** (conformer **I**)	S_1 (D2→A ICT)	3.498	0.597
	S_2 (A $^1\pi\pi^*$)	3.646	0.242
	S_3 (D1→A ICT)	4.268	0.039
	S_4 (D2→A ICT)	4.281	0.003
	T_1 (A $^3\pi\pi^*$)	2.455	0
	T_2 (A $^3\pi\pi^*$)	3.194	0
	T_3 (D2→A ICT)	3.605	0
	T_4 (D1 $^3\pi\pi^*$)	3.618	0
**56.2** (conformer **I**)	S_1 (D2→A ICT)	3.521	0.328
	S_2 (A $^1\pi\pi^*$)	3.759	0.356
	S_3 (D1→A ICT)	4.067	0.235
	S_4 (D2→A ICT)	4.281	0.003
	T_1 (A $^3\pi\pi^*$)	2.477	0
	T_2 (A $^3\pi\pi^*$)	3.249	0
	T_3 (D1 $^3\pi\pi^*$)	3.619	0
	T_4 (D2 $^3\pi\pi^*$)	3.621	0
**56.3** (conformer **I**)	S_1 (D2→A ICT)	3.429	0.600
	S_2 (A $^1\pi\pi^*$)	3.766	0.256
	S_3 (D2→A ICT)	4.276	0.025
	S_4 (A $^1\pi\pi^*$)	4.313	0.057
	T_1 (A $^3\pi\pi^*$)	2.447	0
	T_2 (A $^3\pi\pi^*$)	3.320	0
	T_3 (D2→A ICT)	3.526	0
	T_4 (D1 $^3\pi\pi^*$)	3.617	0
**56.4** (conformer **I**)	S_1 (D2→A ICT)	3.466	0.390
	S_2 (A $^1\pi\pi^*$)	3.881	0.185
	S_3 (D1→A ICT)	4.060	0.410
	S_4 (D2→A ICT)	4.285	0.006
	T_1 (A $^3\pi\pi^*$)	2.472	0
	T_2 (A $^3\pi\pi^*$)	3.413	0
	T_3 (D2→A ICT)	3.545	0
	T_4 (D1→$^3\pi\pi^*$)	3.619	0

As noted in the main body of the manuscript, the electronic structures of the excited electronic states of compounds **53.1–53.4** and **56.1–56.4** were analyzed by plotting their electron density difference maps (EDDMs). The EDDM of a given excited state is defined as the difference between its electron density, and the electron density of the ground state, at the same molecular geometry.

In the present section, we show plots the EDDMs for the excited states of compounds in the series **53.1–53.4** (in Figure **S1** below) and in the series **56.1–56.4** (Figure **S2**). The diabatic character of the given excited state is identified under the relevant figure.

Figure S1: EDDMs for the low-lying excited electronic states of compounds in the series **53.1** to **53.4**. The EDDMs are plotted in the form isosurfaces with isovalues of ±0.0025 e/a_0^3 . The red and blue isosurfaces delimit regions in which the electron density is increased and decreased, respectively, relative to the ground state (S_0).

(a) compound **53.1**, conformer D1-*eq*, D2-*eq*

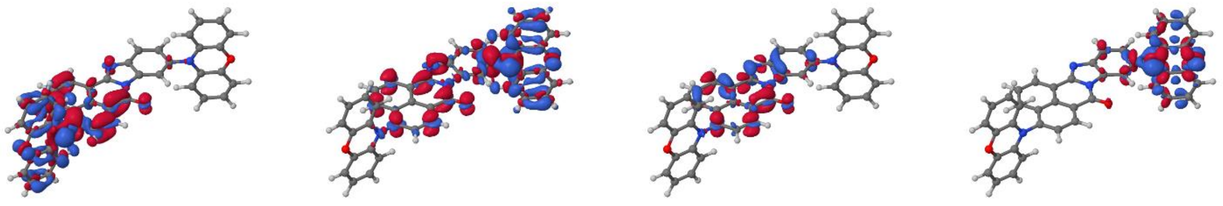

(i) S_1 (D2→A ICT) (ii) S_2 (D1→A ICT) (iii) S_3 (A $^1\pi\pi^*$) (iv) S_4 (D1 $^1\pi\pi^*$)

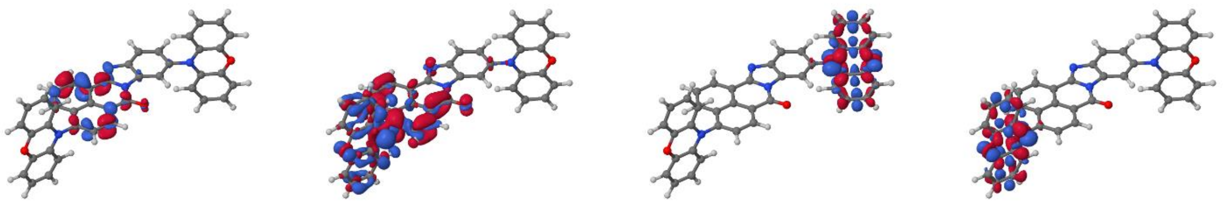

(v) T_1 (A $^3\pi\pi^*$) (vi) T_2 (D2→A ICT) (vii) T_3 (D1 $^3\pi\pi^*$) (viii) T_4 (D2 $^3\pi\pi^*$)

(b) compound **53.1**, conformer D1-*ax*, D2-*eq*

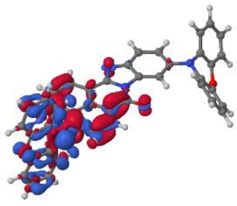

(i) S_1 (D2→A ICT)

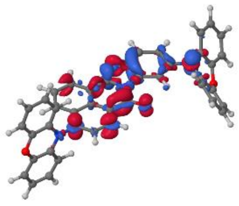

(ii) S_2 (A $^{1}\pi\pi^*$)

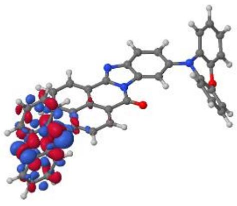

(iii) S_3 (D2 $^{1}\pi\pi^*$)

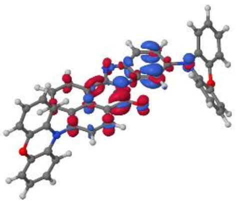

(iv) S_4 (A $^{1}\pi\pi^*$)

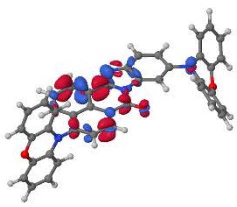

(v) T_1 (A $^{3}\pi\pi^*$)

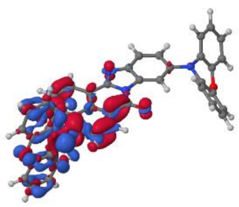

(vi) T_2 (D2→A ICT)

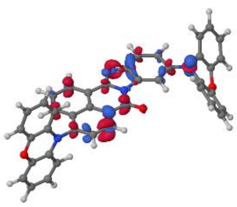

(vii) T_3 (A $^{3}\pi\pi^*$)

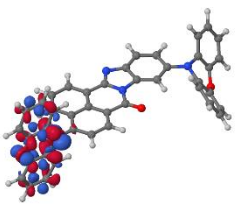

(viii) T_4 (D2 $^{3}\pi\pi^*$)

(c) compound **53.2**, conformer D1-*eq*, D2-*eq*

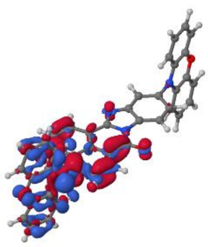

(i) S_1 (D2→A ICT)

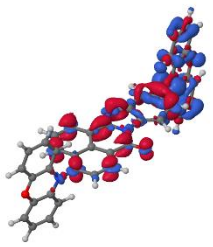

(ii) S_2 (D1→A ICT)

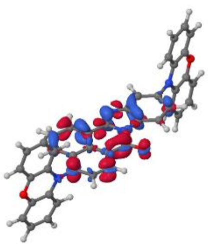

(iii) S_3 (A $^{1}\pi\pi^*$)

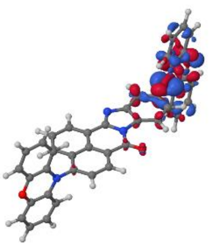

(iv) S_4 (D1 $^{1}\pi\pi^*$)

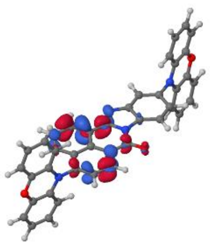

(v) T_1 (A $^3\pi\pi^*$)

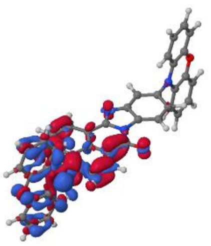

(vi) T_2 (D2→A ICT)

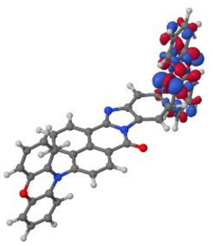

(vii) T_3 (D1 $^3\pi\pi^*$)

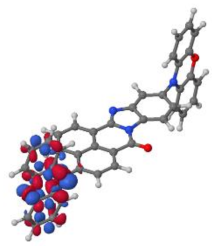

(viii) T_4 (D2 $^3\pi\pi^*$)

(d) compound **53.2**, conformer D1-*ax*, D2-*eq*

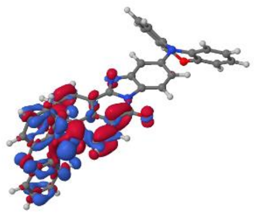

(i) S_1 (D2→A ICT)

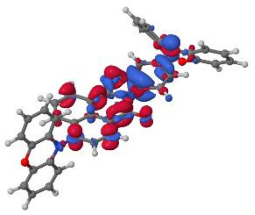

(ii) S_2 (A $^1\pi\pi^*$)

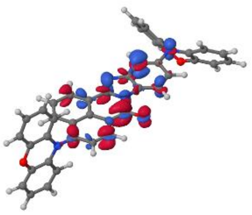

(iii) S_3 (A $^1\pi\pi^*$)

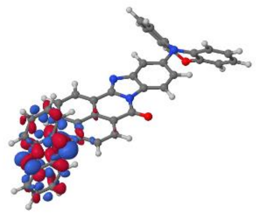

(iv) S_4 (D2 $^1\pi\pi^*$)

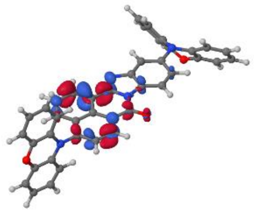

(v) T_1 (A $^3\pi\pi^*$)

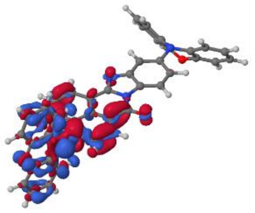

(vi) T_2 (D2→A ICT)

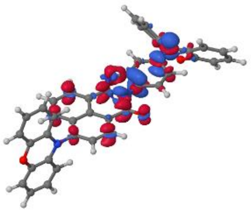

(vii) T_3 (D1→A ICT)

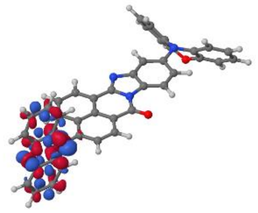

(viii) T_4 (D2 $^3\pi\pi^*$)

(e) compound **53.3**, conformer D1-*eq*, D2-*eq*

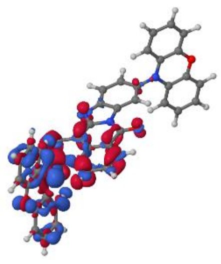

(i) S_1 (D2→A ICT)

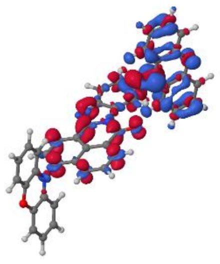

(ii) S_2 (D1→A ICT)

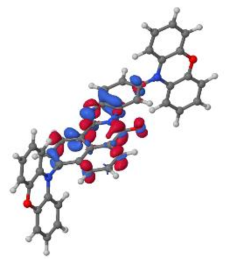

(iii) S_3 (A $^1\pi\pi^*$)

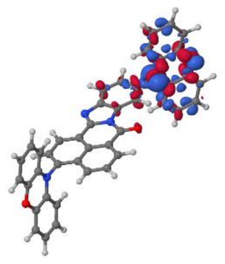

(iv) S_4 (D1 $^1\pi\pi^*$)

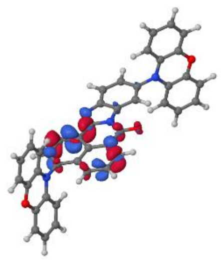

(v) T_1 (A $^3\pi\pi^*$)

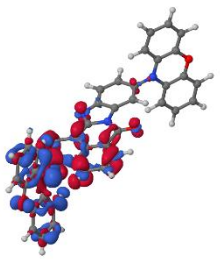

(vi) T_2 (D2→A ICT)

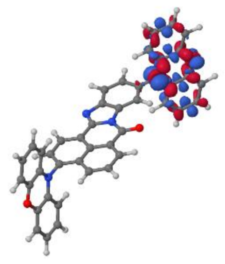

(vii) T_3 (D1 $^3\pi\pi^*$)

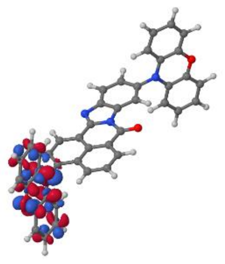

(viii) T_4 (D2 $^3\pi\pi^*$)

(f) compound **53.3**, conformer D1-*ax*, D2-*eq*

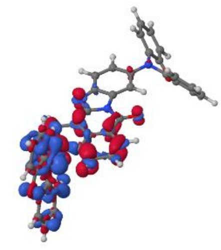

(i) S_1 (D2→A ICT)

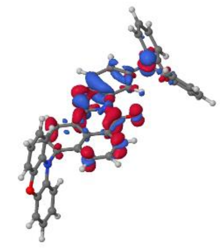

(ii) S_2 (A $^1\pi\pi^*$)

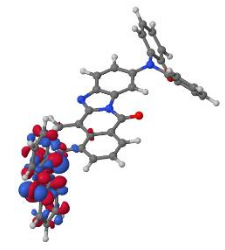

(iii) S_3 (D2 $^1\pi\pi^*$)

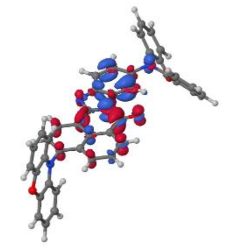

(iv) S_4 (A $^1\pi\pi^*$)

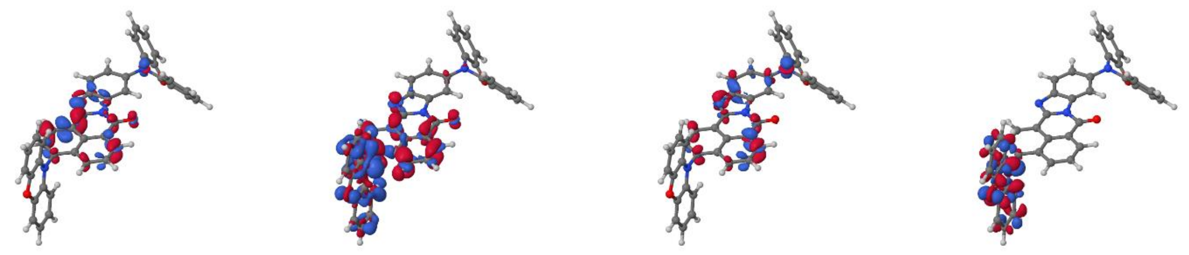

(v) T_1 (A $^3\pi\pi^*$) (vi) T_2 (D2→A ICT) (vii) T_3 (A $^3\pi\pi^*$) (viii) T_4 (D2 $^3\pi\pi^*$)

(g) compound **53.4**, conformer D1-*eq*, D2-*eq*

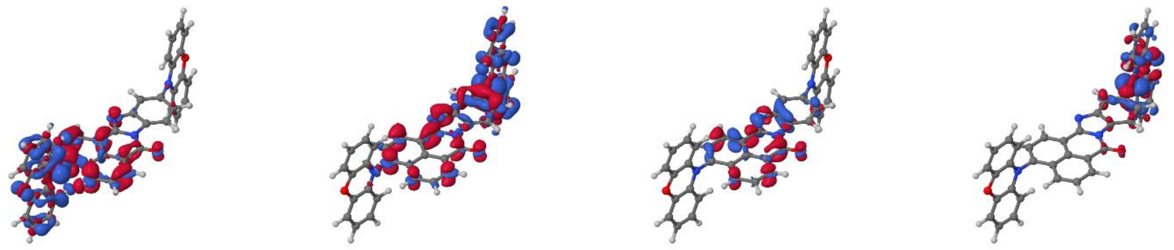

(i) S_1 (D2→A ICT) (ii) S_2 (D1→A ICT) (iii) S_3 (A $^1\pi\pi^*$) (iv) S_4 (D1 $^1\pi\pi^*$)

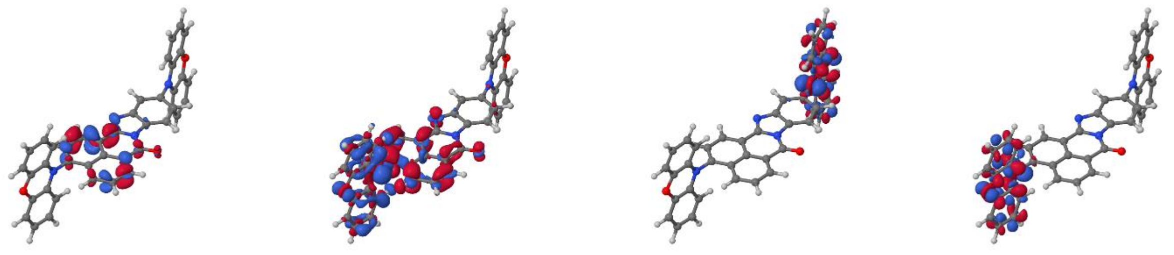

(v) T_1 (A $^3\pi\pi^*$) (vi) T_2 (D2→A ICT) (vii) T_3 (D1 $^3\pi\pi^*$) (viii) T_4 (D2 $^3\pi\pi^*$)

(h) compound **53.4**, conformer D1-*ax*, D2-*eq*

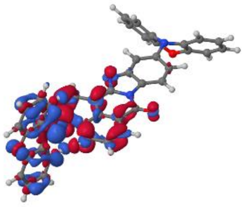

(i) S_1 (D2→A ICT)

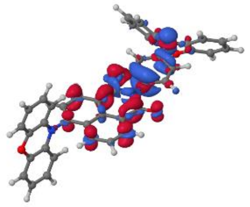

(ii) S_2 (A $^1\pi\pi^*$)

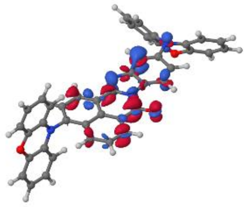

(iii) S_3 (A $^1\pi\pi^*$)

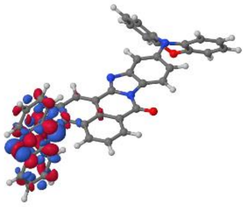

(iv) S_4 (D2 $^1\pi\pi^*$)

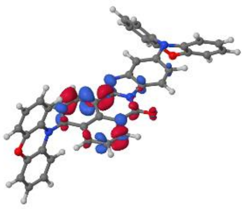

(v) T_1 (A $^3\pi\pi^*$)

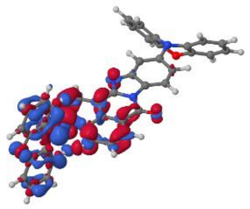

(vi) T_2 (D2→A ICT)

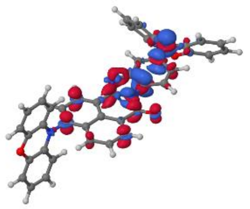

(vii) T_3 (D1→A ICT)

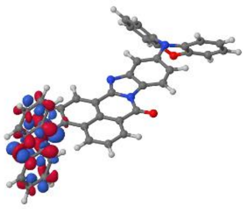

(viii) T_4 (D2 $^3\pi\pi^*$)

Figure S2: EDDMs for the low-lying excited electronic states of compounds in the series **56.1** to **56.4**. The EDDMs are plotted in the form isosurfaces with isovalues of ±0.0025 e/a_0^3 . The red and blue isosurfaces delimit regions in which the electron density is increased and decreased, respectively, relative to the ground state (S_0).

(a) compound **56.1**, conformer **I** (D1 and D2 are roughly coplanar with one another)

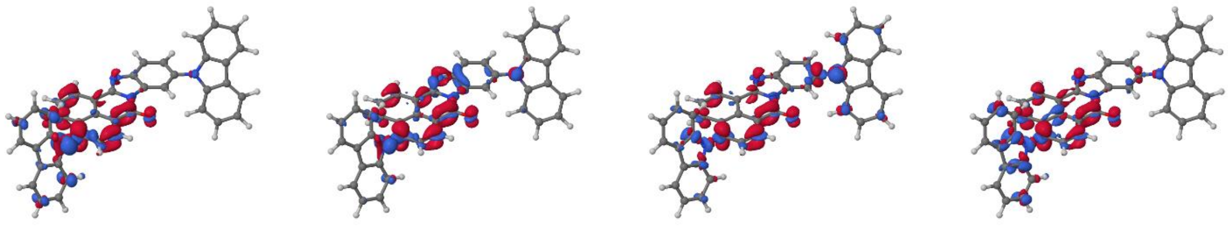

(i) S_1 (D2→A ICT) (ii) S_2 (A $^1\pi\pi^*$) (iii) S_3 (D1→A ICT) (iv) S_4 (D2→A ICT)

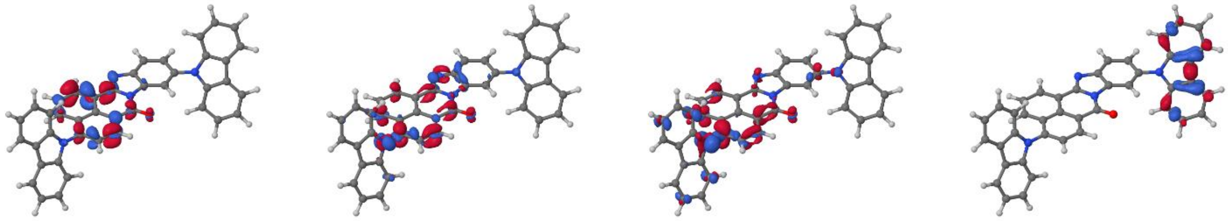

(v) T_1 (A $^3\pi\pi^*$) (vi) T_2 (A $^3\pi\pi^*$) (vii) T_3 (D2→A ICT) (viii) T_4 (D1 $^3\pi\pi^*$)

(b) compound **56.1**, conformer **II** (D1 and D2 are roughly perpendicular to one another)

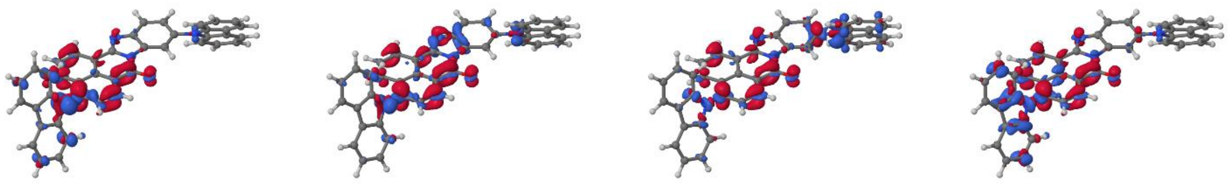

(i) S_1 (D2→A ICT) (ii) S_2 (A $^1\pi\pi^*$) (iii) S_3 (D1→A ICT) (iv) S_4 (D2→A ICT)

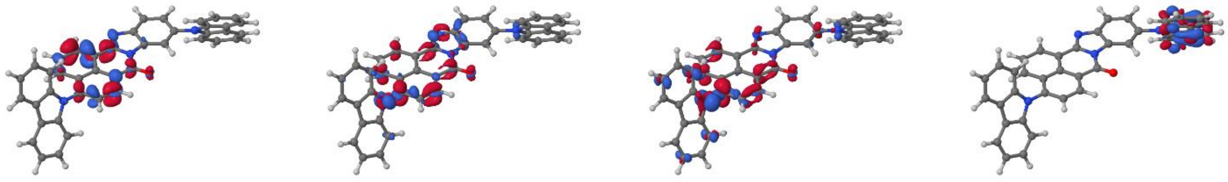

(v) T_1 (A $^3\pi\pi^*$) (vi) T_2 (A $^3\pi\pi^*$) (vii) T_3 (D2→A ICT) (viii) T_4 (D1 $^3\pi\pi^*$)

(c) compound **56.2**, conformer **I** (D1 and D2 are roughly coplanar with one another)

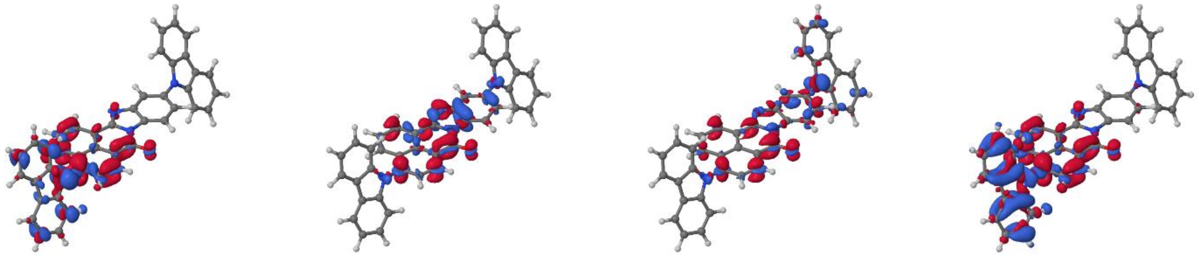

(i) S_1 (D2→A ICT) (ii) S_2 (A $^1\pi\pi^*$) (iii) S_3 (D1→A ICT) (iv) S_4 (D2→A ICT)

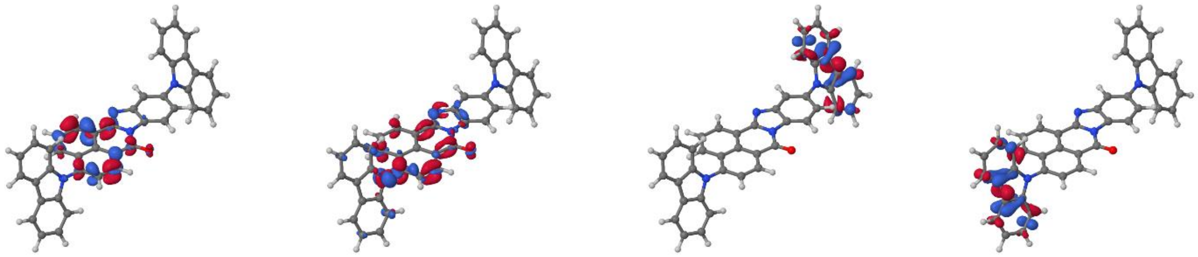

(v) T_1 (A $^3\pi\pi^*$) (vi) T_2 (A $^3\pi\pi^*$) (vii) T_3 (D1 $^3\pi\pi^*$) (viii) T_4 (D2 $^3\pi\pi^*$)

(d) compound **56.2**, conformer **II** (D1 and D2 are roughly perpendicular to one another)

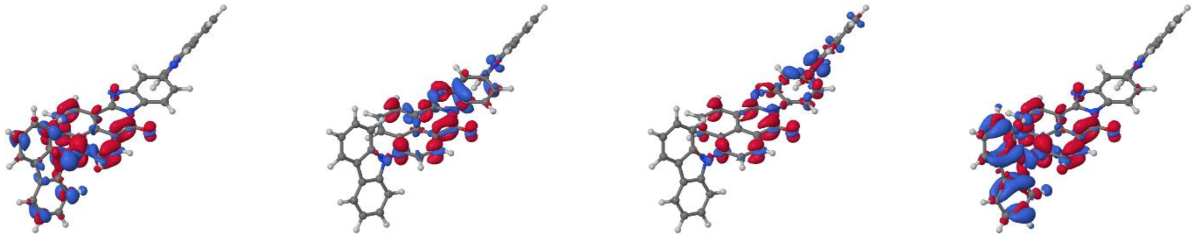

(i) S_1 (D2→A ICT) (ii) S_2 (A $^1\pi\pi^*$) (iii) S_3 (D1→A ICT) (iv) S_4 (D2→A ICT)

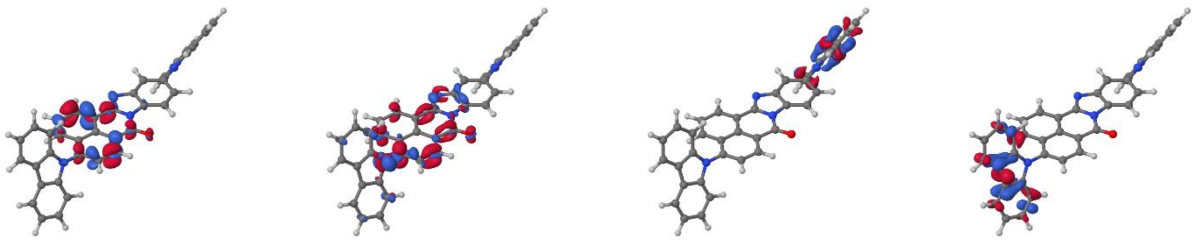

(v) T_1 (A $^3\pi\pi^*$) (vi) T_2 (A $^3\pi\pi^*$) (vii) T_3 (D1 $^3\pi\pi^*$) (viii) T_4 (D2 $^3\pi\pi^*$)

(e) compound **56.3**, conformer **I** (D1 and D2 are roughly coplanar with one another)

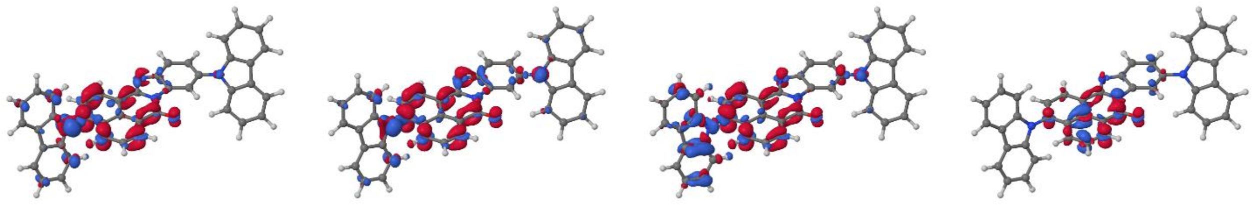

(i) S_1 (D2→A ICT) (ii) S_2 (A $^1\pi\pi^*$) (iii) S_3 (D2→A ICT) (iv) S_4 (A $^1\pi\pi^*$)

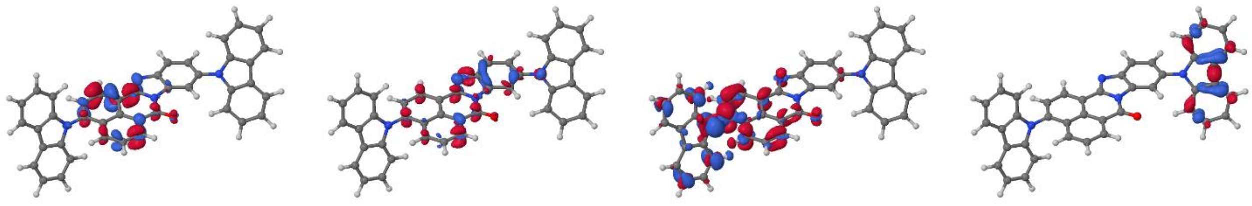

(v) T_1 (A $^{3}\pi\pi^{*}$) (vi) T_2 (A $^{3}\pi\pi^{*}$) (vii) T_3 (D2→A ICT) (viii) T_4 (D1 $^{3}\pi\pi^{*}$)

(f) compound **56.3**, conformer **II** (D1 and D2 are roughly perpendicular to one another)

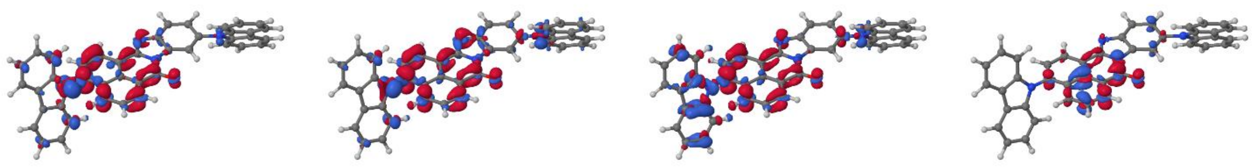

(i) S_1 (D2→A ICT) (ii) S_2 (A $^{1}\pi\pi^{*}$) (iii) S_3 (D2→A ICT) (iv) S_4 (A $^{1}\pi\pi^{*}$)

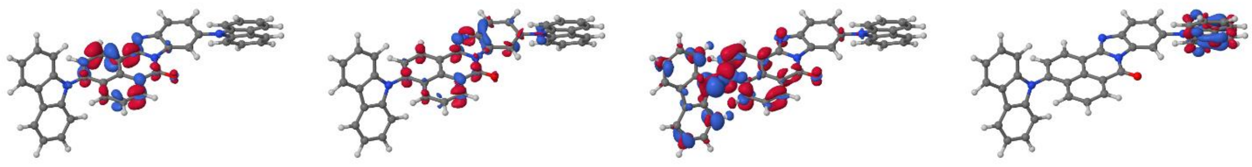

(v) T_1 (A $^{3}\pi\pi^{*}$) (vi) T_2 (A $^{3}\pi\pi^{*}$) (vii) T_3 (D2→A ICT) (viii) T_4 (D1 $^{3}\pi\pi^{*}$)

(g) compound **56.4**, conformer **I** (D1 and D2 are roughly coplanar with one another)

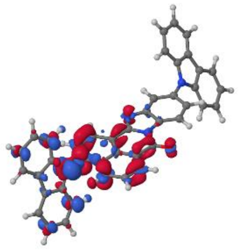
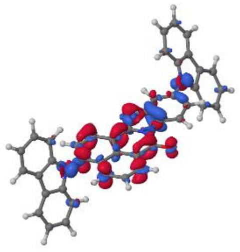
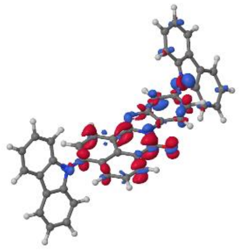
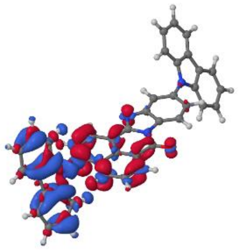

(i) S_1 (D2→A ICT) (ii) S_2 (A $^1\pi\pi^*$) (iii) S_3 (D1→A ICT) (iv) S_4 (D2→A ICT)

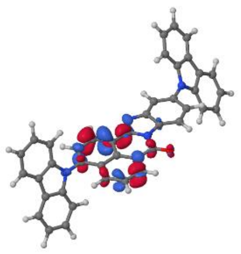
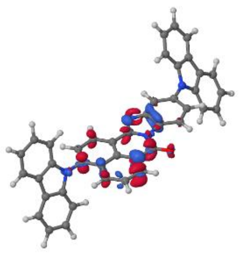
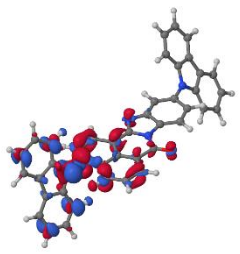
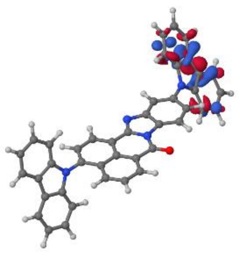

(v) T_1 (A $^3\pi\pi^*$) (vi) T_2 (A $^3\pi\pi^*$) (vii) T_3 (D2→A ICT) (viii) T_4 (D1 $^3\pi\pi^*$)

(h) compound **56.4**, conformer **II** (D1 and D2 are roughly perpendicular to one another)

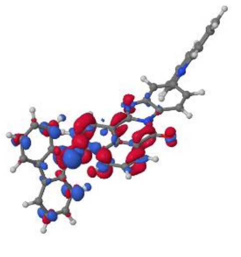
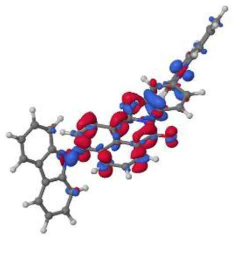
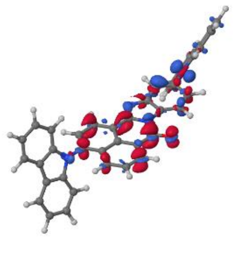
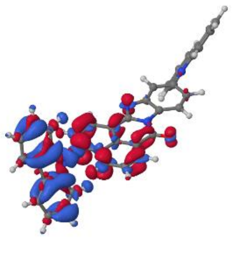

(i) S_1 (D2→A ICT) (ii) S_2 (A $^1\pi\pi^*$) (iii) S_3 (D1→A ICT) (iv) S_4 (D2→A ICT)

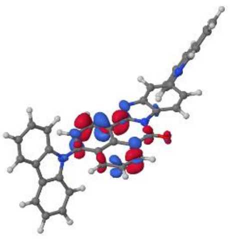

(v) T_1 (A $^3\pi\pi^*$)

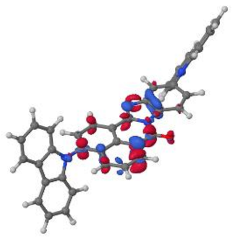

(vi) T_2 (A $^3\pi\pi^*$)

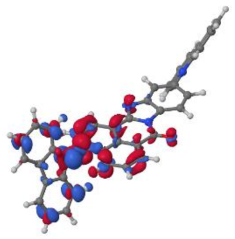

(vii) T_3 (D2→A ICT)

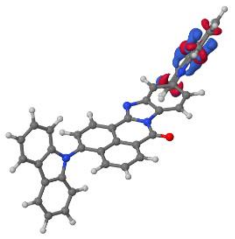

(viii) T_4 (D1 $^3\pi\pi^*$)

SI-2 Fluorescence Emission Spectra

In this section a simulation of the fluorescence emission spectra[1-5] for the **53.X** and the **56.X** series of compounds is presented and discussed. For each compound under study, we searched for minima on the PES of the S_1 state. Compound **53.4**, **56.1**, **56.3**, and **56.4** proved to have two minima on the PES of the S_1 state. The question arises of what the populations of the two excited-state conformers will be following photoexcitation. In what follows, we assumed that the two excited-state conformers of each compound can interconvert with one another, and that they establish a quasi-equilibrium. Building on that, we estimated the populations of the two excited-state conformers with the use of the Boltzmann distribution. We furthermore assumed a temperature of 300 K. The calculated vertical emission energies are listed in **Table S3** below. For compounds **53.1**, **53.2**, and **53.3**, we find only a conformer on the PES of the S_1 state each, which is the D1-*eq*,D2-*eq* conformer. For compound **53.4**, in turn, we find two conformers on the PES of the S_1 state: the D1-*eq*, D2-*eq*, and the D1-*ax*, D2-*eq* conformer. The D1-*eq*,D2-*ax* conformer is higher in energy than the D1-*ax*,D2-*eq* conformer by roughly 34 kJ/mol. Given the large energy difference between the D1-*eq*,D2-*eq* and the D1-*ax*,D2-*eq* conformers, we estimate that the D1-*eq*,D2-*eq* conformer will not be populated to a significant extent. It follows that all four compounds in the **53.X** series predominantly emit from D1-*eq*,D2-*eq* geometries. In all cases, the calculated emission energy is around 2.2 eV, which corresponds to a wavelength of roughly 560 nm. This most likely corresponds to the E1 band that is seen experimentally at around 500 nm. Among the **56.X** series of compounds, compounds 56.1, **56.3**, and **56.4** emit from structures with intermediate D2→A ^{1}ICT and $^1\pi\pi^*$ character. As a result, these compounds show large oscillator strengths for $S_1 \rightarrow S_0$ vertical emission. Conversely, compound **56.2** adopts a twisted ICT geometry (TICT) in which the D2 group is roughly perpendicular to the A moiety. In this compound, the S_1 state is a pure ICT state, with no admixture of $^1\pi\pi^*$ character. For this reason, this compound shows a very low oscillator strength for $S_1 \rightarrow S_0$ vertical emission.

Table S3 Calculated vertical emission energies for compounds in the **53.X** and the **56.X** series (ΔE). f is the corresponding oscillator strength. x is the estimated population of the given excited-state conformer.

Compound	Conformer in S_1	x	ΔE, eV	f
**53.1**	D1-*eq*,D2-*eq*	1	2.211	$< 1 \times 10^{-4}$
**53.2**	D1-*eq*,D2-*eq*	1	2.210	$< 1 \times 10^{-4}$
**53.3**	D1-*eq*,D2-*eq*	1	2.252	$< 1 \times 10^{-4}$
**53.4**	D1-*eq*,D2-*eq*	1.00	2.249	$< 1 \times 10^{-4}$
	D1-*ax*,D2-*eq*	1×10^{-6}	2.351	$< 1 \times 10^{-4}$
**56.1**	I	0.50	2.828	0.703
	II	0.50	2.829	0.704
**56.2**	TICT	1	3.066	1×10^{-4}
**56.3**	I	0.48	2.819	0.840
	II	0.52	2.816	0.839
**56.4**	I	0.50	2.940	0.639
	II	0.50	2.940	0.641

SI-3 Cyclic Voltammetry

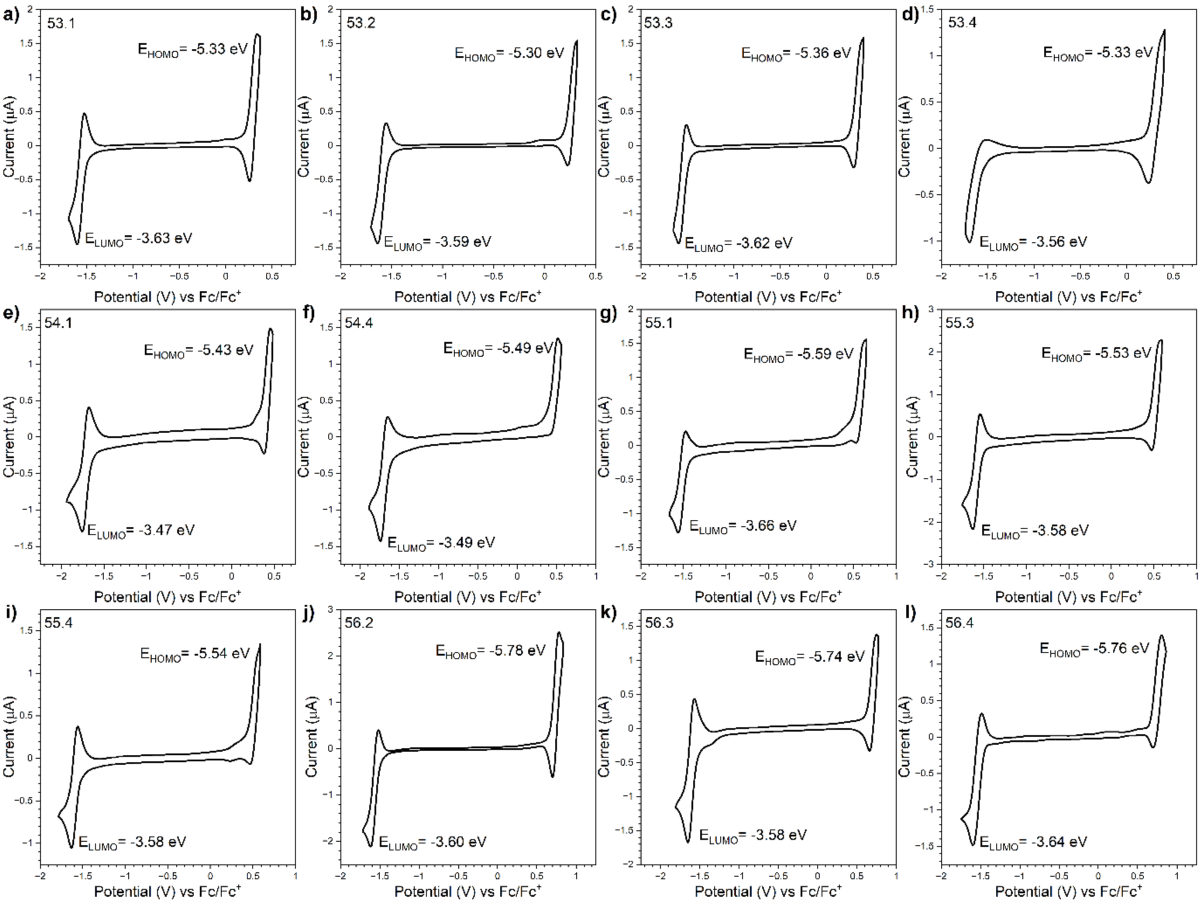

Figure S3: CV of 1 mM of compounds in 0.1 M Bu_4NBF_4 in DCM electrolyte (a-l) at a scan rate of 50 mV/s.

Cyclic voltammograms (CV) of all compounds were recorded in 0.1 M Bu_4NBF_4 electrolyte solution of dichloromethane three electrodes arrangement being a working electrode (platinum disc), a reference electrode (silver wire) and a counter electrode (platinum wire). CV of all compounds were calibrated with ferrocene/ferrocenium ion (Fc/Fc^+) redox couple as the internal standard. The oxidation and reduction potentials, and HOMO and LUMO levels, were depicted in **Table S4**. All compounds bearing phenoxazine donors showed one prominent one-electron reversible redox peaks. In first set of compounds, there was no significant difference was found in the oxidation and reduction potential of all the isomers. But the donor connected to the 3,11 positions to the acceptor (**53.1**) shown relatively larger E_g than the molecule which has the donor connection to the 3,10-,4,10-,4,11- positions to the acceptor. Similarly, For the dye sets (ISB bearing isomers), (DMAC bearing isomers) having the same trends of E_g, **54.1**<**54.4** and **55.1**<**55.3**<**55.4**. Whereas in the case **56.X** series, the E_g trend is exactly reversed, **56.4**<**56.3**<**56.2**. This deviation can be due to presence of bulky group (t-Bu) in the donor moiety, which increase the higher electron donation towards the acceptor unit. The HOMO-LUMO values obtained from this electrochemical measurement will be used to design the device assembly.

Table S4 Electrochemical parameters extracted from cyclic voltammograms.

Compound	E_{HOMO} (eV)	E_{LUMO} (eV)	Band gap (eV)
**53.1**	-5.33	-3.63	-1.70
**53.2**	-5.30	-3.59	-1.71
**53.3**	-5.36	-3.62	-1.74
**53.4**	-5.33	-3.56	-1.77
**54.1**	-5.43	-3.47	-1.96
**54.4**	-5.49	-3.49	-2.00
**55.1**	-5.59	-3.66	-1.93
**55.3**	-5.53	-3.58	-1.95
**55.4**	-5.54	-3.58	-1.96
**56.2**	-5.78	-3.60	-2.18
**56.3**	-5.74	-3.58	-2.16
**56.4**	-5.76	-3.64	-2.12

SI-4 Additional steady state photophysics

Photophysics. All steady-state absorption spectroscopy was performed on an HP 8453 spectrophotometer, using clean 1 cm path-length UV-visible spectroscopic grade quartz cuvettes (Aeika Cells). Steady-state photoluminescence spectra were performed on a Jobin Yvon Horiba Fluoromax 3, with solvent studies performed in clean 1 cm path-length photoluminescence cuvettes (Aeika Cells) and temperature dependent film photoluminescence films studies performed on within a liquid N_2 cooled cryostat (Janis Research).

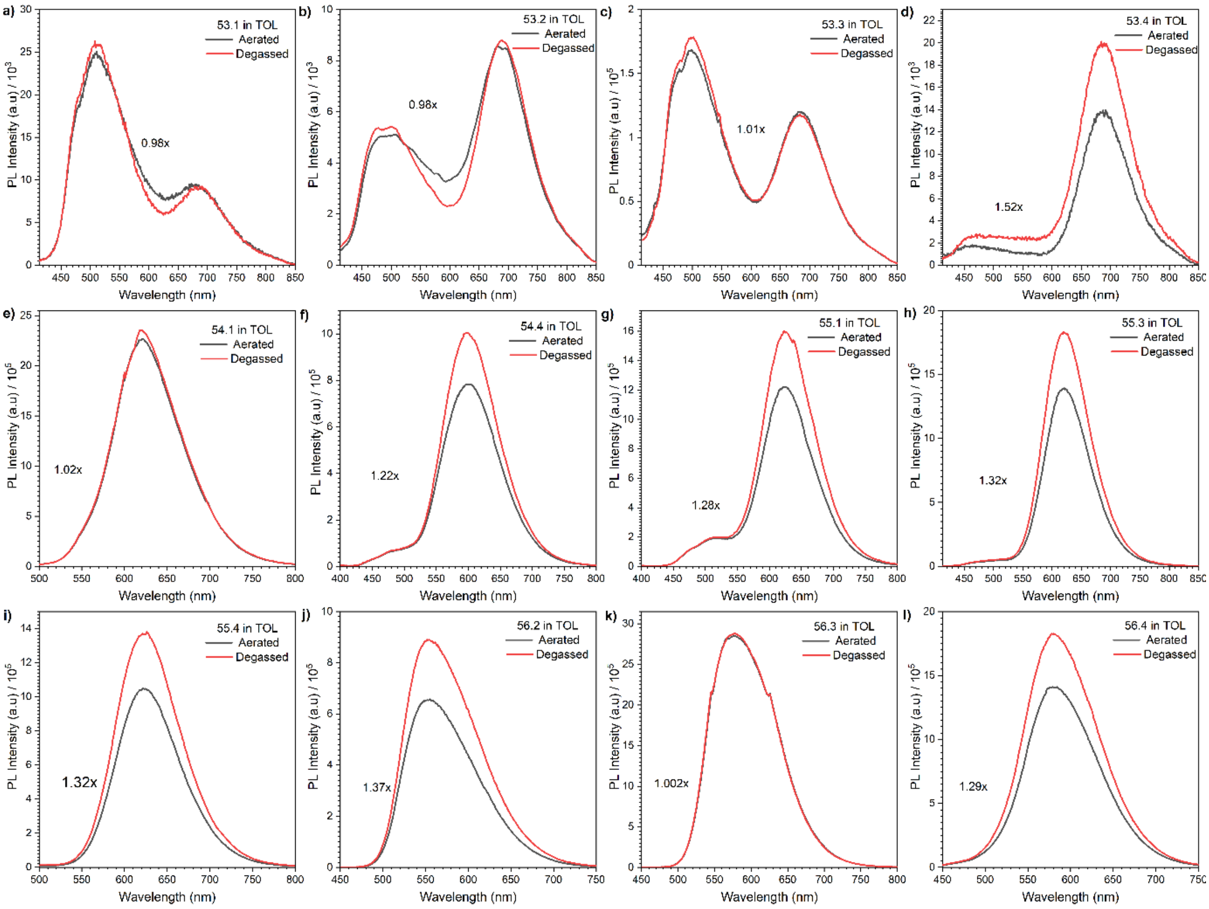

Figure S4: steady-state PL emission in degassed and aerated conditions (a-l): compounds in Toluene (c 10^{-5} M) diluted solution. Recorded at 300K using λ_{ex} = 355 nm.

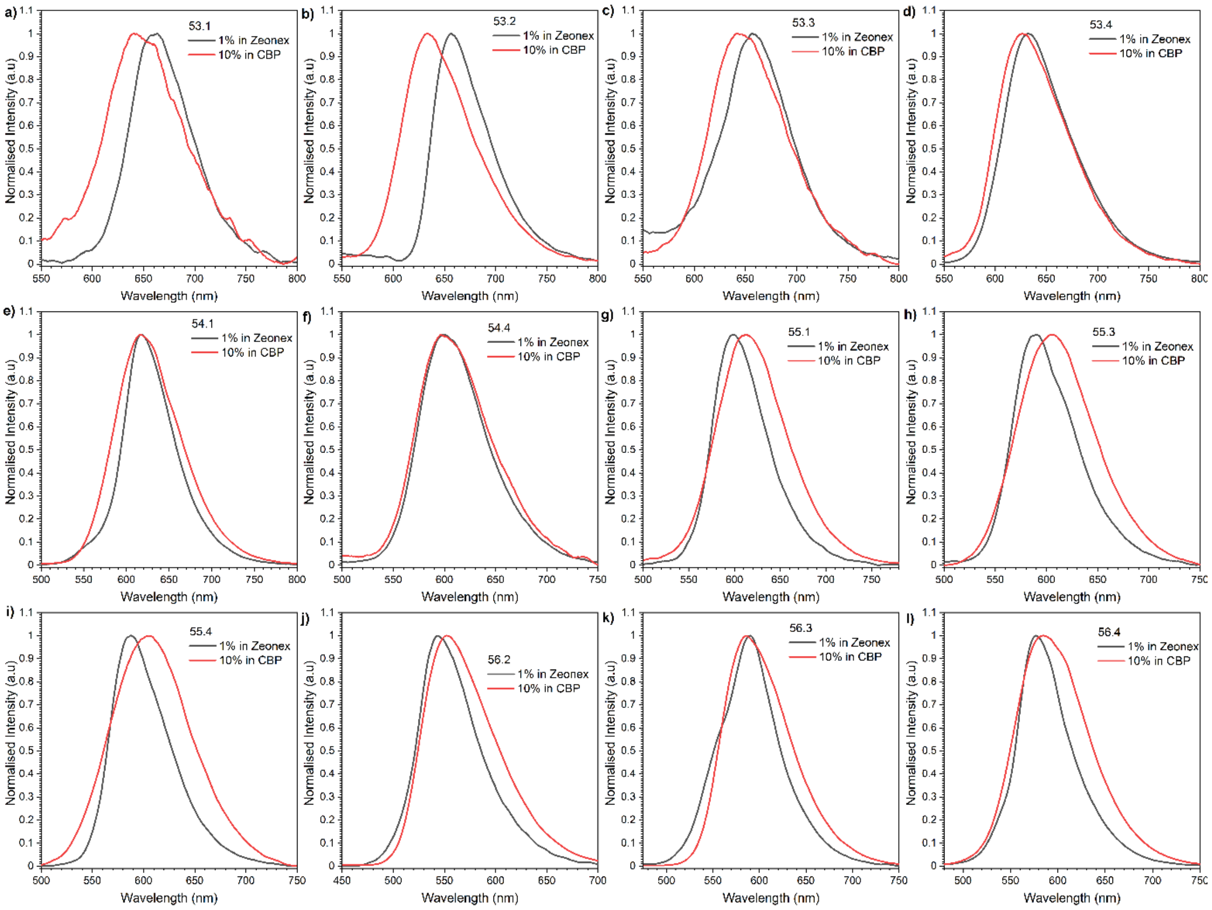

Figure S5: PL spectra of compounds in solid Zeonex and CBP matrix (a-l). Recorded at 300K using λ_{ex} = 355 nm.

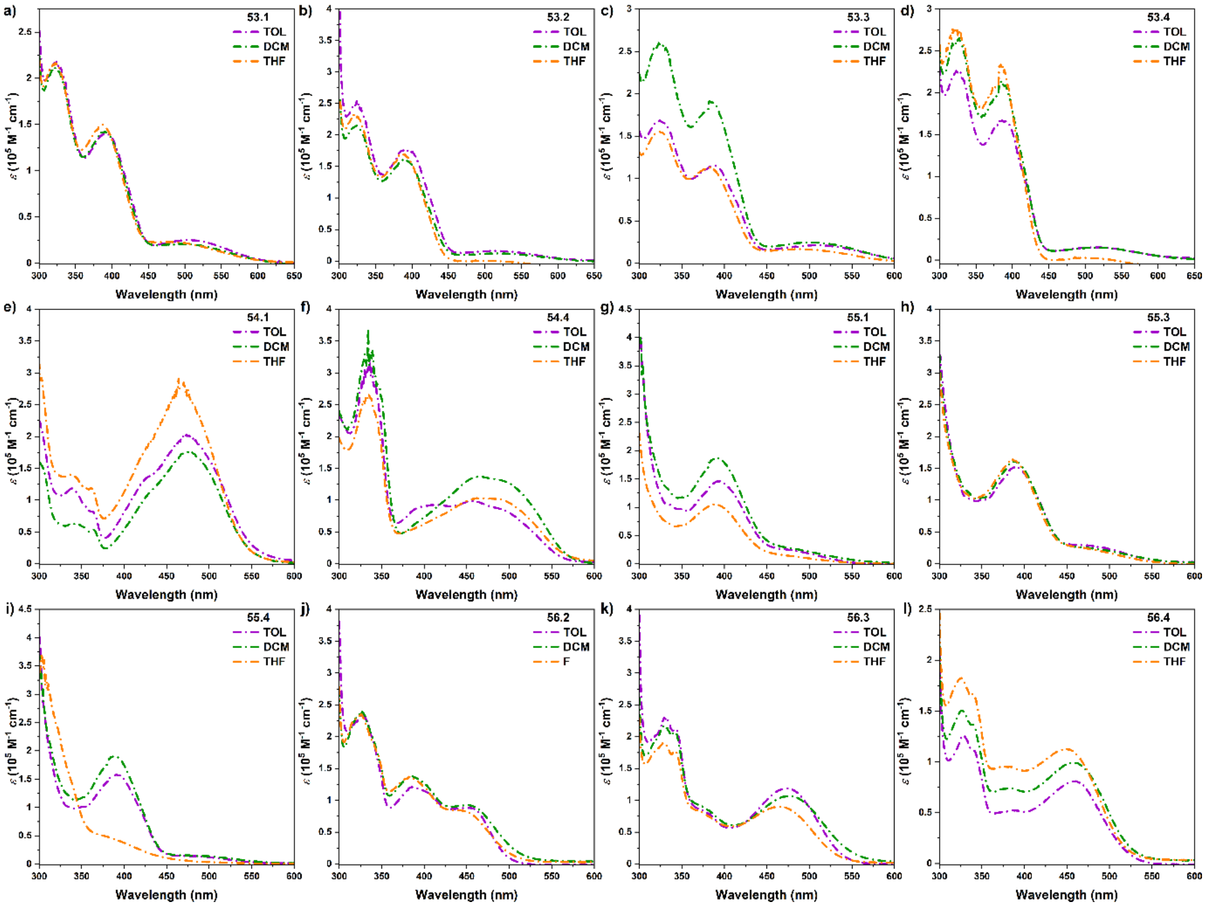

Figure S6: Absorption spectra of investigated compounds (a-l) in the different solvents (c 10^{-5} M).

SI-5 Time resolved photophysics

Samples preparation:

Samples in the Zeonex matrix were prepared from toluene solvents, where firstly pure compound and pure zeonex were dissolved. As next step, samples were mixed to obtain particular concentration of the compound in zeonex matrix (for example 1%), then the layer was spin coated at rate 2000 RPM on sapphire substrate to form uniform layer.
Samples in the CBP matrix were prepared by thermal evaporation in the high vacuum conditions through the adjusted emitter evaporation rate to the constant CBP rate at 1 A/s to obtain particular concentration.

Measurements:

Photophysics. Phosphorescence, prompt fluorescence (PF), and delayed fluorescence (DF) spectra and decays were recorded using nanosecond gated luminescence and lifetime measurements (from 400 ps to 1 s) using either third harmonics of a high energy pulsed DPSS laser emitting at 355 nm (Q-Spark A50-TH-RE). Emission was focused onto a spectrograph and detected on a sensitive gated iCCD camera (Stanford Computer Optics) having a sub-nanosecond resolution. PF/DF time-resolved measurements were performed

by exponentially increasing gate and integration times. Temperature-dependent experiments were conducted using a helium cryostat (Janis Research) under a vacuum.

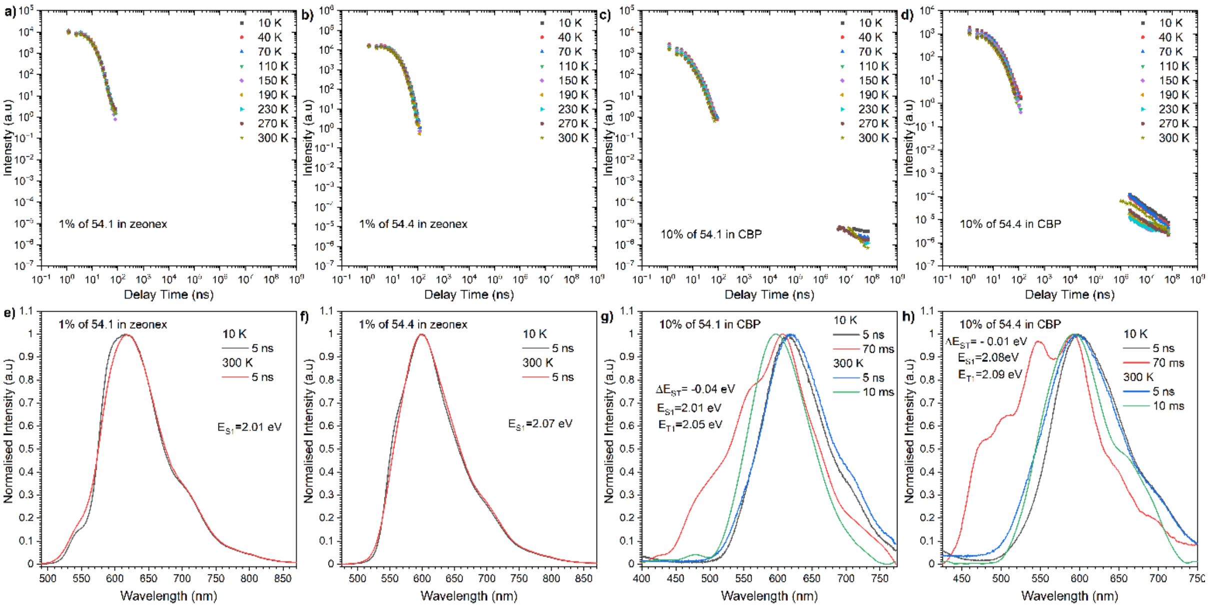

Figure S7: Time-resolved PL decay profiles (intensity vs. delay time) (a-d) and spectra (e-h) of compounds **54.1** and **54.4** in Zeonex® (a,b & e,f) and CBP (c,d & g,h) The energies correspond to the maximum emission peaks and λ_{ex} = 355 nm.

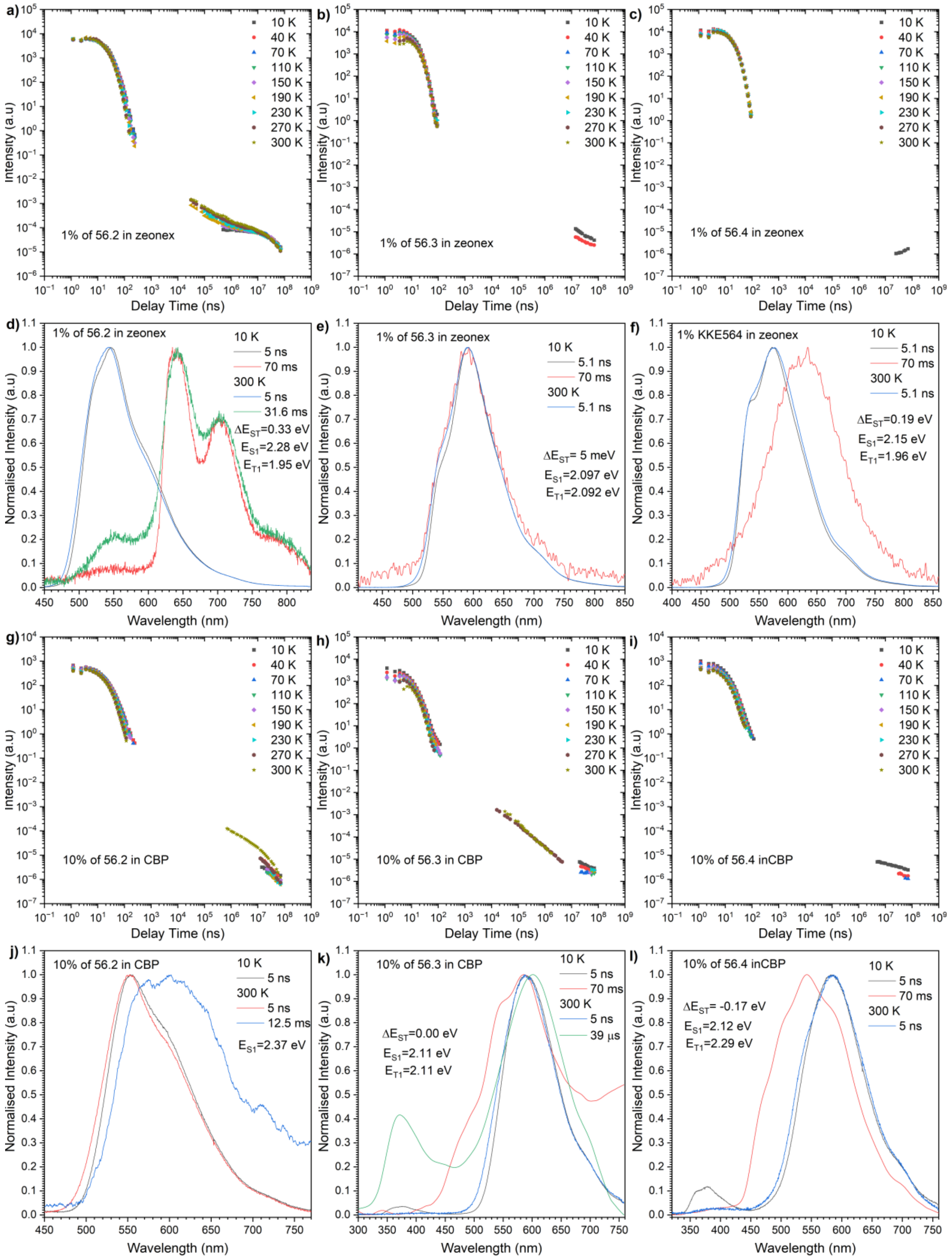

Figure S8: Time-resolved PL decay profiles (intensity vs. delay time) (a-c &g-i) and spectra (d-f & j-i) of compounds **56.2** - **56.4** in Zeonex® (a-f) and CBP (g-l) The energies correspond to the maximum emission peaks and λ_{ex} = 355 nm.

SI-6 OLED devices

Devices. HAT-CN (Hexaazatriphenylenehexacarbonitrile) was used as a Hole Injection Layer (HIL), NPB (*N*,*N′*-di(1-naphthyl)-*N*,*N′*-diphenyl-(1,1′-biphenyl)-4,4′-diamine) was used as a Hole Transport Layer (HTL), TmPyPB (1,3,5-Tri(m-pyridin-3-ylphenyl)benzene) was introduced as an Electron Transport Layer (ETL). Lithium fluoride (LiF) and aluminium were used as the cathode. Organic semiconductors and aluminium were deposited at a rate of 1 $Ås^{-1}$, and the LiF layer was deposited at 0.1 $Ås^{-1}$. CBP 4,4′-bis(*N*-carbazolyl)-1,1′-biphenyl, was used as host for all emitters. All materials were purchased from Sigma Aldrich or Lumtec and were purified by temperature-gradient sublimation in a vacuum. OLEDs have been fabricated on pre-cleaned, patterned indium-tin-oxide (ITO) coated glass substrates with a sheet resistance of 20 Ω/sq and ITO thickness of 100 nm. All small molecules and cathode layers were thermally evaporated in a Kurt J. Lesker Spectros evaporation system under pressure of 10^{-7} mbar without breaking the vacuum. The sizes of pixels were 4 mm^2, 8 mm^2 and 16 mm^2. Each emitting layer has been formed by co-deposition of dopant and host at the specific rate to obtain 10% content of the emitter. The characteristics of the devices were recorded using a 6-inch integrating sphere (Labsphere) inside the glovebox connected to a Source Meter Unit and Ocean Optics USB4000 spectrometer.

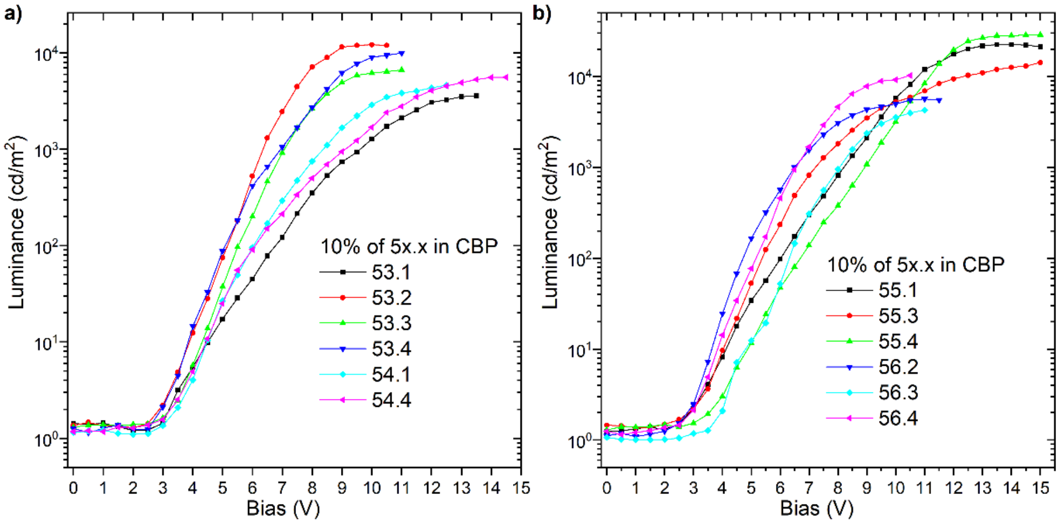

Figure S9: OLED voltage-luminance (a, b) relationship .

SI-7 TGA/DSC and DSC (heating/cooling) measurements

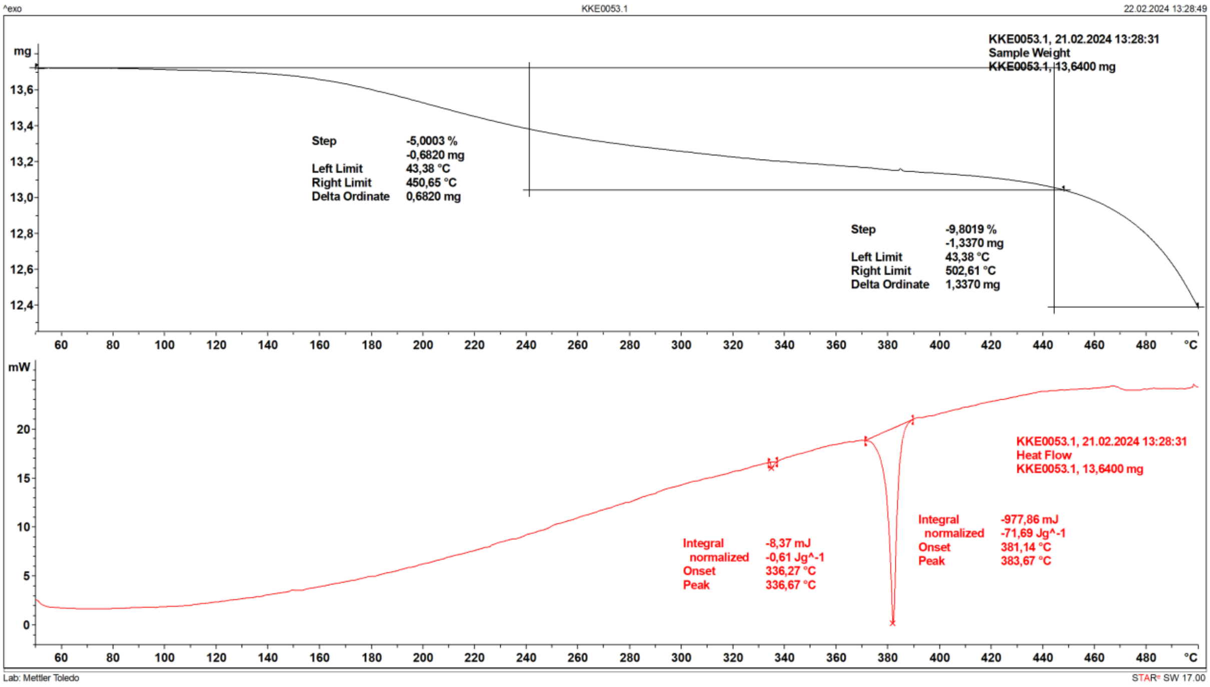

Figure S10: TGA/DSC curve of 53.1

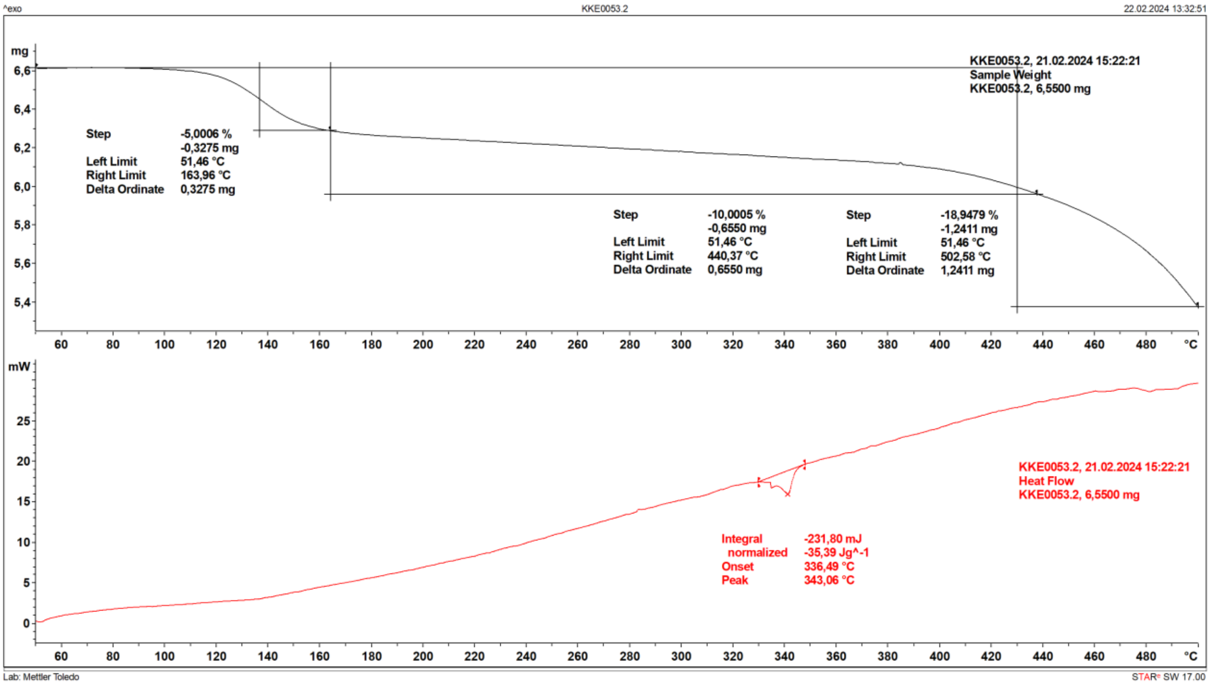

Figure S11: TGA/DSC curve of 53.2

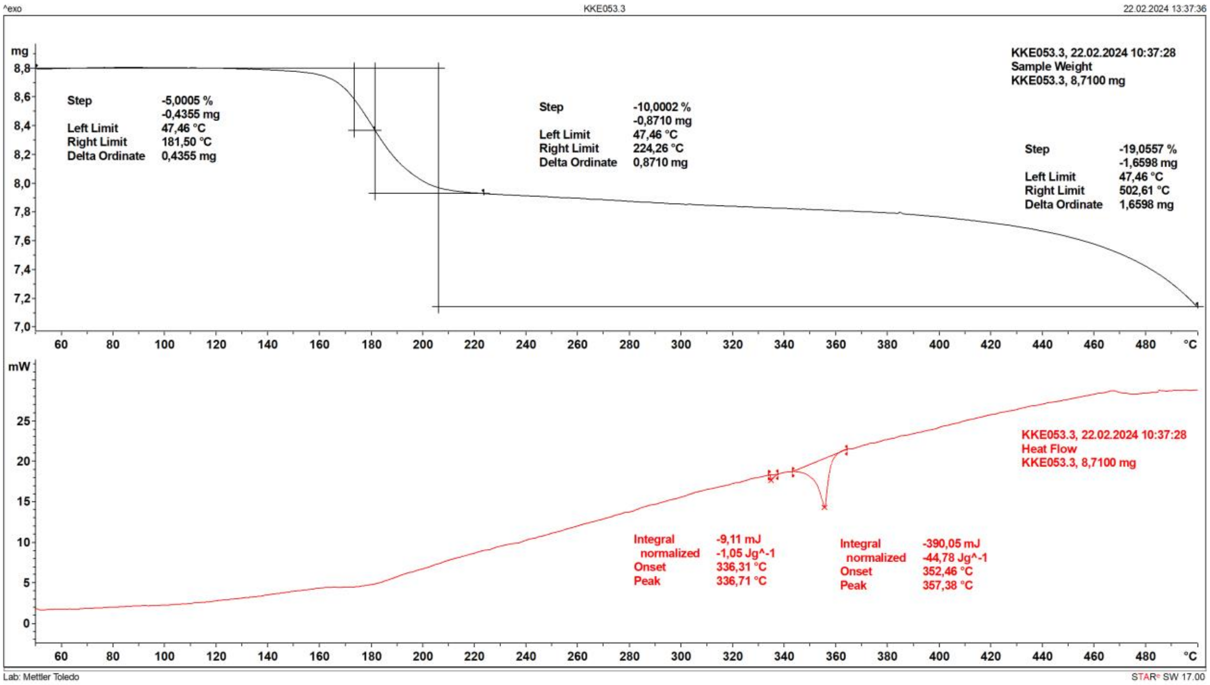

Figure S12: TGA/DSC curve of 53.3

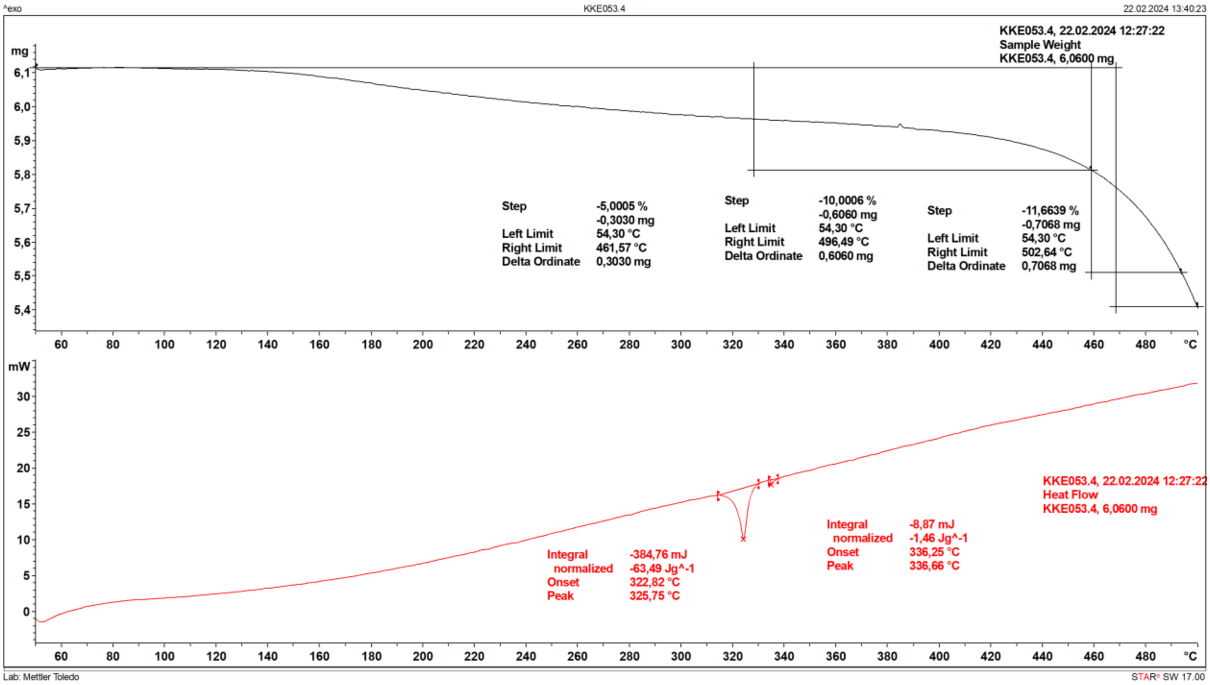

Figure S13: TGA/DSC curve of 53.4

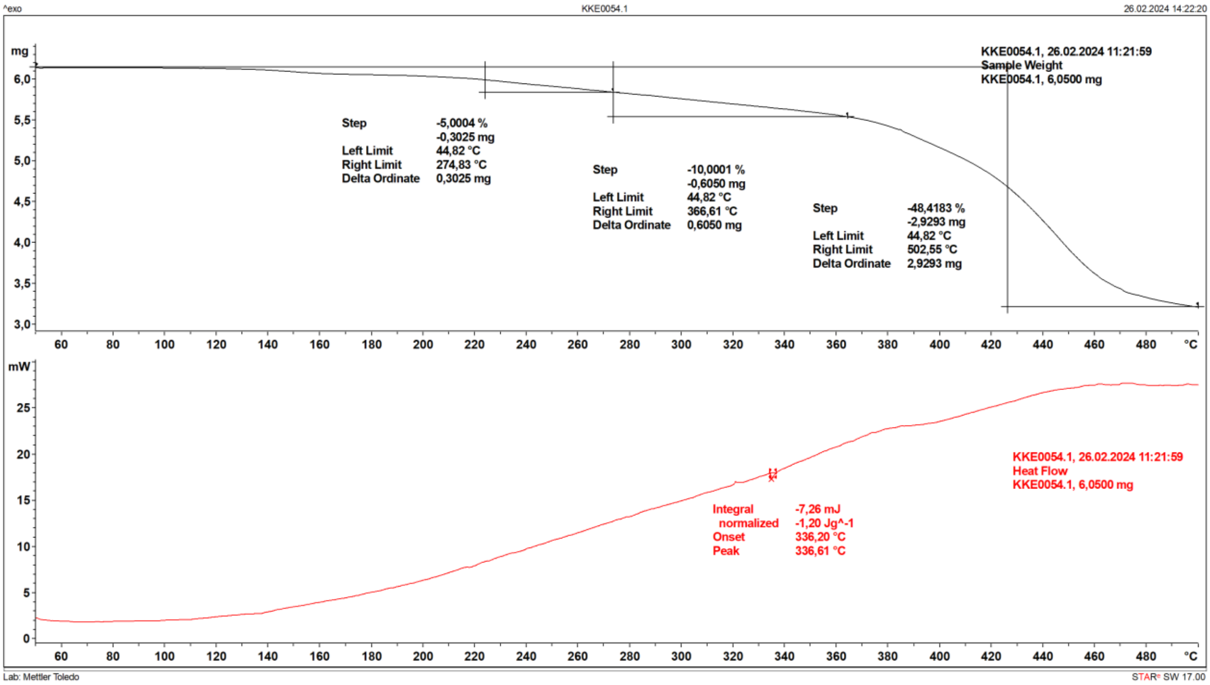

Figure S14: TGA/DSC curve of 54.1

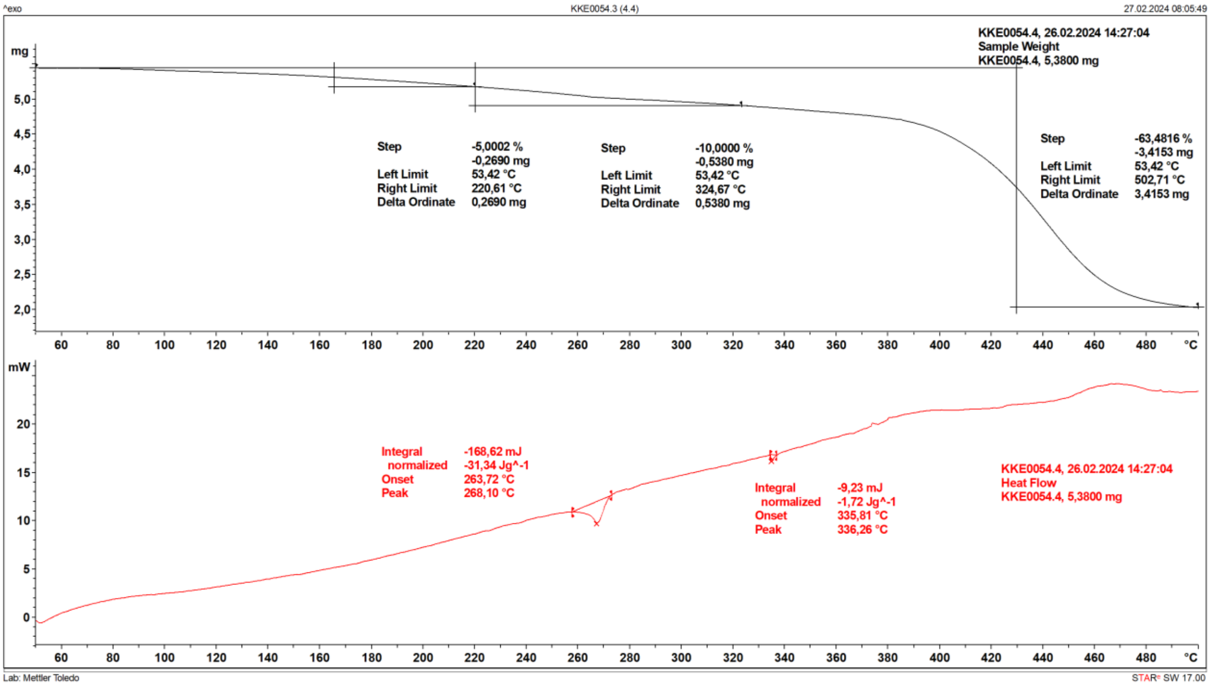

Figure S15: TGA/DSC curve of 54.4

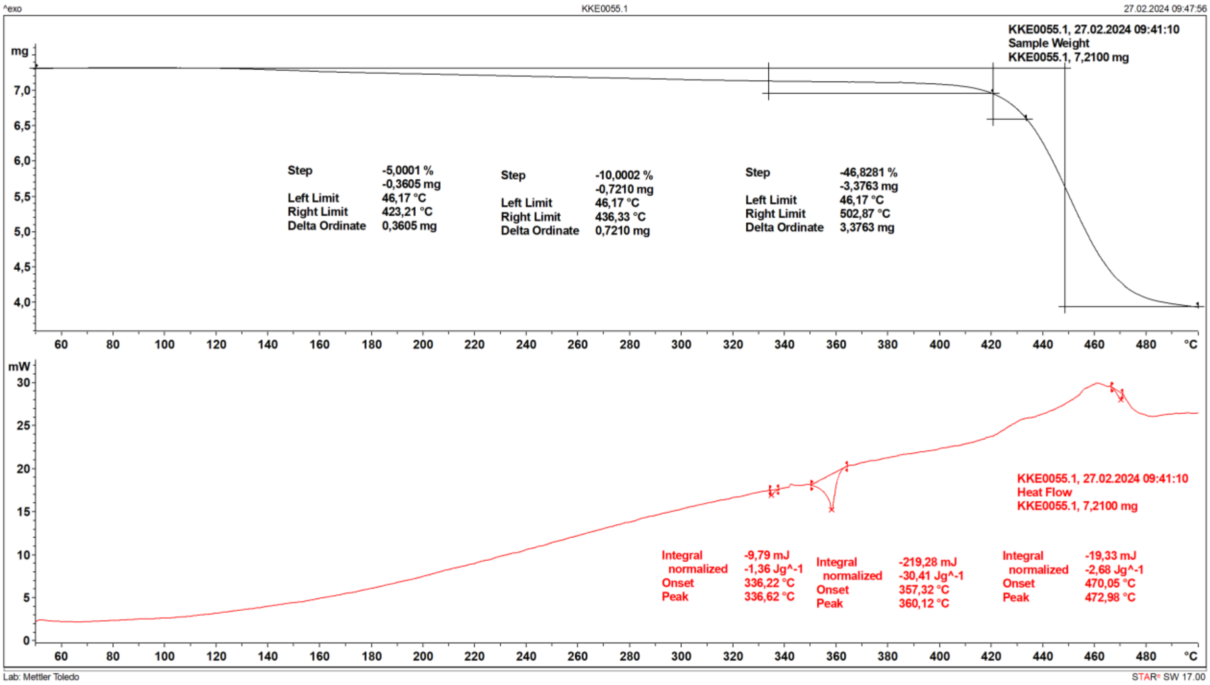

Figure S16: TGA/DSC curve of 55.1

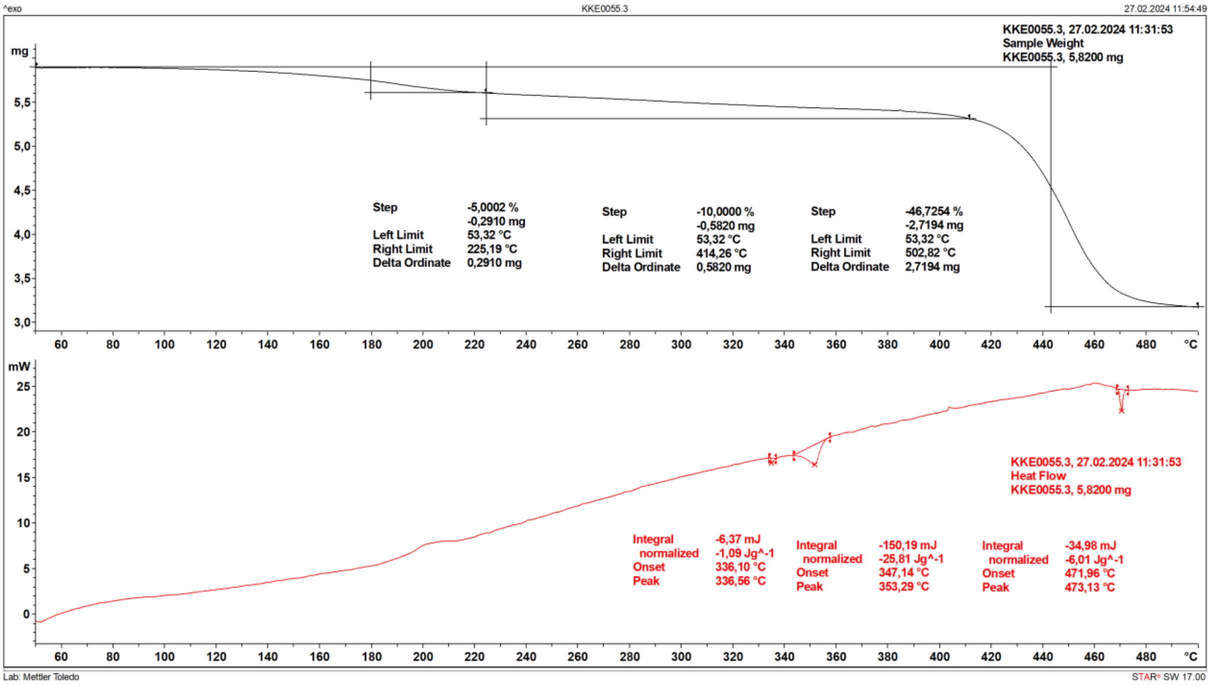

Figure S17: TGA/DSC curve of 55.3

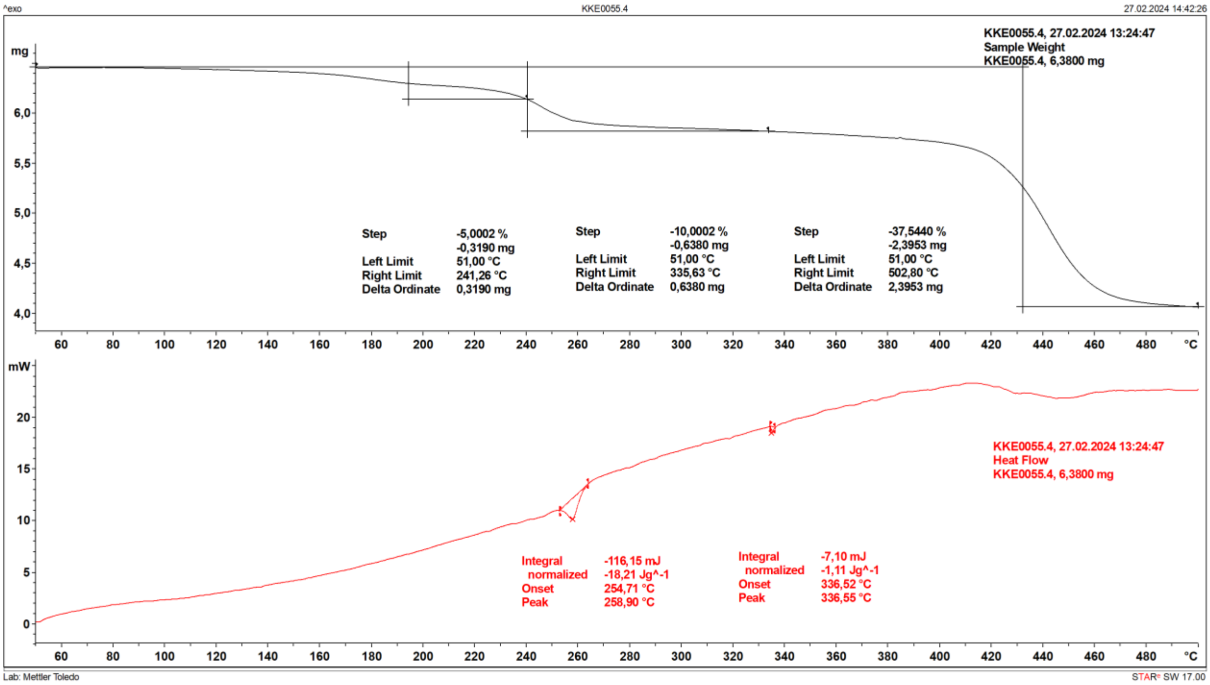

Figure S18: TGA/DSC curve of 55.4

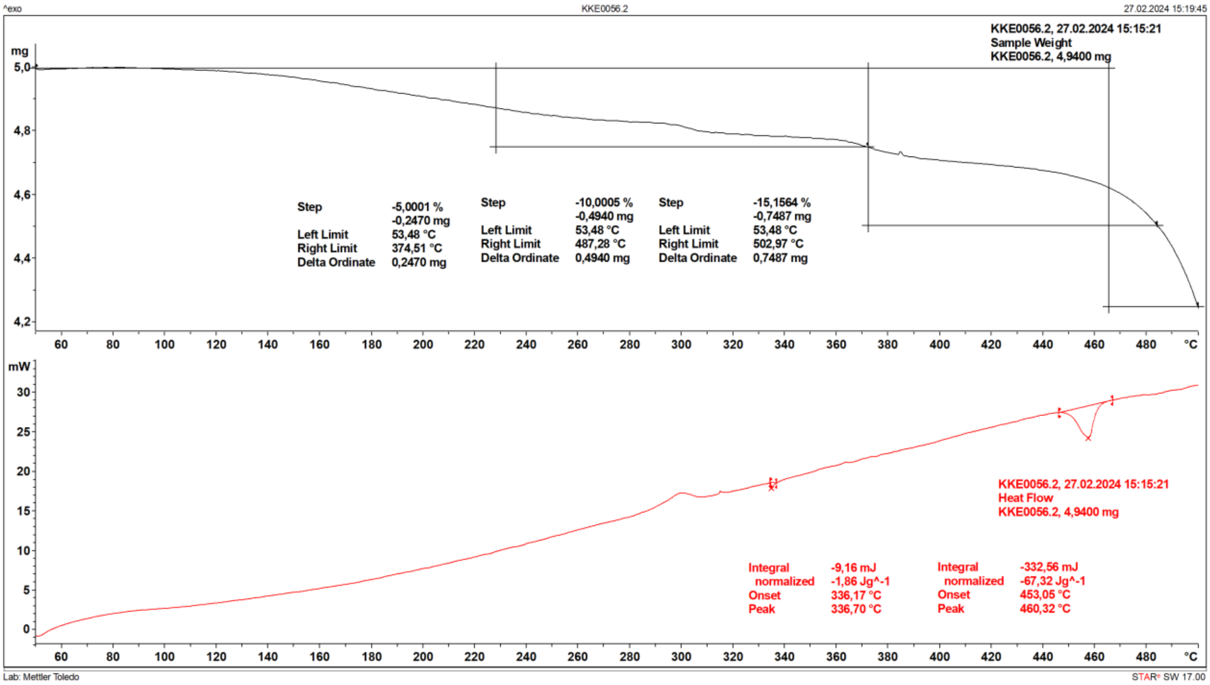

Figure S19: TGA/DSC curve of 56.2

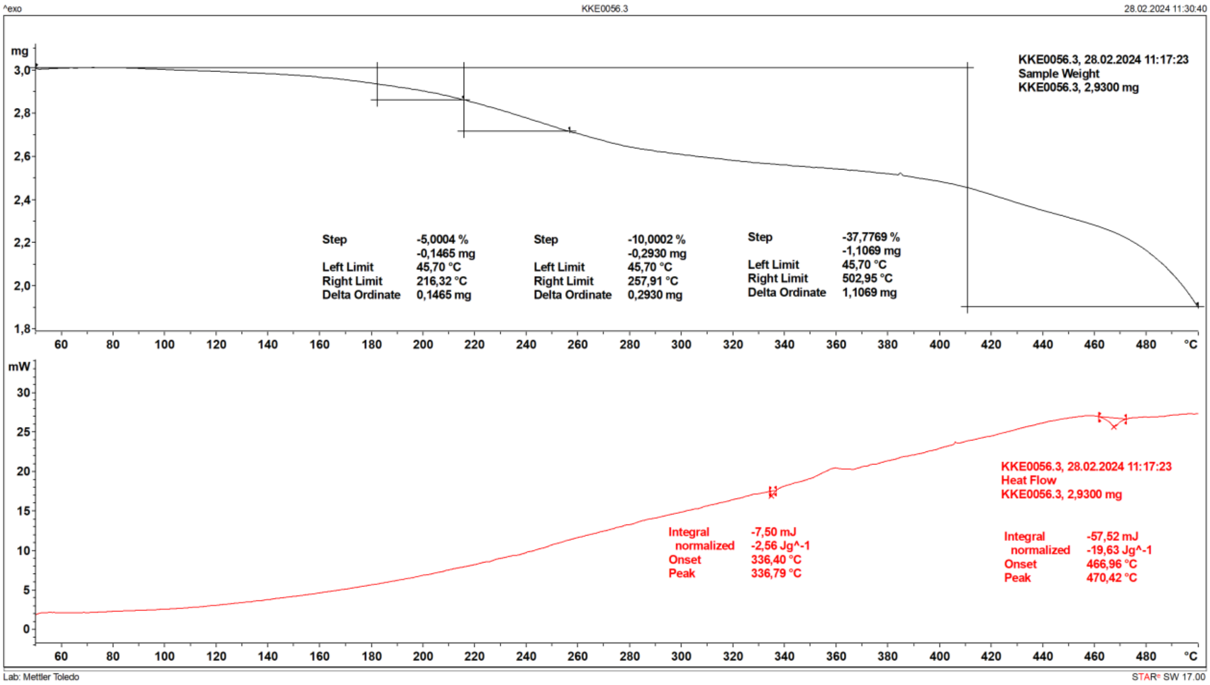

Figure S20: TGA/DSC curve of 56.3

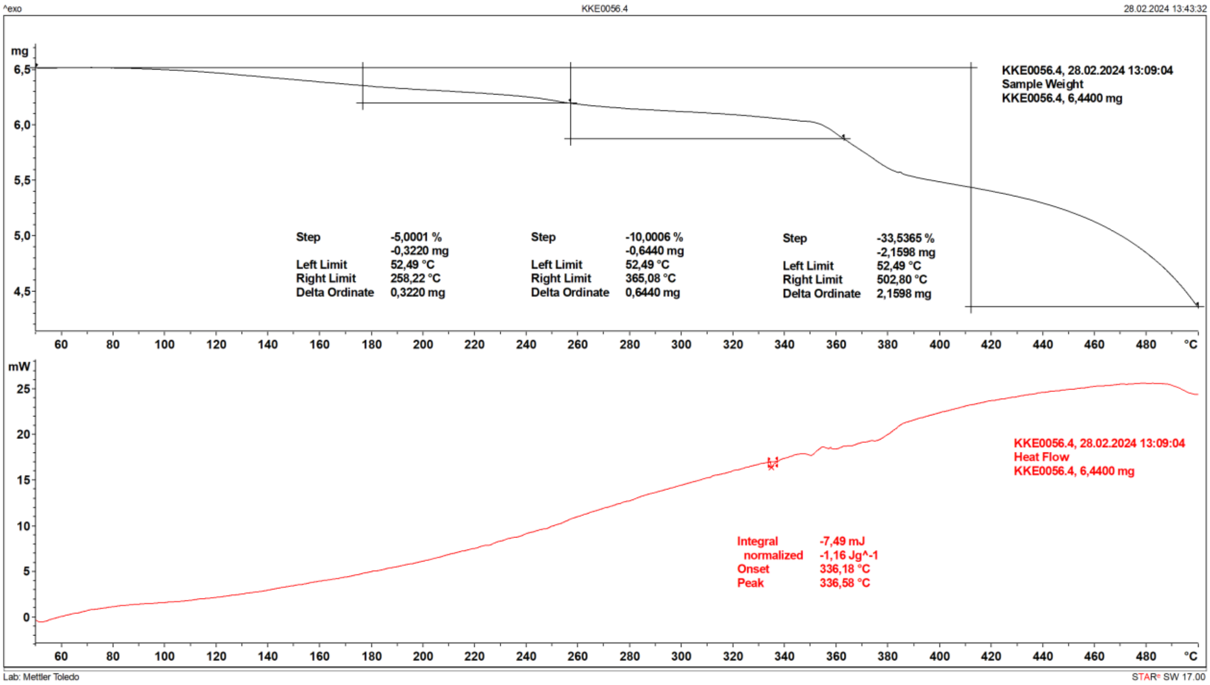

Figure S21: TGA/DSC curve of 56.4

SI-8. Synthesis - general information

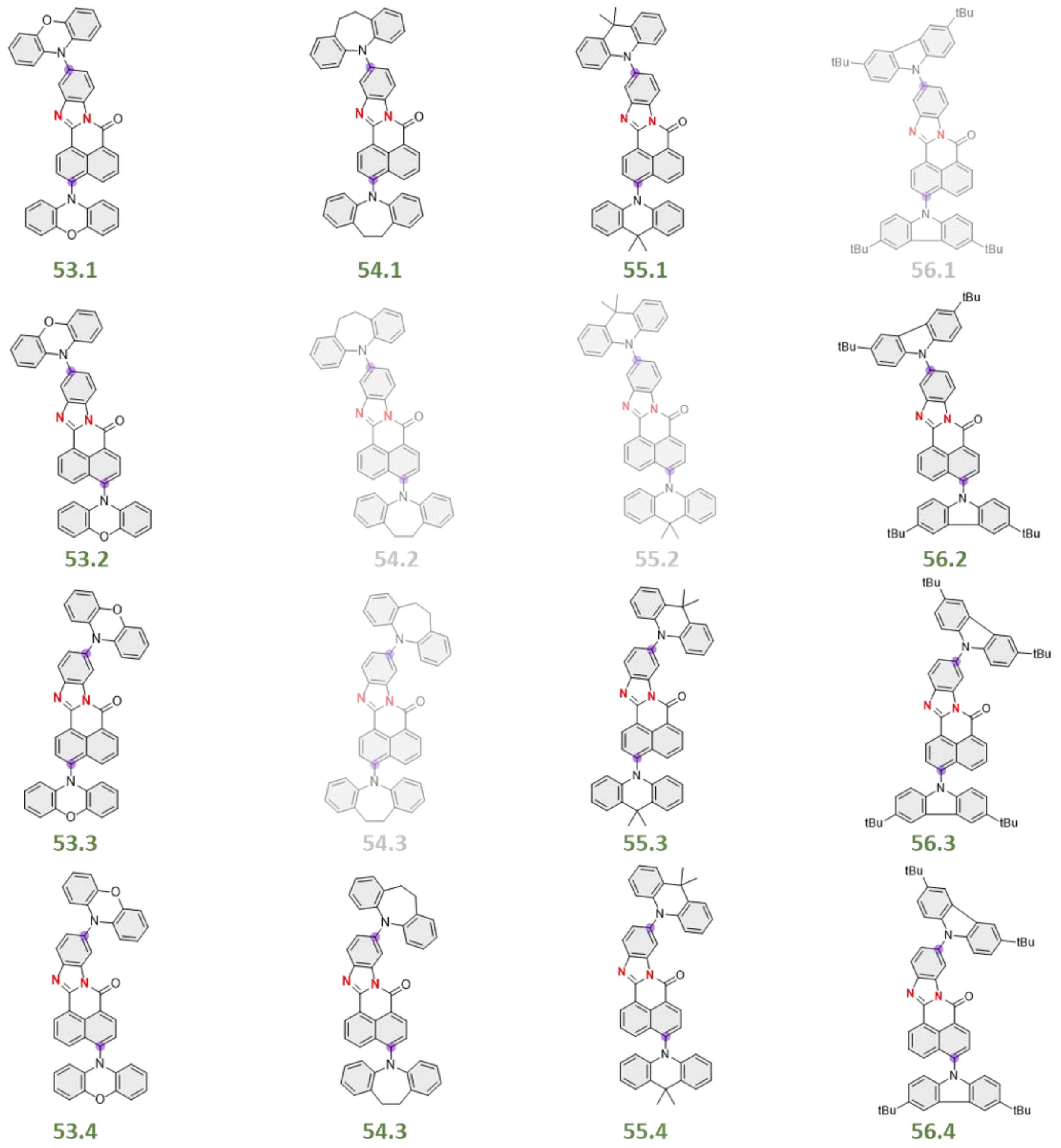

Figure S22 All possible variants of regioisomers designed in these studies. Compounds **54.2, 54.3, 55.2, 56.1** were isolated as a mixture, hence they were not subjected to photophysical analyses.

All solvents and commercially available reagents were purchased as reagent grade and were used without further purification, unless otherwise stated. NMR spectra were recorded on **Varian-Agilent 500 MHz or** 600 MHz and calibrated using residual nondeuterated solvent ($CHCl_3$ – 7.26 ppm ^{1}H NMR, 77.16 ppm ^{13}C NMR) as an internal reference. High-resolution mass spectra (HRMS) were recorded on a Waters AutoSpec Premier instrument using electron ionization (EI).
A general note on the reaction of dibromo substituted benzo[de]benzo[4,5]imidazo[2,1-a]isoquinolin-7-one with phenoxazine, 9,10-dihydrodibenzazepine, 9,9-dimethyl-10H-acridine and 3,6-di-tert-butylcarbazole, respectively: each reaction leads to four regioisomers of the product as a result of using a mixture of regioisomers as a substrate. Separation of the products was challenging and not always successful; therefore, isomers that were not isolated and not analyzed are presented in the schemes in gray.

SI-8.1 Synthetic protocols

<u>Synthesis of a starting material</u>

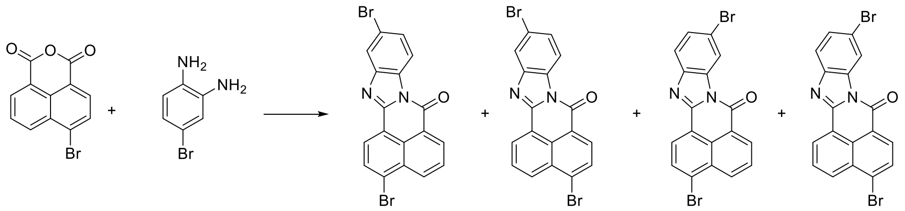

In a pressure flask, bromo-naphthalic anhydride (5 mmol, 1.0 equiv.), 1,2-diamino-4-bromobenzene (5 mmol, 1.0 equiv.) and 15 mL of acetic acid were placed under an argon atmosphere. The mixture was degassed with argon for 15 minutes, then the flask was tightly closed, and the reaction was carried out at the boiling point of acid (118 ^{o}C) for 4 hours. The completion of the reaction was confirmed with thin layer chromatography (TLC). The mixture was poured into a beaker with ice and water. The obtained precipitate was filtered and then crystallized in toluene to obtain a mixture of four regioisomers. The mixture was used without further purification in the next synthetic step with phenoxazine, 9,10-dihydrodibenzazepine, 9,9-dimethyl-10H-acridine and 3,6-di-tert-butylcarbazole (see below).

<u>Synthesis of compounds **53.1-4**</u>

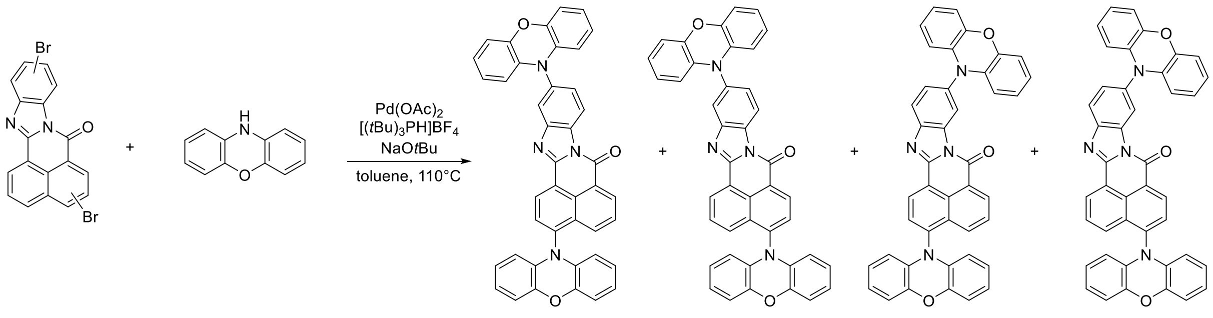

53.1 **53.2** **53.3** **53.4**

To the mixture of isomers dibromo substituted benzo[de]benzo[4,5]imidazo[2,1-a]isoquinolin-7-one (500 mg; 1.17 mmol), phenoxazine (535 mg; 2.92 mmol; 2.5 equiv.), sodium tert-butoxide (281 mg; 2.92 mmol; 2.5 equiv.) 15 ml of degassed toluene were added. The mixture was degassed again by bubbling argon through the solution for 5 minutes. Tri-tert-butylphosphonium tetrafluoroborate (204 mg; 0.7 mmol, 20 mol%) and

palladium acetate (53 mg; 0.234 mmol, 20 mol%) were then added. The reaction mixture was heated at 110°C for 16 hours. Water was added to the reaction mixture, followed by extraction with methylene chloride. The organic layer was dried over Na_2SO_4.The reaction yielded a mixture of regioisomers of the product, which were subsequently separated through column chromatography using a toluene and ethyl acetate mixture as the eluent.

Compound ***53.1***
^{1}H NMR ($CDCl_3$, 600 MHz): δ 9.06 (d, *J* = 7.7 Hz, 1H), 8.88 (dd, *J* = 7.2, 0.9 Hz, 1H), 8.77 (d, *J* = 8.4 Hz, 1H), 8.59 (dd, *J* = 8.4, 0.9 Hz, 1H), 8.01 (d, *J* = 1.6 Hz, 1H), 7.92 (d, *J* = 7.7 Hz, 1H), 7.85 (dd, *J* = 8.3, 7.3 Hz, 1H), 7.47 (dd, *J* = 8.4, 1.9 Hz, 1H), 6.81 (dd, *J* = 8.0, 1.3 Hz, 2H), 6.74 – 6.70 (m, 2H), 6.66 – 6.60 (m, 4H), 6.60 – 6.54 (m, 4H), 5.98 – 5.93 (m, 2H), 5.81 (dd, J = 8.0, 1.2 Hz, 2H) ppm. ^{13}C NMR ($CDCl_3$, 151 MHz): δ 160.3, 149.8, 146.0, 144.1, 144.0, 140.0, 136.8, 134.6, 134.1, 132.9, 131.8, 131.4, 131.3, 131.2, 129.6, 128.8, 128.4, 128.3, 124.3, 123.7, 123.4, 123.3, 122.4, 121.5, 121.3, 118.5 116.1, 115.5, 113.6, 113.4 ppm. HRMS (EI): m/z calculated for: $C_{42}H_{24}N_4O_3$ [$M^{\cdot+}$] = 632.1848; found: 632.1860, yield: 22% (0.16g)
Compound ***53.2***
^{1}H NMR ($CDCl_3$, 600 MHz): 9.04 (d, *J* = 7.7 Hz, 1H), 8.94 (d, *J* = 7.1 Hz, 1H), 8.79 (d, *J* = 8.4 Hz, 1H), 8.45 (d, *J* = 8.1 Hz, 1H), 7.97 (d, *J* = 7.7 Hz, 1H), 7.93 (d, *J* = 1.3 Hz, 1H), 7.85 – 7.81 (m, 1H), 7.49 (dd, *J* = 8.5, 1.4 Hz, 1H), 6.82 (dd, *J* = 8.0, 0.9 Hz, 2H), 6.75 – 6.70 (m, 4H), 6.66 (t, *J* = 7.6 Hz, 2H), 6.62 – 6.52 (m, 4H), 6.01 (d, J = 8.0 Hz, 2H), 5.76 (dd, *J* = 8.0, 1.0 Hz, 2H) ppm. ^{13}C NMR ($CDCl_3$, 151 MHz): 160.0, 150.4, 146.1, 144.1, 144.0, 143.4, 137.0, 134.7, 133.7, 133.5, 131.8, 131.4, 130.7, 129.6, 128.9, 128.5, 128.3, 128.2, 123.7, 123.6, 123.4, 123.1, 122.5, 121.8, 121.6, 118.5, 116.2, 115.6, 113.7, 113.6 HRMS (EI): m/z calculated for: $C_{42}H_{24}N_4O_3$ [$M^{\cdot+}$] = 632.1848; found: 632.1860; yield: 15% (0.11g)
Compound ***53.3***
^{1}H NMR ($CDCl_3$, 400 MHz): 9.09 (d, *J* = 7.7 Hz, 1H), 8.85 (d, *J* = 6.9 Hz, 1H), 8.64 (d, *J* = 1.5 Hz, 1H), 8.57 (d, *J* = 8.3 Hz, 1H), 8.12 (d, *J* = 8.5 Hz, 1H), 7.94 (d, *J* = 7.7 Hz, 1H), 7.86 – 7.79 (m, 1H), 7.48 (dd, *J* = 8.4, 1.7 Hz, 1H), 6.81 (d, *J* = 7.0 Hz, 2H), 6.75 – 6.69 (m, 4H), 6.69 – 6.63 (m, 2H), 6.62 – 6.52 (m, 4H), 6.01 (d, *J* = 8.1 Hz, 2H), 5.78 (dd, *J* = 7.9, 0.6 Hz, 2H) ppm. HRMS (EI): m/z calculated for: $C_{42}H_{24}N_4O_3$ [$M^{\cdot+}$] = 632.1848; found 632.1860; yield: 18% (0.13g)
Compound ***53.4***
^{1}H NMR ($CDCl_3$, 500 MHz): δ 8.96 (d, *J* = 7.7 Hz, 1H), 8.93 (d, *J* = 7.3 Hz, 1H), 8.70 (d, *J* = 1.7 Hz, 1H), 8.45 (dd, *J* = 8.5, 0.7 Hz, 1H), 8.08 (d, *J* = 8.4 Hz, 1H), 7.91 (d, *J* = 7.7 Hz, 1H), 7.83 (dd, *J* = 8.3, 7.4 Hz, 1H), 7.45 (dd, *J* = 8.4, 1.9 Hz, 1H), 6.81 (dd, *J* = 7.9, 1.3 Hz, 2H), 6.73 (td, *J* = 7.7, 1.3 Hz, 2H), 6.65 – 6.50 (m, 8H), 5.97 (d, J = 7.5 Hz, 2H), 5.78 (dd, J = 8.0, 1.2 Hz, 2H) ppm. ^{13}C NMR ($CDCl_3$, 125 MHz): δ 159.9, 150.6, 144.00, 143.96, 143.8, 143.2, 136.5, 134.6, 133.9, 133.6, 133.5, 131.3, 130.7, 129.6, 128.80, 128.78, 128.3, 128.2, 123.8, 123.6, 123.3, 122.6, 122.5, 121.9, 121.6, 119.0, 116.1, 115.5, 113.7, 113.5 ppm. HRMS (EI): m/z calculated for: $C_{42}H_{24}N_4O_3$ [$M^{\cdot+}$] = 632.1848; found 632.1860; yield: 17% (0.12g)

Synthesis of compounds **54.1-4**

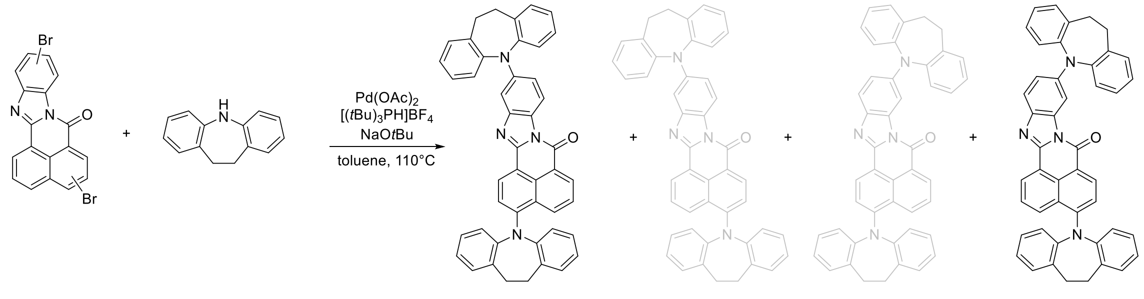

54.1 54.4

To the mixture of isomers of dibromo substituted benzo[de]benzo[4,5]imidazo[2,1-a]isoquinolin-7-one (500 mg; 1.17 mmol), 9,10-dihydrodibenzazepine (570 mg; 2.92 mmol ; 2.5 equiv.), sodium tert-butoxide (281 mg; 2.92 mmol; 2.5 equiv.) 15 mL of degassed toluene were added. The mixture was degassed again by bubbling argon through the solution for 5 minutes. Tri-tert-butylphosphonium tetrafluoroborate (204 mg; 0.7 mmol, 60 mol%) and palladium acetate (53 mg; 0.234 mmol, 20 mol%) were then added. The reaction mixture was heated at 110^{0}C for 16 hours. Water was added to the reaction mixture, followed by extraction with methylene chloride. The organic layer was dried over Na_2SO_4. The reaction yielded a mixture of regioisomers of the product, which were subsequently separated through column chromatography using a toluene and ethyl acetate mixture as the eluent.

*Compound **54.1***
^{1}H NMR ($CDCl_3$, 600 MHz): δ 8.68 (dd, *J* = 7.2, 0.8 Hz, 1H), 8.61 (d, *J* = 8.4 Hz, 1H), 8.20 (d, *J* = 8.9 Hz, 1H), 8.17 (dd, *J* = 8.6, 0.8 Hz, 1H), 7.50 (d, *J* = 7.4 Hz, 2H), 7.46 (d, *J* = 8.4 Hz, 1H), 7.43 (dd, *J* = 8.5, 7.4 Hz, 1H), 7.27 (m, 8H), 7.15 – 7.09 (m, 6H), 7.02 (d, *J* = 2.2 Hz, 1H), 6.72 (dd, *J* = 8.9, 2.4 Hz, 1H), 3.26 (s, 4H), 3.02 (s, 4H) ppm. ^{13}C NMR ($CDCl_3$, 150 MHz) δ 160.6, 150.1, 148.6, 148.1, 146.0, 145.5, 143.9, 138.7, 135.8, 132.7, 131.5, 131.2, 131.1, 130.5, 129.6, 128.3, 127.44, 127.40, 127.38, 126.3, 125.8, 125.7, 125.1, 124.3, 123.7, 119.0, 115.7, 114.4, 111.1, 102.6, 32.7, 31.0 ppm. HRMS (EI): m/z calculated for: $C_{46}H_{32}N_4O$ [$M^{\cdot+}$] = 656.2576; found 656.2582; yield: 19% (0.14g)
*Compound **54.4***
^{1}H NMR ($CDCl_3$, 600 MHz): δ 8.63 (dd, *J* = 7.3, 0.6 Hz, 1H), 8.47 (d, *J* = 8.6 Hz, 1H), 7.83 (d, *J* = 2.4 Hz, 1H), 7.80 (dd, *J* = 8.7, 0.6 Hz, 1H), 7.57 (d, *J* = 8.9 Hz, 1H), 7.53 (dd, *J* = 7.7, 0.7 Hz, 2H), 7.35 – 7.20 (m, 16H), 6.78 (dd, *J* = 8.9, 2.5 Hz, 1H), 3.22 (s, 4H), 3.04 (s, 4H) ppm. ^{13}C NMR ($CDCl_3$, 150 MHz): δ 160.8, 150.6, 148.0, 147.9, 145.7, 143.9, 138.4, 136.7, 133.4, 132.8, 131.23, 131.22, 130.24, 130.20, 128.7, 127.7, 127.50, 127.48, 127.4, 125.8, 124.7, 123.4, 121.4, 119.7, 114.8, 114.4, 112.5, 99.4, 31.3, 31.1 ppm. HRMS (EI): m/z calculated for: $C_{46}H_{32}N_4O$ [$M^{\cdot+}$] = 656.2576; found 656.2582; yield: 17% (0.13g)

Synthesis of compounds **55.1-4**

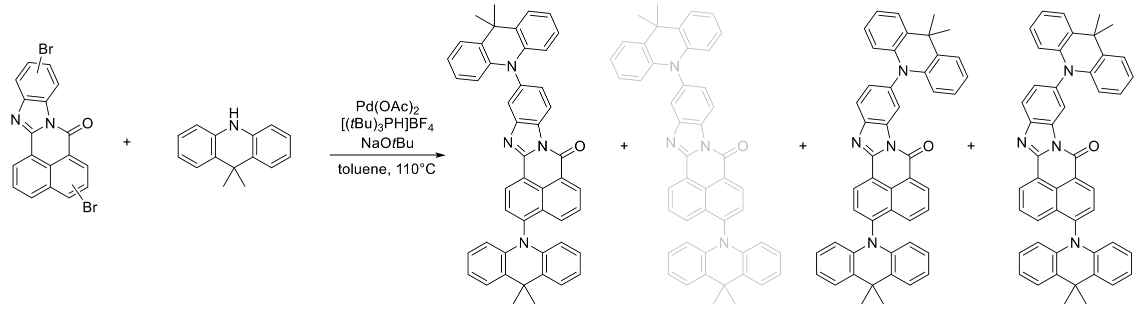

To the mixture of isomers dibromo substituted benzo[de]benzo[4,5]imidazo[2,1-a]isoquinolin-7-one (500 mg; 1.17 mmol), 9,9-dimethyl-10H-acridine (611 mg; 2.92 mmol; 2.5 equiv.), sodium tert-butoxide (281 mg; 2.92 mmol; 2.5 equiv.) 15 mL of degassed toluene were added. The mixture was degassed again by bubbling argon through the solution for 5 minutes. Tri-tert-butylphosphonium tetrafluoroborate (204 mg; 0.7 mmol, 60 mol%) and palladium acetate (53 mg; 0.234 mmol, 20 mol%) were then added. The reaction mixture was heated at 110°C for 16 hours. Water was added to the reaction mixture, followed by extraction with methylene chloride. The organic layer was dried over Na_2SO_4. The reaction yielded a mixture of regioisomers of the product, which were subsequently separated through column chromatography using a toluene and ethyl acetate mixture as the eluent.

*Compound **55.1***

^{1}H NMR ($CDCl_3$, 600 MHz): δ 9.12 (d, *J* = 7.6 Hz, 1H), 8.89 (dd, *J* = 7.2, 1.0 Hz, 1H), 8.85 (d, *J* = 8.4 Hz, 1H), 8.21 (dd, *J* = 8.5, 0.9 Hz, 1H), 7.98 (d, *J* = 1.7 Hz, 1H), 7.91 (d, *J* = 7.7 Hz, 1H), 7.76 (dd, *J* = 8.4, 7.3 Hz, 1H), 7.58 (dd, *J* = 7.8, 1.3 Hz, 2H), 7.54 – 7.49 (m, 3H), 7.03 – 6.96 (m, 6H), 6.95 – 6.91 (m, 2H), 6.43 (d, *J* = 8.1 Hz, 2H), 6.09 (d, *J* = 8.2 Hz, 2H), 1.91 (s, 3H), 1.81 (s, 3H), 1.77 (s, 6H) ppm. ^{13}C NMR ($CDCl_3$, 150 MHz): δ 160.5, 149.9, 146.0, 142.6, 141.3, 140.5, 139.2, 132.8, 131.8, 131.7, 131.6, 131.4, 130.3, 130.1, 129.5, 129.0, 128.9, 128.2, 126.9, 126.5, 126.1, 125.3, 124.2, 123.5, 121.4, 121.1, 120.8, 118.2, 114.3, 114.2, 36.20, 36.17, 33.0, 32.1, 31.3 ppm. HRMS (EI): m/z calculated for: $C_{48}H_{36}N_4O$ [$M^{\cdot+}$] = 684.2889; found: 684.2873; yield: 16% (0.13g).

*Compound **55.3***

^{1}H NMR ($CDCl_3$, 600 MHz): δ 9.14 (d, *J* = 7.6 Hz, 1H), 8.82 – 8.76 (m, 1H), 8.66 – 8.62 (m, 1H), 8.16 (dd, *J* = 8.4, 3.0 Hz, 2H), 7.90 (d, *J* = 7.7 Hz, 1H), 7.74 – 7.68 (m, 1H), 7.56 (dd, *J* = 7.8, 1.1 Hz, 2H), 7.50 (d, *J* = 8.4 Hz, 3H), 7.02 – 6.94 (m, 6H), 6.93 – 6.88 (m, 2H), 6.39 (d, *J* = 7.9 Hz, 2H), 6.05 (d, *J* = 8.2 Hz, 2H), 1.88 (s, 3H), 1.79 (s, 3H), 1.75 (s, 6H) ppm. ^{13}C NMR ($CDCl_3$, 150 MHz): δ 160.4, 150.2, 143.7, 142.6, 141.3, 140.5, 138.9, 133.7, 132.8, 131.7, 131.6, 131.3, 130.3, 130.1, 129.53, 129.47, 128.8, 128.3, 126.9, 126.5, 126.1, 125.3, 124.3, 122.3, 121.4, 121.2, 120.8, 119.2, 114.4, 114.3, 36.23, 36.19, 33.1, 32.2, 31.4 ppm. HRMS (EI): m/z calculated for: $C_{48}H_{36}N_4O$ [$M^{\cdot+}$] = 684.2889; found: 684.2873; yield: 15% (0.12g)

*Compound **55.4***

^{1}H NMR ($CDCl_3$, 600 MHz): δ 9.00 (d, *J* = 7.6 Hz, 1H), 8.96 (d, *J* = 7.2 Hz, 1H), 8.63 (d, *J* = 1.5 Hz, 1H), 8.15 (d, *J* = 8.4 Hz, 1H), 8.06 (d, *J* = 8.6 Hz, 1H), 7.88 (d, *J* = 7.7 Hz, 1H), 7.74 (t, *J* = 7.9 Hz, 1H), 7.56 (d, *J* = 7.8 Hz, 2H), 7.53 – 7.47 (m, 3H), 7.02 – 6.93 (m, 6H), 6.90 (t, *J* = 7.2 Hz, 2H), 6.39 (d, *J* = 8.0 Hz, 2H), 6.00 (d, *J* = 8.2 Hz, 2H), 1.88 (s, 3H), 1.80 (s, 3H), 1.77 (s, 6H) ppm. ^{13}C NMR ($CDCl_3$, 125 MHz): δ 160.1, 150.6, 145.8, 143.7, 141.33, 140.29, 138.8, 138.0, 133.7, 133.5, 131.7, 131.0, 130.3, 130.1, 129.6, 129.5, 129.2, 128.7, 128.5, 128.4, 128.2, 126.9, 126.5, 126.1, 125.43, 125.38, 123.5, 122.2, 121.8, 121.5, 120.8, 119.2,

114.4, 114.2, 36.22, 36.20, 32.9, 32.3, 31.5 ppm. HRMS (EI): m/z calculated for: $C_{48}H_{36}N_4O$ $[M^{\cdot+}]$ = 684.2889; found: 684.2873; yield: 13% (0.1g)

Synthesis of compounds **56.1-4**

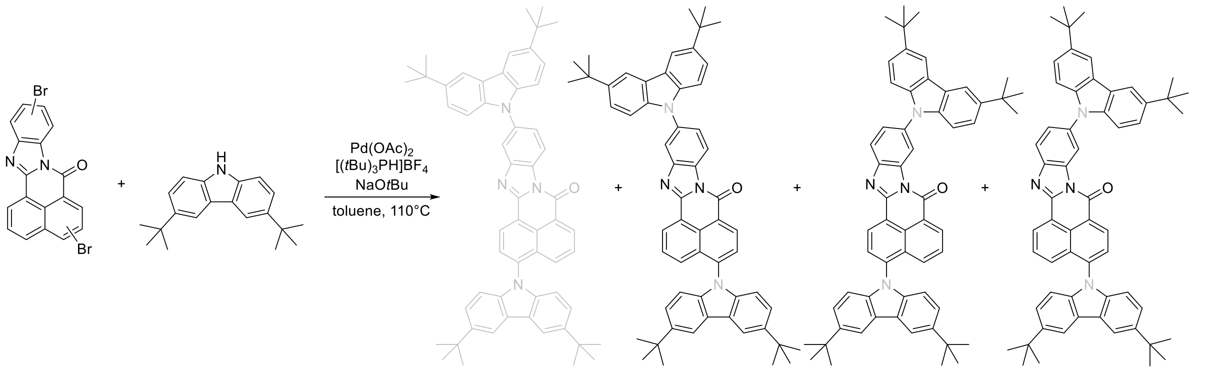

56.2 56.3 56.4

To the mixture of isomers dibromosubstituted benzo[de]benzo[4,5]imidazo[2,1-a]isoquinolin-7-one (500 mg; 1.17 mmol), 3,6-di-tert-butylcarbazole (816 mg; 2.92 mmol; 2.5 equiv.), sodium tert-butoxide (281 mg; 2.92 mmol; 2.5 equiv.) 15 mL of degassed toluene were added. The mixture was degassed again by bubbling argon through the solution for 5 minutes. Tri-tert-butylphosphonium tetrafluoroborate (204 mg; 0.7 mmol, 60 mol%) and palladium acetate (53 mg; 0.234 mmol, 20 mol%) were then added. The reaction mixture was heated at 110°C for 16 hours. Water was added to the reaction mixture, followed by extraction with methylene chloride. The organic layer was dried over Na_2SO_4. The reaction yielded a mixture of regioisomers of the product, which were subsequently separated through column chromatography using a toluene and ethyl acetate mixture as the eluent

Compound ***56.2***
^{1}H NMR ($CDCl_3$, 600 MHz): δ 9.01 (d, *J* = 7.7 Hz, 1H), 8.96 (dd, *J* = 7.2, 0.9 Hz, 1H), 8.79 (d, *J* = 8.5 Hz, 1H), 8.24 (d, *J* = 1.7 Hz, 2H), 8.20 (d, *J* = 1.4 Hz, 2H), 8.13 (d, *J* = 1.7 Hz, 1H), 7.99 (d, *J* = 7.7 Hz, 1H), 7.95 (dd, *J* = 8.5, 0.9 Hz, 1H), 7.71 (dd, *J* = 8.4, 1.9 Hz, 2H), 7.52 – 7.44 (m, 6H), 7.07 (d, *J* = 8.6 Hz, 2H), 1.50 (s, 36H) ppm. ^{13}C NMR ($CDCl_3$, 150 MHz): δ 160.2, 150.6, 145.2, 144.1, 143.1, 142.7, 140.4, 139.6, 136.3, 132.5, 130.8, 129.8, 129.02, 128.99, 128.3, 128.0, 127.1, 124.6, 124.23, 124.17, 123.9, 123.6, 122.7, 121.4, 118.4, 117.0, 116.7, 116.4, 109.8, 109.4, 35.0, 34.9, 32.2, 32.1 ppm. HRMS (EI): m/z calculated for: $C_{60}H_{58}N_2O$ $[M^{\cdot+}]$ = 824.4454; found: 824.4437; yield: 16% (0.18g)

Compound ***56.3***
^{1}H NMR ($CDCl_3$, 500 MHz): δ 9.06 (d, *J* = 7.6 Hz, 1H), 8.84 (s, 2H), 8.23 (d, *J* = 1.3 Hz, 2H), 8.19 (s, 2H), 8.10 (d, *J* = 8.4 Hz, 1H), 8.02 (d, *J* = 8.4 Hz, 1H), 7.97 (d, *J* = 7.7 Hz, 1H), 7.75 – 7.69 (m, 2H), 7.51 (s, 4H), 7.45 (dd, *J* = 8.6, 1.6 Hz, 2H), 7.03 (d, *J* = 8.6 Hz, 2H), 1.495 (s, 18H), 1.501 (s, 18H) ppm. ^{13}C NMR ($CDCl_3$, 125 MHz): δ 160.6, 149.9, 143.9, 143.2, 142.8, 140.7, 140.6, 139.7, 139.3, 136.0, 132.9, 132.7, 132.0, 130.1, 128.9, 127.8, 127.7, 127.6, 125.1, 124.2, 124.0, 123.9, 123.7, 121.1, 120.6, 116.7, 116.4, 114.5, 109.7, 109.5, 35.0, 34.9, 32.19, 32.16 ppm. HRMS (EI): m/z calculated for: $C_{60}H_{58}N_2O$ $[M^{\cdot+}]$ = 824.4454; found: 824.4437; yield: 15% (0.17g)

Compound ***56.4***
^{1}H NMR ($CDCl_3$, 600 MHz): δ 8.97 (d, *J* = 7.2 Hz, 1H), 8.94 (d, *J* = 7.7 Hz, 1H), 8.85 (d, *J* = 1.8 Hz, 1H), 8.23 (d, *J* = 1.8 Hz, 2H), 8.19 (d, *J* = 0.7 Hz, 2H), 8.09 (d, *J* = 8.4 Hz, 1H), 7.96

– 7.92 (m, 2H), 7.75 – 7.71 (m, 2H), 7.53 – 7.48 (m, 4H), 7.45 (dd, *J* = 8.6, 1.9 Hz, 2H), 7.04 (d, *J* = 8.6 Hz, 2H), 1.49 (s, 18H), 1.50 (s, 18H) ppm. ^{13}C NMR ($CDCl_3$, 150 MHz): δ 160.2, 150.4, 144.1, 143.2, 142.8, 142.6, 140.4, 139.7, 135.9, 132.9, 132.4, 129.8, 129.1, 128.8, 128.0, 127.1, 125.1, 124.2, 124.1, 123.9, 123.7, 122.7, 121.5, 121.1, 119.2, 116.7, 116.4, 114.5, 109.8, 109.5, 35.0, 34.9, 32.2, 32.1 ppm. HRMS (EI): m/z calculated for: $C_{60}H_{58}N_2O$ [$M^{\cdot+}$] = 824.4454; found: 824.4437; yield: 14% (0.16g)

SI-8.2 Copies of NMR spectra and 2D NMR signal assignments

^{1}H NMR spectrum of compound **53.1**

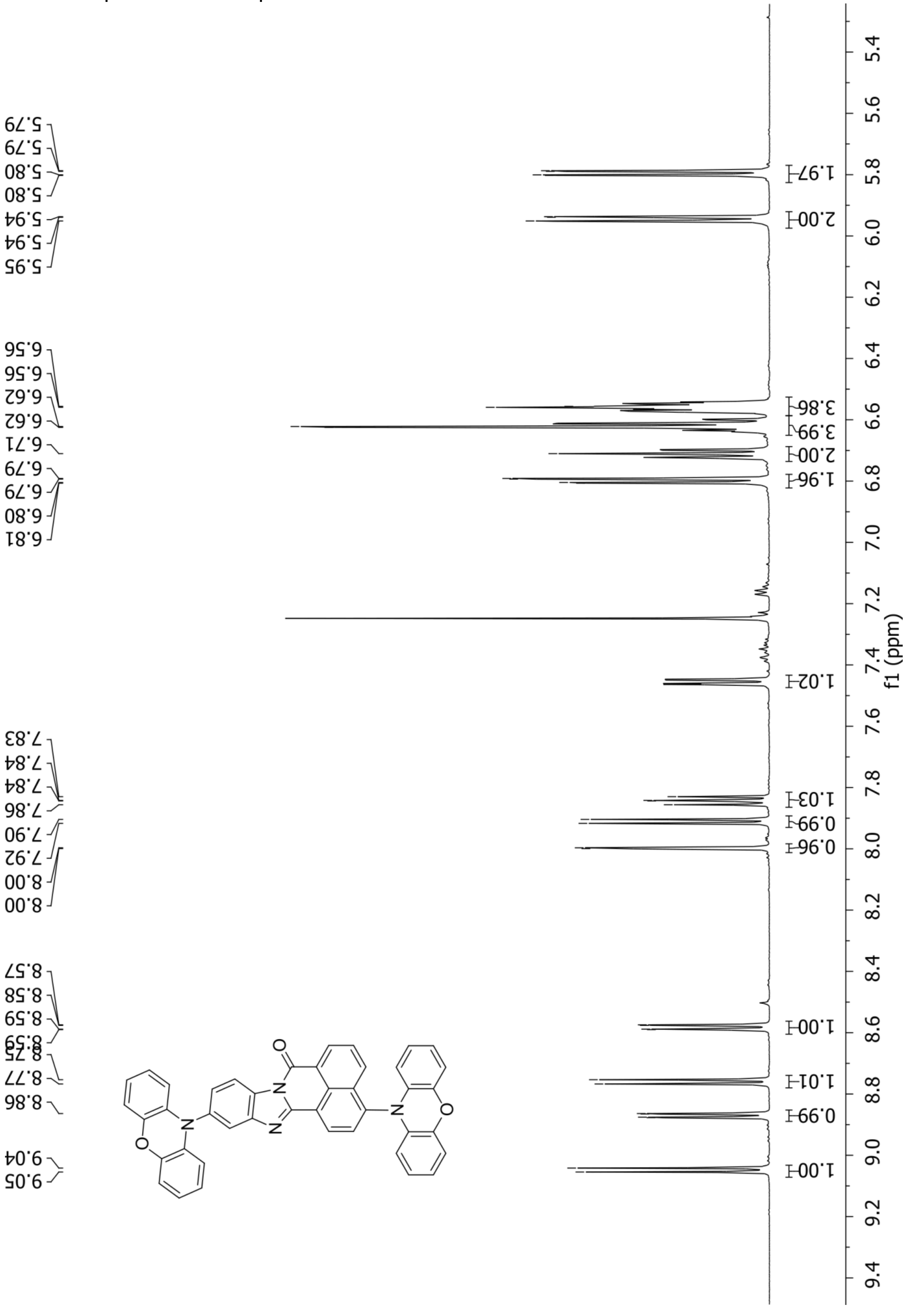

^{13}C NMR spectrum of compound **53.1**

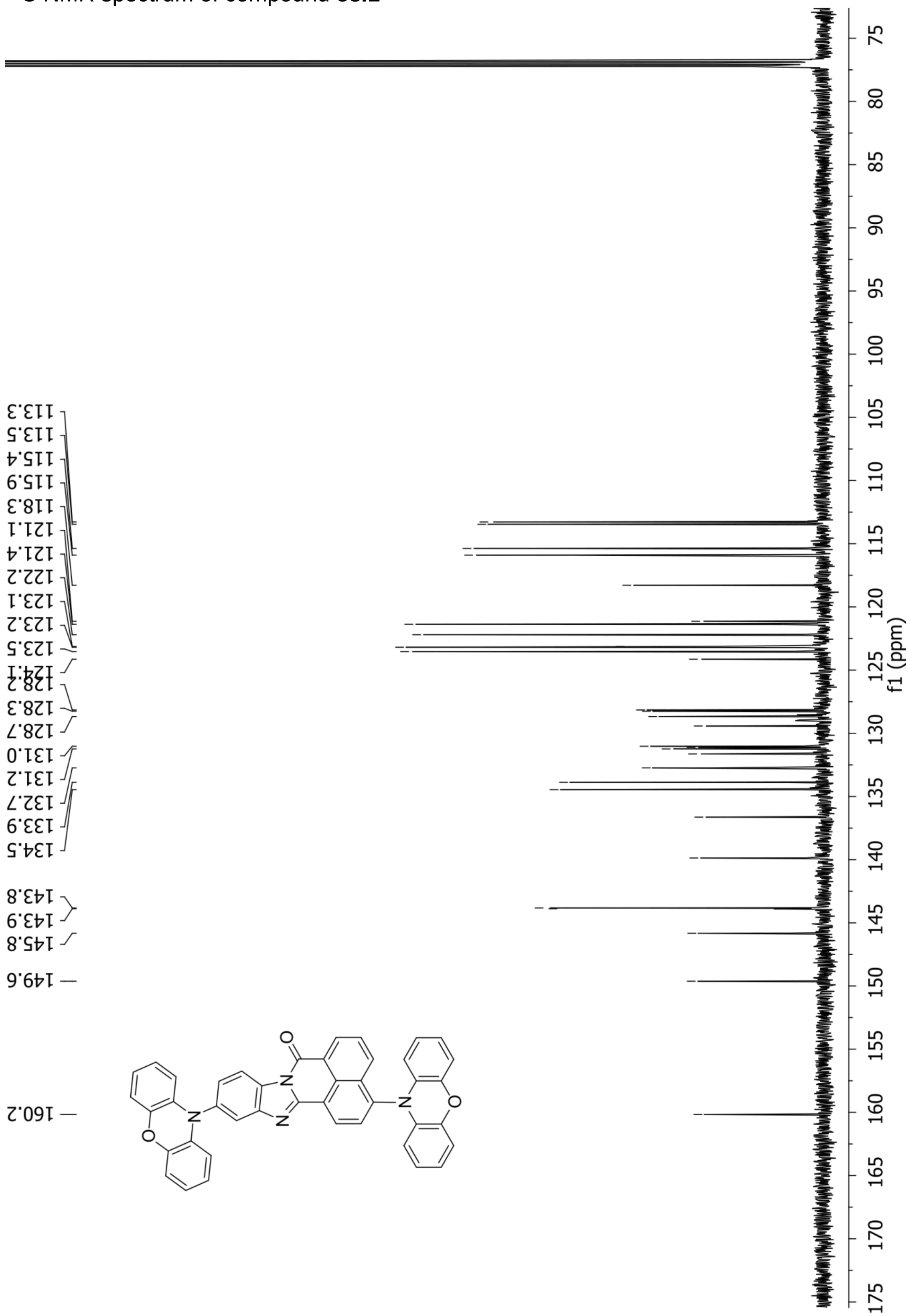

^{1}H-^{1}H COSY NMR spectrum of compound **53.1**

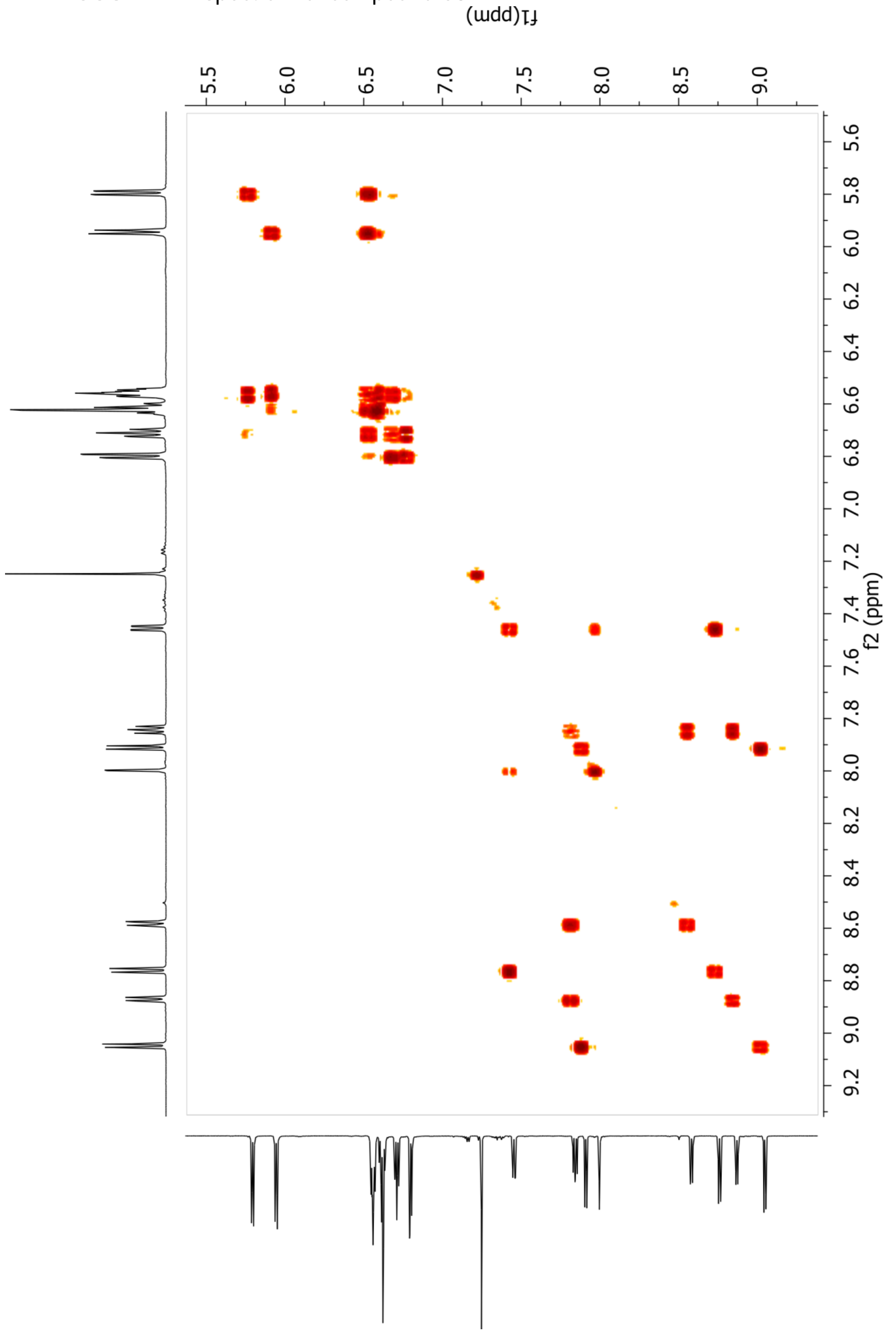

^{1}H-^{13}C HSQC NMR spectrum of compound **53.1**

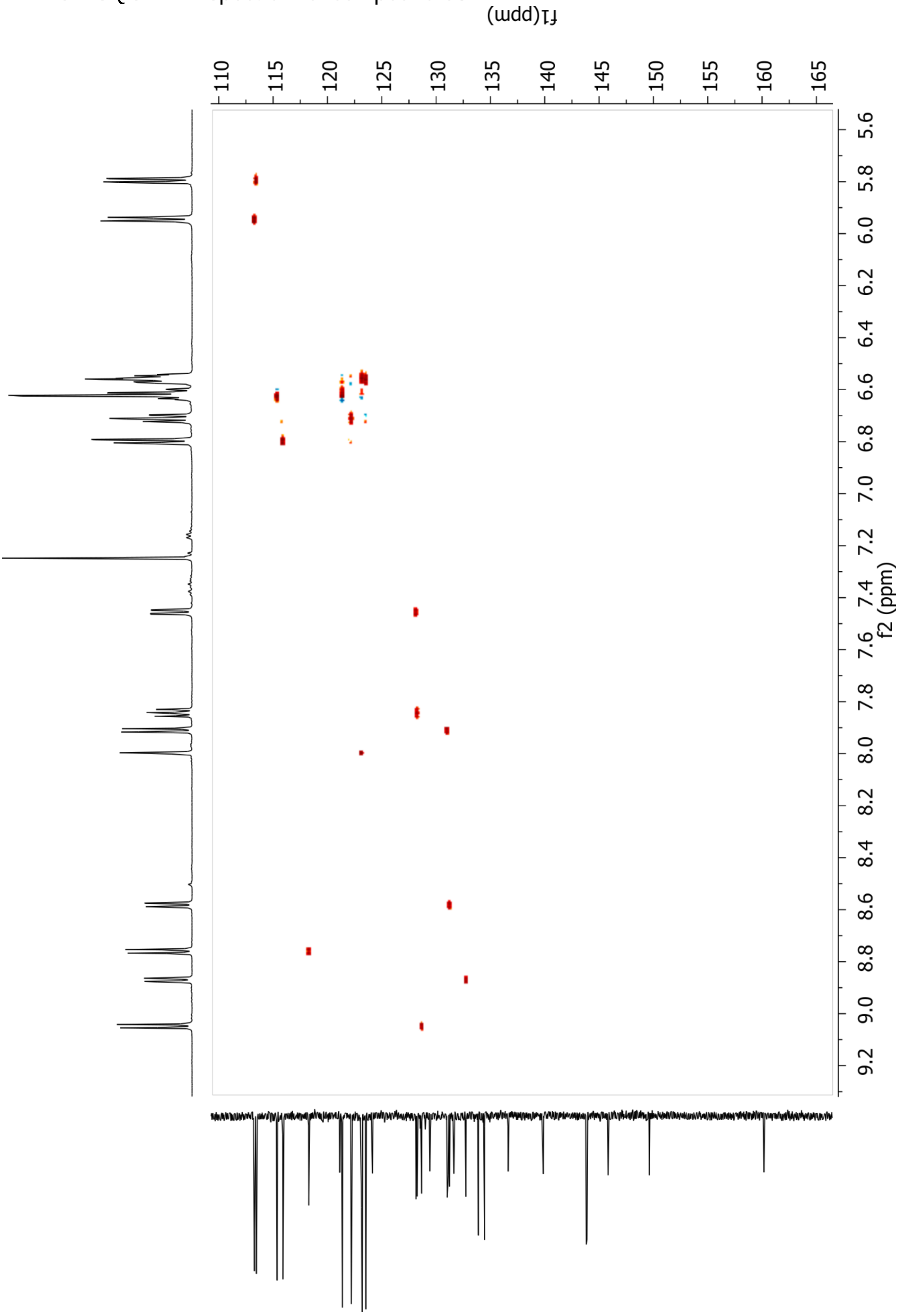

[1] H-^{13}C HMBC NMR spectrum of compound **53.1**

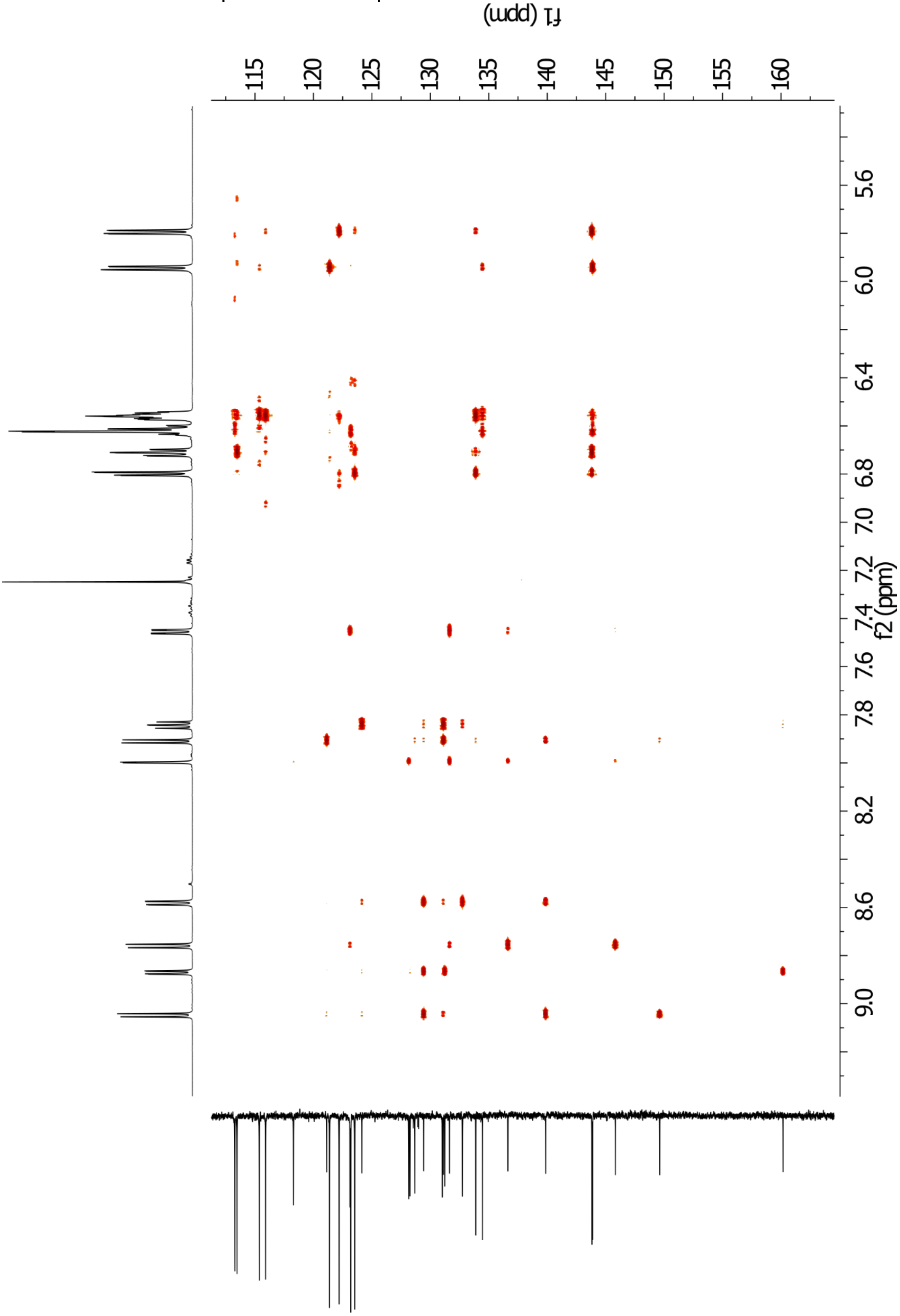

2D NMR assignments of **53.1**

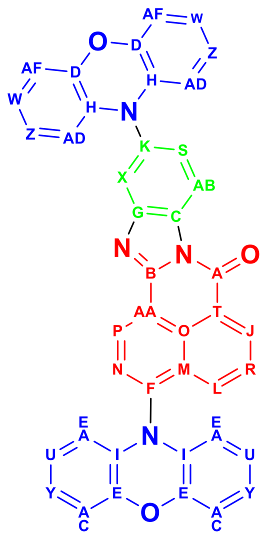

Nr	Symbol	^{13}C [ppm]	HSQC [ppm]	HMBC	COSY
**1**	A	160.34		J, R	
**2**	B	149.79		P, N	
**3**	C	145.99		AB, X, S	
**4**	D	144.05		AF, Z, AD	
**5**	E	143.99		AE, AC, Y	
**6**	F	140.03		P, R, N, L	
**7**	G	136.80		AB, X, S	
**8**	H	134.62		AF, AD, Z, W	
**9**	I	134.05		AE, AC, Y, U, N	
**10**	J	132.91	8.88	R, L, P	R
**11**	K	131.80		AB, X, S	
**12**	L	131.40	8.59		R
**13**	M	131.28		P, L, N, R	
**14**	N	131.19	7.92		P
**15**	O	129.59		P, R, N, J, L	
**16**	P	128.84	9.06	N	N
**17**	R	128.42	7.85	J	L, J
**18**	S	128.32	7.47	X	X, AB
**19**	T	124.31		P, J, L, R	
**20**	U	123.70	6.60 – 6.54	AE, AC, Y	AE, Y
**21**	W	123.36	6.60 – 6.54	AF, AD	AF
**22**	X	123.29	8.01	S, AB	S
**23**	Y	122.36	6.74 – 6.70	AE, U, AC	AC, U
**24**	Z	121.54	6.66 – 6.60	AF	W
**25**	AA	121.30		N, P, J, R, L	
**26**	AB	118.46	8.77	X	S
**27**	AC	116.07	6.81	AE, U	Y
**28**	AD	115.54	6.66 – 6.60	AF, W	Z
**29**	AE	113.63	5.81	U, Y	U, Y
**30**	AF	113.44	5.98 – 5.93	W, Z/AD	W, Z

^{1}H NMR spectrum of compound **53.2**

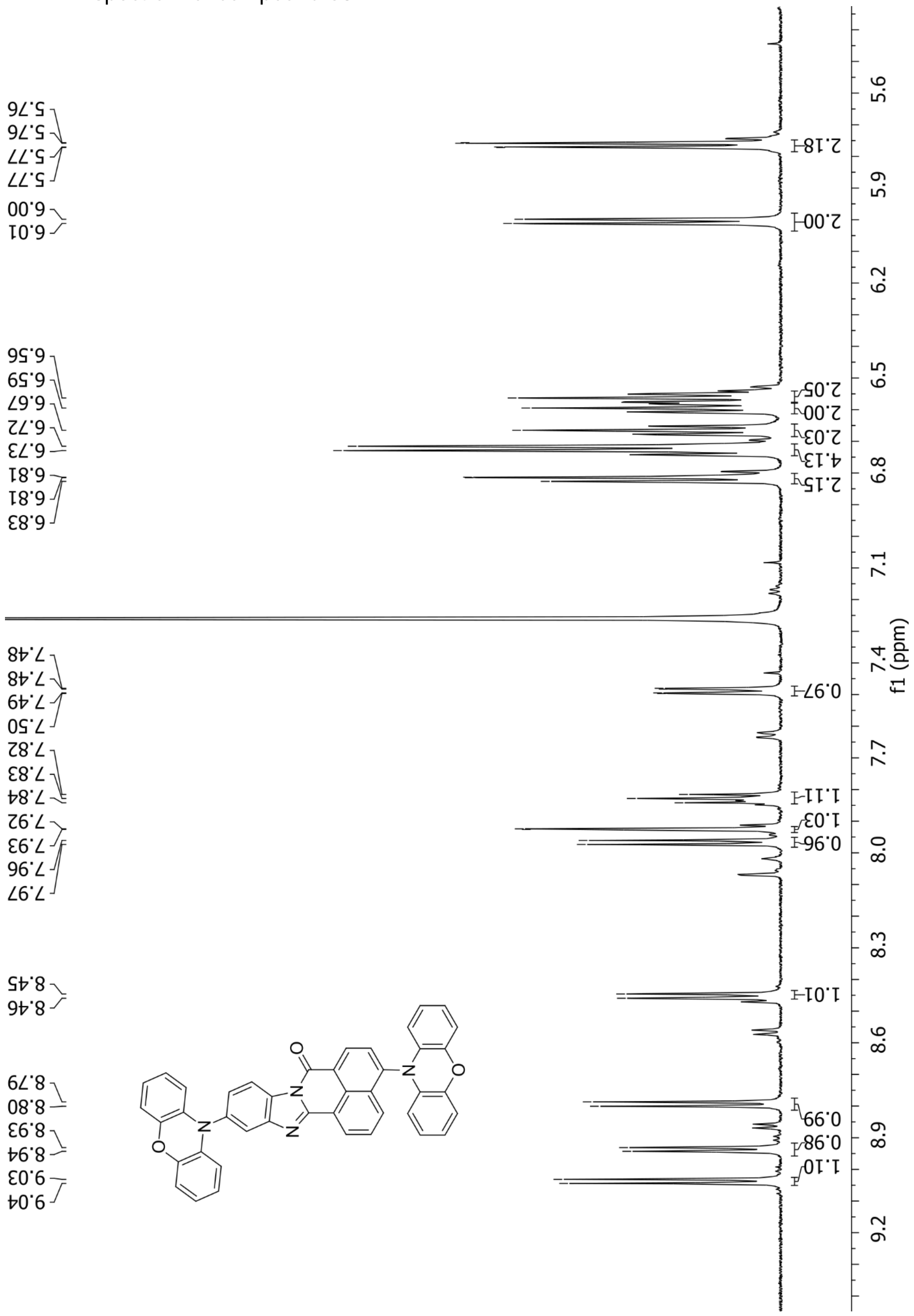

^{13}C NMR spectrum of compound **53.2**

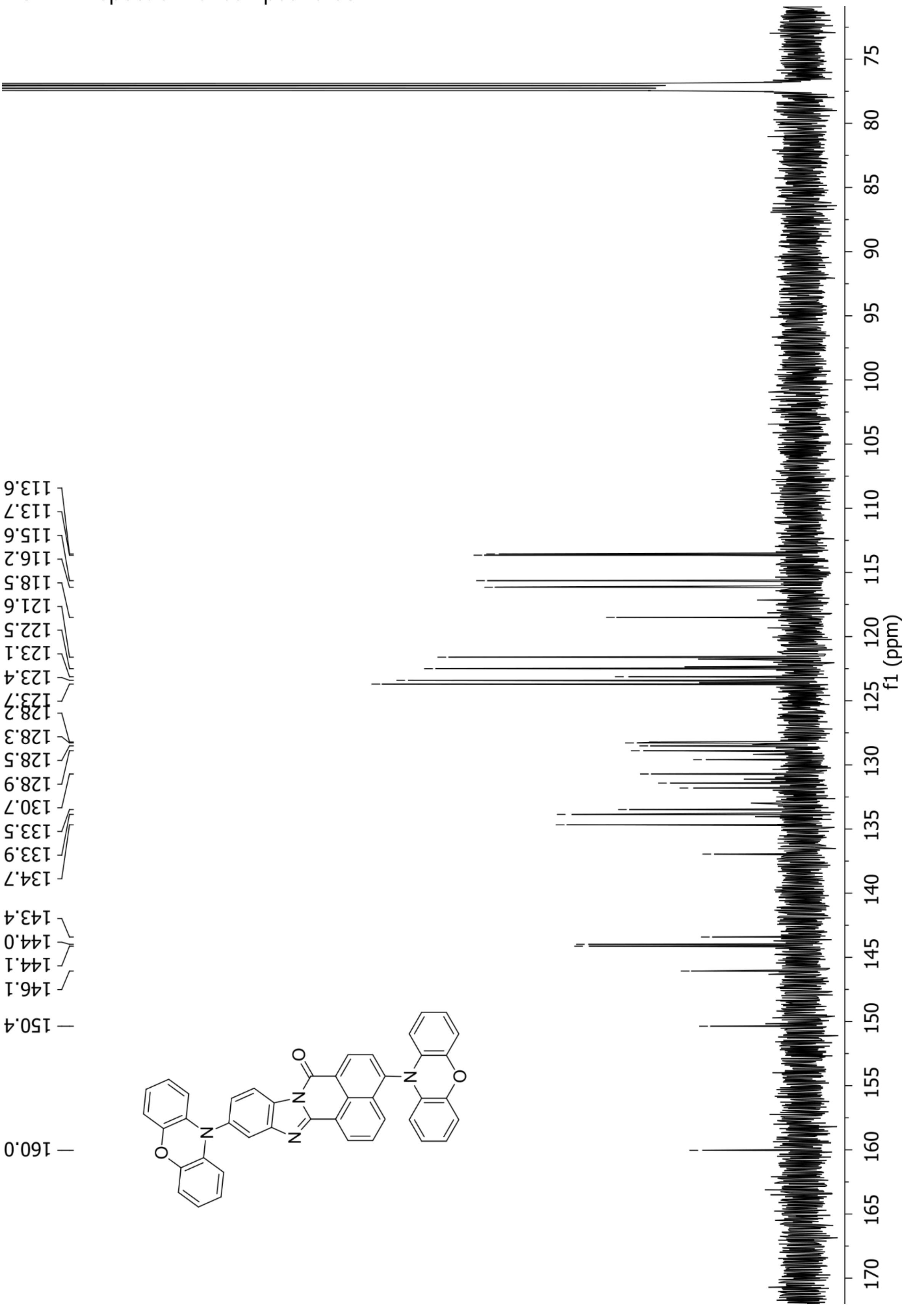

^{1}H-^{1}H COSY NMR spectrum of compound **53.2**

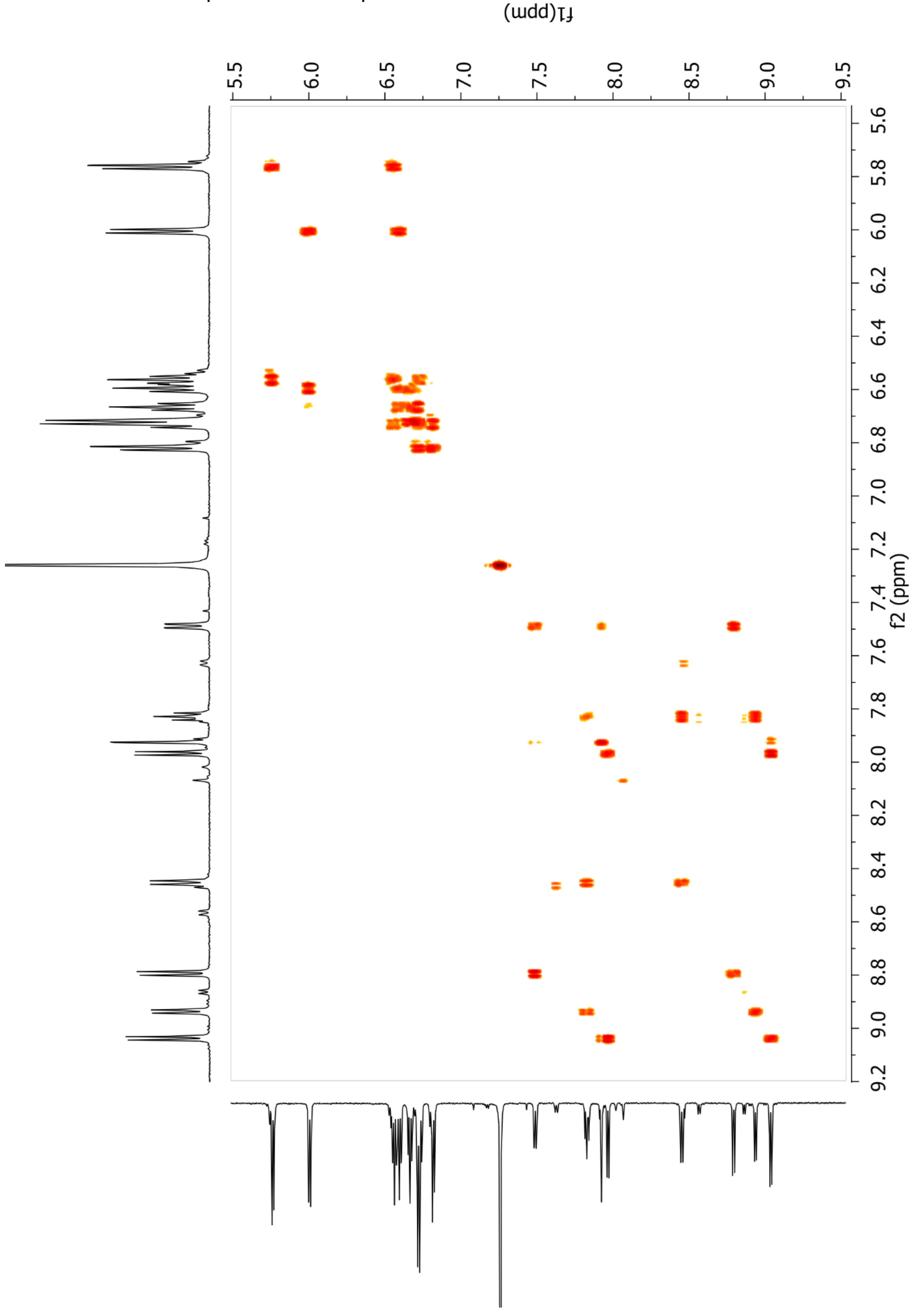

^{1}H-^{13}C HSQC NMR spectrum of compound **53.2**

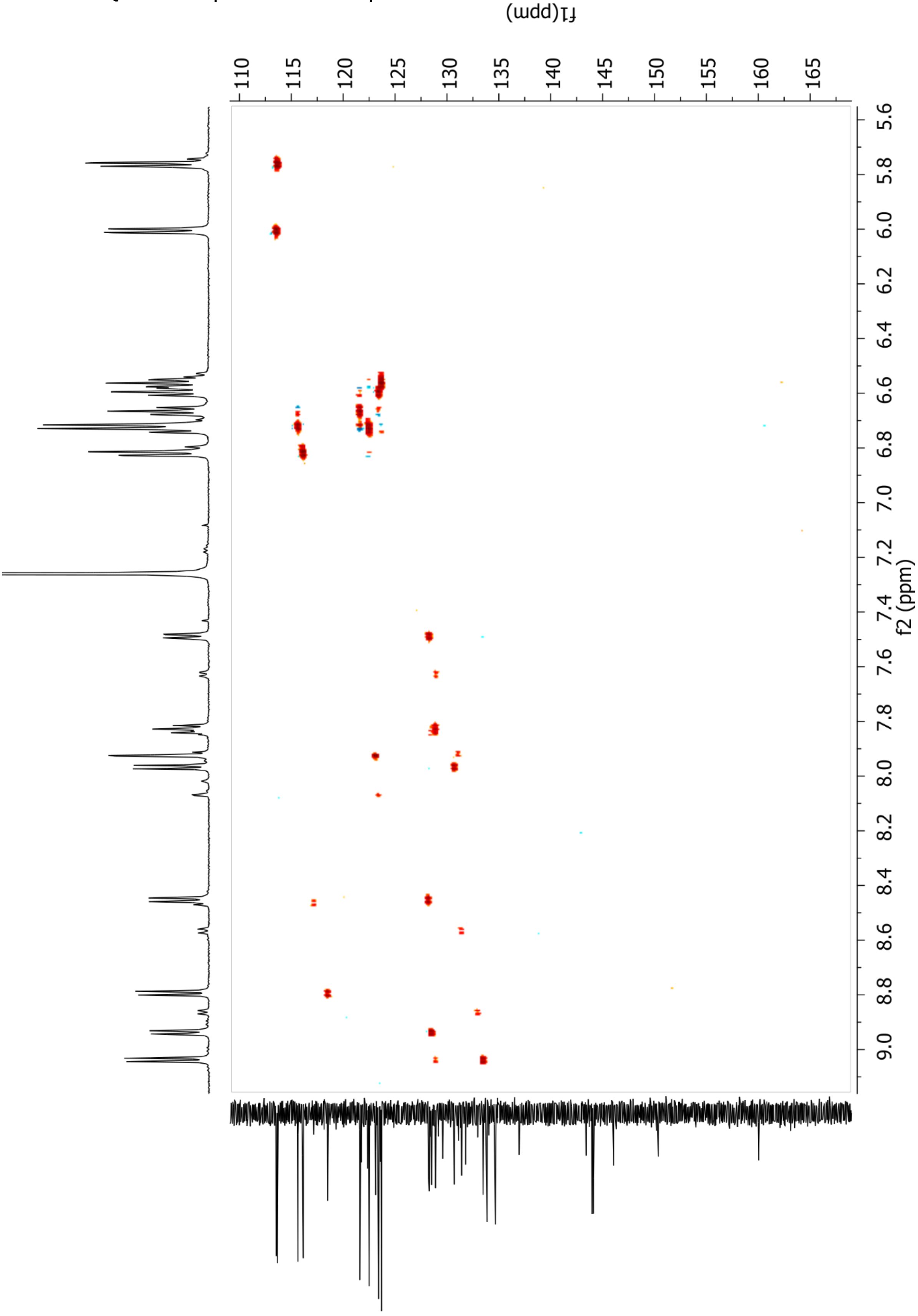

^{1}H-^{13}C HMBC NMR spectrum of compound **53.2**

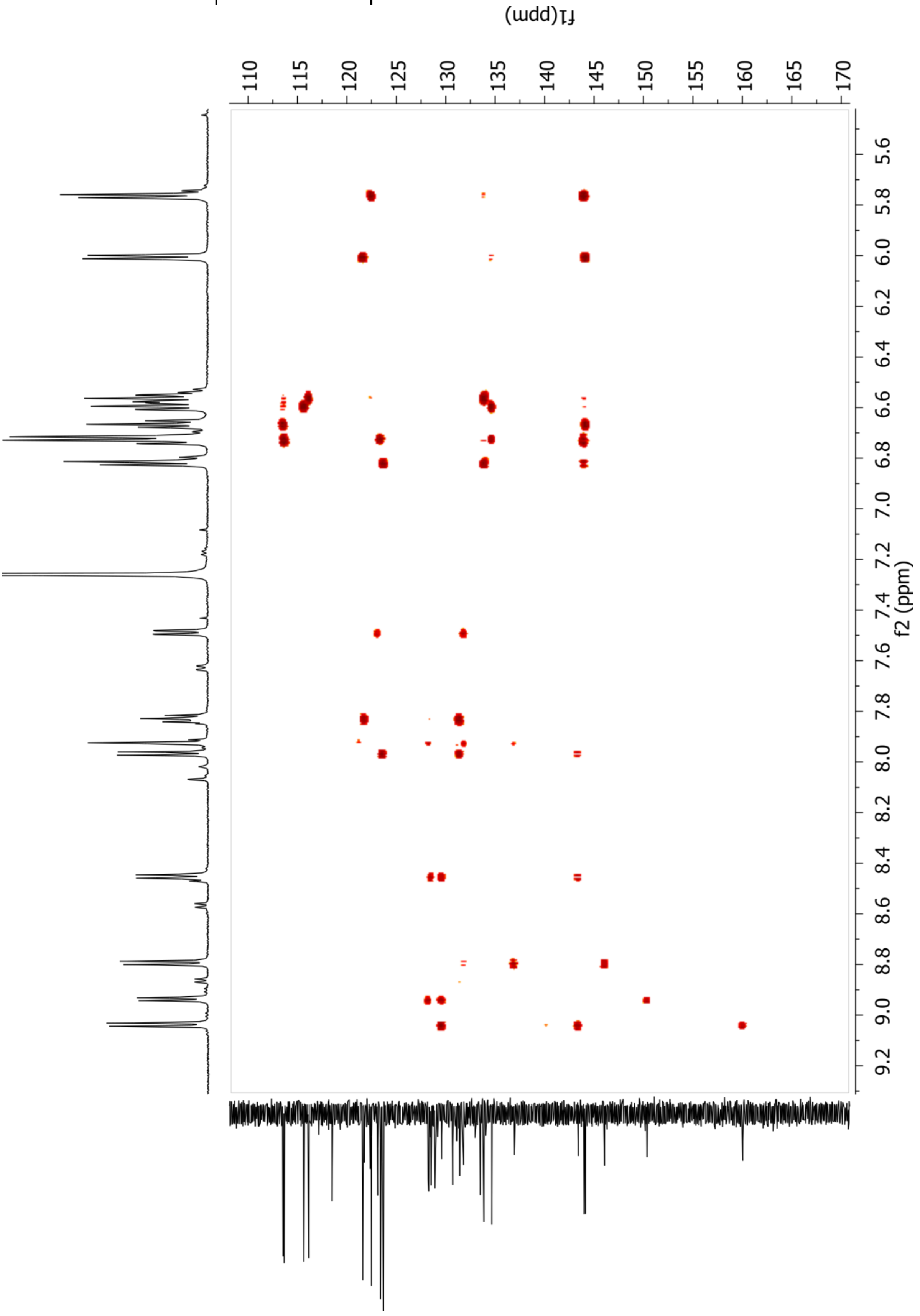

2D NMR assignments of **53.2**

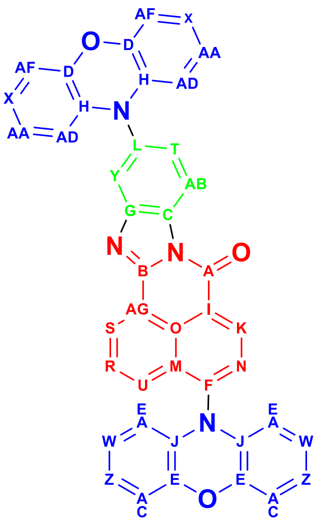

Nr	Symbol	^{13}C [ppm]	HSQC [ppm]	HMBC	COSY
**1**	A	160.04		K	
**2**	B	150.36		S	
**3**	C	146.07		AB	
**4**	D	144.13		AA, X, AF	
**5**	E	143.98		AC, Z, AE	
**6**	F	143.41		K, U, N	
**7**	G	136.95		AB, T, Y	
**8**	H	134.67		AD, X, AF	
**9**	J	133.86		AC, W, AE	
**10**	K	133.48	9.04		N
**11**	L	131.80		T, Y, AB	
**12**	M	131.42		R, N	
**13**	N	130.71	7.97		K
**14**	O	129.59		K, S, U	
**15**	R	128.90	7.83		U, S
**16**	S	128.52	8.94	R, U	R
**17**	T	128.29	7.49	Y	AB, Y
**18**	U	128.22	8.45	S	R
**19**	W	123.70	6.56	AC	AE, Z
**20**	I	123.59		N	
**21**	X	123.41	6.59	AD	AF, AA
**22**	Y	123.13	7.93	T	T
**23**	Z	122.50	6.75 – 6.70	AE	W, AC
**24**	AG	121.76		R	
**25**	AA	121.60	6.69 – 6.65	AF	X, AD
**26**	AB	118.50	8.79		T
**27**	AC	116.15	6.82	W	Z
**28**	AD	115.64	6.75 – 6.70	X	AA
**29**	AE	113.65	5.76	W, Z	W
**30**	AF	113.55.	6.01	X, AA	X

^{1}H NMR spectrum of compound **53.3**

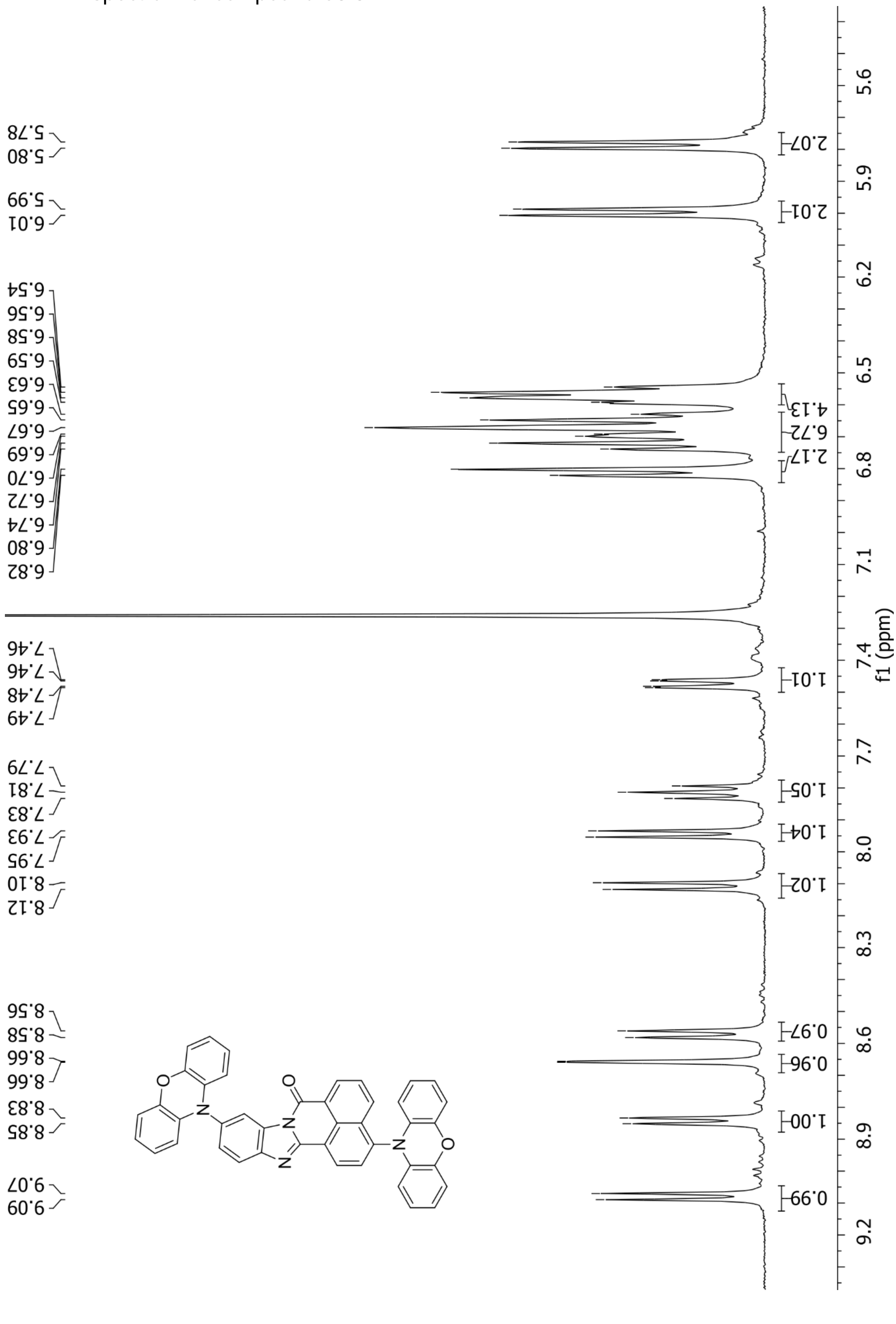

^{1}H NMR spectrum of compound **53.4**

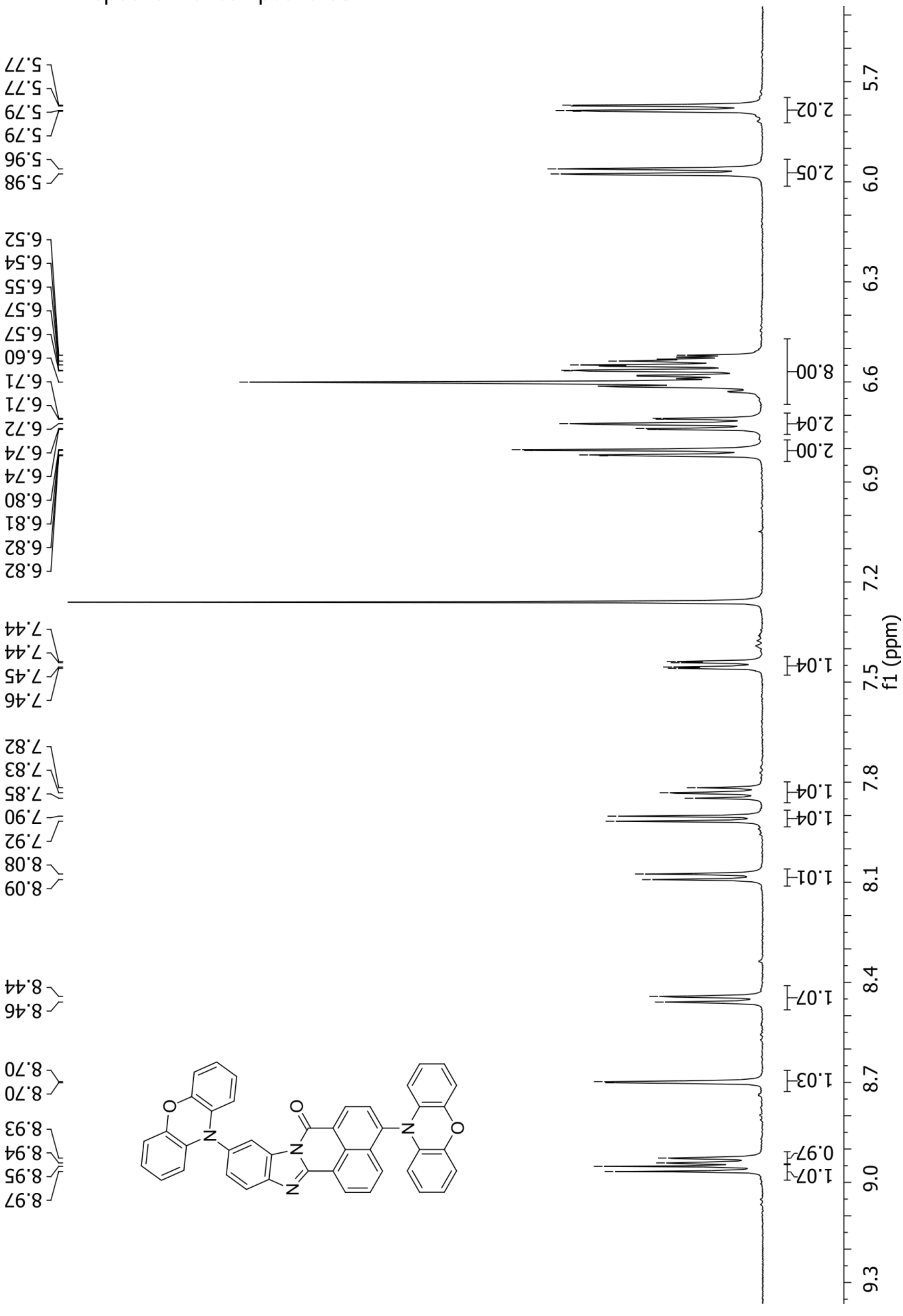

^{13}C NMR spectrum of compound **53.4**

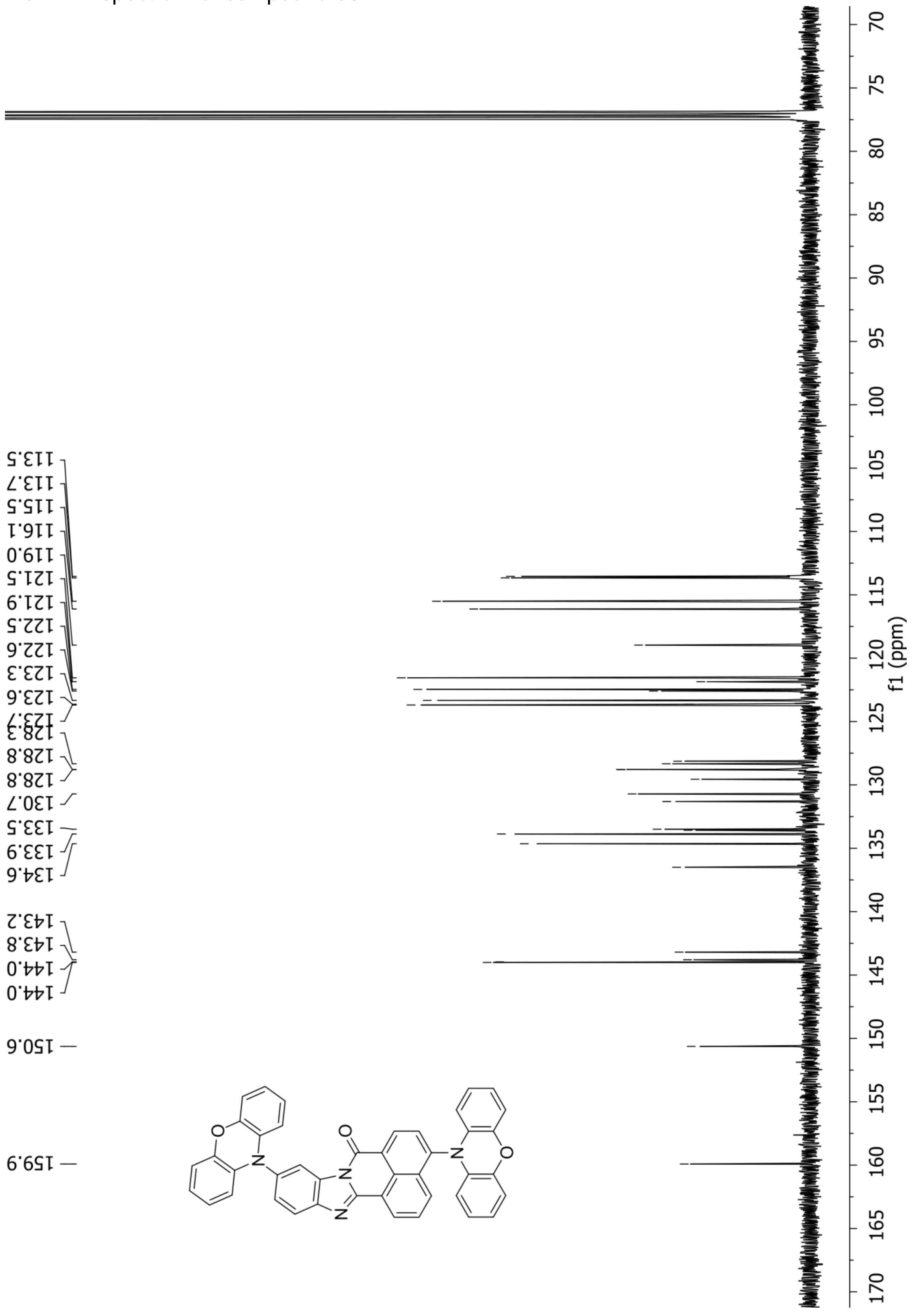

^{1}H-^{1}H COSY NMR spectrum of compound **53.4**

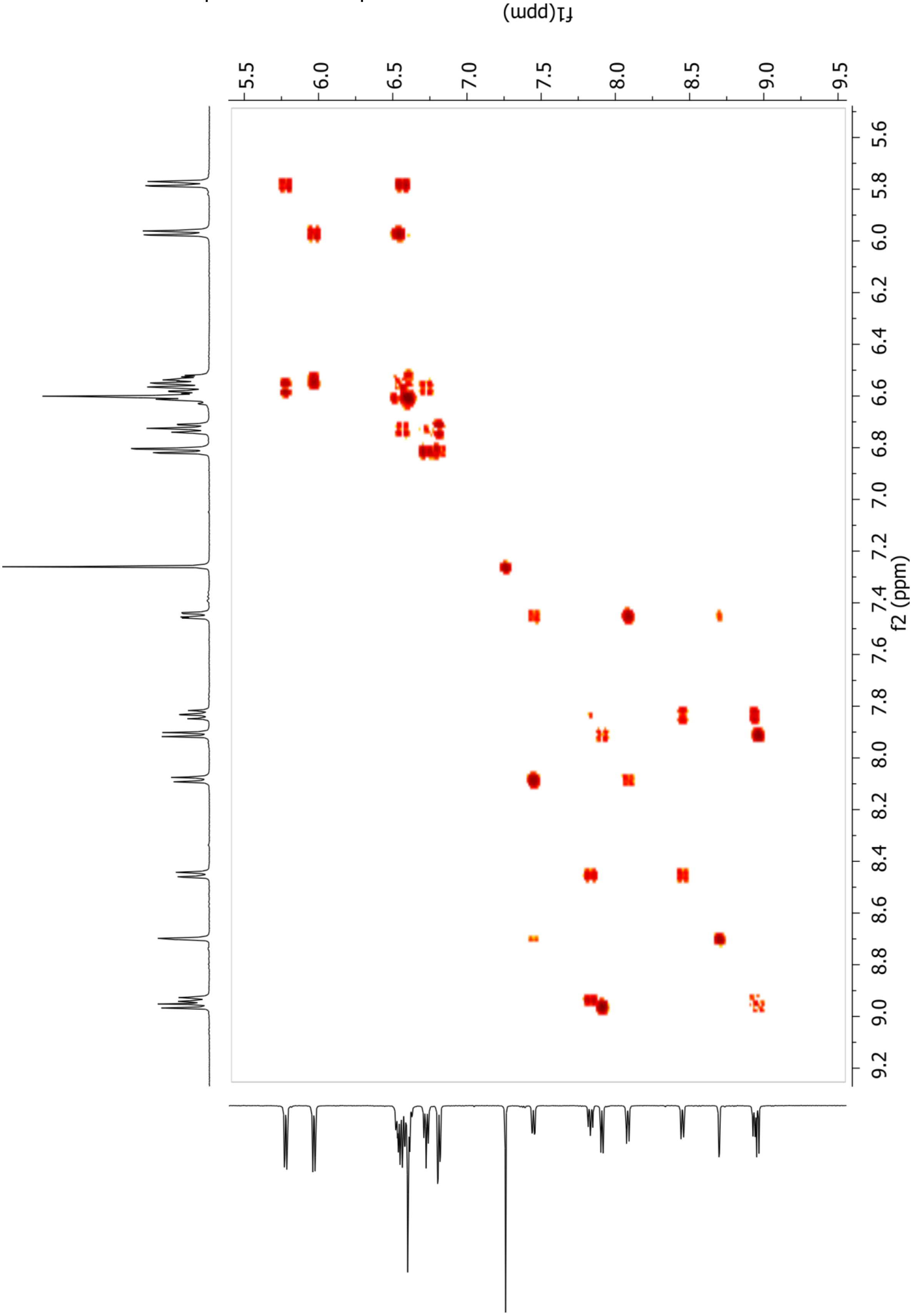

^{1}H-^{13}C HSQC NMR spectrum of compound **53.4**

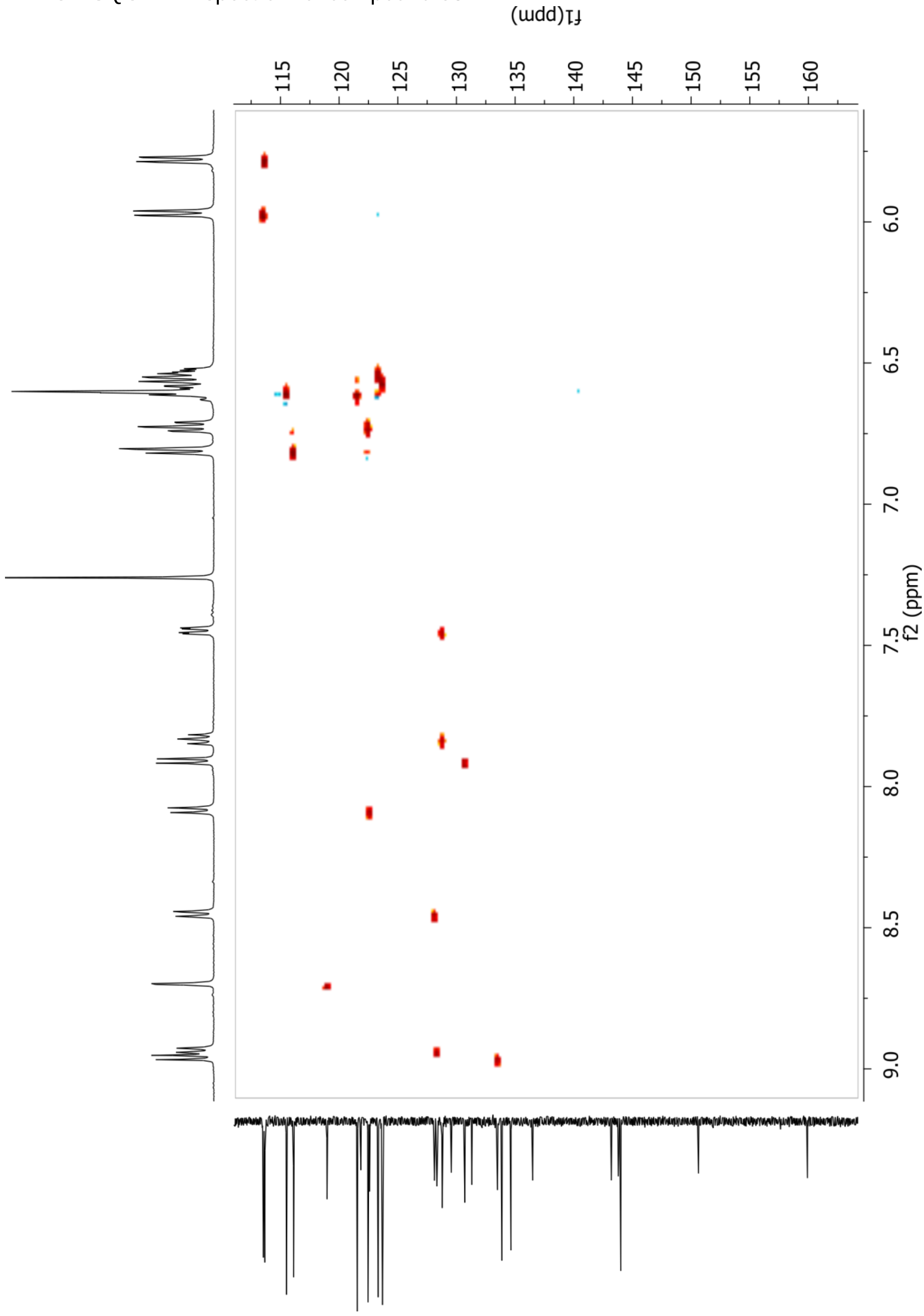

^{1}H-^{13}C HMBC NMR spectrum of compound **53.4**

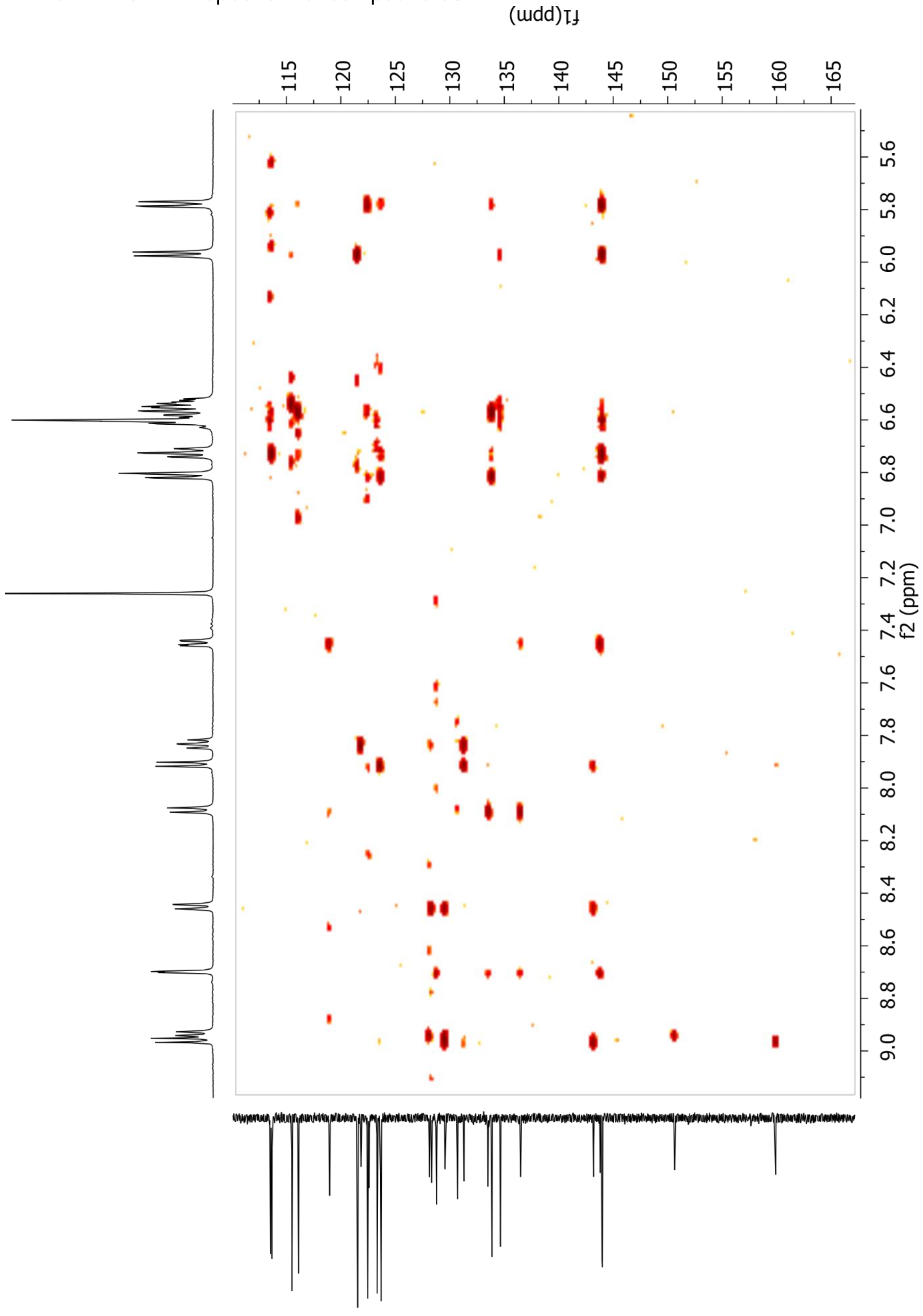

2D NMR assignments of **53.4**

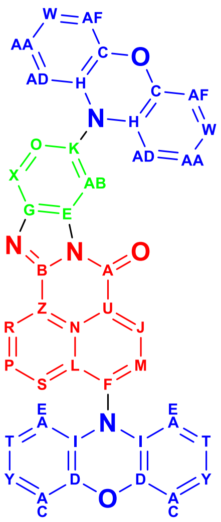

Isomer 4					
**Nr**	**Symbol**	**^{13}C [ppm]**	**HSQC [ppm]**	**HMBC**	**COSY**
**1**	A	159.91		J	
**2**	B	150.63		R	
**3**	**C**	144.00		AF, AA, AD	
**4**	D	143.96		AE, AC, Y	
**5**	E	143.79		AB, O	
**6**	F	143.20		J, S, M	
**7**	G	136.49		O, X, AB	
**8**	**H**	134.64		W, AA, AD, AF	
**9**	I	133.87		AE, T, Y, AC	
**10**	J	133.57	8.96		M
**11**	K	133.50		AB, X	
**12**	L	131.31		M, P	
**13**	M	130.71	7.91		J
**14**	N	129.56		J, S	
**15**	O	128.80	7.45	AB	X, AB
**16**	P	128.78	7.83		S, R
**17**	R	128.33	8.93	S	P
**18**	S	128.12	8.45	R	P
**19**	T	123.69	6.67 – 6.47	AE, AC, Y	AE, Y
**20**	U	123.63		M	
**21**	**W**	123.33	6.67 – 6.47	AD	AF, AA
**22**	X	122.59	8.08		O
**23**	Y	122.46	6.73	AE, T, AC	AC, T
**24**	Z	121.85		P	
**25**	**AA**	121.55	6.67 – 6.47	AF	W
**26**	AB	118.97	8.70	O	O
**27**	AC	116.12	6.81	T	Y
**28**	**AD**	115.51	6.67 – 6.47	W	
**29**	AE	113.67	5.78	T, Y	T
**30**	**AF**	113.54	5.97	W	W

^{1}H NMR spectrum of compound **54.1**

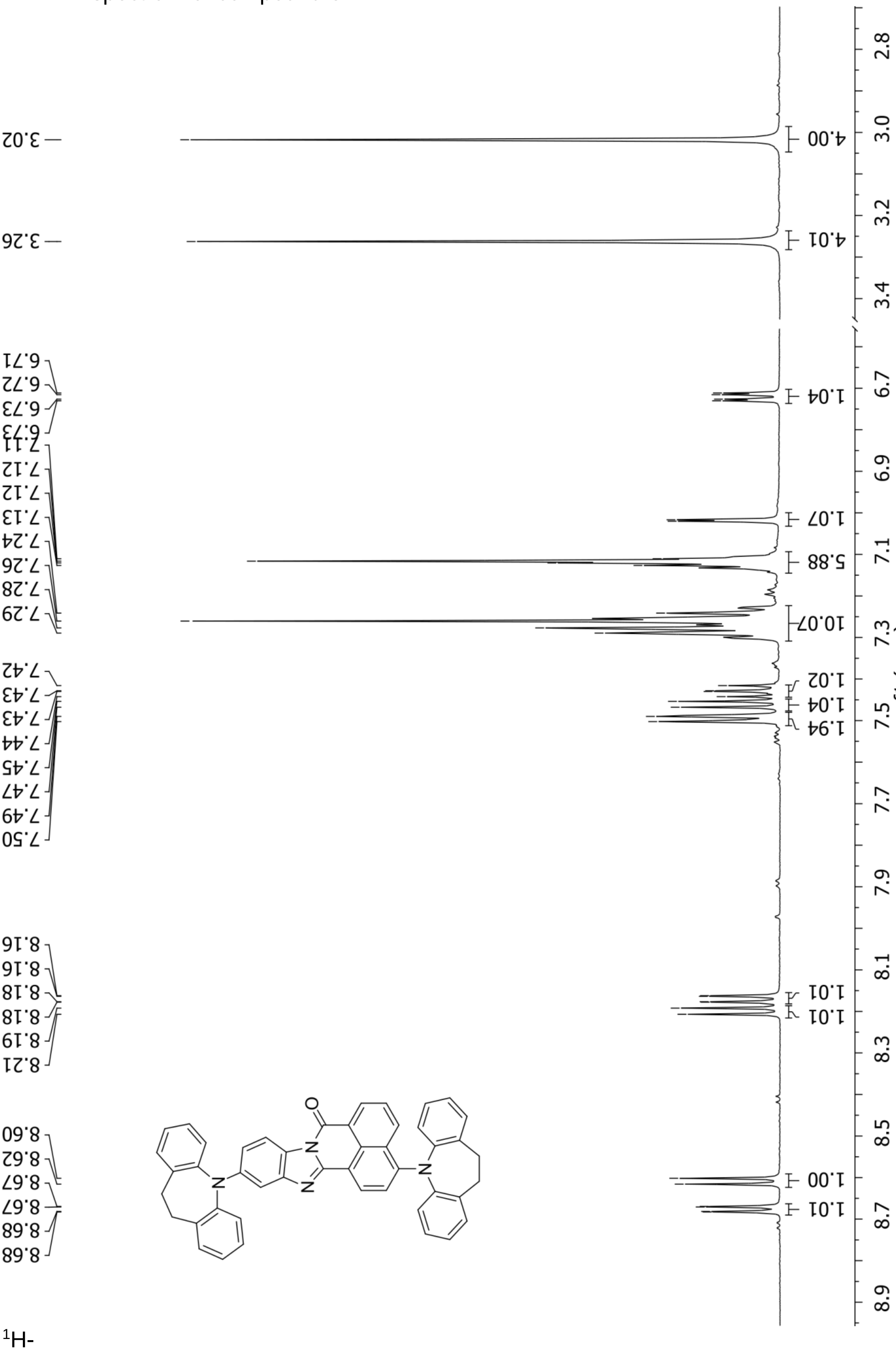

[1]H-

^{13}C NMR spectrum of compound **54.1**

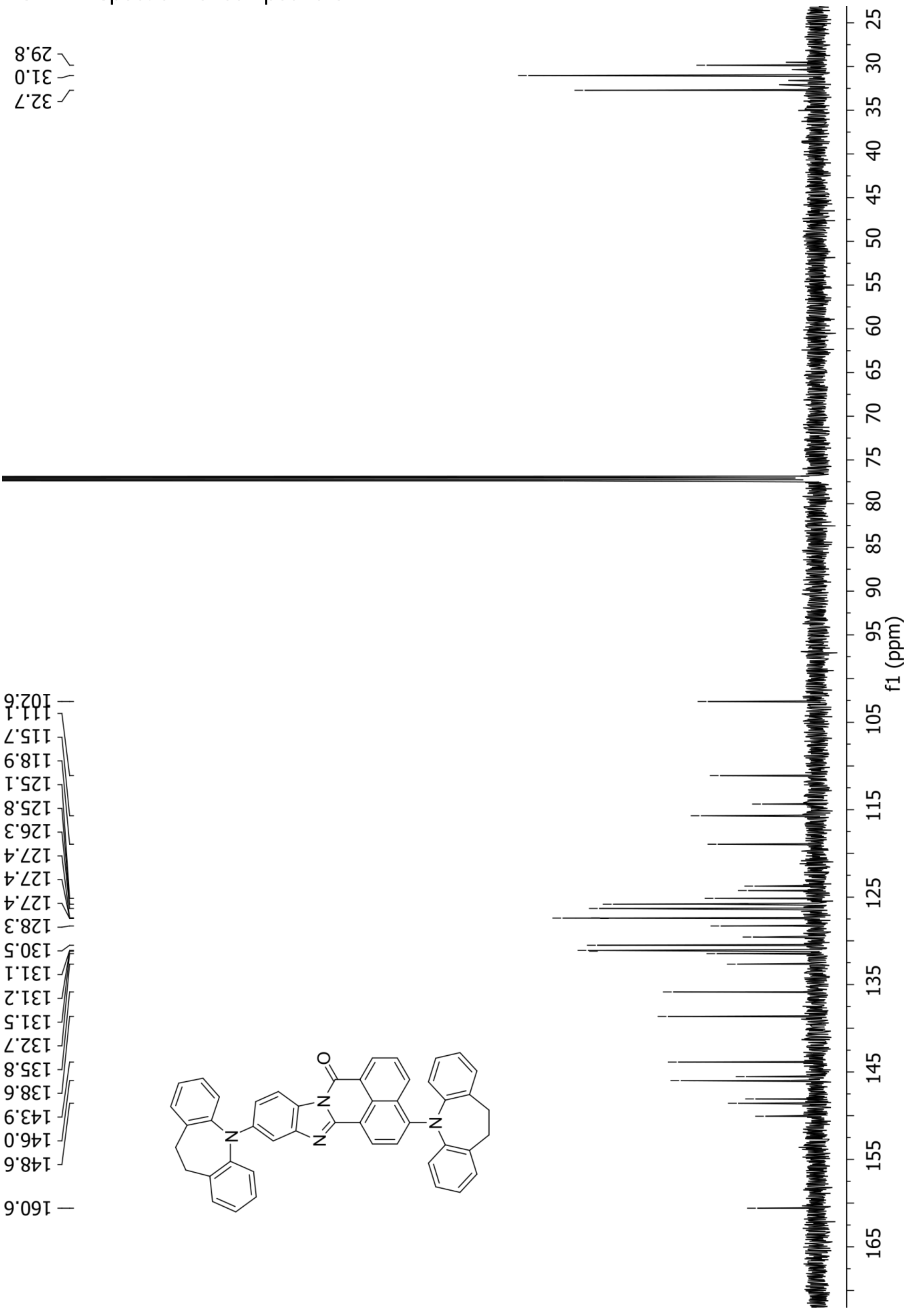

^1H-^1H COSY NMR spectrum of compound **54.1**

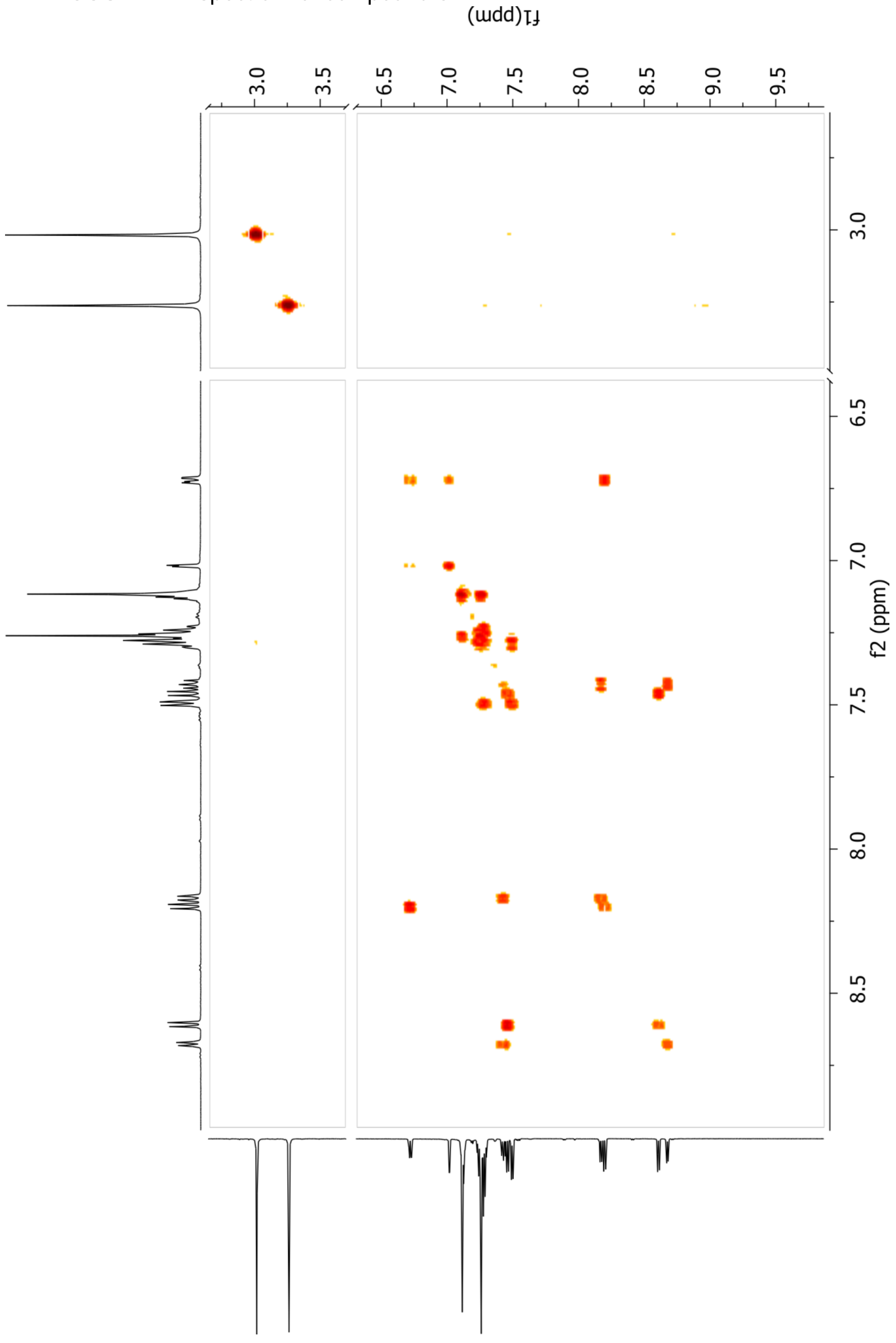

^{1}H-^{13}C HSQC NMR spectrum of compound **54.1**

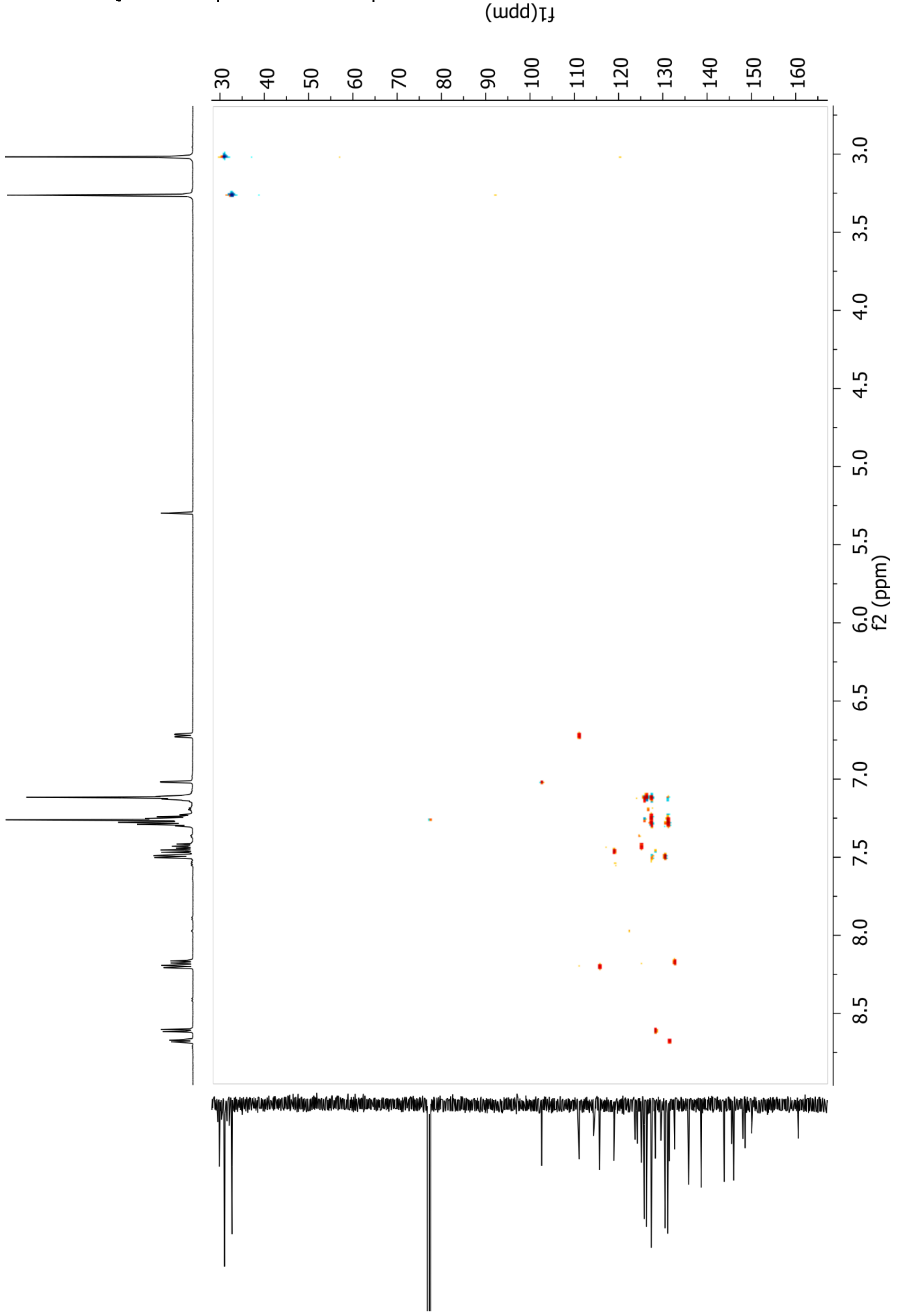

^{1}H-^{13}C HMBC NMR spectrum of compound **54.1**

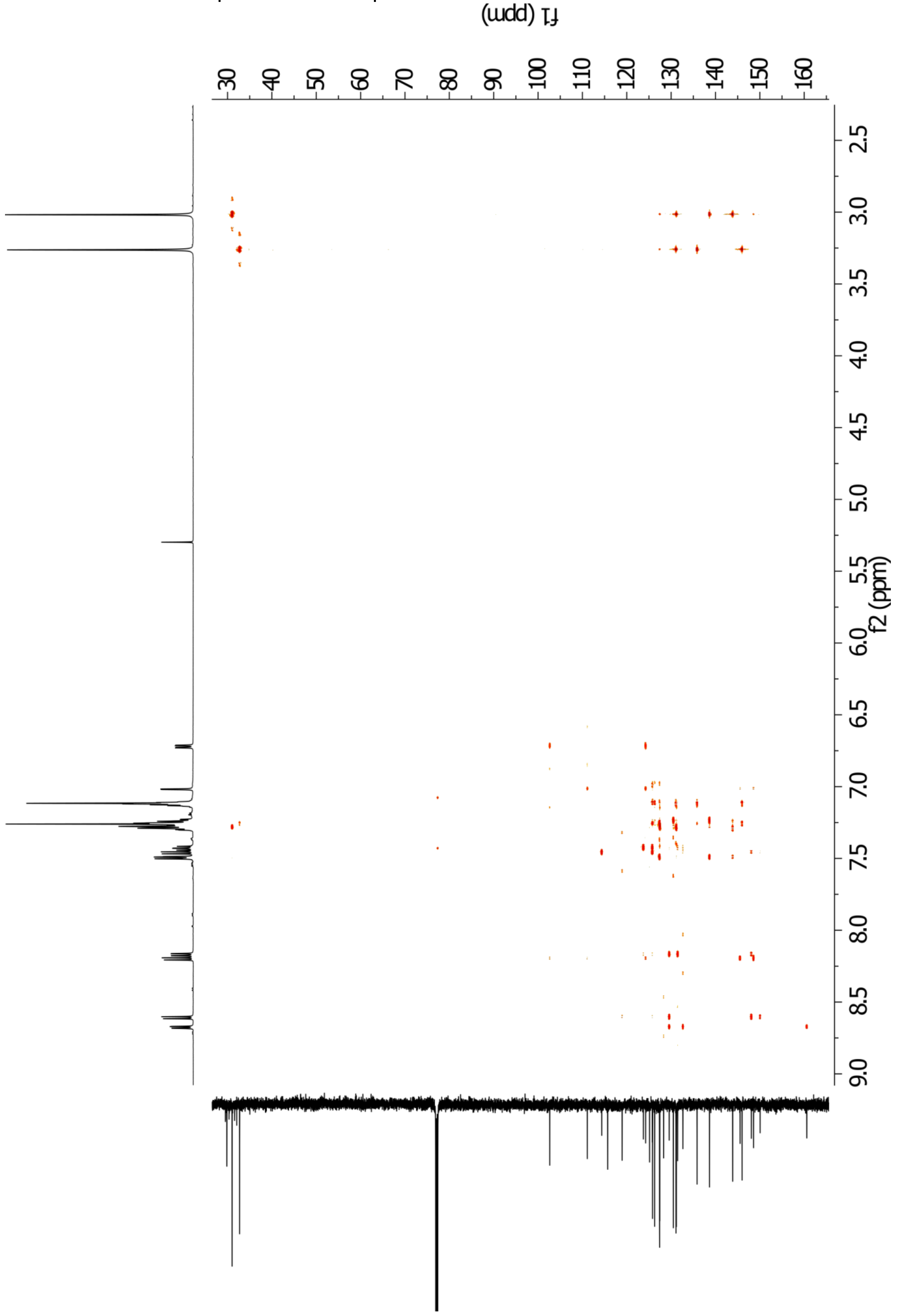

2D NMR assignments of **54.1**

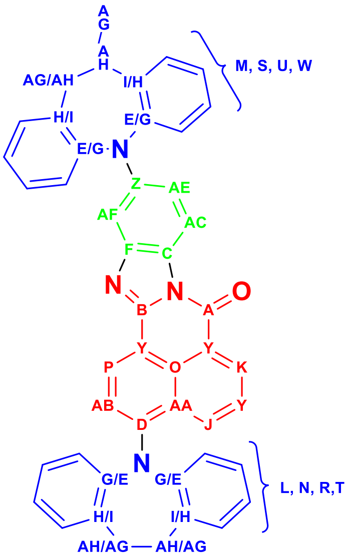

Isomer 1					
**Nr**	**Assignment**	**^{13}C Shift [ppm]**	**HSQC [ppm]**	**HMBC**	**COSY**
**1**	A	160.58		K, Y	
**2**	B	150.06		P, Aw	
**3**	C	148.58		AC, AF, AE	
**4**	D	148.08		P, J, AB	
**5**	E	145.99		AG, M, U, W	
**6**	F	145.54		AF, AC	
**7**	G	143.87		N, AH, R,T	
**8**	H	138.65		AH, T, N	
**9**	I	135.84		AG, S, M	
**10**	J	132.66	8.17	K, AB, Y	Y, K
**11**	K	131.47	8.68	Y, J	Y, J
**12**	L	131.18	7.23 – 7.30	R, N, AH	N
**13**	M	131.10	7.23 – 7.30	U, AG	nd
**14**	N	130.51	7.50	T	L
**15**	O	129.57		K, P, J, AB, Y	
**16**	P	128.30	8.61		AB
**17**	R	127.44	7.23 – 7.30	T, L, AH	nd
**18**	S	127.40	7.15 – 7.09	M, W, AG	nd
**19**	T	127.38	7.23 – 7.30	N, R, L	nd
**20**	U	126.29	7.15 – 7.09	W	nd
**21**	W	125.81	7.15 – 7.09	M, U	nd
**22**	X	125.75		K, P, J, AB, Y	
**23**	Y	125.14	7.43	K	J, K
**24**	Z	124.25		AC, AF, AE	
**25**	AA	123.74		Y, K, P, J	
**26**	AB	118.95	7.46	P	P
**27**	AC	115.70	8.20		AE
**28**	AD	114.36		AB, J	
**29**	AE	111.11	6.72	AC, AF	AF, AC
**30**	AF	102.63	7.02	AE, AC	AE
**31**	AG	32.72	3.26	M	
**32**	AH	31.03	3.02	L	

^{1}H NMR spectrum of compound **54.4**

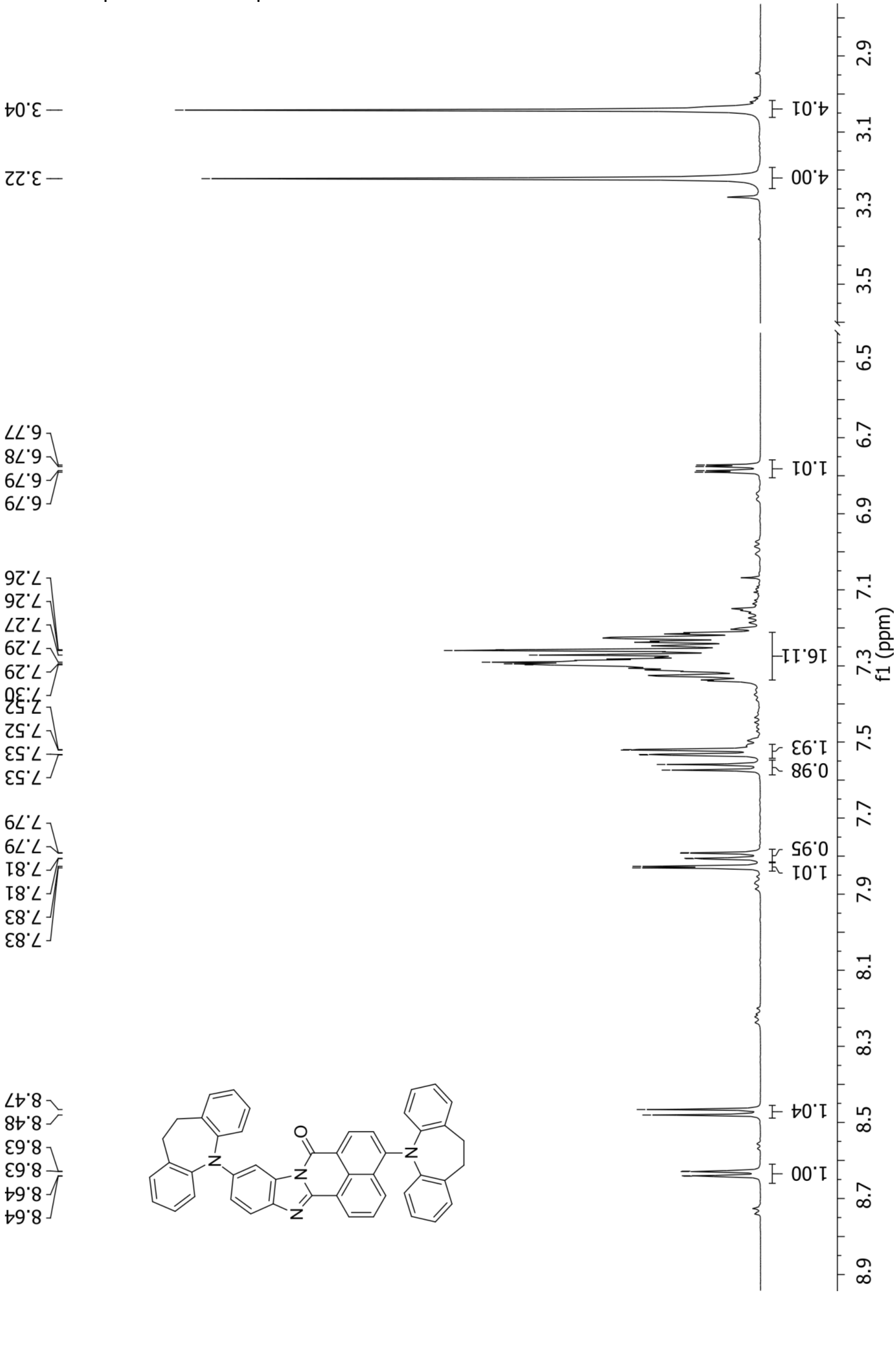

^{13}C NMR spectrum of compound **54.4**

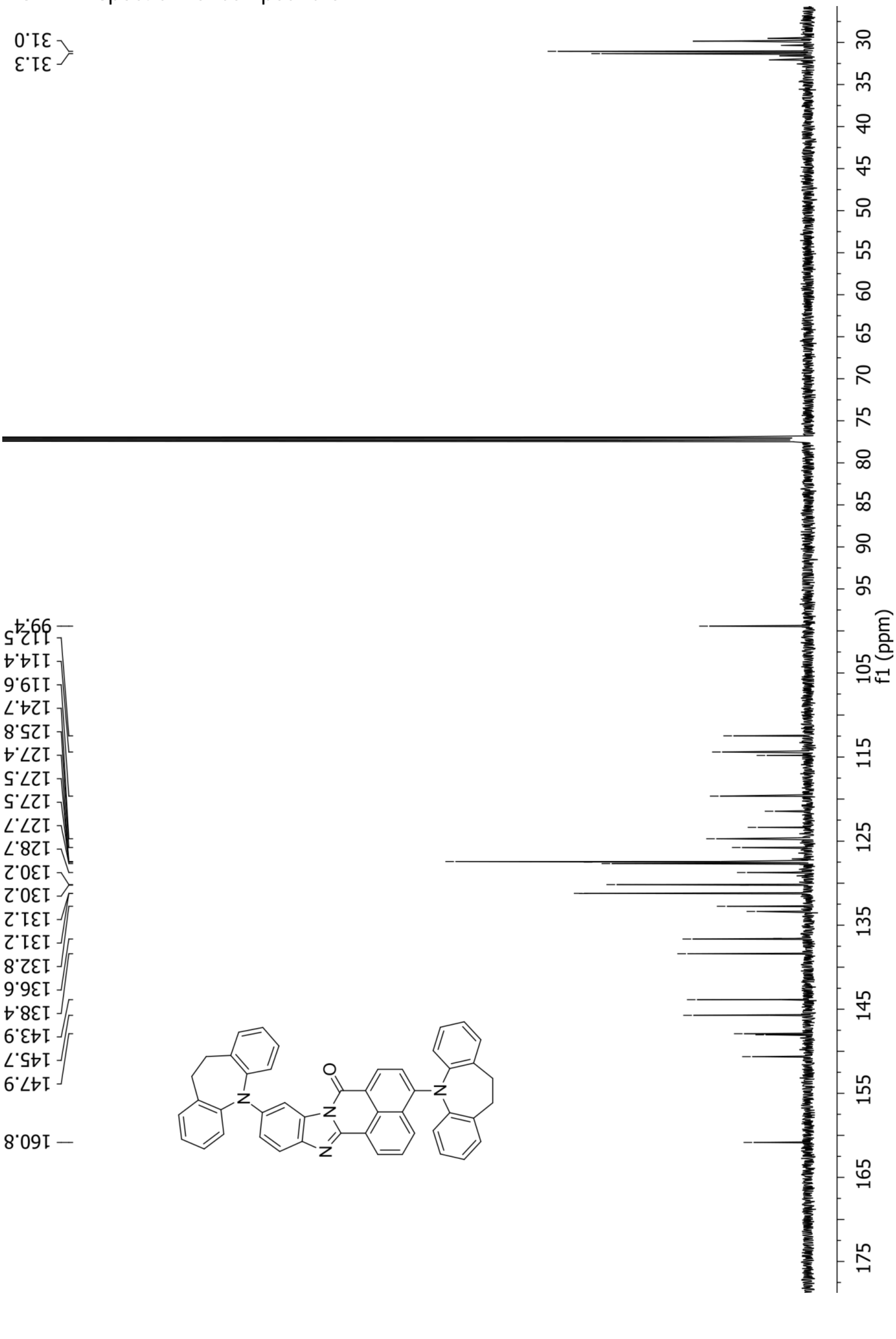

^{1}H-^{1}H COSY NMR spectrum of compound **54.4**

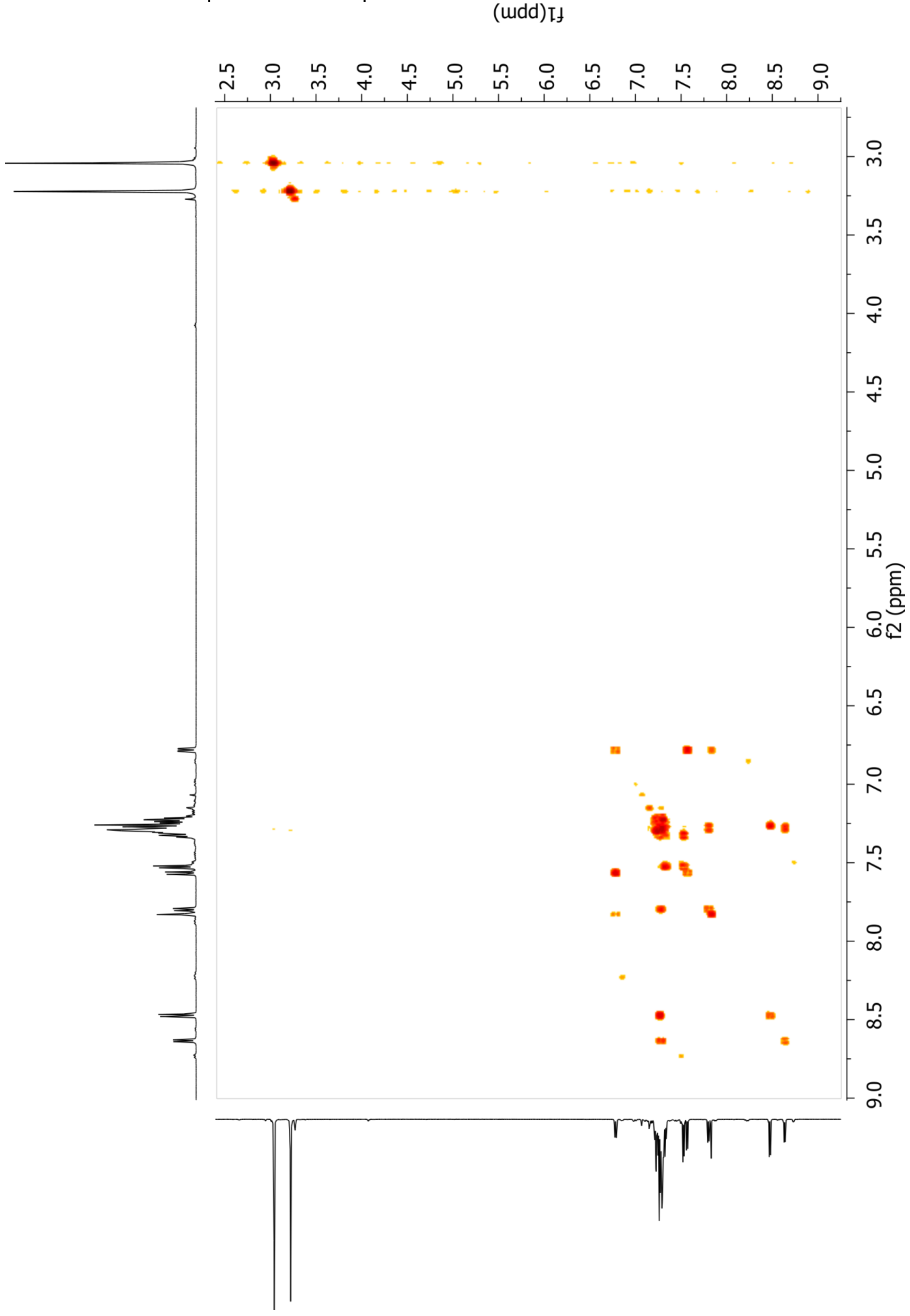

^{1}H-^{13}C HSQC NMR spectrum of compound **54.4**

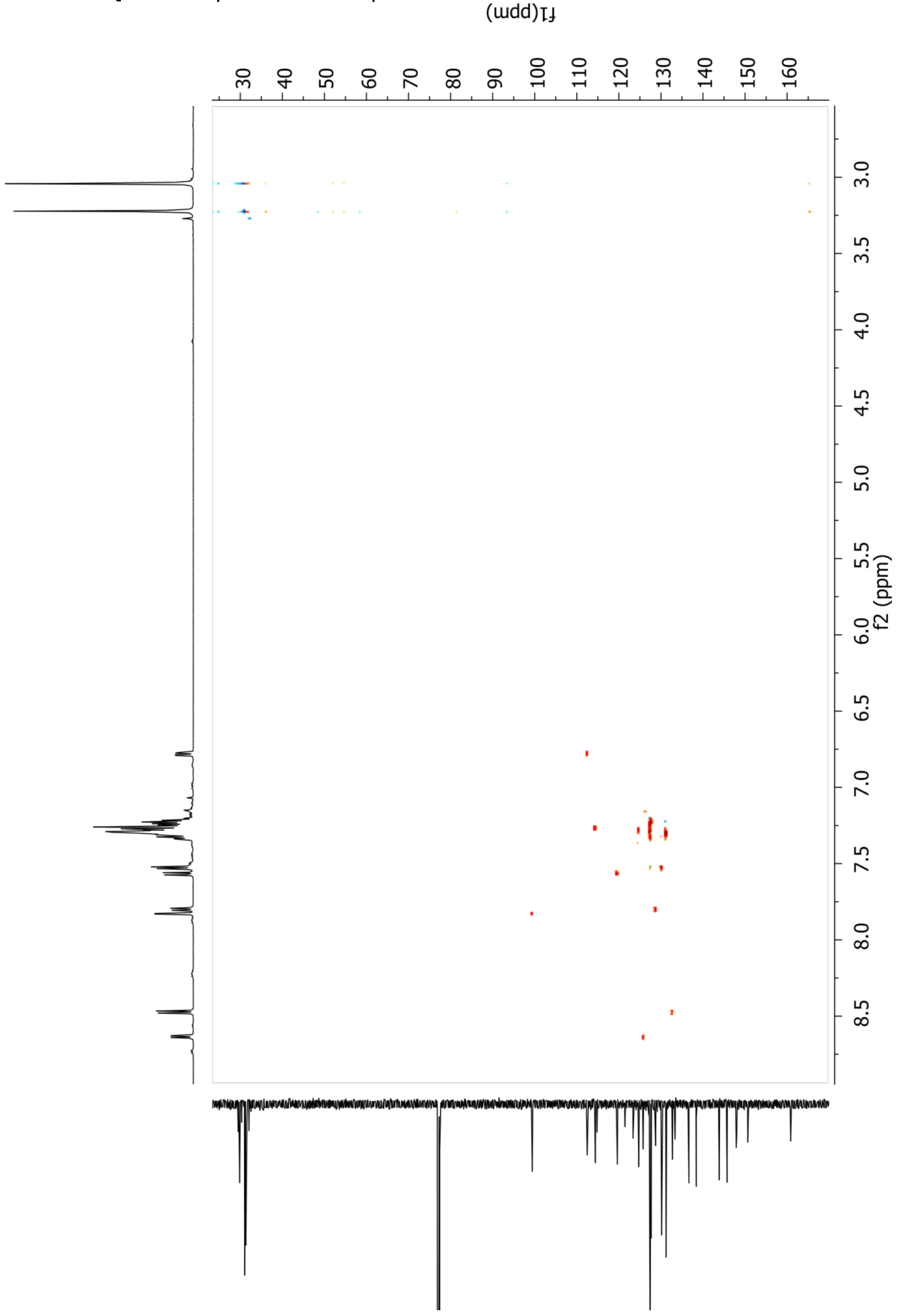

^{1}H-^{13}C HMBC NMR spectrum of compound **54.4**

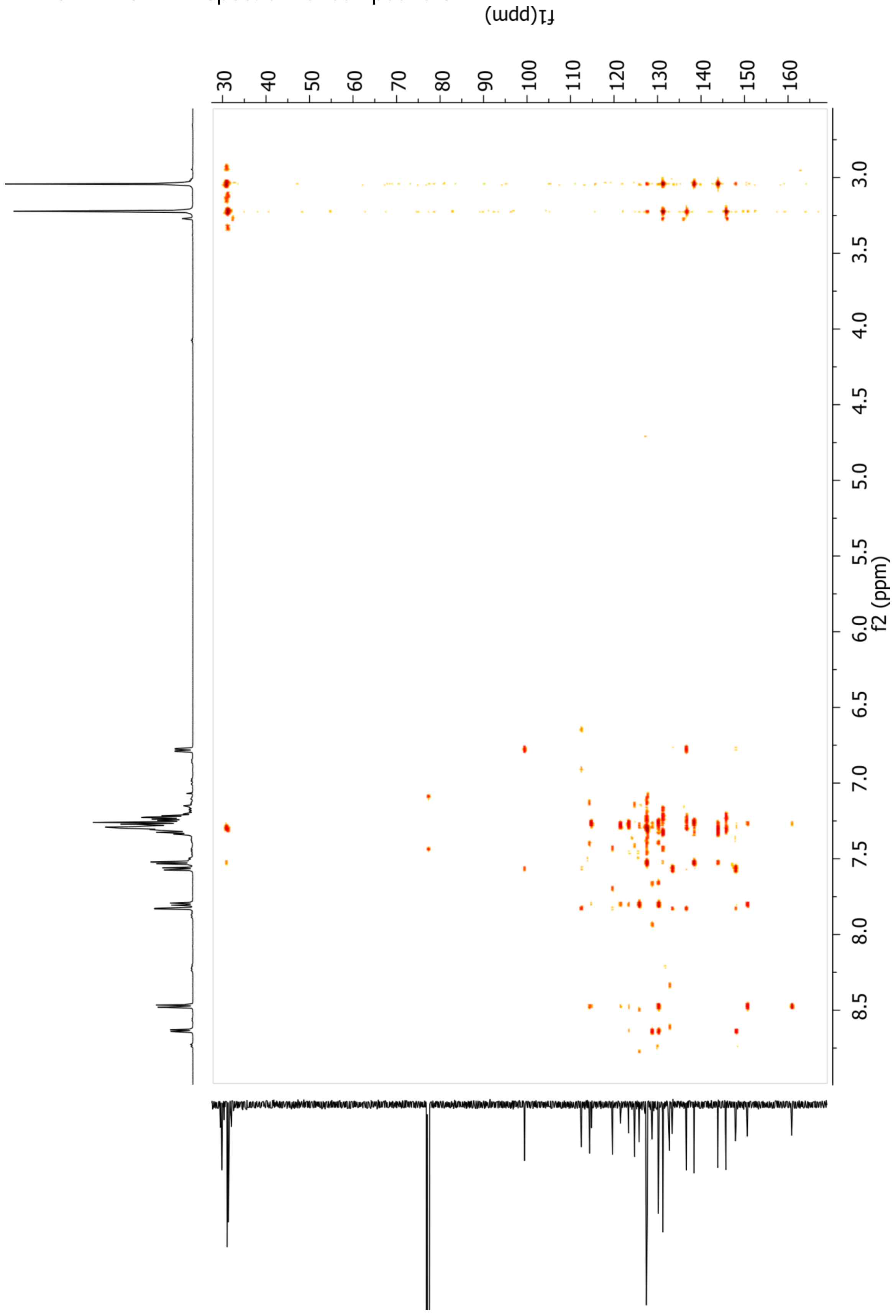

2D NMR assignments of **54.4**

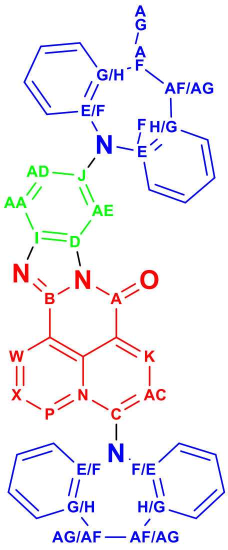

Nr	Assignment	^{13}C Shift [ppm]	HSQC [ppm]	HMBC	COSY
		**Isomer 3**			
**1**	A	160.84		K, AC	
**2**	B	150.64		K, P, AC	
**3**	C	148.04		W, X	
**4**	D	147.91		AE, AA, AD	
**5**	E	145.72		AF	
**6**	F	143.86		AG, O	
**7**	G	138.40		O, AG	
**8**	H	136.65		AF	
**9**	I	136.58		AD, AE, AA	
**10**	J	133.37		AE, AA, AD	
**11**	K	132.76	8.47	AC	AC
**12**	L	131.23	7.35 – 7.20	AF	nd
**13**	M	131.22	7.35 – 7.20	AG	nd
**14**	N	130.24		W, K, P	
**15**	O	130.17	7.53	nd	nd
**16**	P	128.73	7.80	W, X	X
**17**	R	127.67	7.35 – 7.20	nd	nd
**18**	S	127.50	7.35 – 7.20	nd	nd
**19**	T	127.48	7.35 – 7.20	nd	nd
**20**	U	127.43	7.35 – 7.20	nd	nd
**21**	W	125.77	8.63	X, P	X, P
**22**	X	124.72	7.35 – 7.20	nd	K, W
**23**	Y	123.37		X, P, W, K	
**24**	Z	121.44		K, P, X	
**25**	AA	119.65	7.57		AD
**26**	AB	114.81		P, X	
**27**	AC	114.40	7.35 – 7.20	K	K
**28**	AD	112.48	6.78	AE, AA	AE, AA
**29**	AE	99.43	7.83	AA, AD	AD
**30**	AF	31.33	3.22	nd	
**31**	AG	31.05	3.04	O	

^{1}H NMR spectrum of compound **55.1**

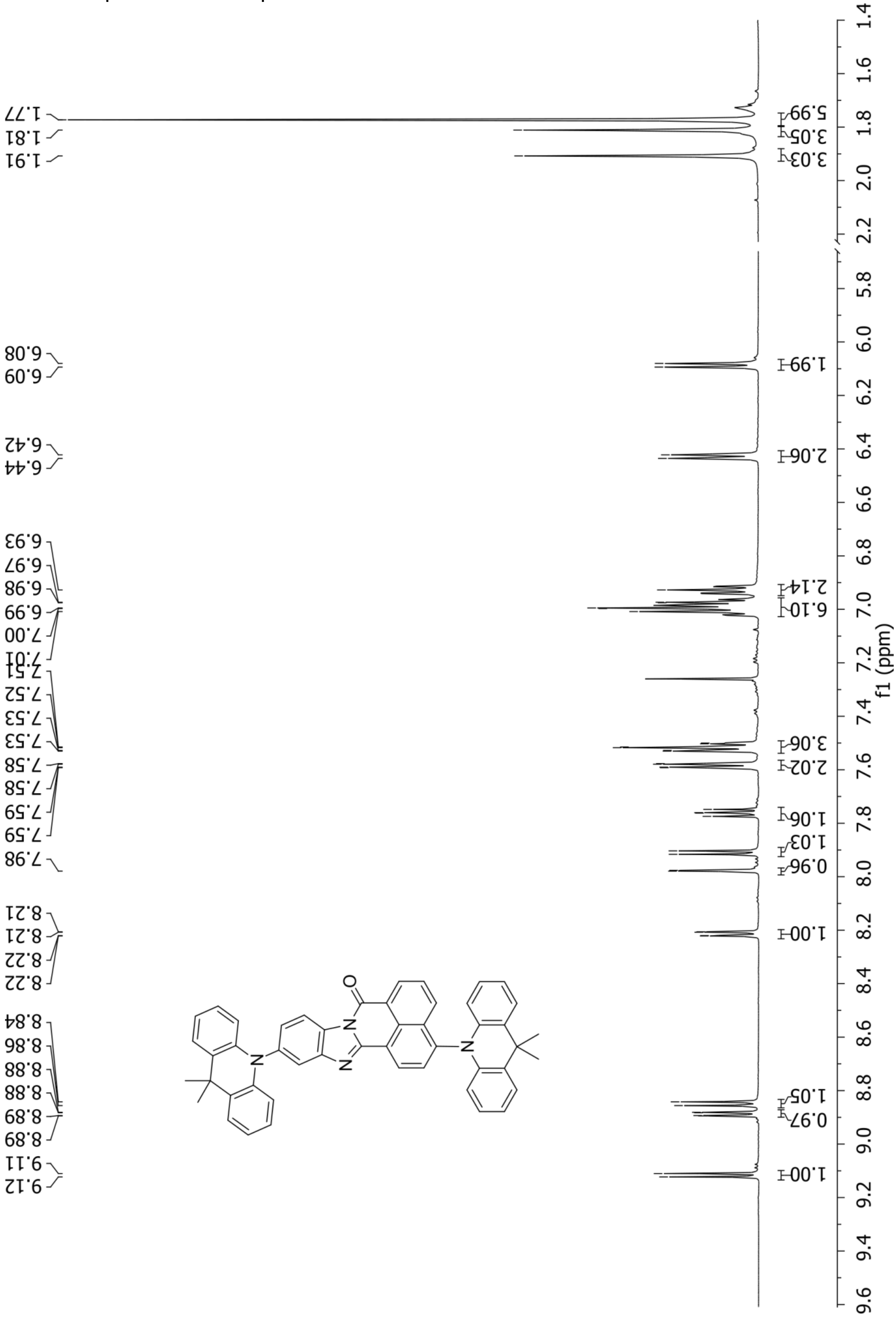

^{13}C NMR spectrum of compound **55.1**

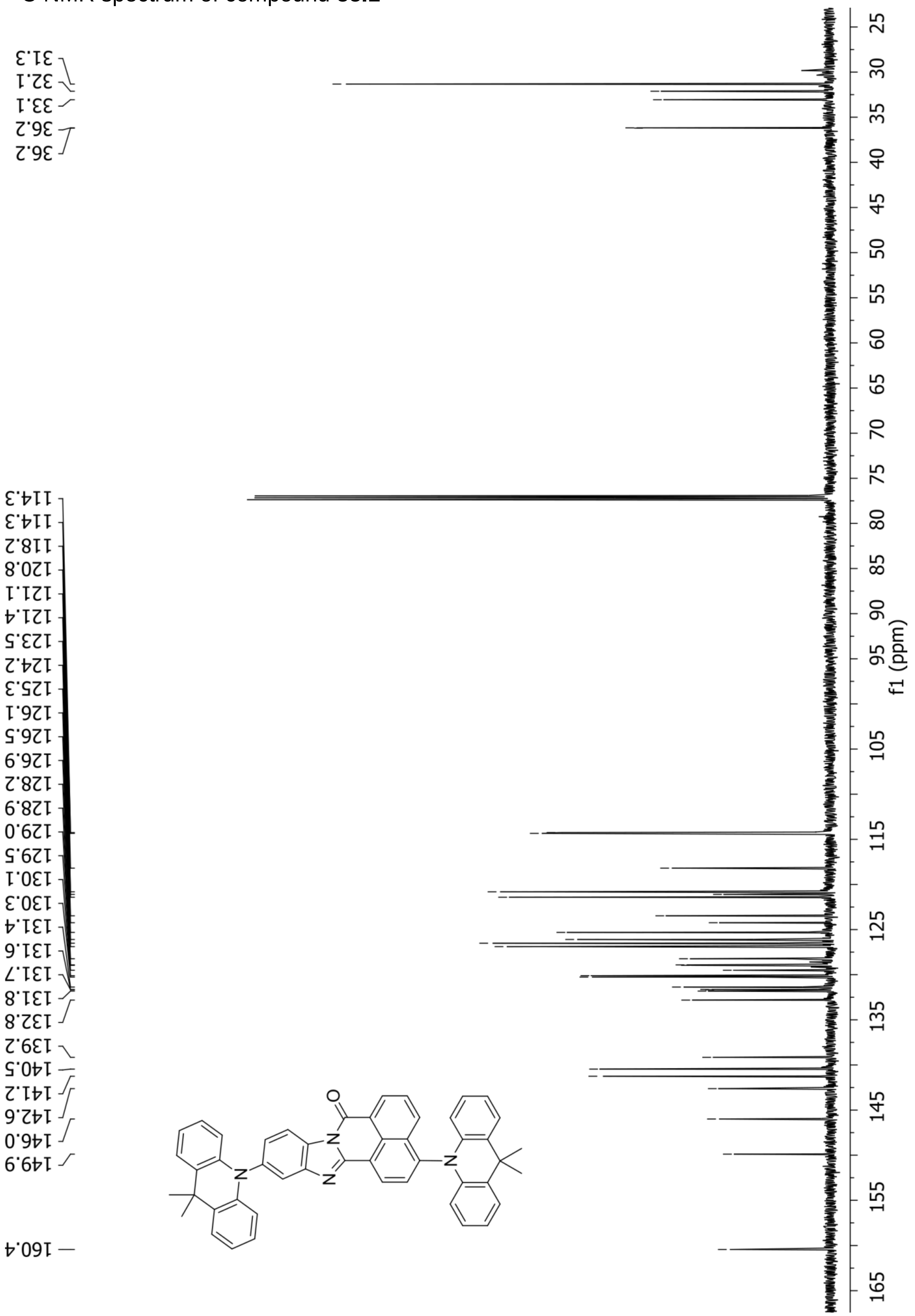

^{1}H-^{1}H COSY NMR spectrum of compound **55.1**

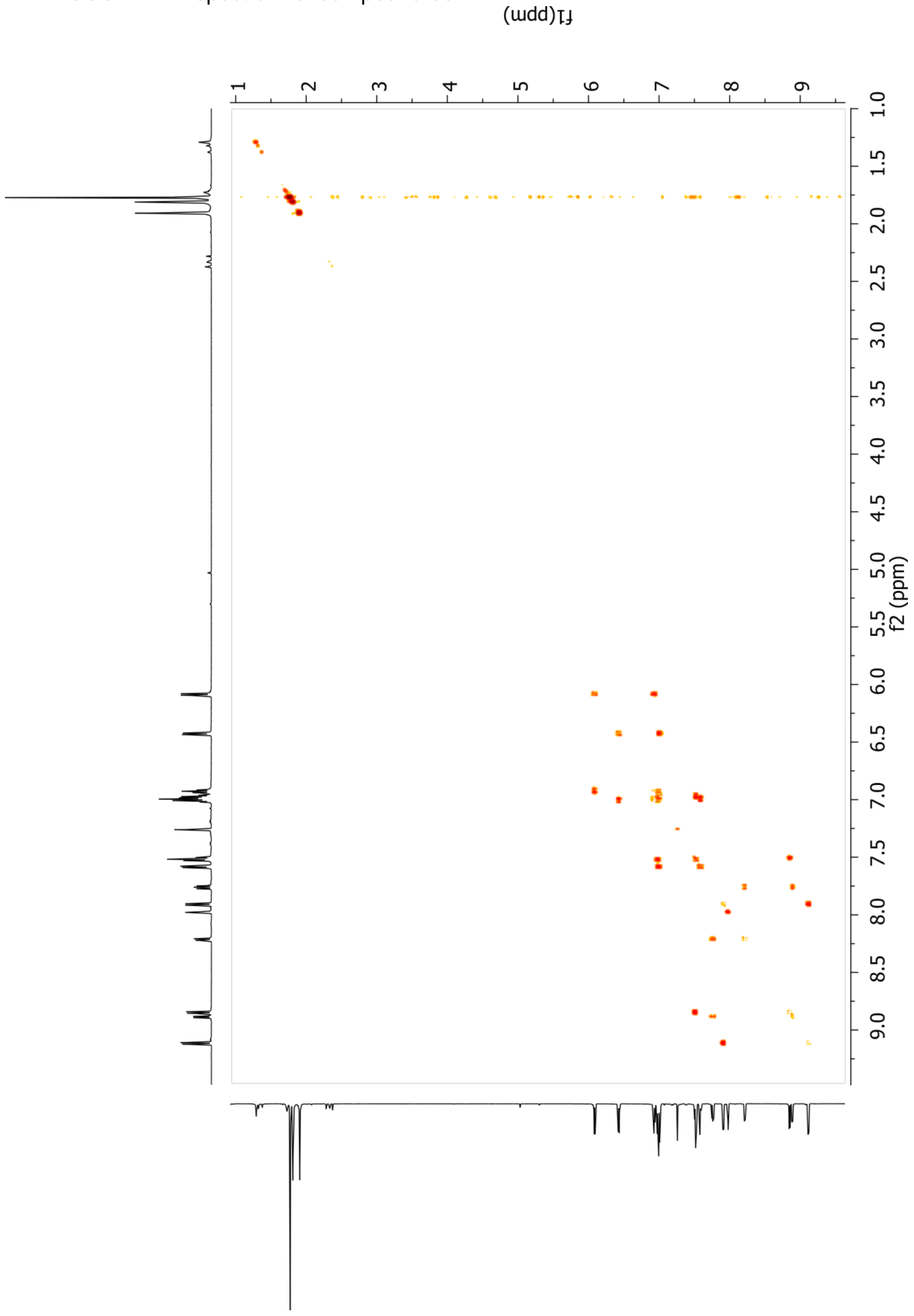

^{1}H-^{13}C HSQC NMR spectrum of compound **55.1**

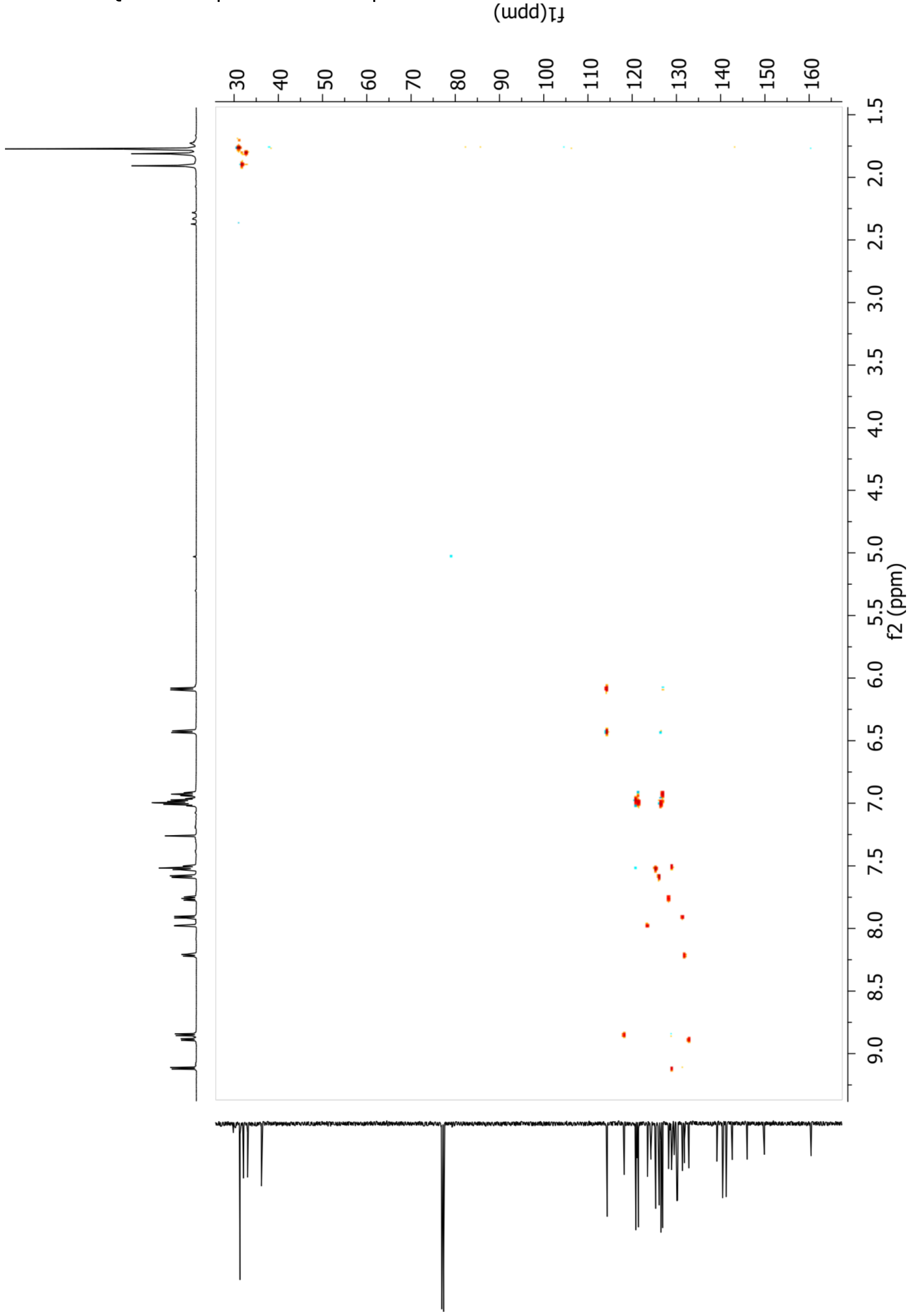

^{1}H-^{13}C HMBC NMR spectrum of compound **55.1**

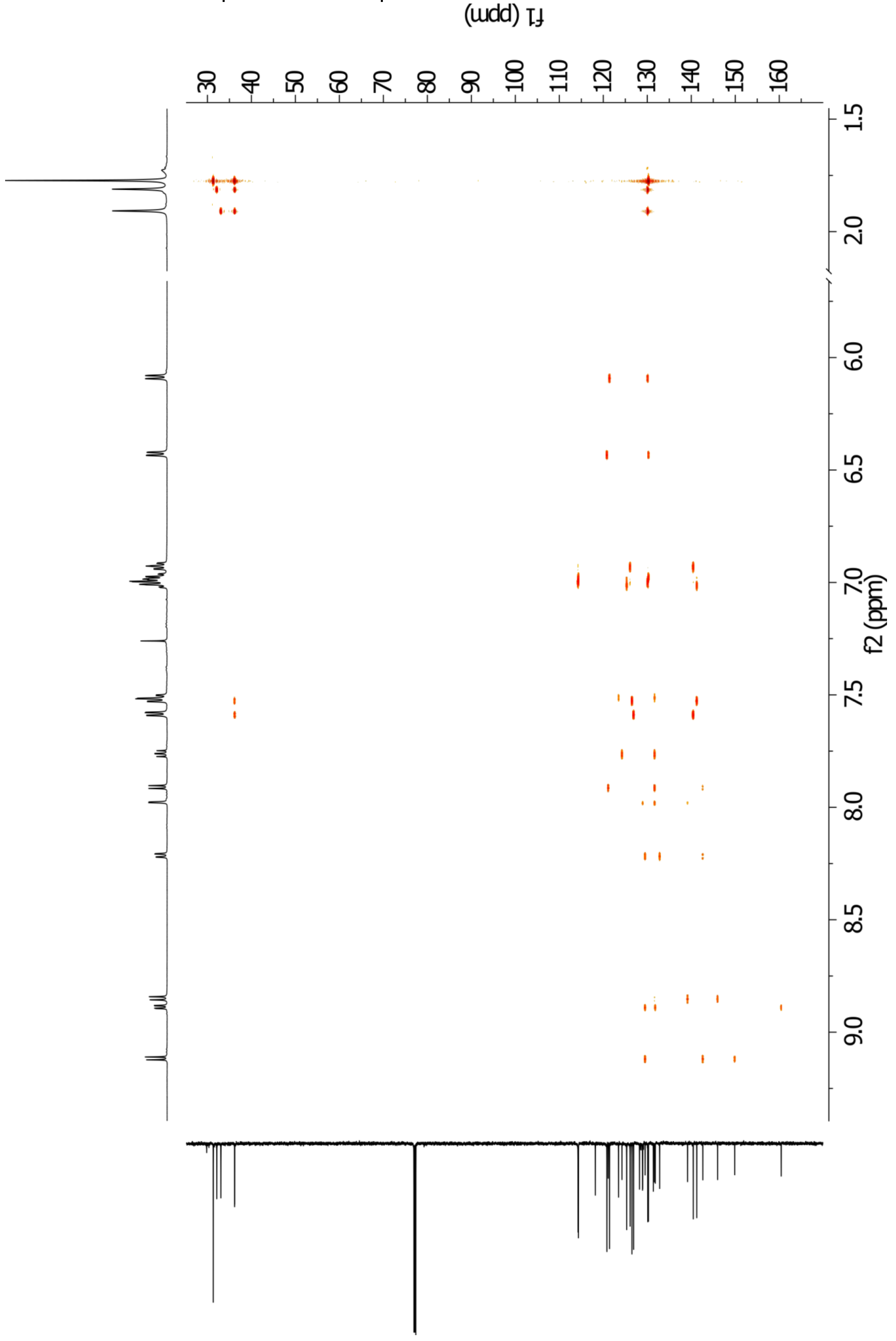

2D NMR assignments of **55.1**

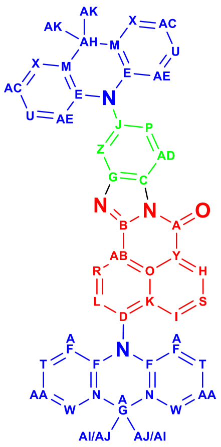

Nr	Assignment	^{13}C Shift [ppm]	HSQC [ppm]	HMBC	COSY
			Isomer 1		
1	A	160.45		H, S	
2	B	149.88		R, L	
3	C	145.99		AD, Z, P	
4	D	142.62		R, I, L	
5	E	141.25		X, AE, U	
6	F	140.45		AF, T, W	
7	G	139.15		AD, Z, P	
8	H	132.81	8.89	I, S	S
9	I	131.81	8.21	H	S
10	J	131.66		P, Z, AD	
11	K	131.64		S, L	
12	L	131.37	7.91		R
13	M	130.26		AK, AE, AC	
14	N	130.10		AI, AJ, AF, AA	
15	O	129.51		I, H, S	
16	P	128.96	7.54 – 7.49	Z	AD, Z
17	R	128.91	9.12	L	L
18	S	128.23	7.76	H	H, I
19	T	126.89	6.95 – 6.91	W	AF, AA
20	U	126.51	7.03 – 6.96	X	AE
21	W	126.10	7.58	AF, T, AA	AA
22	X	125.31	7.54 – 7.49	U	AC
23	Y	124.24		S, I, R, H	
24	Z	123.47	7.98	AD, P	P
25	AA	121.41	7.03 – 6.96	AF	W, T
26	AB	121.11		L, R	
27	AC	120.81	7.03 – 6.96	AE	X
28	AD	118.19	8.85	Z	P
29	AE	114.34	6.43	X, AC	U
30	AF	114.25	6.09	W, T, AA	T
31	AG	36.20		W, AA, AF, AI, AJ	
32	AH	36.17		X, U, AC, AE, AK	
33	AI	33.05	1.81	AJ	
34	AJ	32.12	1.91	AI	
35	AK	31.32	1.77	AK	

^{1}H NMR spectrum of compound **55.3**

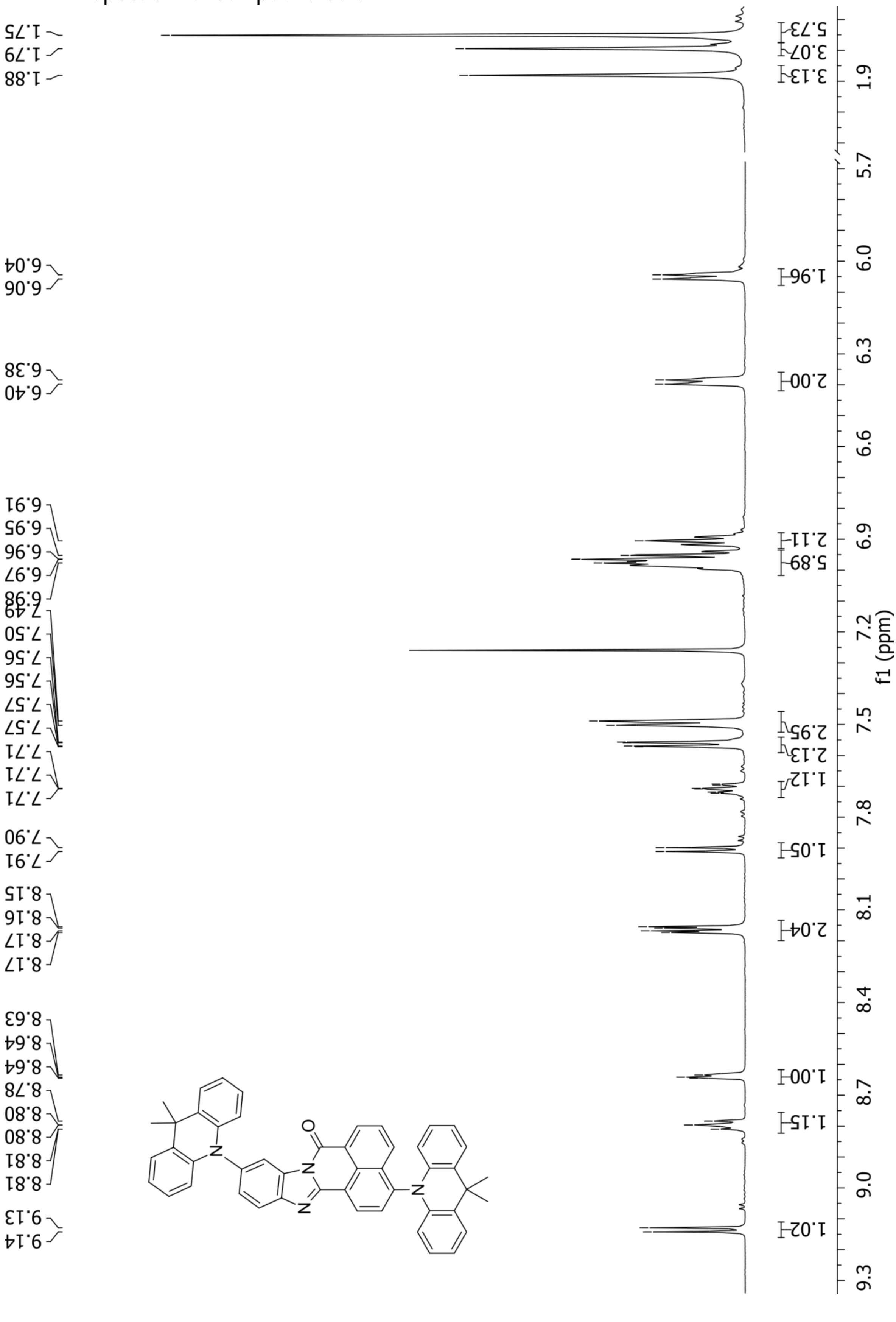

^{13}C NMR spectrum of compound **55.3**

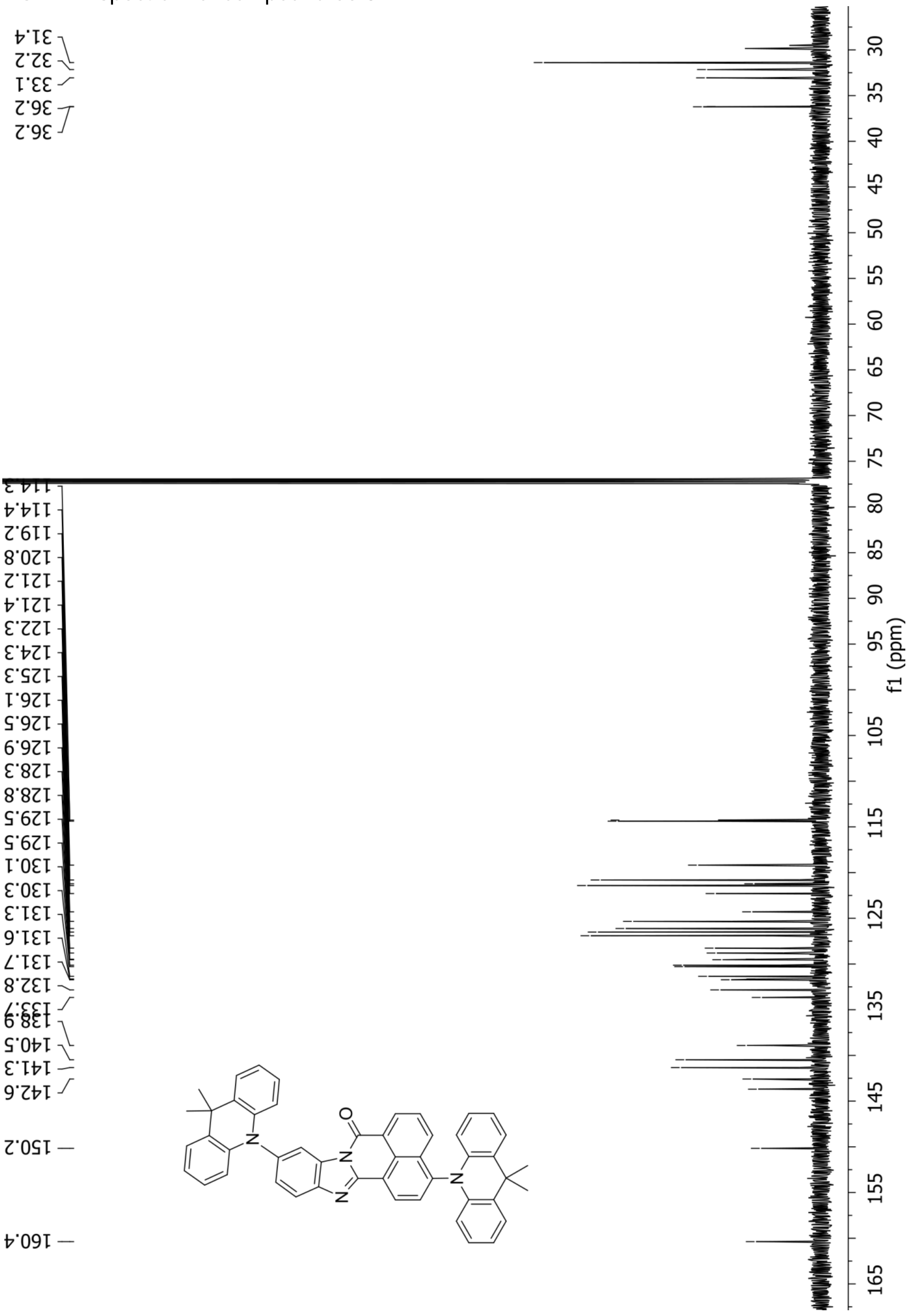

^{1}H-^{1}H COSY NMR spectrum of compound **55.3**

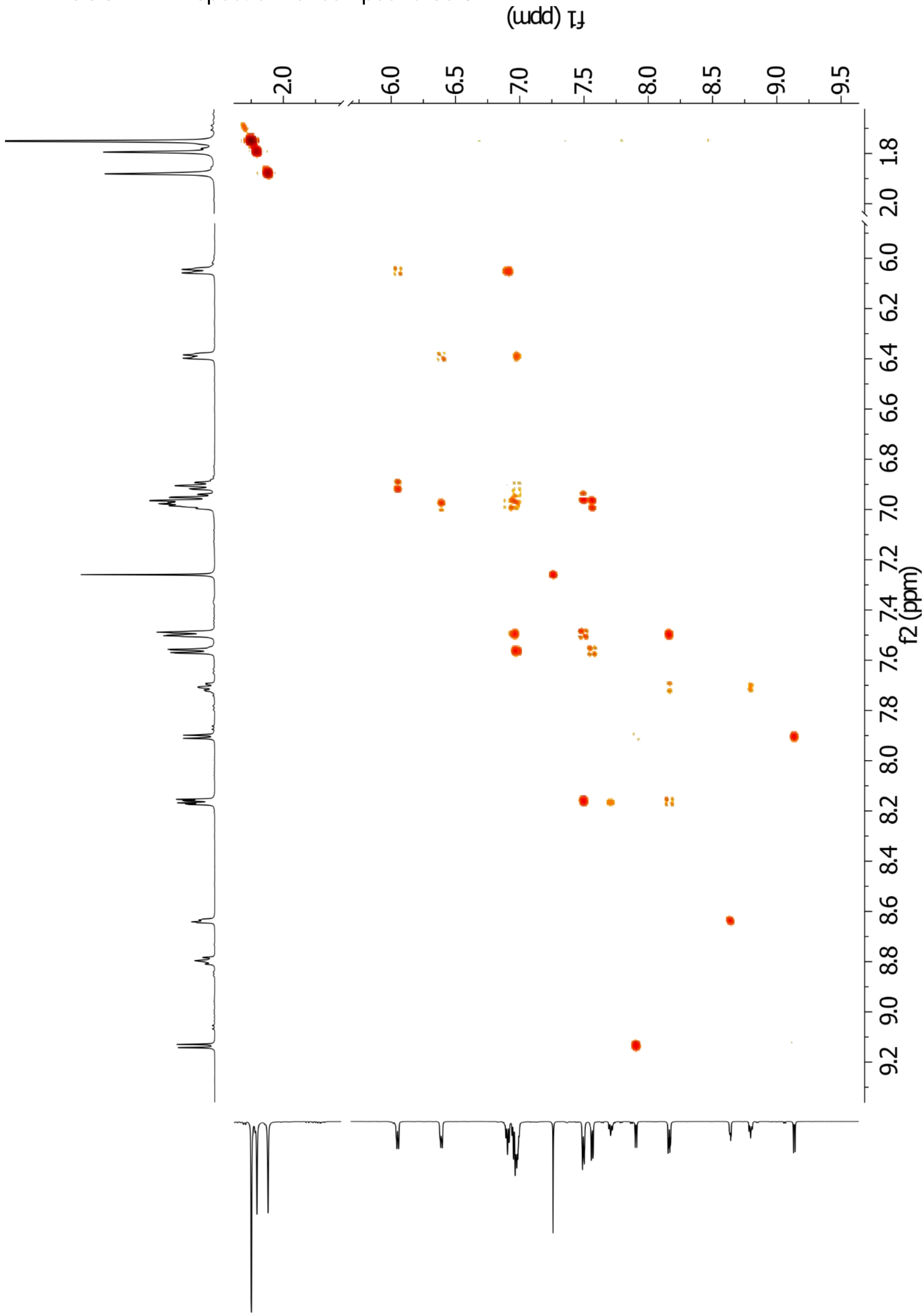

^{1}H-^{13}C HSQC NMR spectrum of compound **55.3**

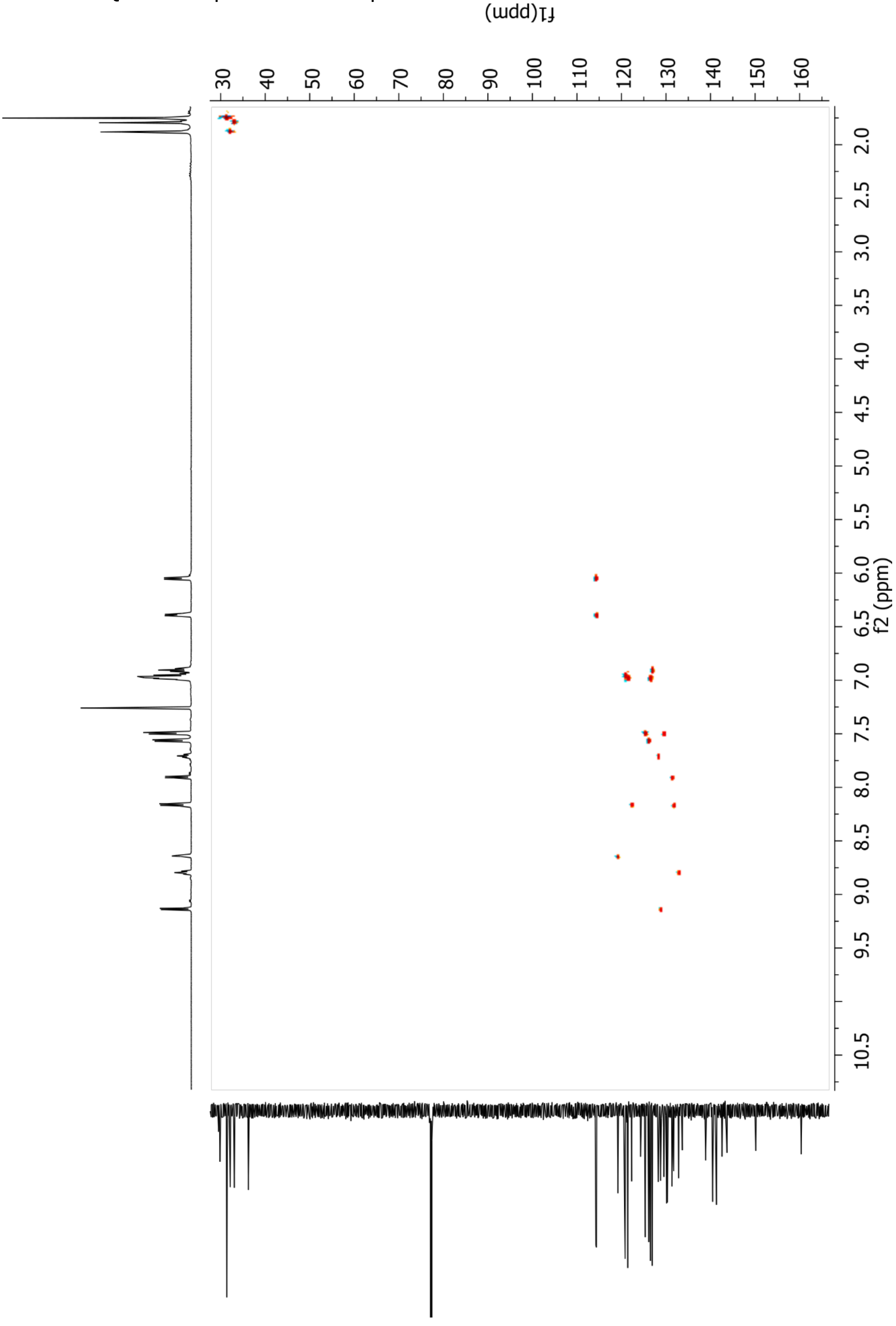

^{1}H-^{13}C HMBC NMR spectrum of compound **55.3**

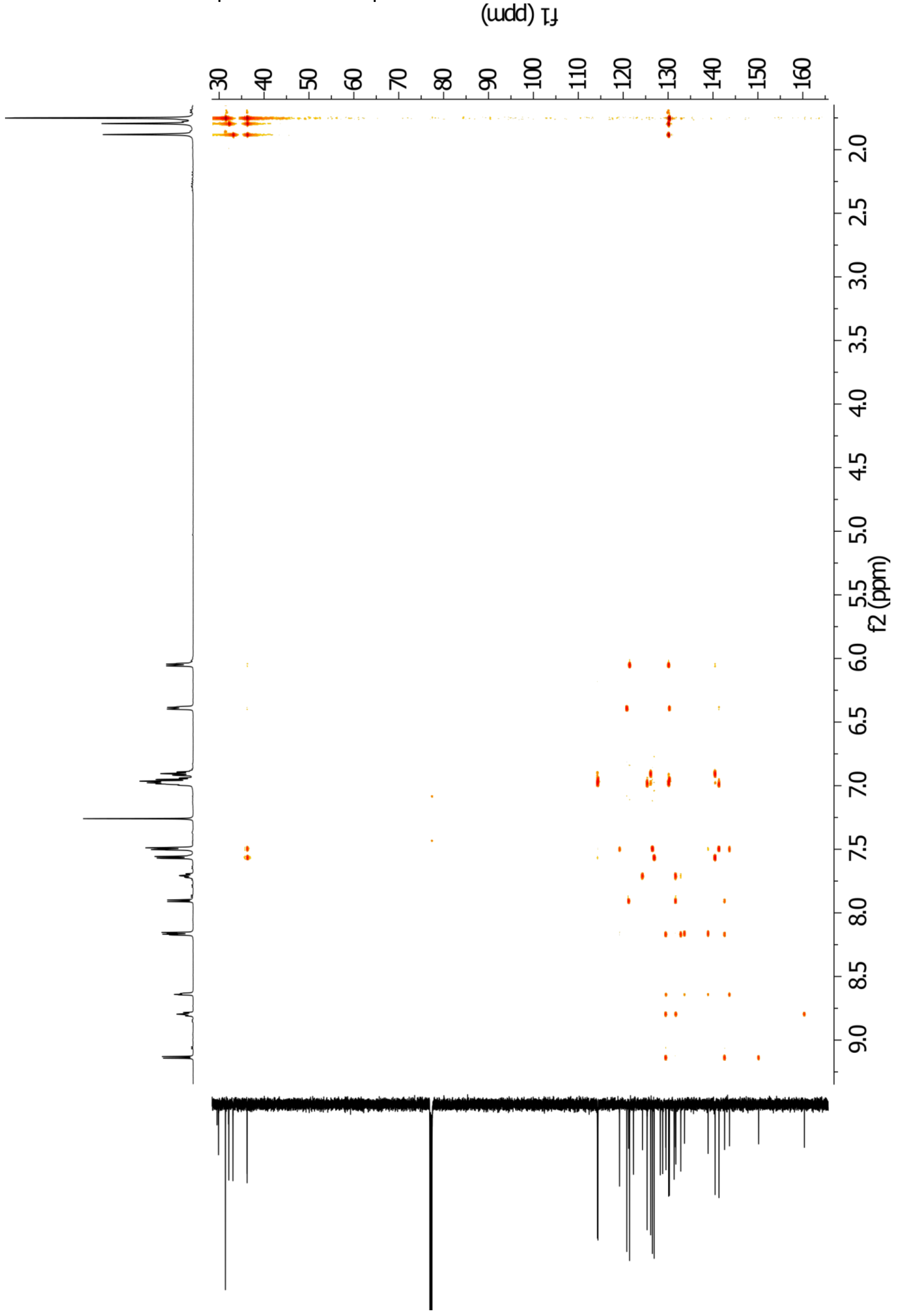

2D NMR assignments of **55.3**

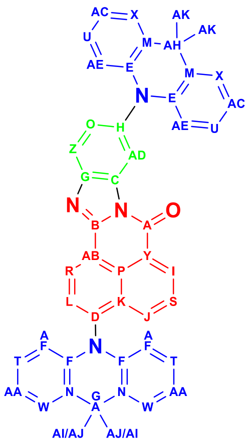

Isomer 3					
**Nr**	**Assignment**	**^{13}C Shift [ppm]**	**HSQC [ppm]**	**HMBC**	**COSY**
**1**	A	160.36		I	
**2**	B	150.17		R	
**3**	C	143.68		AD, O	
**4**	D	142.58		R, L, J	
**5**	E	141.33		X, U, AC, AE	
**6**	F	140.47		W, T, AA, AF	
**7**	G	138.91		Z, AD, O	
**8**	H	133.66		Z, AD	
**9**	I	132.83	8.82 – 8.76	J, S	S
**10**	J	131.72	8.16	I, L, S	S
**11**	K	131.64		R, J	
**12**	L	131.34	7.90		R
**13**	M	130.29		AC, AE, AK	
**14**	N	130.13		T, AA, AF, AI, AJ	
**15**	O	129.53	7.50	AD	Z
**16**	P	129.47		R, I, J	
**17**	R	128.79	9.14		L
**18**	S	128.26	7.71		I, J
**19**	T	126.90	6.93 – 6.88	W	AA, AF
**20**	U	126.50	7.02 – 6.94	X	AE
**21**	W	126.12	7.56	T, AA	AA
**22**	X	125.33	7.50	U, AC	AC
**23**	Y	124.29		S	
**24**	Z	122.29	8.16		O
**25**	AA	121.41	7.02 – 6.94	AF	W, T
**26**	AB	121.22		L	
**27**	AC	120.81	7.02 – 6.94	AE	X
**28**	AD	119.18	8.66 – 8.62	O	O
**29**	AE	114.37	6.39	AC, X	U
**30**	AF	114.26	6.05	T, W, AA	T
**31**	AG	36.23		AJ, AI, W	
**32**	AH	36.19		AK, X	
**33**	AI	33.06	1.79	AJ	
**34**	AJ	32.15	1.88	AI	
**35**	AK	31.39	1.75	AK	

^{1}H NMR spectrum of compound **55.4**

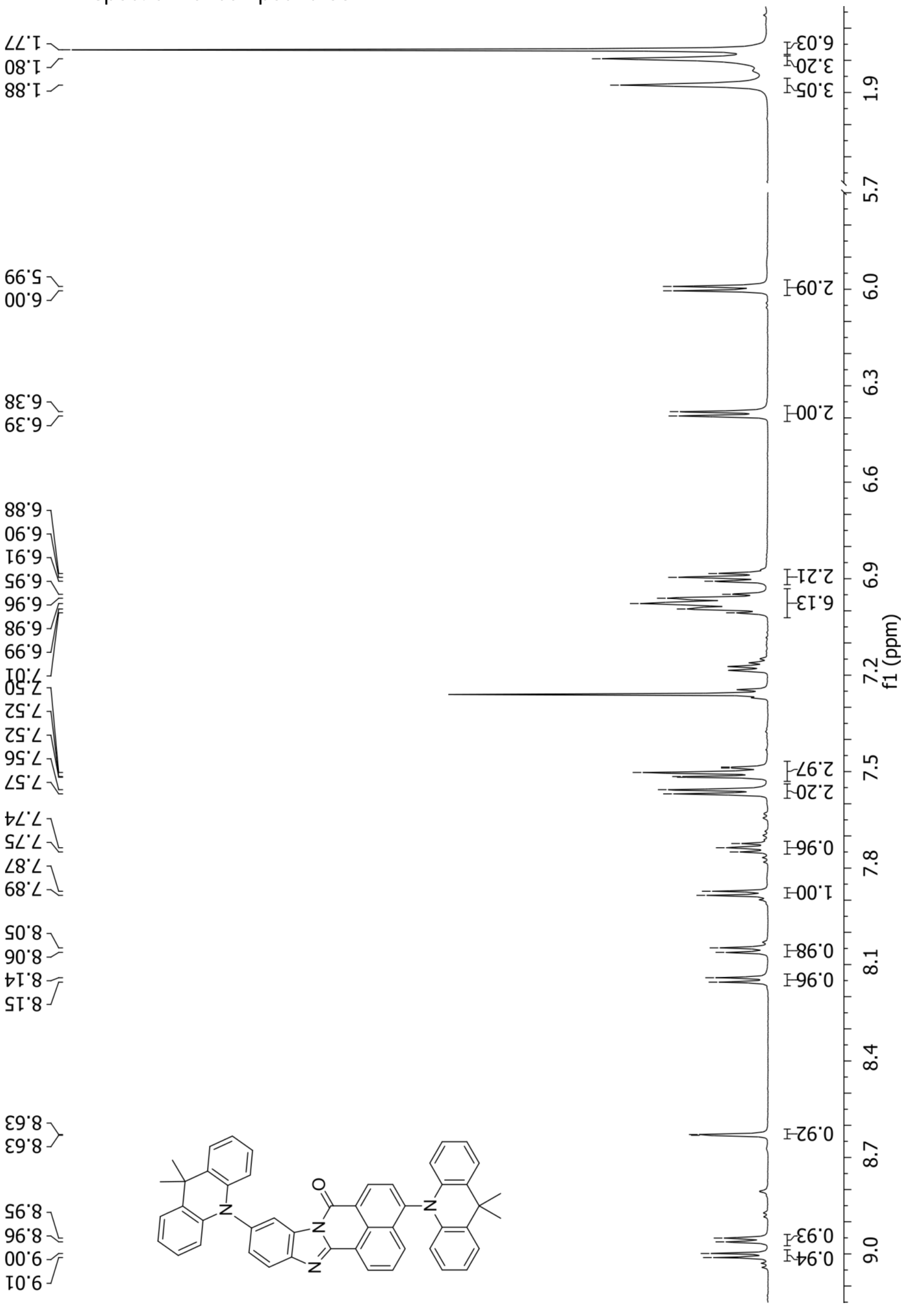

^{13}C NMR spectrum of compound **55.4**

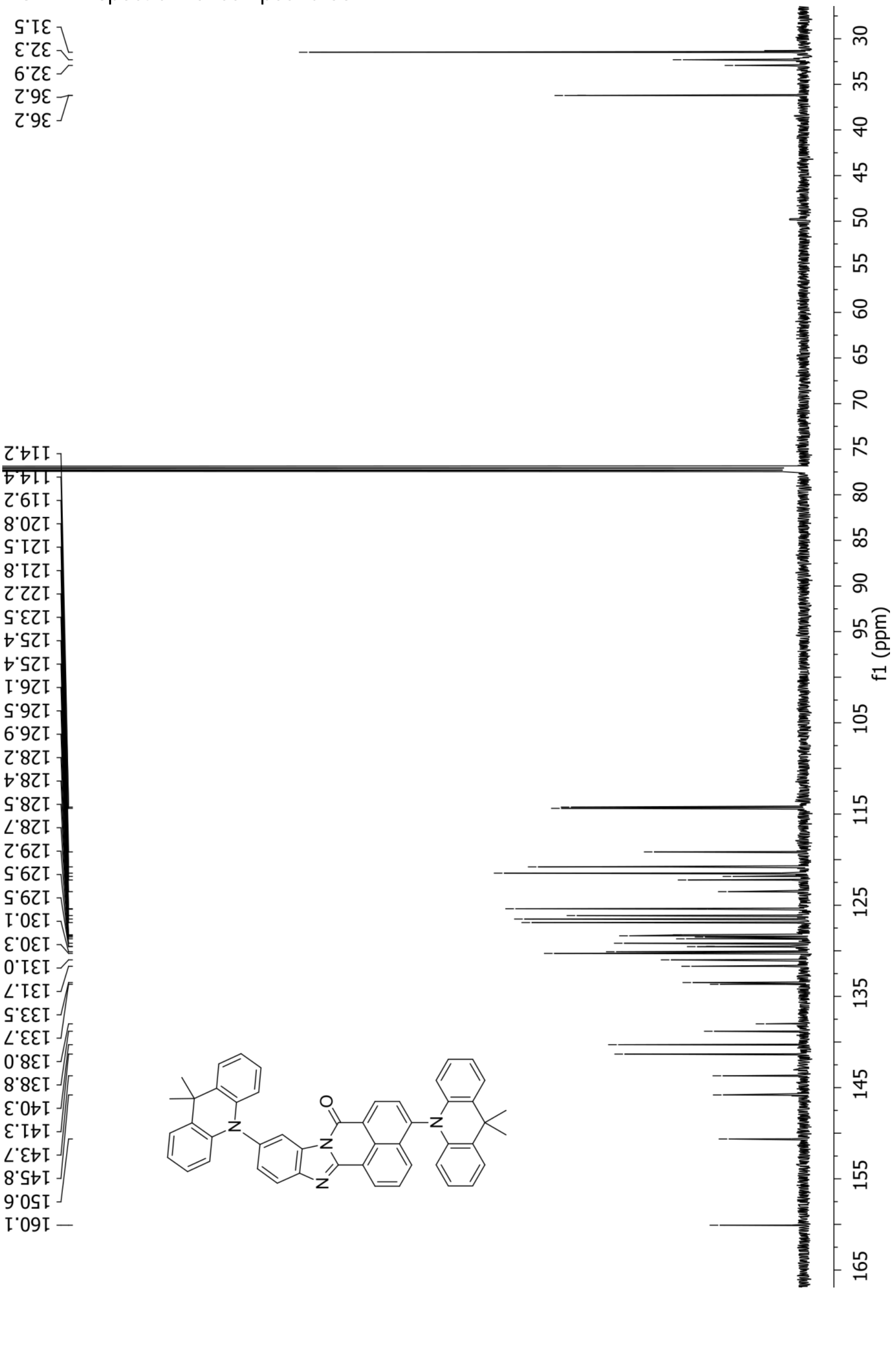

^{1}H-^{1}H COSY NMR spectrum of compound **55.4**

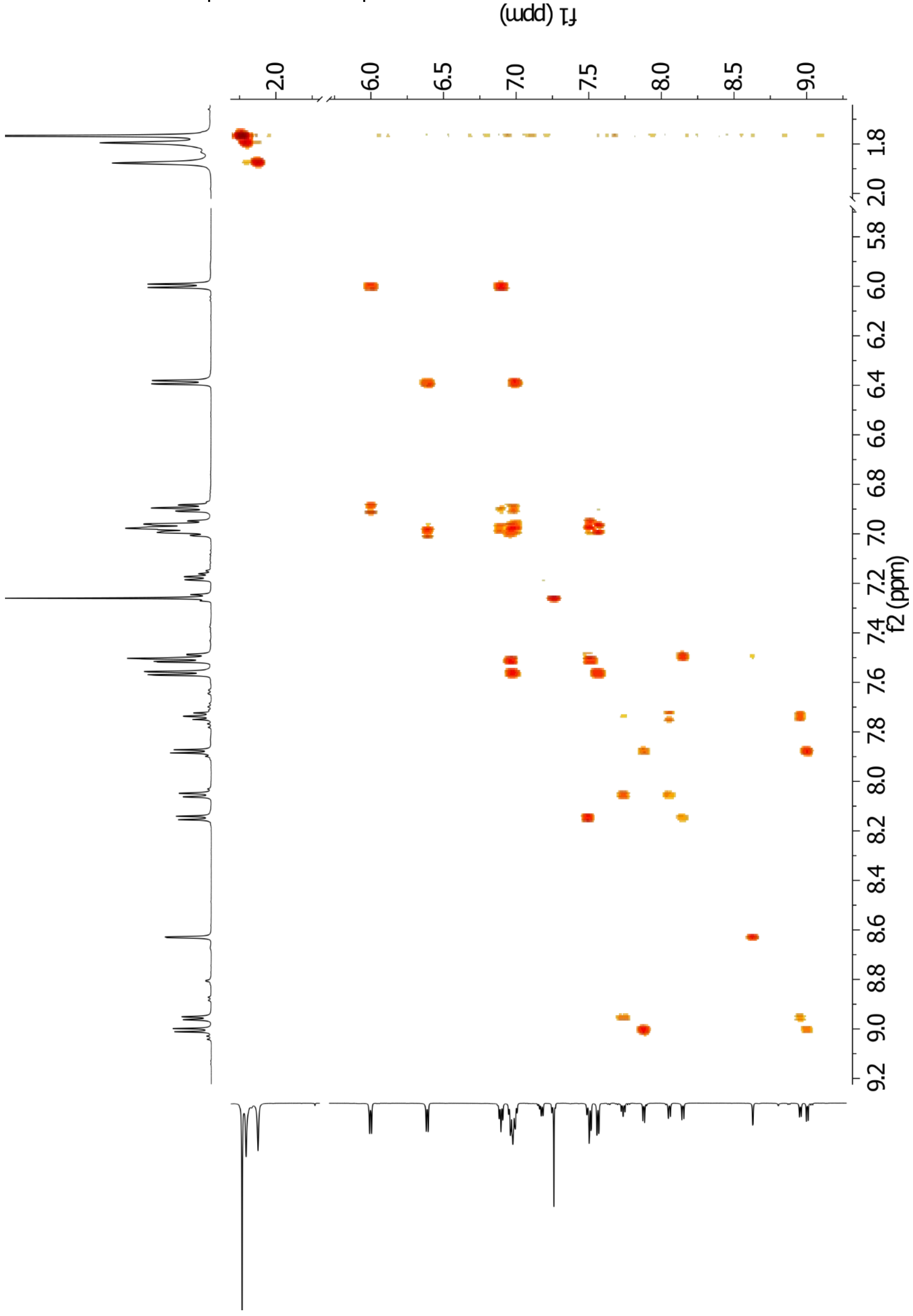

^{1}H-^{13}C HSQC NMR spectrum of compound **55.4**

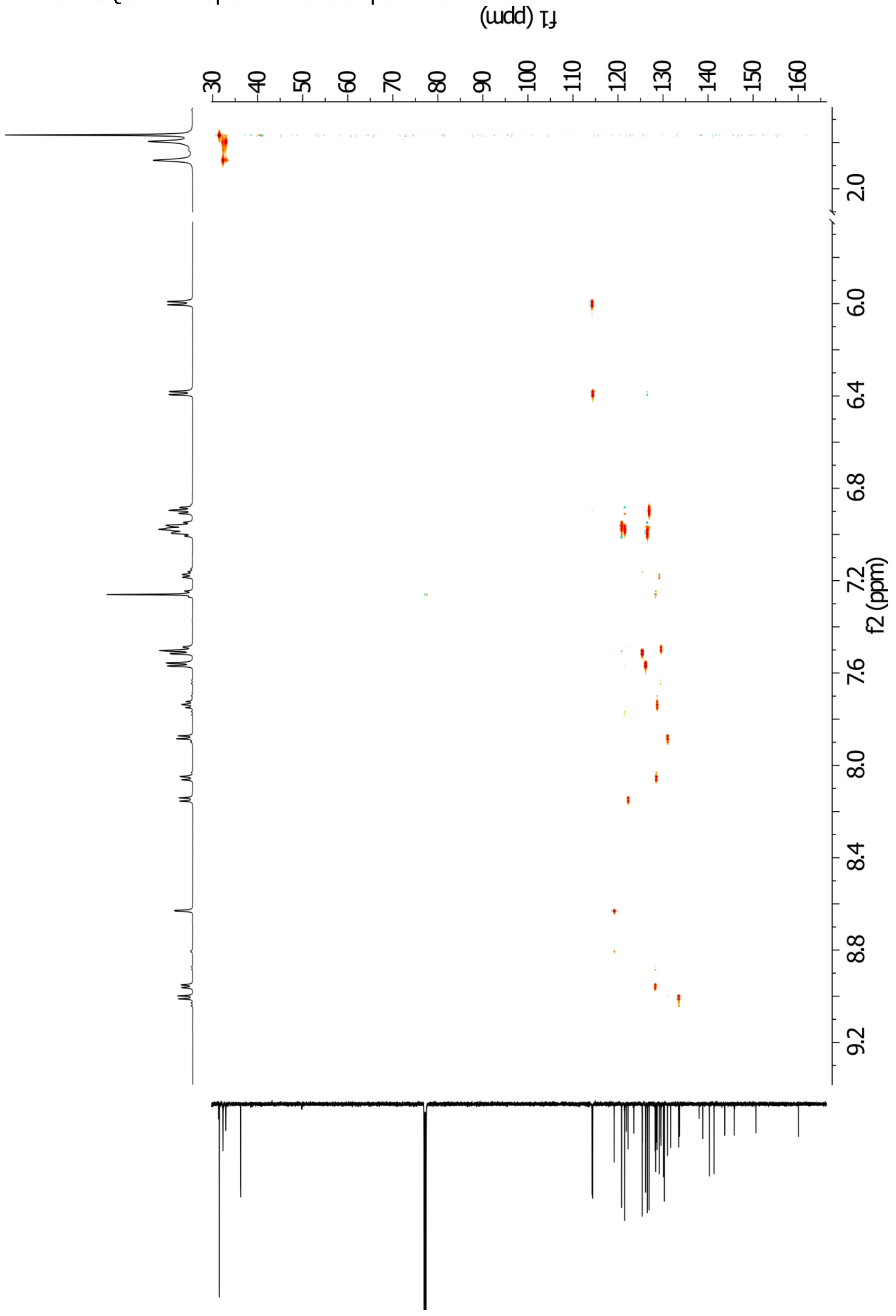

^{1}H-^{13}C HMBC NMR spectrum of compound **55.4**

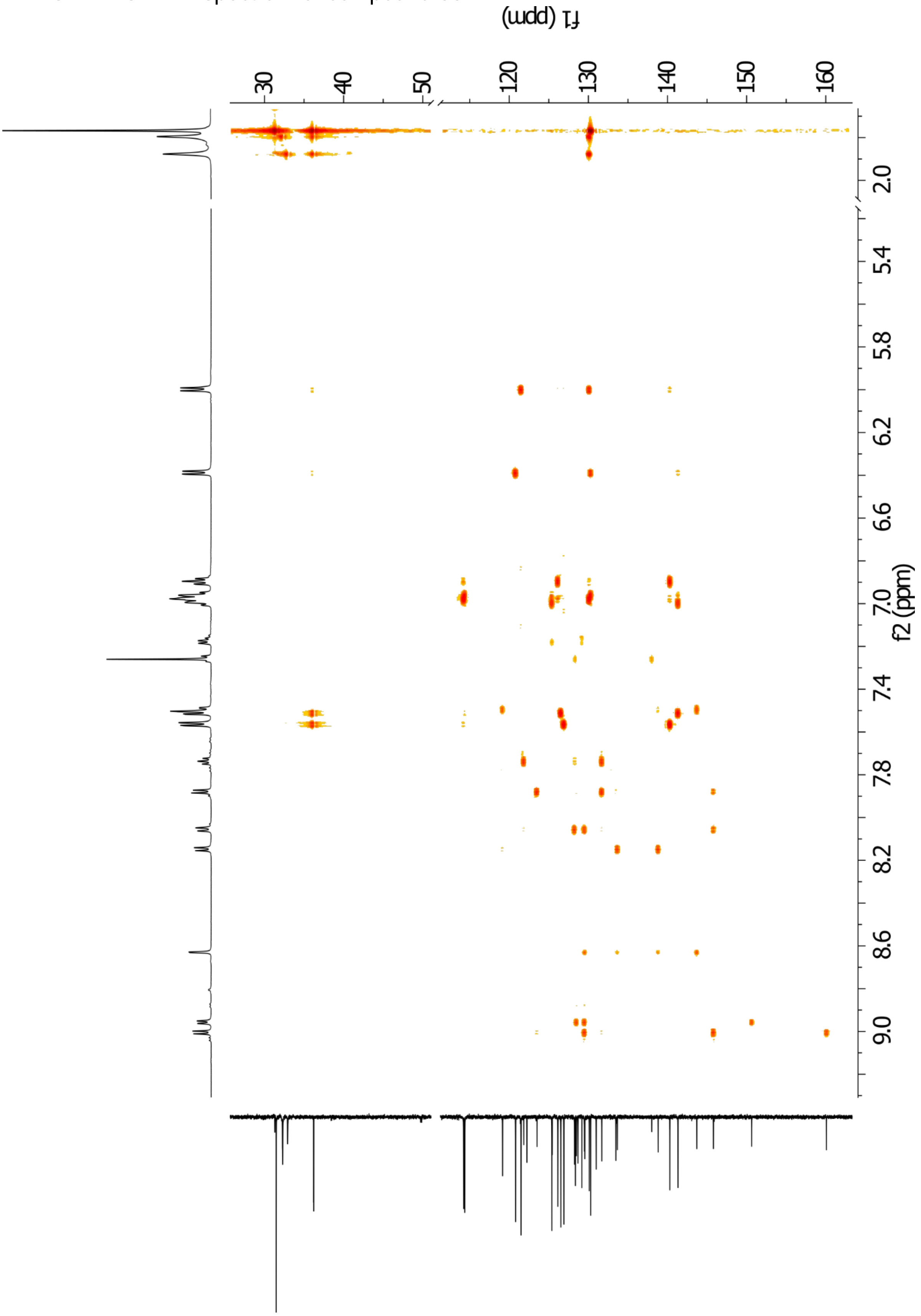

2D NMR assignments of **55.4**

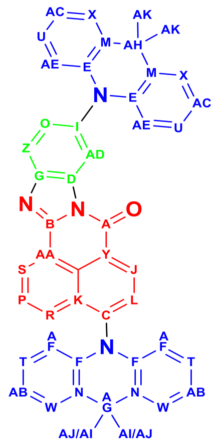

Isomer 4					
**Nr**	**Assignment**	**^{13}C Shift [ppm]**	**HSQC [ppm]**	**HMBC**	**COSY**
**1**	A	160.09		J	
**2**	B	150.63		S	
**3**	C	145.80		J, R, L	
**4**	D	143.70		AD, O	
**5**	E	141.33		X, U, AC, AE	
**6**	F	140.29		W, T, AB, AF	
**7**	G	138.82		AD, Z, O	
**8**	I	133.67		AD, Z, O	
**9**	J	133.48	9.00	L	L
**10**	K	131.69		J, S, R, L, P	
**11**	L	130.99	7.88	J	J
**12**	M	130.29		AE, AC	
**13**	N	130.11		AF, T, AB, W	
**14**	O	129.55	7.53 – 7.47	J, S, AD, R	AD, Z
**15**	P	128.68	7.74	L	S, R
**16**	R	128.49	8.06	S	P
**17**	S	128.24	8.96	P, R	P
**18**	T	126.90	6.90	W, AF	AF, AB
**19**	U	126.52	7.02 – 6.93	X	AE
**20**	W	126.14	7.56	T, AB, AF	AB
**21**	X	125.38	7.53 – 7.47	U, AC, AE	AC
**22**	Y	123.51		J, L	
**23**	Z	122.23	8.15		O
**24**	AA	121.84		J, R, P	
**25**	AB	121.49	7.02 – 6.93	AF	W, T
**26**	AC	120.80	7.02 – 6.93	AE	X
**27**	AD	119.16	8.63	Z, O	O
**28**	AE	114.38	6.39	X	U
**29**	AF	114.24	6.00	T, AB, W	T
**30**	AG	36.22		W, AF, AI/AJ	
**31**	AH	36.20		X, AE, AK	
**32**	AI	32.92	1.80	AJ	
**33**	AJ	32.30	1.88	AI	
**34**	AK	31.48	1.77	AK	

^{1}H NMR spectrum of compound **56.2**

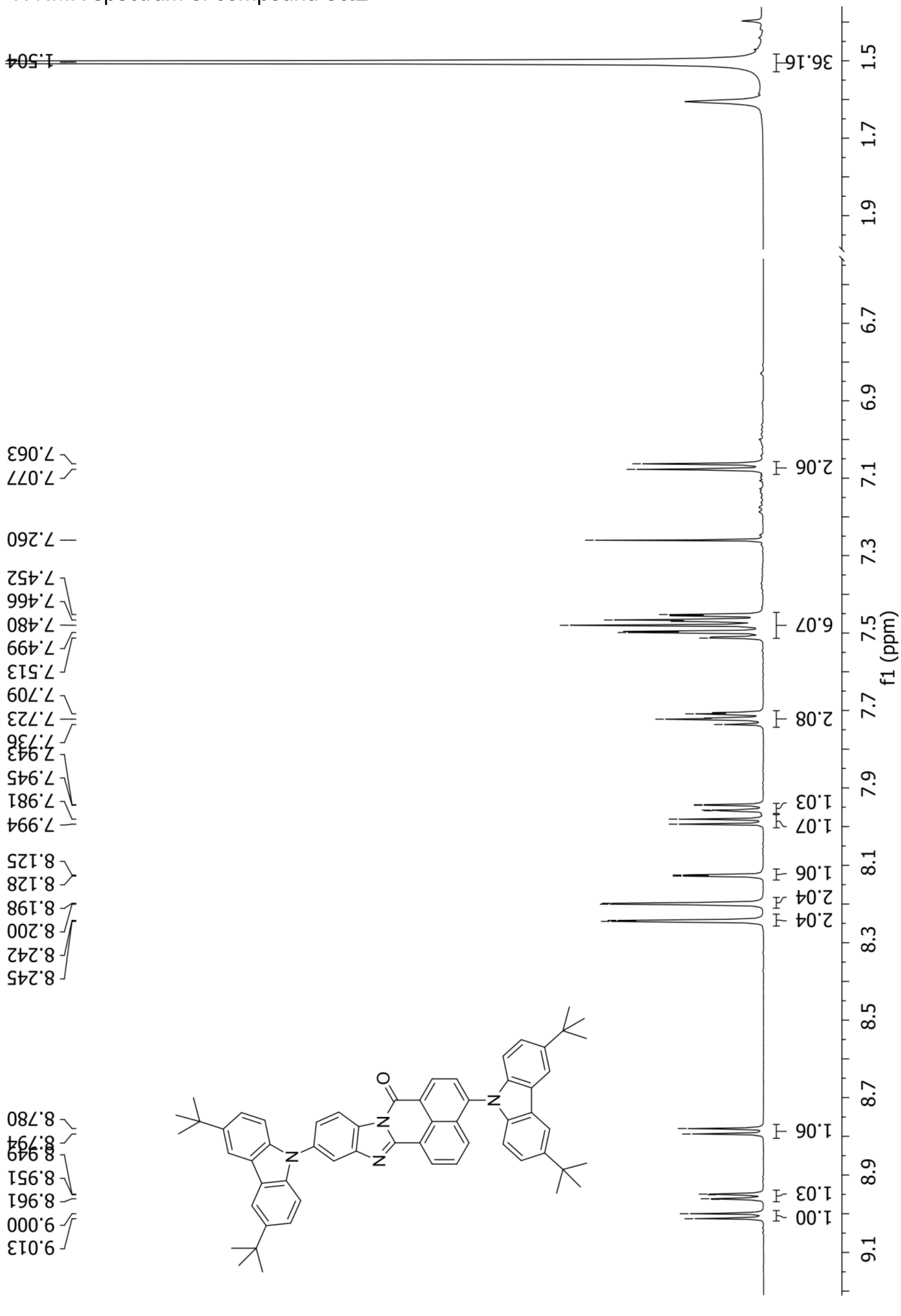

^{13}C NMR spectrum of compound **56.2**

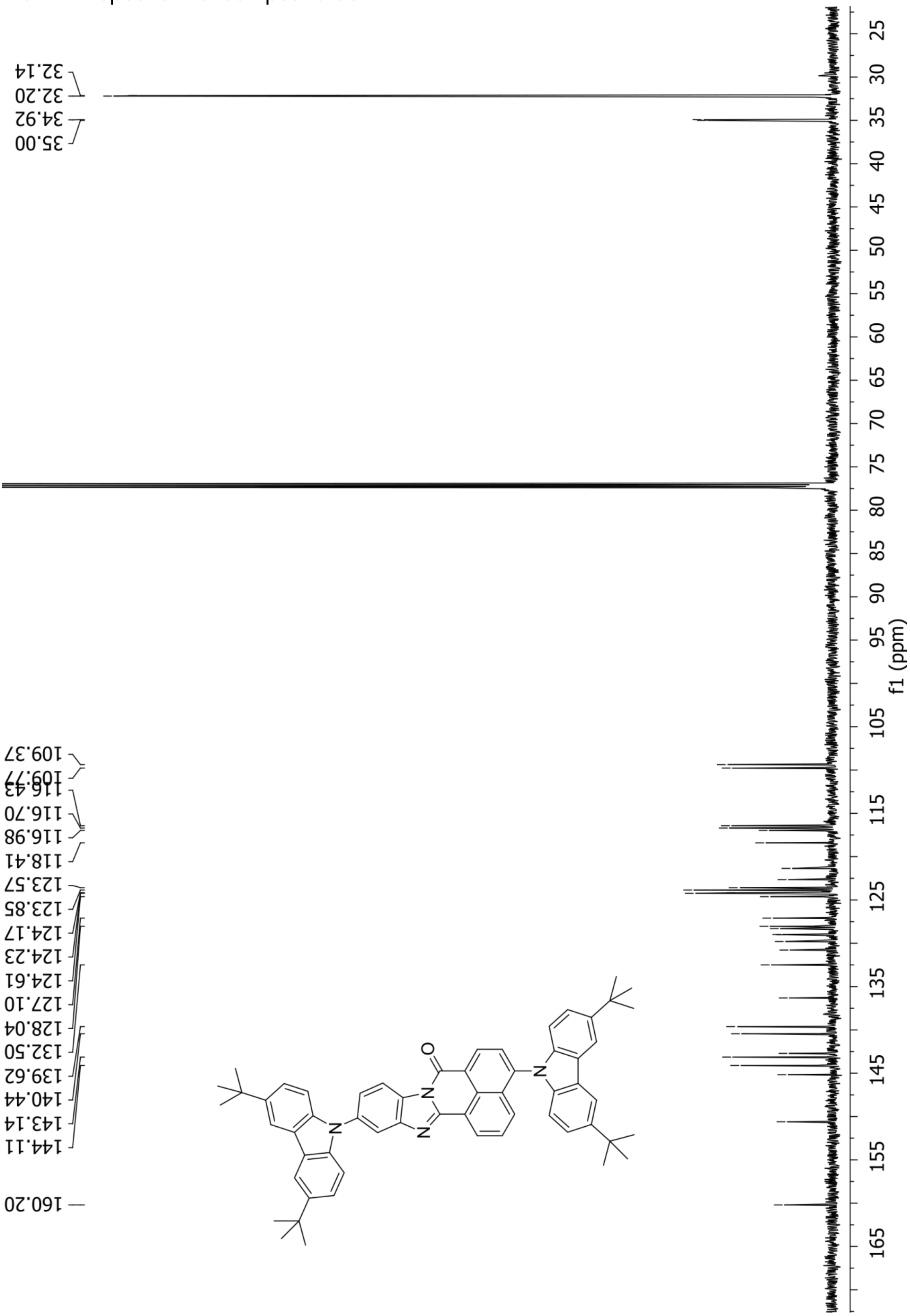

^{1}H-^{13}H COSY NMR spectrum of compound **56.2**

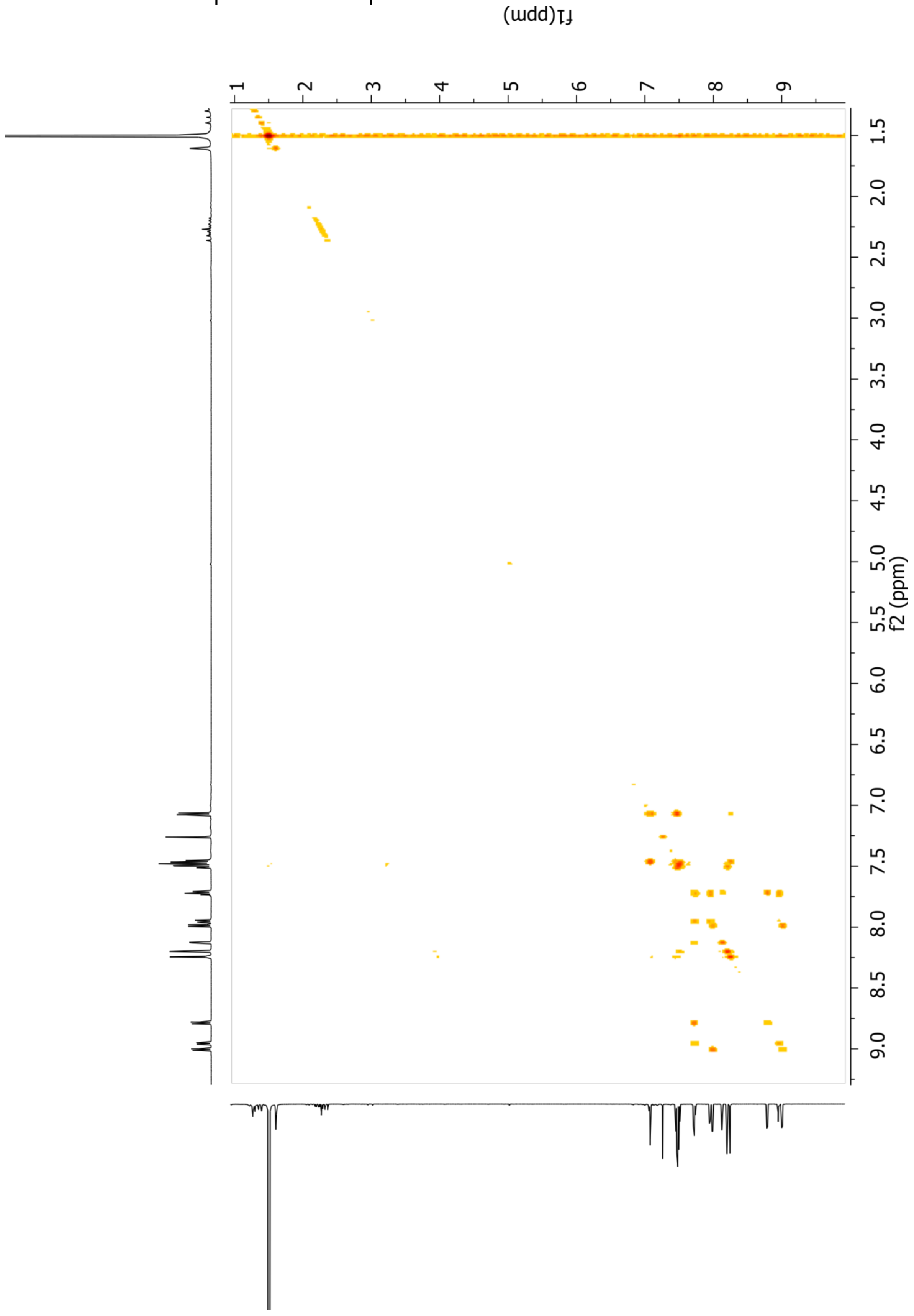

^{1}H-^{13}C HSQC NMR spectrum of compound **56.2**

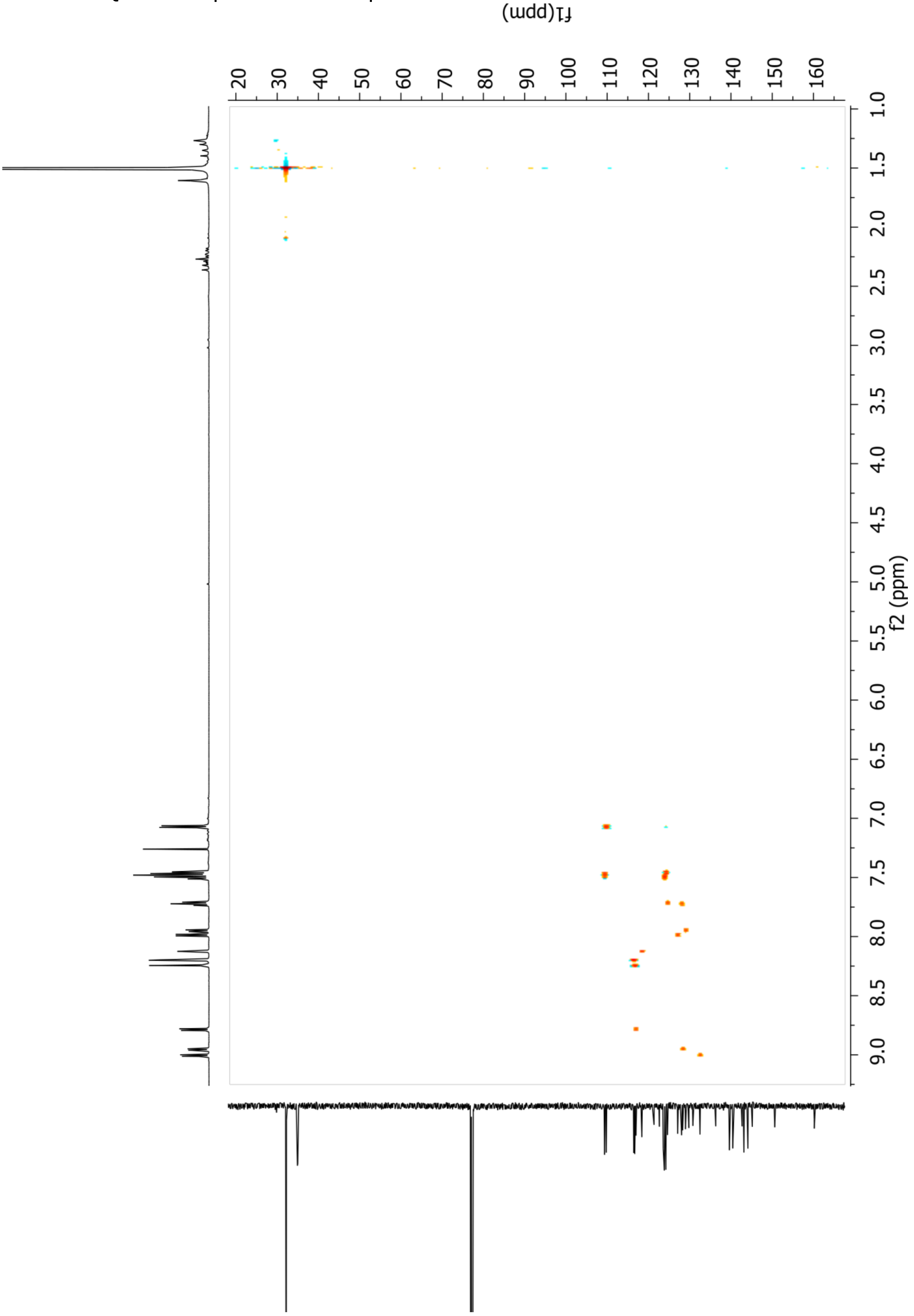

^{1}H-^{13}C HMBC NMR spectrum of compound **56.2**

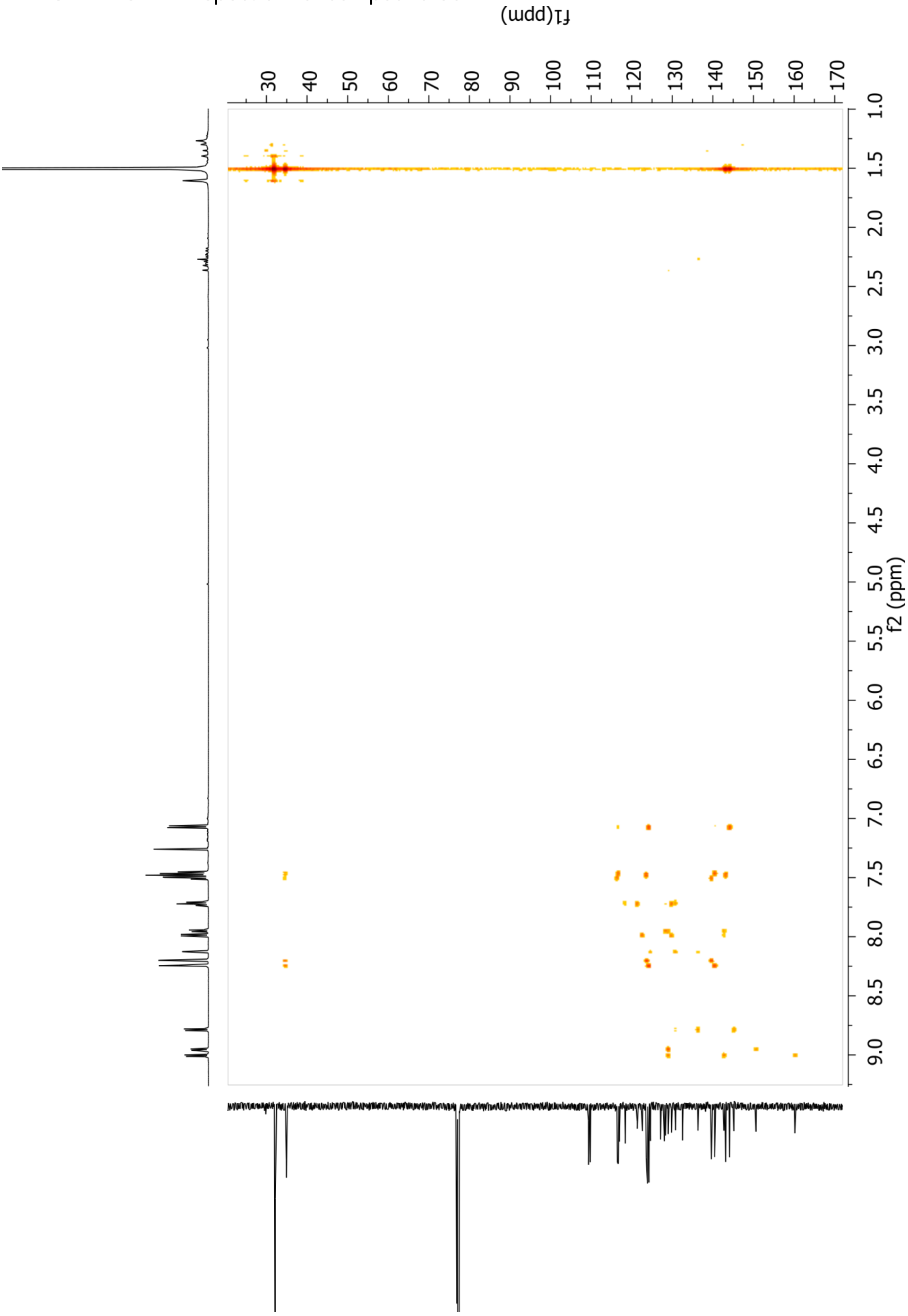

2D NMR assignments of **56.2**

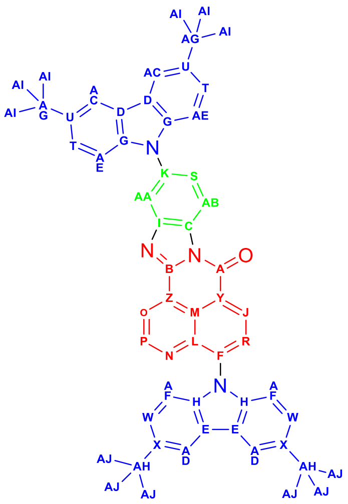

Isomer 2					
**Nr**	**Assignment**	**^{13}C Shift [ppm]**	**HSQC [ppm]**	**HMBC**	**COSY**
**1**	A	160.20		J, R	
**2**	B	150.61		O, P, N	
**3**	C	145.17		AB, AA, S	
**4**	D	144.11		AE, AI	
**5**	**E**	143.14		AF, AJ	
**6**	F	142.71		J, R, M	
**7**	G	140.44		AC, T	
**8**	**H**	139.62		AD, W	
**9**	I	136.31		AB, AA, S	
**10**	J	132.50	9.01	R	R
**11**	K	130.78		AB, AA, S	
**12**	L	129.79		P, R	
**13**	M	129.02		N	
**14**	N	128.99	7.95	P, J, O	P
**15**	O	128.30	8.96		P
**16**	P	128.04	7.74 – 7.70	O, N	O, N
**17**	R	127.10	7.99	J	J
**18**	S	124.61	7.73 – 7.70	AB, AA	AB, AA
**19**	T	124.23	7.52 – 7.44	AC	AE, AC
**20**	U	124.17		AC, AE	
**21**	**W**	123.85	7.52 – 7.44	AD	AF, AD
**22**	**X**	123.57		AF	
**23**	Y	122.65		J, R	
**24**	Z	121.36		P, N	
**25**	AA	118.41	8.13	AB, S	S
**26**	AB	116.98	8.79		S
**27**	AC	116.70	8.24	AE, T	T, AE
**28**	**AD**	116.43	8.20	W	W
**29**	AE	109.77	7.07	AC	T, AC
**30**	**AF**	109.37	7.52 – 7.44	AD	W
**31**	AG	35.00		AC, W, AI	
**32**	**AH**	34.92		AD, T, AJ	
**33**	AI	32.20	1.50		
**34**	**AJ**	32.14	1.50		

^{1}H NMR spectrum of compound **56.3**

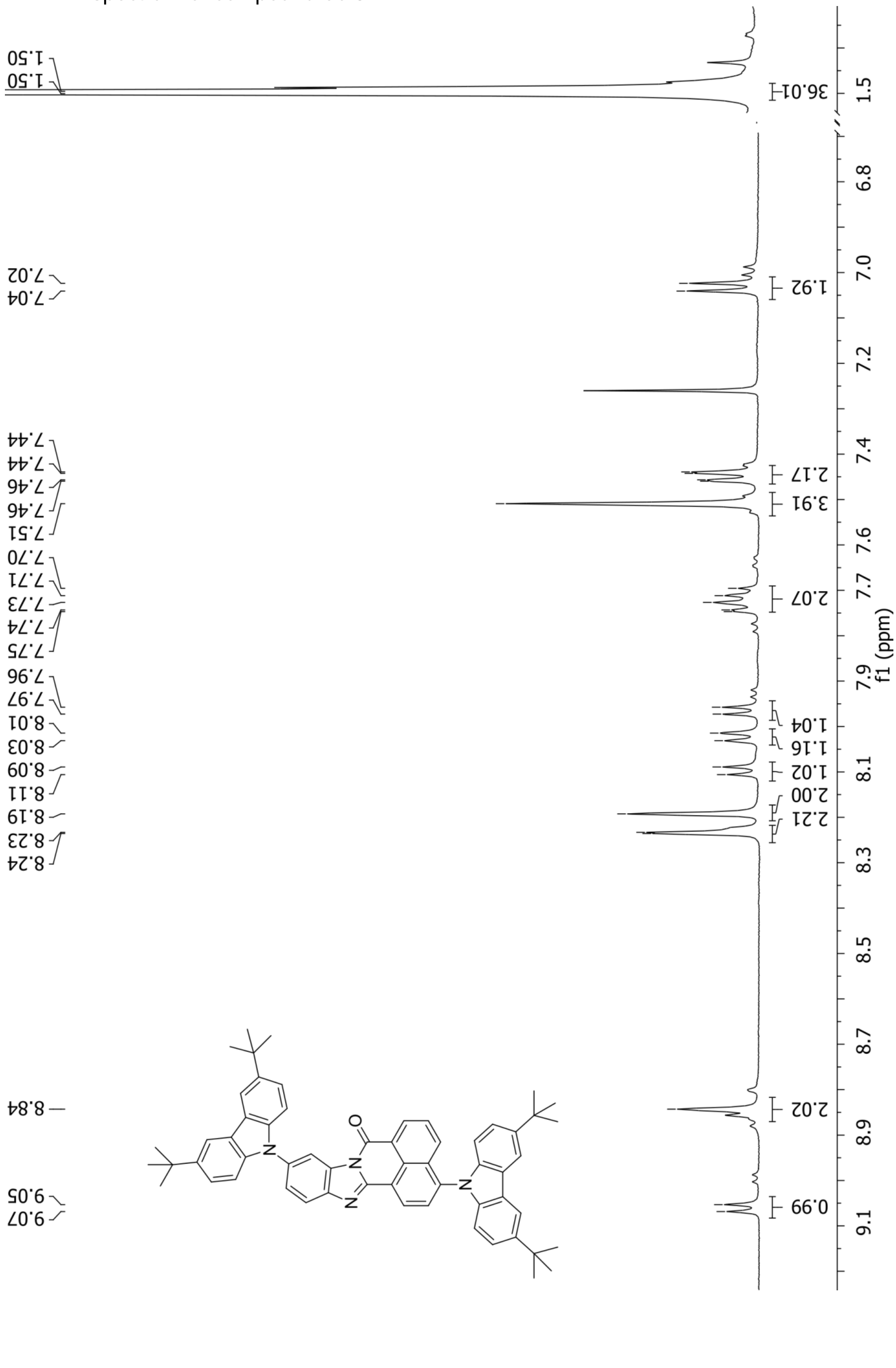

^{13}C NMR spectrum of compound **56.3**

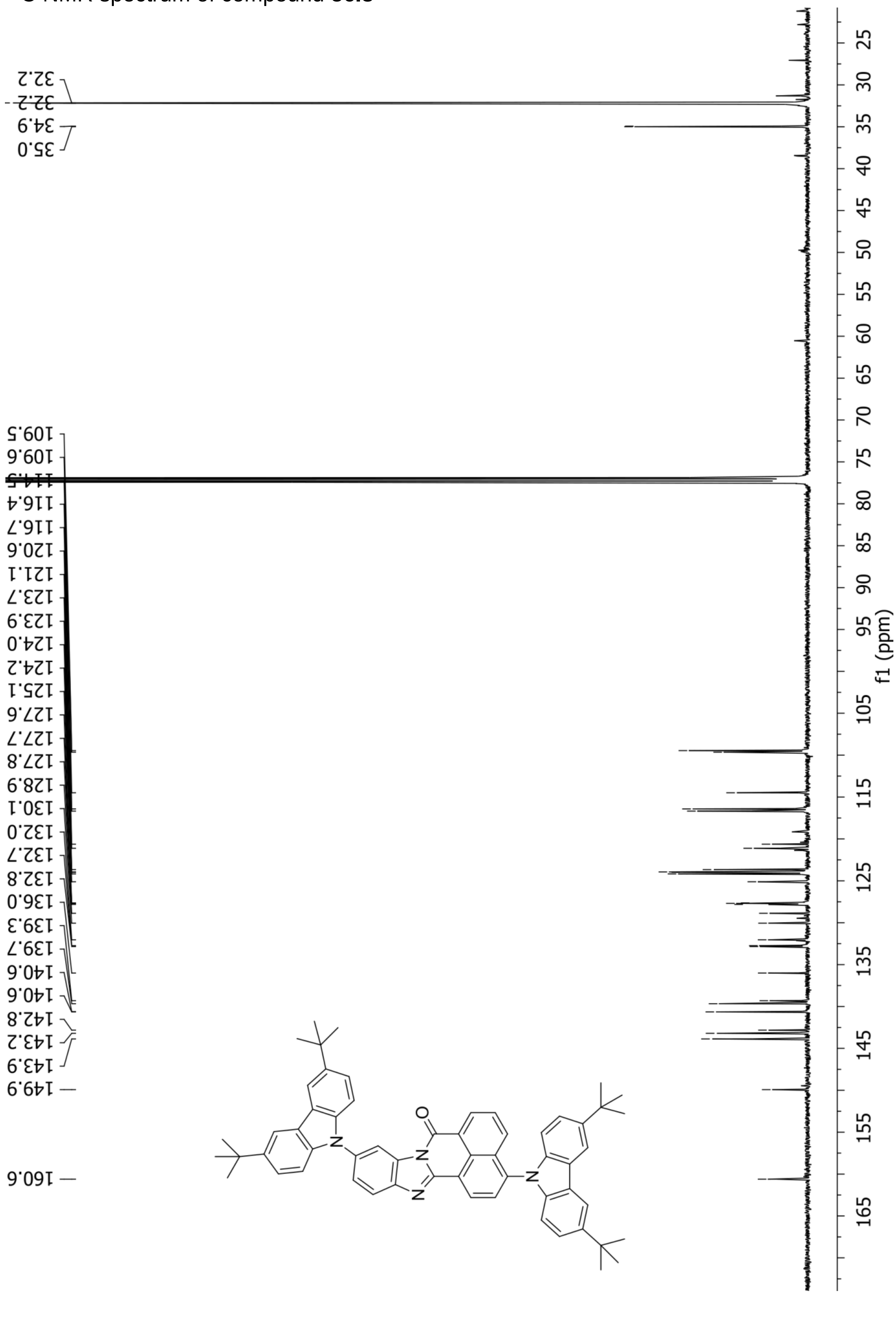

^{1}H-^{1}H COSY NMR spectrum of compound **56.3**

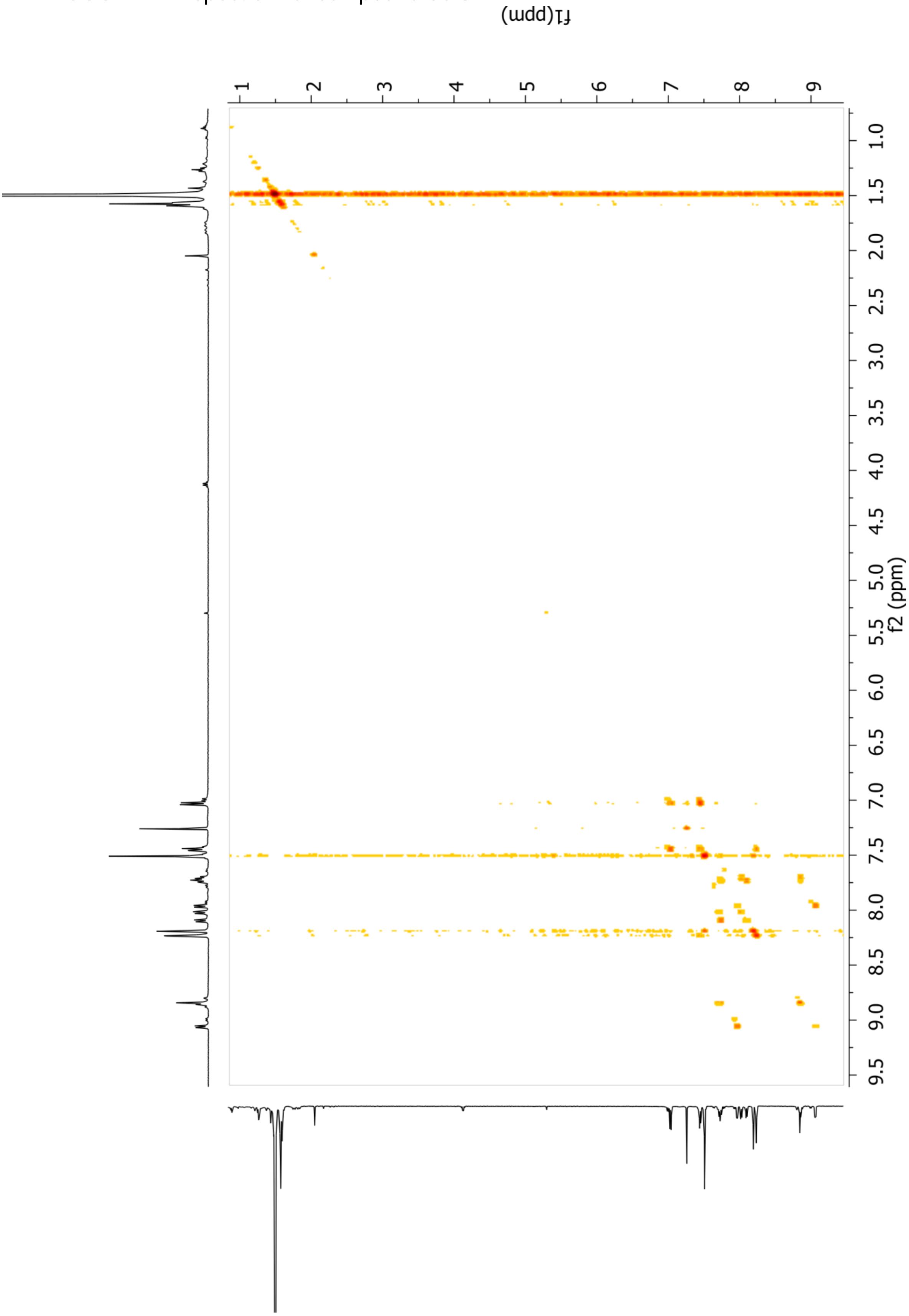

^{1}H-^{13}C HSQC NMR spectrum of compound **56.3**

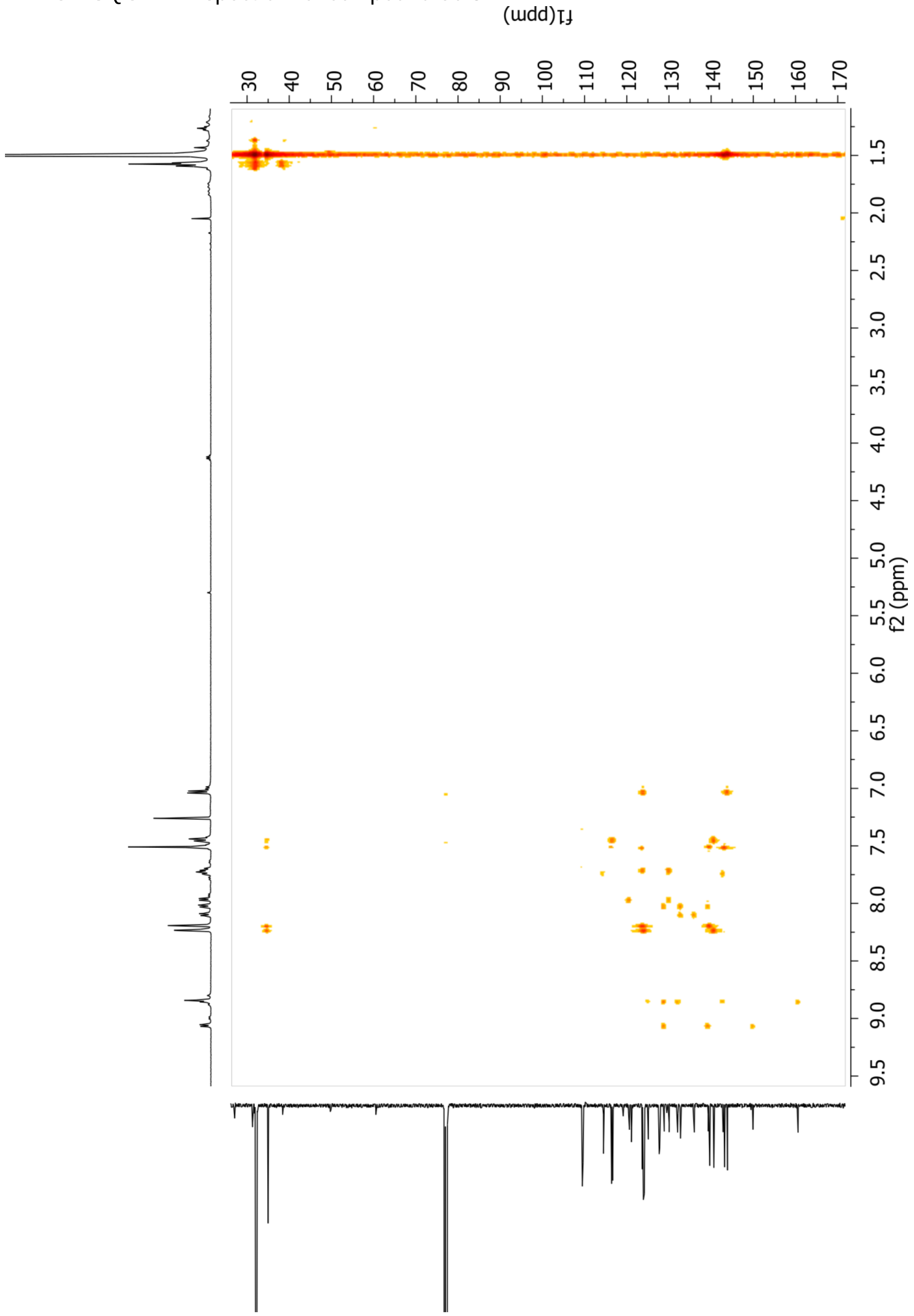

^{1}H-^{13}C HMBC NMR spectrum of compound **56.3**

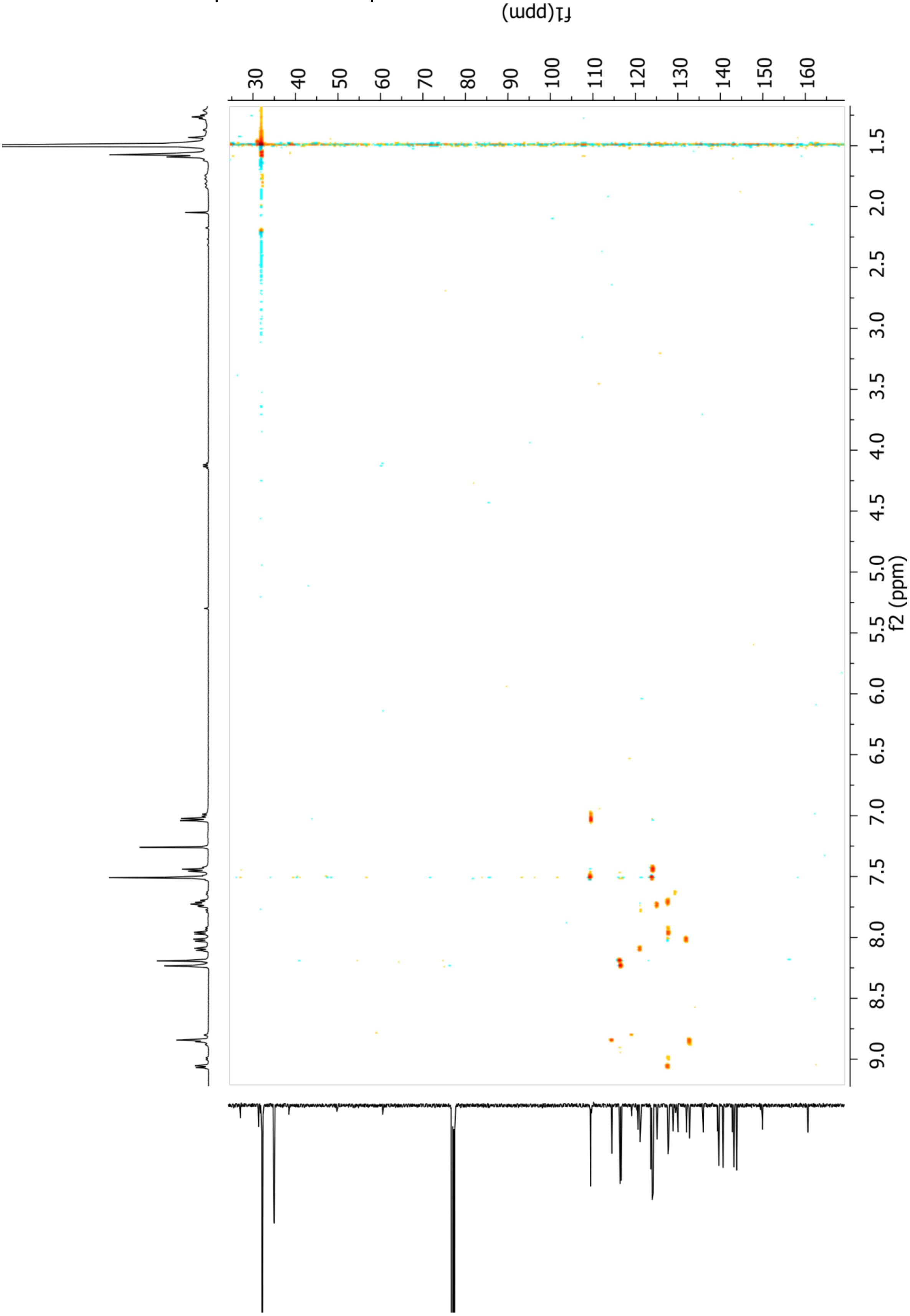

2D NMR assignments of **56.3**

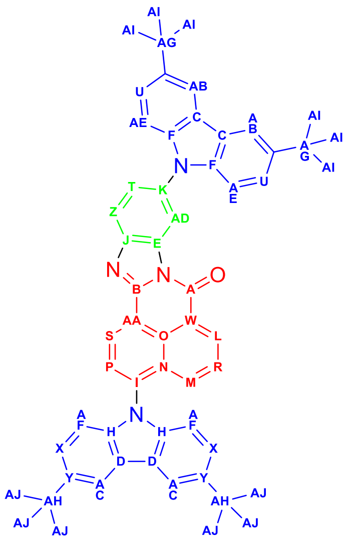

		Isomer 3			
**Nr**	**Assignment**	**^{13}C Shift [ppm]**	**HSQC [ppm]**	**HMBC**	**COSY**
**1**	A	160.61		L	
**2**	B	149.92		S	
**3**	C	143.87		AI, AE	
**4**	D	143.19		AJ, X	
**5**	E	142.83		AD, T	
**6**	F	140.65		AB, U	
**7**	H	139.66		AC, AF	
**8**	I	139.30		M, P, S	
**9**	J	136.01		AD, Z	
**10**	K	132.85		Z	
**11**	L	132.74	8.84	M	R
**12**	M	132.04	8.02	L	R
**13**	N	130.06		P, R	
**14**	O	128.87		S, L, M	
**15**	P	127.82	7.97		S
**16**	R	127.69	7.75 – 7.69		L, M
**17**	S	127.64	9.06		P
**18**	T	125.11	7.75 – 7.69	AD	AD, Z
**19**	U	124.18	7.45	AB	AB, AE
**20**	W	123.98		R	
**21**	X	123.93	7.51	AC	AC
**22**	Y	123.66		X	
**23**	Z	121.11	8.10		T
**24**	AA	120.62		P	
**25**	AB	116.67	8.23	U	U
**26**	AC	116.42	8.19	AF	AF
**27**	AD	114.49	8.84	T	T
**28**	AE	109.65	7.03		U
**29**	AF	109.46	7.51	AC	AC
**30**	AG	34.99		AB, U, AI	
**31**	AH	34.93		AC, AF, AJ	
**32**	AI	32.19	1.495		
**33**	AJ	32.16	1.500		

^{1}H NMR spectrum of compound **56.4**

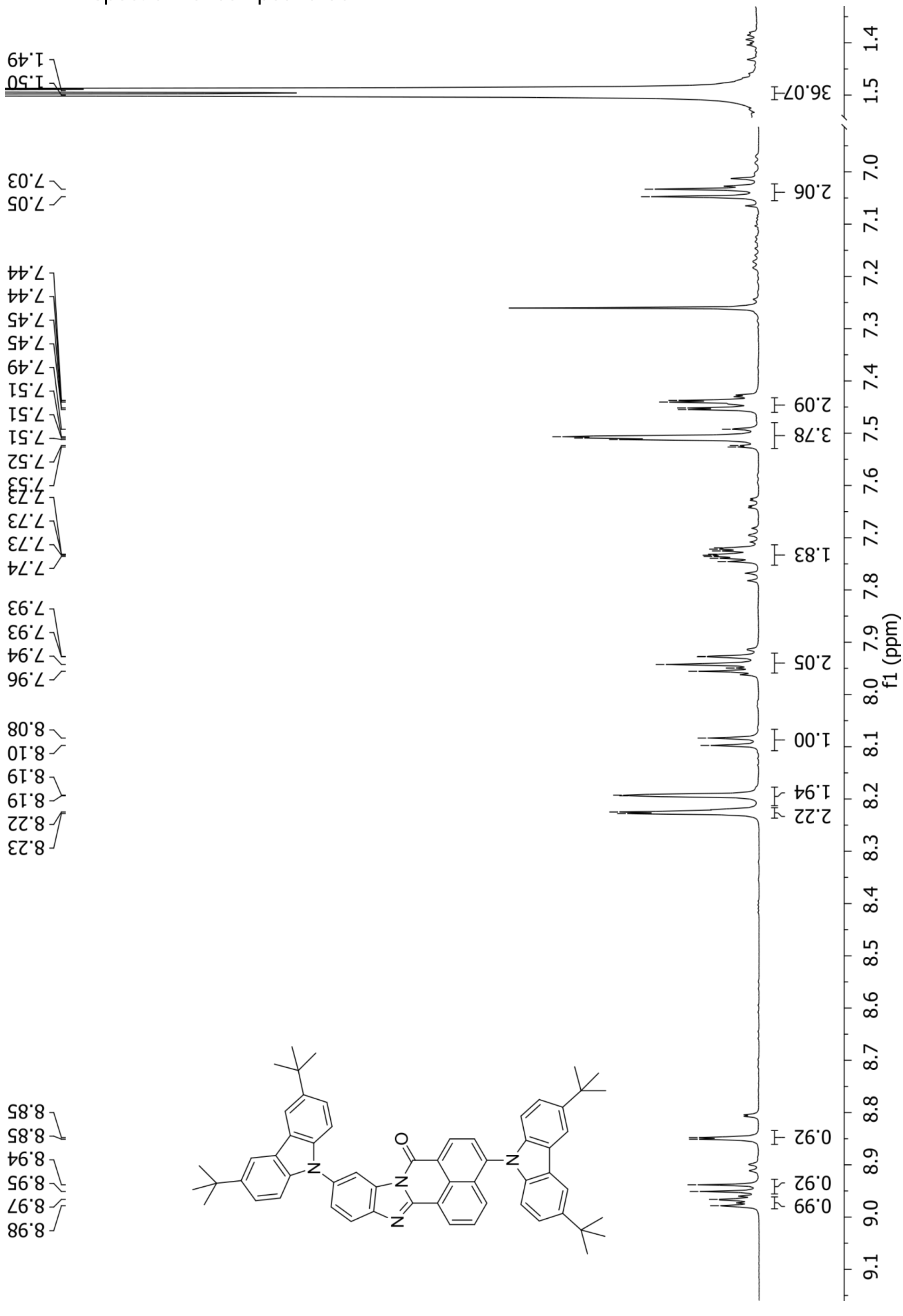

^{13}C NMR spectrum of compound **56.4**

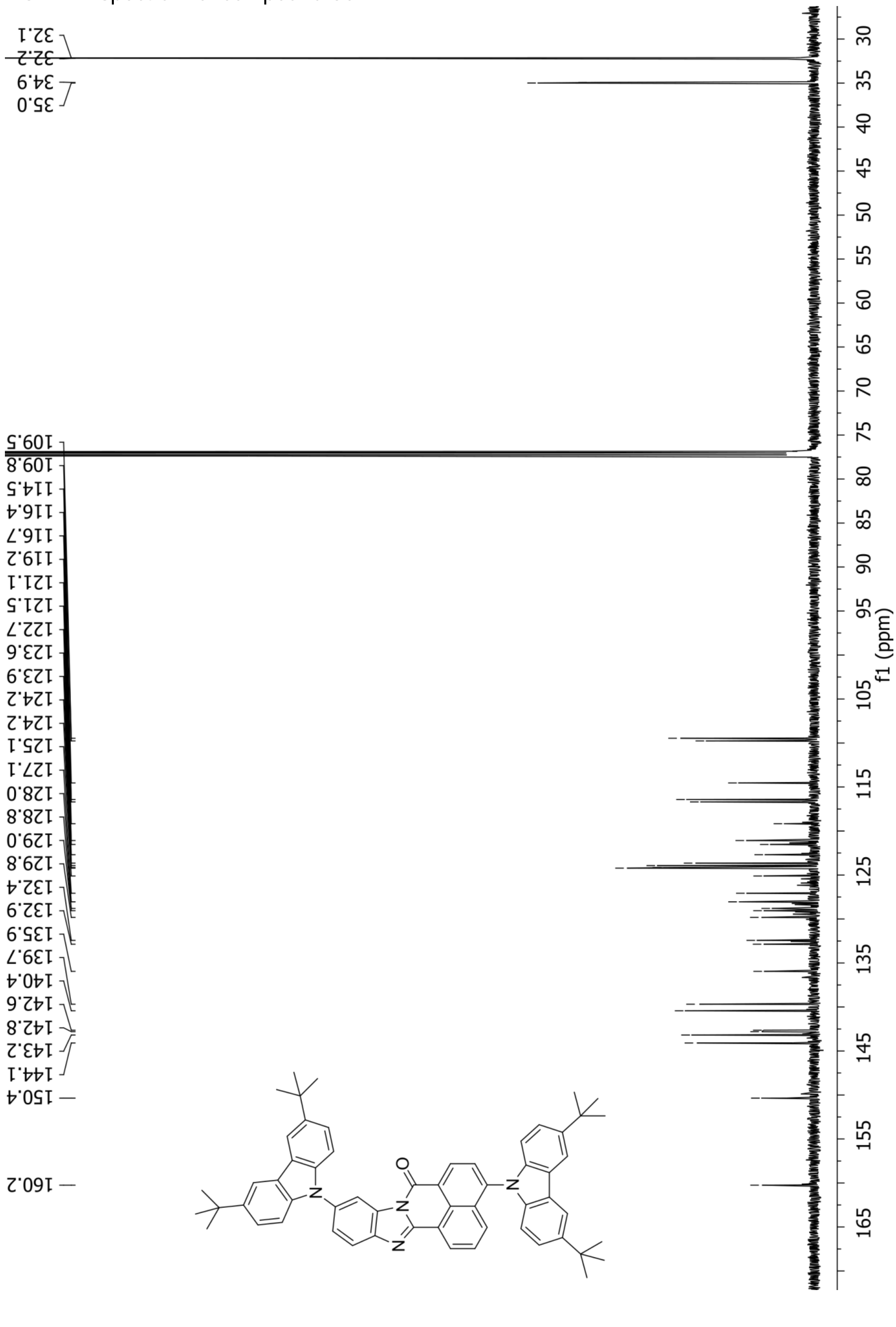

^{1}H-^{1}H COSY NMR spectrum of compound **56.4**

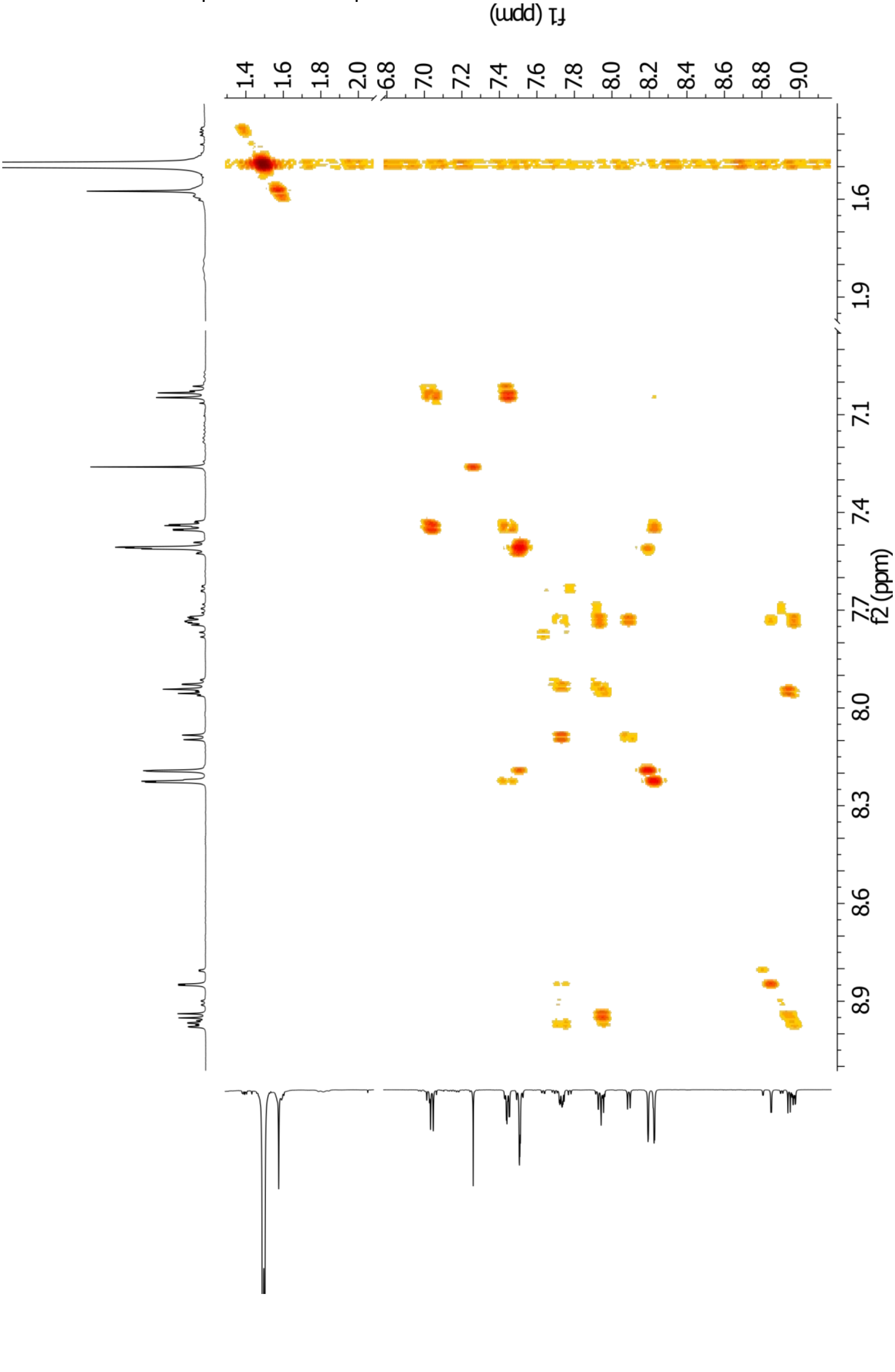

^{1}H-^{13}C HSQC NMR spectrum of compound **56.4**

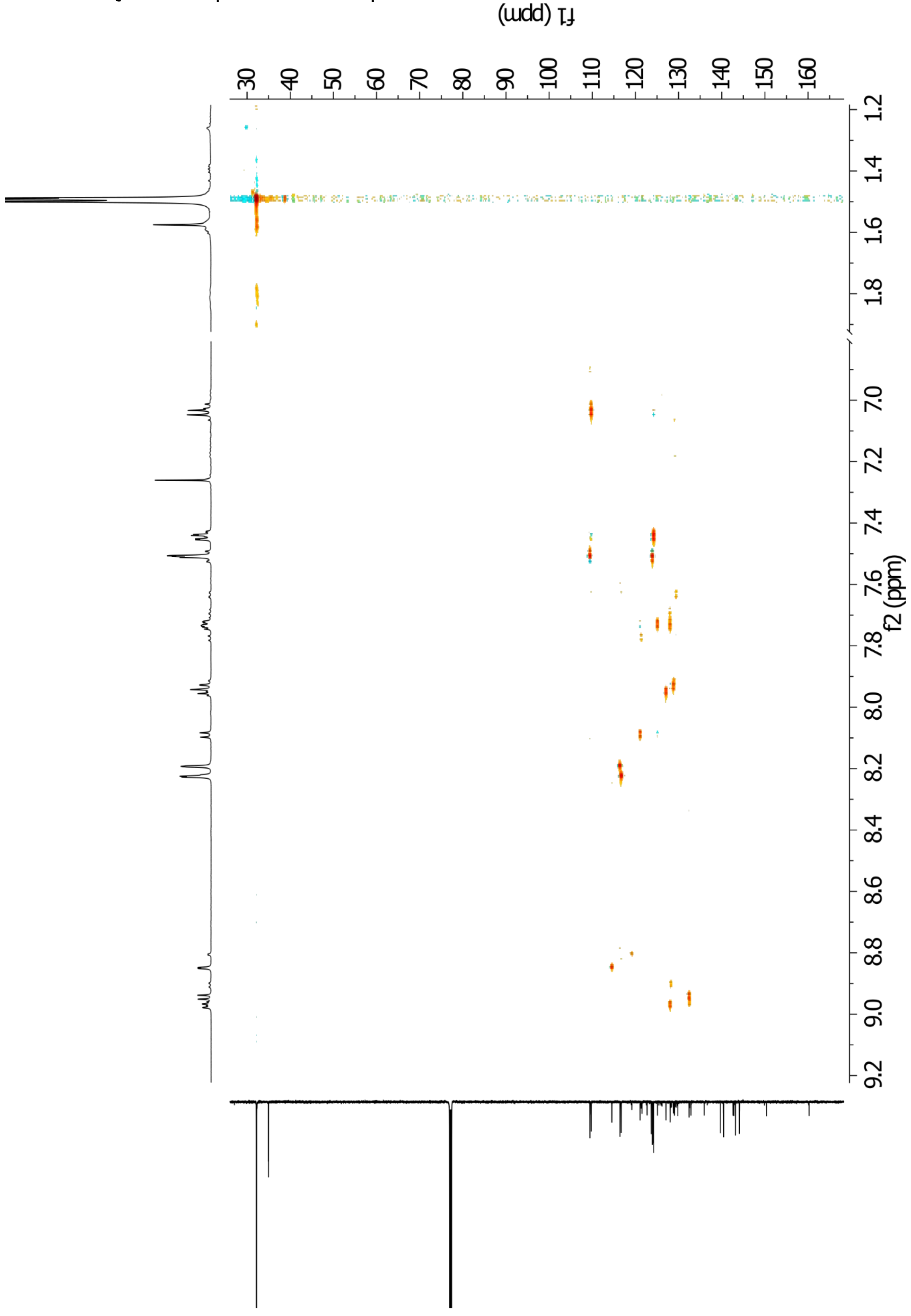

^{1}H-^{13}C HMBC NMR spectrum of compound **56.4**

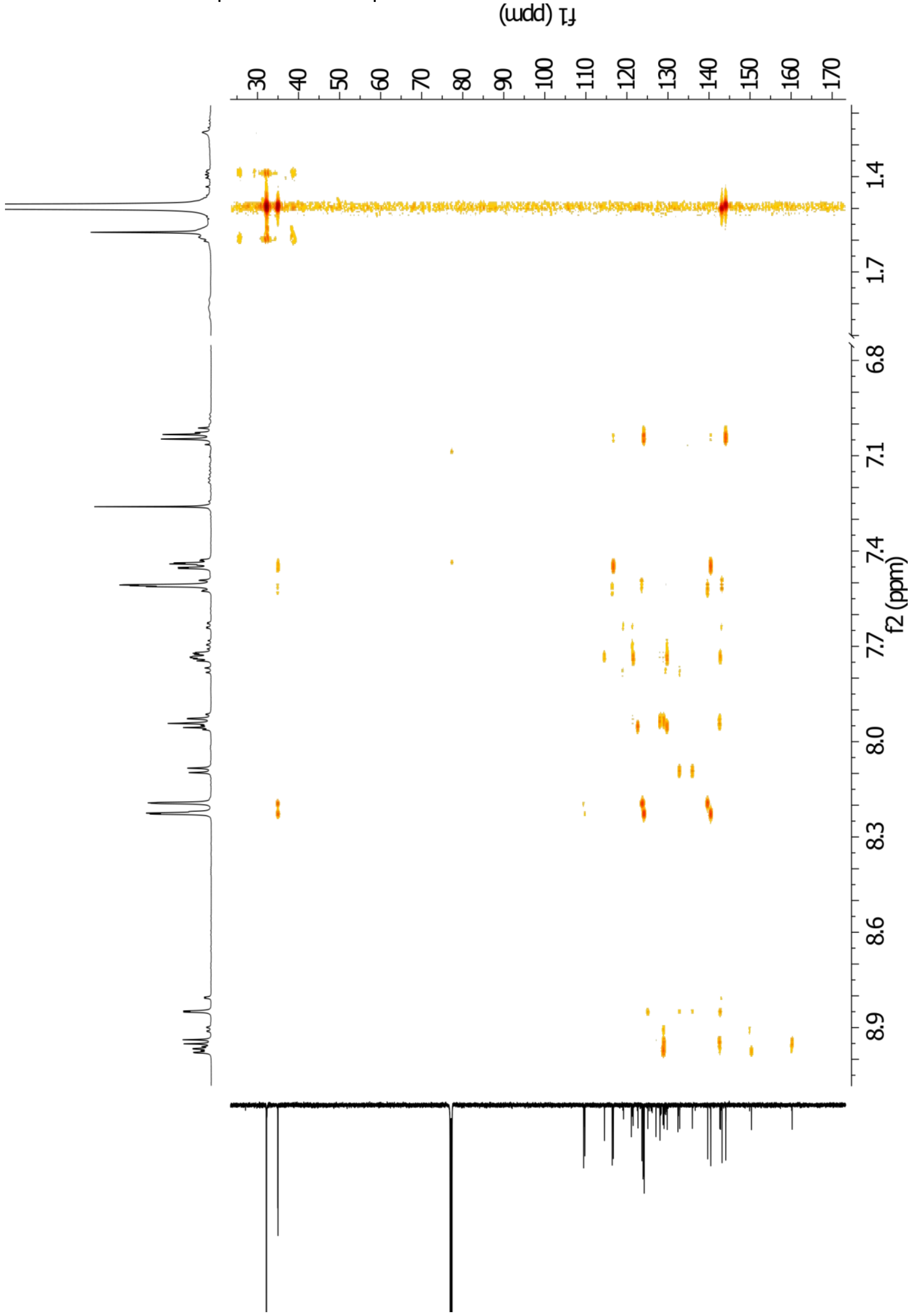

2D NMR assignments of **56.4**

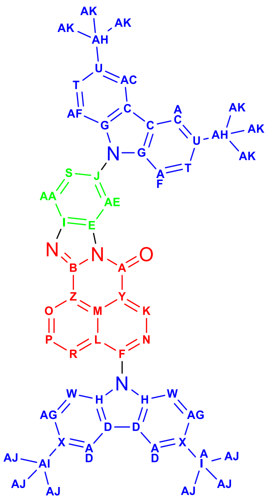

Isomer 4					
**Nr**	**Assignment**	**^{13}C Shift [ppm]**	**HSQC [ppm]**	**HMBC**	**COSY**
**1**	A	160.24		K	
**2**	B	150.35		O	
**3**	C	144.08		AF, AK	
**4**	D	143.18		AG, AJ	
**5**	E	142.80		AE, S	
**6**	F	142.63		K, R	
**7**	G	140.43		AC, T, AF	
**8**	H	139.68		AD, W	
**9**	I	135.94		AE, AA	
**10**	J	132.86		AE, AA	
**11**	K	132.43	8.94	N	N
**12**	L	129.82		N, P	
**13**	M	129.05		O, K, R	
**14**	N	128.80	7.96 – 7.92	P	K
**15**	O	128.06	8.97	P, R	P
**16**	P	128.04	7.75 – 7.71	O, R	O, R
**17**	R	127.07	7.96 – 7.92		P
**18**	S	125.10	7.75 – 7.71	AE, AA	AE, AA
**19**	T	124.22	7.45	AC	AC, AF
**20**	U	124.15		AF	
**21**	W	123.93	7.53 – 7.48	AD	
**22**	X	123.65		W, AG	
**23**	Y	122.70		N	
**24**	Z	121.52		P	
**25**	AA	121.08	8.09	S	S
**26**	AC	116.69	8.23	AF, T	T
**27**	AD	116.42	8.19		AG
**28**	AE	114.54	8.85	S	S
**29**	AF	109.76	7.04	AC	
**30**	AG	109.46	7.53 – 7.48	AD	W
**31**	AH	35.00		AC, T, AK	
**32**	AI	34.93		AD, W, AG, AJ	
**33**	AJ	32.20	1.50		
**34**	AK	32.14	1.49		

SI-8.3 Structural analysis based on 2D NMR spectra for compound 53.1

The reaction yields four different structural isomers, a consequence of using a substrate in the form of a mixture of isomers. The product mixture was separated through liquid column chromatography using a combination of toluene and ethyl acetate. To determine the structures of individual isomers, an analysis of NMR correlation spectra was conducted,

including ^{1}H-^{1}H COSY, ^{1}H-^{13}C HSQC, and ^{1}H-^{13}C HMBC. The numbering of the isomers corresponds to the order of their elution during chromatographic purification. Isomer 1 (**53.1**) is the least polar, and isomer 4 (**53.4**) is the most polar, with the longest elution time. Isomer 3 (**53.3**) exhibited very limited solubility. Consequently, only the ^{1}H NMR spectrum was measured for this derivative, and the structure of this isomer was determined using the elimination method, following the prior determination of the structures of isomers 1 (**53.1**), 2 (**53.2**), and 4 (**53.4**).

Selected correlations confirming the structure of isomer 1 (**53.1**) are presented below. The symbols refer to individual carbon atoms, as well as the hydrogen atoms associated with them.

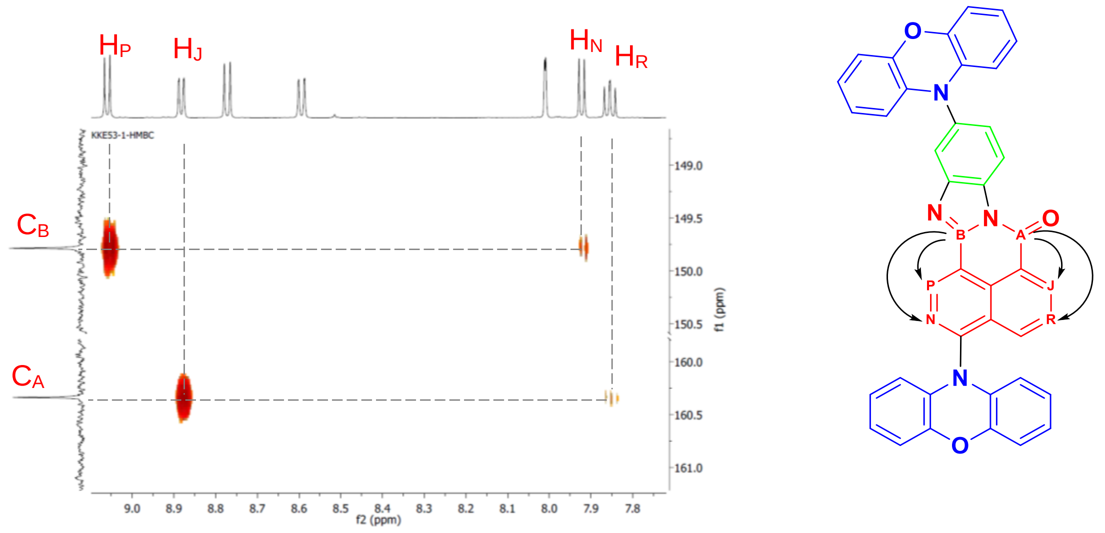

Figure S23. Fragment of the ^{1}H-^{13}C HMBC spectrum confirming correlations within the central part of the molecule.

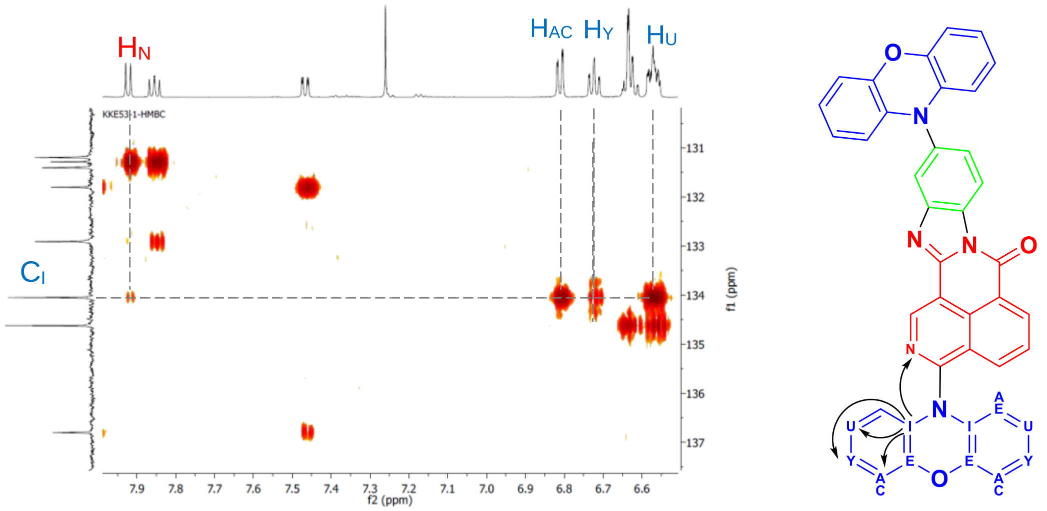

Figure S24. Fragment of the ^{1}H-^{13}C HMBC spectrum confirming the site of substitution of the phenoxazine within the naphthalene ring.

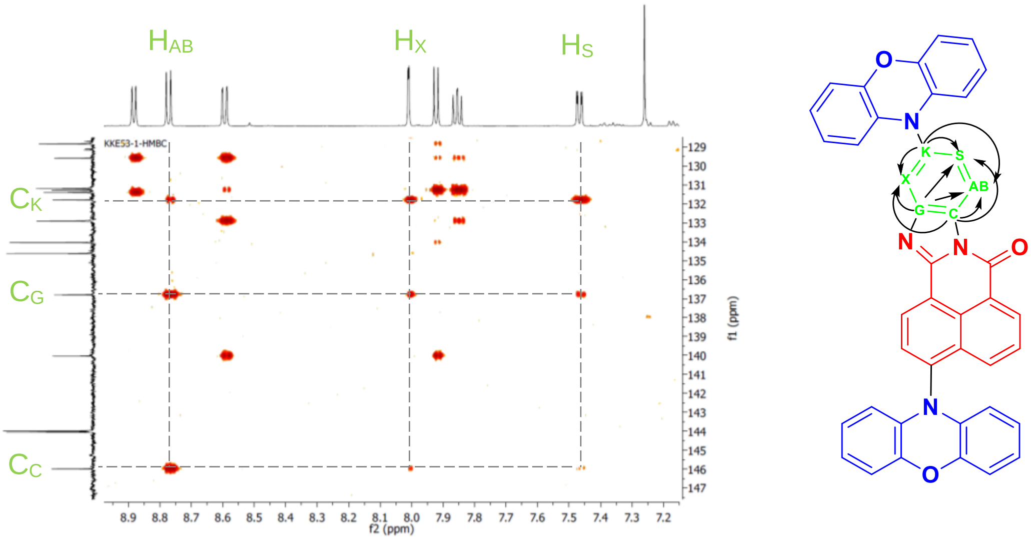

Figure S25. Fragment of the ^{1}H-^{13}C HMBC spectrum confirming the site of substitution of the phenoxazine molecule within the benzene ring.

Analogous spectral analyzes were performed for the remaining isomers. All correlations are presented in tables is section ***Copies of NMR spectra and 2D NMR signal assignments*** of this document. The structures of isomers 1-4 (**53.1-4)** are presented in Figure 26.

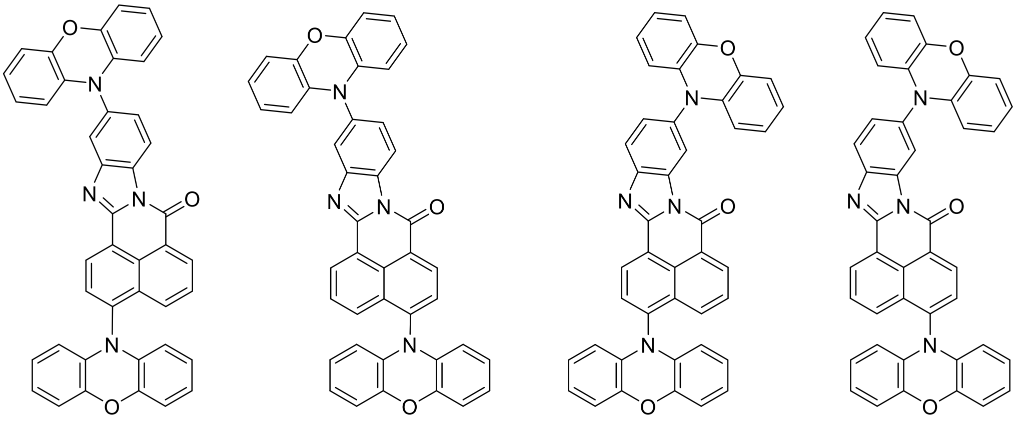

Isomer 1
53.1

Isomer 2
53.2

Isomer 3
53.3

Isomer 4
53.4

Figure S26. Structures of isomers 1-4 (**53.1-4**) identified from NMR correlation spectra.

Important note: Products of the reaction with 9,10-dihydrodibenzazepine, 9,9-dimethyl-10H-acridine and 3,6-di-tert-butylcarbazole exhibited the same structural behavior as phenoxazine derivatives. The same substitution pattern correlated with elution time was observed for all other groups of isomers.

References

1) Chai, J.-D.; Head-Gordon, M. Long-Range Corrected Hybrid Density Functionals with Damped Atom-Atom Dispersion Corrections. *Phys. Chem. Chem. Phys.* **2008**, *10*, 6615–6620. DOI: 10.1039/B810189B

2) Loos, P.-F.; Comin, M.; Blase, X.; Jacquemin, D. Reference Energies for Intramolecular Charge-Transfer Excitations. *J. Chem. Theory. Comput*. **2021**, *17*, 3666–3686. DOI: 10.1021/acs.jctc.1c00226

3) Mester, D.; Kállay, M. Charge-Transfer Excitations within Density Functional Theory: How Accurate Are the Most Recommended Approaches? *J. Chem. Theory Comput.* **2022**, *18*, 1646–1662. DOI: 10.1021/acs.jctc.1c01307

4) Gaussian 09, Revision D.01, Frisch, M. J.; Trucks, G. W.; Schlegel, H. B.; Scuseria, G. E.; Robb, M. A.; Cheeseman, J. R.; Scalmani, G.; Barone, V.; Mennucci, B.; Petersson, G. A.; Nakatsuji, H.; Caricato, M.; Li, X.; Hratchian, H. P.; Izmaylov, A. F.; Bloino, J.; Zheng, G.; Sonnenberg, J. L.; Hada, M.; Ehara, M.; Toyota, K.; Fukuda, R.; Hasegawa, J.; Ishida, M.; Nakajima, T.; Honda, Y.; Kitao, O.; Nakai, H.; Vreven, T.; Montgomery, J. A., Jr.; Peralta, J. E.; Ogliaro, F.; Bearpark, M.; Heyd, J. J.; Brothers, E.; Kudin, K. N.; Staroverov, V. N.; Kobayashi, R.; Normand, J.; Raghavachari, K.; Rendell, A.; Burant, J. C.; Iyengar, S. S.; Tomasi, J.; Cossi, M.; Rega, N.; Millam, J. M.; Klene, M.; Knox, J. E.; Cross, J. B.; Bakken, V.; Adamo, C.; Jaramillo, J.; Gomperts, R.; Stratmann, R. E.; Yazyev, O.; Austin, A. J.; Cammi, R.; Pomelli, C.; Ochterski, J. W.; Martin, R. L.; Morokuma, K.; Zakrzewski, V. G.; Voth, G. A.; Salvador, P.; Dannenberg, J. J.; Dapprich, S.; Daniels, A. D.; Farkas, Ö.; Foresman, J. B.; Ortiz, J. V.; Cioslowski, J.; Fox, D. J. Gaussian, Inc., Wallingford CT, 2009

5) Weigend, F.; Ahlrichs, R. Balanced Basis Sets of Split Valence, Triple Zeta Valence and Quadruple Zeta Valence Quality for H to Rn: Design and Assessment of Accuracy. *Phys. Chem. Chem. Phys.* **2005**, *7*, 3297−3305. DOI: 10.1039/b508541a
